# A light-fuelled nanoratchet shifts a coupled chemical equilibrium

**DOI:** 10.1038/s41565-021-01021-z

**Published:** 2021-12-16

**Authors:** Michael Kathan, Stefano Crespi, Niklas O. Thiel, Daniel L. Stares, Denis Morsa, John de Boer, Gianni Pacella, Tobias van den Enk, Piermichele Kobauri, Giuseppe Portale, Christoph A. Schalley, Ben L. Feringa

**Affiliations:** 1 Stratingh Institute for Chemistry, Center for Systems Chemistry and Zernike Institute for Advanced Materials, Faculty of Mathematics and Natural Sciences, University of Groningen, Groningen, the Netherlands; 2 Institut für Chemie und Biochemie der Freien Universität Berlin, Berlin, Germany; 3 Fritz-Haber-Institut der Max-Planck-Gesellschaft, Berlin, Germany; 4 Macromolecular Chemistry and New Polymeric Materials and Zernike Institute for Advanced Materials, Faculty of Mathematics and Natural Sciences, University of Groningen, Groningen, the Netherlands

**Keywords:** Chemistry, Photochemistry

## Abstract

Biological molecular machines enable chemical transformations, assembly, replication and motility, but most distinctively drive chemical systems out of-equilibrium to sustain life^1,2^. In such processes, nanometre-sized machines produce molecular energy carriers by driving endergonic equilibrium reactions. However, transforming the work performed by artificial nanomachines^3–5^ into chemical energy remains highly challenging. Here, we report a light-fuelled small-molecule ratchet capable of driving a coupled chemical equilibrium energetically uphill. By bridging two imine^6–9^ macrocycles with a molecular motor^10,11^, the machine forms crossings and consequently adopts several distinct topologies by either a thermal (temporary bond-dissociation) or photochemical (unidirectional rotation) pathway. While the former will relax the machine towards the global energetic minimum, the latter increases the number of crossings in the system above the equilibrium value. Our approach provides a blueprint for coupling continuous mechanical motion performed by a molecular machine with a chemical transformation to reach an out-of-equilibrium state.

## Abstract (web summary)

An artificial molecular machine was designed by coupling a chemical equilibrium to a photoresponsive molecular motor. Upon light illumination, the rotary movement of the motor performs work on the chemical equilibrium generating a far-from-equilibrium state.

## Main

Taking inspiration from the fundamental property of biological nanoscale machines governing transmission and directionality of motion to produce energy carriers and allowing precise mechanical functioning at the molecular level, recent years have witnessed remarkable advances towards the design of artificial molecular motors and machines (AMMs)^2–4,12–14^. Autonomous rotary and translational movement and dynamic control of assembly, transport and catalysis have been demonstrated using molecular motors, as well as amplification, propagation and coupling of motion^3,5,15–19^. AMMs can drive the directional movement of a secondary geared unit^19,20^, transport and assemble molecular fragments in a specific manner^17,21^, actively pump molecular entities^22–25^ or transduce motion from the molecular to the macroscopic scale^26,27^. However, in order to address the fundamental question of how to shift a chemical reaction out of its equilibrium state and develop AMMs that can drive endergonic chemical processes, we need to find ways to couple a chemical (equilibrium) reaction to the continuous motion of a molecular machine^28–30^.

Herein, we describe the design and operation of an artificial, light-fuelled molecular rotary motor that can drive a coupled chemical equilibrium energetically uphill.

**Fig. 1:**
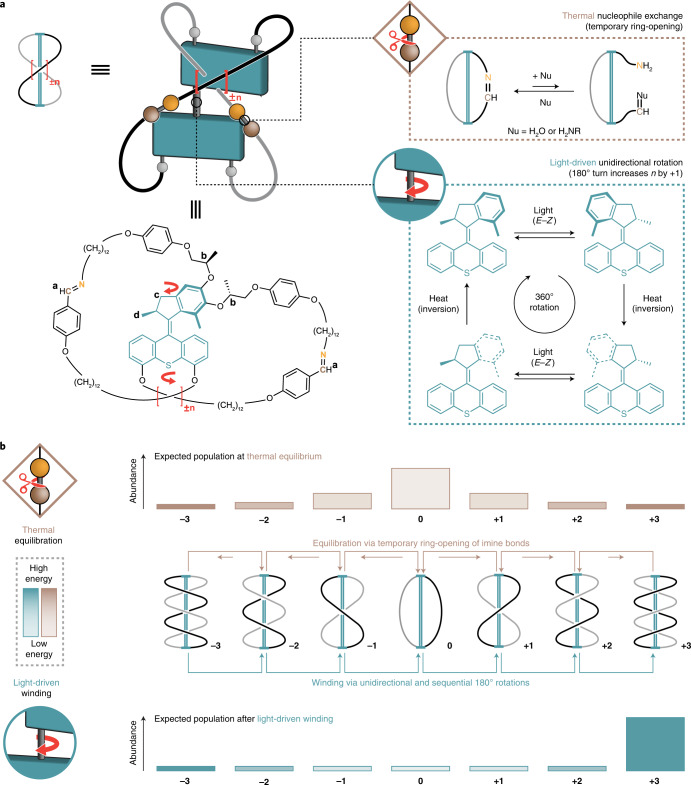
Working principle of AMM **±n**. **a**, A photoresponsive molecular motor is constrained by two imine macrocycles, allowing AMM **±n** to adopt distinct topologies by forming twists in the strands of the bicyclic molecule. Crossings can be established by either thermal nucleophile exchange of the imines (brown), leading to temporary ring-opening of one macrocycle, or by light-driven unidirectional rotation (blue) via photochemical-induced *E–Z* isomerization and subsequent thermal helix inversion (blue), which increases the number of chiral crossing per half-turn by **+1** in the case of (*S,R,R*) configured **±n**. **b**, Thermal equilibration (brown) takes place by temporary ring-opening of the imines and will populate all accessible, distinct topological states so that the Gibbs free energy of the system is minimized. The expected population is indicated by the brown bar diagram. Light-driven winding (blue) twists the bicyclic molecule in a unidirectional and sequential manner, increasing the number of crossings in the system above its equilibrium level until mechanical resistance in the system hinders further winding. The expected population is indicated by the blue bar diagram. The maximum number of crossings that was experimentally observed is **±3**. Lighter colour refers to lower energy, whereas darker colour refers to higher energy. Thermal nucleophile exchange (Fig. 1a, brown) leads to restoration of the initial equilibrium.

Our AMM **±n** (Fig. 1a; *n* represents the number of twists, while ± describes the chirality of the crossing; the (*S,R,R*) configured AMM ±**n** shown in Fig. 1a induces a positive (+) crossing per half-turn)^31^ consists of two complementary parts: two imine macrocycles bridged by a light-driven molecular rotary motor, connecting the contrarotating top and bottom halves of the central motor unit. This bridged bicyclic system can adopt several topologies by forming *n* crossings between the two macrocycles via two orthogonal pathways: a photochemical and a thermal one (Fig. 1a). Basically, we combine two fundamental dynamic functions of (1) a molecular rotary motor^10,11^ and (2) dynamic covalent chemical bond formation^6–9^. The photochemical process takes advantage of a light-driven rotary motor to deliver energy to the system. Our design is based on a second-generation molecular motor^10^ in which the central olefinic bond, connecting the top (indane rotor part) and bottom halves (thioxanthene stator part), functions as the rotary axle. Several intrinsic features, including the combination of two stereogenic elements, that is, the helical structure and the stereogenic centre, stilbene-type *E–Z* photoisomerization and thermal helix inversion, allow for unidirectional light-driven rotary motion. Building on dynamic covalent chemistry^6–9^, we explore reversible imine bond formation in combination with increasing strain in the system via the rotary motor.

In the thermal pathway, the imine bonds can undergo a reversible intermolecular exchange reaction with a nucleophile^6–9^, leading to a temporary ring-opening of the bicyclic compound (Fig. 1a, brown). In this open form, the side arms can slip through or undergo an intramolecular imine exchange with the other macrocycle to generate an entangled structure. Subsequent ring-closure by intramolecular imine formation fixes the twists in the system, thus reforming the bicyclic molecule and leading to the formation of distinct topological isomers. Since the imine exchange is a dynamic and reversible process that is constrained by microscopic reversibility, the minimization of the Gibbs free energy of the system dictates the population of distinct topological states. The population of these distinct states at thermal equilibrium is shown in Fig. 1b, top, with the expected abundance indicated in brown. It should be emphasized that, in the absence of light, the motor moiety is merely serving as a rigid unit to prevent the macrocycles from unwinding in a mechanical manner.

In the photochemical pathway, the motor unit performs a unidirectional 360° rotation around its central double bond, which is solely driven by light energy and therefore not constrained by microscopic reversibility but by the Bose–Einstein relations for absorption and emission of photons. This involves a four-step rotary cycle, that is, two photochemical *E–Z* isomerizations converting a stable to a metastable isomer each followed by a thermal helix inversion (Fig. 1a, blue). Upon illumination with ultraviolet (UV) light, its direction of rotation is governed by the methyl group at the stereogenic centre in the allylic position of the overcrowded alkene (Fig. 1a)^10,11^. This transduction from Euclidean chirality into directed movement enables the mechanical formation of chiral twists within the bicyclic molecule. A positive (+) crossing is induced by every 180° turn of the motor until winding is hindered by mechanical resistance in the system. The molecular motor increases the number of entanglements (strain build-up) in the bicyclic system far from its equilibrium level, performing chemical work on a coupled chemical equilibrium in a topologically stereoconvergent manner, thereby acting as a light-fuelled ratchet (see Supplementary Fig. 42 and the Supplementary Information for an in-depth discussion). The far-from-equilibrium distribution upon light-driven winding is shown in Fig. 1b, bottom, with the expected abundance indicated in blue. The system can return to its equilibrium state by thermal nucleophile-promoted imine exchange (Fig. 1a, brown).

To allow multiple entanglements in AMM **±n**, the imine groups were embedded in two large rings, each featuring two flexible dodecyl hydrocarbon chains, which bridge the upper rotor and lower stator parts of the rotary motor. In order to gain access to both contrarotating diastereomers (*S,R,R*) and (*R,R,R*) **±n** (unless otherwise stated, (*S,R,R*) **±n** was used for all experiments) of our optically active second-generation motors, we utilized a versatile method to access the stereoisomers developed by Giuseppone et al.^27^. After post-functionalizing the rotor part with two azide groups and the stator part with two aldehydes, in situ macrocyclization was accomplished by aza-Wittig reactions using triphenylphosphine.

By choosing this specific substitution pattern, a selective connection between the rotor and stator parts, and thus winding upon rotation, is ensured^27^. The conversion to the imine was followed by proton nuclear magnetic resonance (^1^H-NMR) spectroscopy (typically 5–10% of motors are mono cyclized, Supplementary Fig. 1). The system was further characterized by one-dimensional (1D) and two-dimensional (2D) NMR (Supplementary Figs. 1–22 and Supplementary Tables 1 and 2) and circular dichroism (CD) spectroscopy (Supplementary Figs. 23–25), small angle X-ray scattering (SAXS) (Supplementary Figs. 39 and 40 and Supplementary Table 3), mass spectrometry (MS) and ion mobility (IM) (Supplementary Fig. 36). Our data show that the formation of oligomers under our experimental conditions is negligible.

**Fig. 2:**
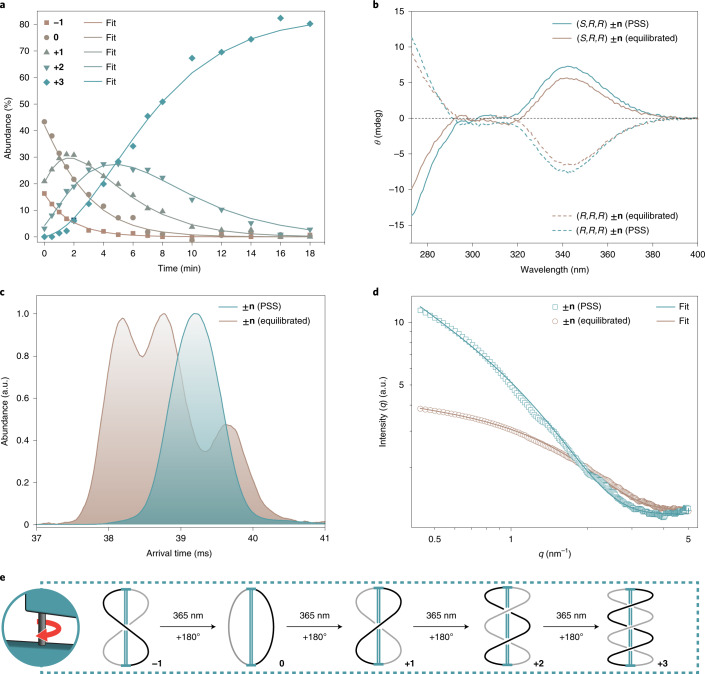
Sequence-specific, light-driven winding mechanism of AMM **±n**. **a**, Illumination (*λ*_irr_ = 365 nm, 18 min) of an equilibrium mixture of (*S,R,R*) motors **−1**, **0** and **+1** yields species **+3** via intermediate **+2**. Because of the unidirectional rotation of the motor, the process is topologically stereoconvergent and sequence specific. The representative kinetic profile was obtained by ^1^H-NMR spectroscopy (500 MHz, 10 °C, 1 mM in C_6_D_6_). **b**, CD spectra (0 °C, 10 μM in THF/C_6_D_6_) of an equilibrated (brown) and illuminated sample (blue) at PSS of both contrarotating motor diastereomers (*S,R,R*) (solid lines) and (*R,R,R*) **±n** (dashed lines). No sign inversion indicates that the motor core retains its helical chirality. **c**, IM–MS measurements: ATDs of the equilibrated (brown) and illuminated (blue) **±n** sample showing a transition from three species **−1**, **0** and **+1** to one species **+3**. **d**, Scattering intensities of a **±n** sample obtained by SAXS measurements (23 °C, 1 mM in toluene-*d*_8_) at equilibrium (brown, globular particles) and at PSS (blue, rod-like particles). **e**, Proposed mechanism for the photochemical formation of **+3** in sequential and unidirectional half-turns, starting from **−1**.

**Fig. 3:**
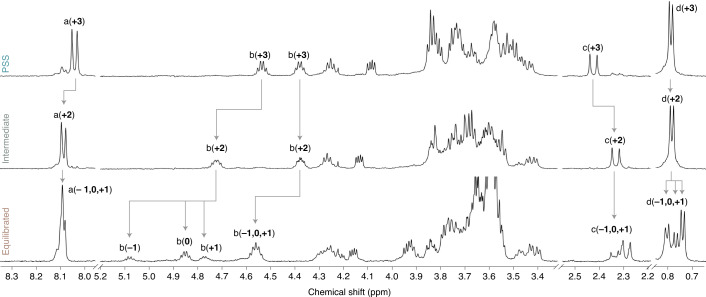
NMR spectra of AMM **±n** at different winding states. Partial ^1^H-NMR spectra (500 MHz, 10 °C, 1 mM in C_6_D_6_) of (*S,R,R*) **±n** at PSS (*λ*_irr_ = 365 nm, 18 min; top), after partial thermal relaxation (60 °C, 6 min; middle) and at equilibrium (bottom). The conversion of key signals is indicated with grey arrows (signal labels are shown in Fig. 1a; for more information see Supplementary Figs. 1–22 and Supplementary Tables 1 and 2).

To experimentally investigate the hypothesized operational routine of the nanoratchet, we initially studied the light-driven winding process of AMM **±n**. Figure 2a shows the sequential formation of distinct topological isomers as a function of time for (*S,R,R*) **±n** (Supplementary Fig. 35 and Supplementary Data Set 1). With the help of ^1^H-NMR spectroscopy (Fig. 3), we could follow the compositional change of a fully equilibrated sample upon illumination with UV light (irradiation wavelength *λ*_irr_ = 365 nm). In thermal equilibrium, AMM ±**n** can adopt up to one crossing and therefore exists as a mixture of distinct topological isomers **−1**, **0** and **+1** (the ratio is ~1:2:1). The obtained kinetic profile revealed a sequence-specific and stereoconvergent reaction mechanism (Supplementary Fig. 35), only possible due to the unidirectionality of the rotary molecular motor (Supplementary Fig. 37), by which the crossings in the bicyclic molecule are increased stepwise by one, until the system selectively reaches its final state **+3** via intermediate **+2** (Figs. 2a and 2e). The quantum yield (*Φ*) decreases with more crossings (see Supplementary Figs. 35 and 41 and later Fig. 5a). Repeating the illumination experiment with a non-macrocyclized motor showed no winding (Supplementary Figs. 21 and 22). Interestingly, the helical chirality of the motor moiety of both diastereomers (*S,R,R*) and (*R,R,R*) **±n** at the photostationary state (PSS) is retained during the winding process as evidenced by a relative increase in the intensity of their respective CD spectra (Fig. 2b and Supplementary Figs. 23–25 and 38). The formation of topological isomer **+3** from an equilibrium mixture of **−1**, **0** and **+1** was also observed using ion mobility interfaced with mass spectrometry (IM–MS) (Fig. 2c and Supplementary Fig. 36). The arrival time distribution (ATD) of the fully equilibrated sample shows three contributions that correspond to collision cross-sections (^DT^CCS_He_) of 351 Å², 342 Å² and 338 Å². These were respectively assigned to **0**, **−1** and **+1** by matching the experimental with theoretical collision cross-sections calculated for in silico candidate structures. After illumination with UV light, the ATD becomes narrower and shows a single contribution corresponding to ^DT^CCS_He_ = 344 Å², which was analogously attributed to **+3**. Additional support for the change in shape of the machine came from SAXS measurements (Fig. 2d; Supplementary Figs. 39 and 40 and Supplementary Table 3). At thermal equilibrium, AMM **±n** adopts a globular shape with a gyration radius of *r* = 0.9 nm. After illumination with UV light, an increase in scattering intensity was observed^27^, which can be attributed to the formation of elongated, rod-like and more compact objects with a cross-sectional radius of *r* = 0.7 nm. The combined data support a winding mechanism that is caused by the unidirectional rotation of the motor.

**Fig. 4:**
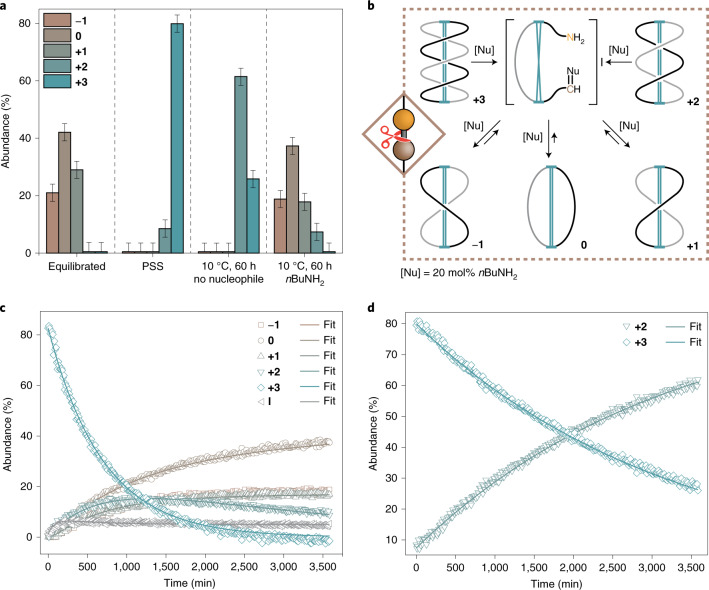
Unwinding mechanism and thermal stability of AMM **±n**. **a**, Chemical composition of an equilibrated **±n** sample (left), a sample at PSS (middle left), without addition of external nucleophile (middle right) and after relaxation at 10 °C for 60 h with 20 mol% of *n*BuNH_2_ (right). Full relaxation at 10 °C only occurs in the presence of nucleophiles. **b**, Proposed mechanism for the thermal relaxation of wound up **±n** via temporary ring-opening in the presence of catalytic amounts of nucleophile. **c**,**d**, Representative examples for the relaxation kinetics of an irradiated **±n** sample at 10 °C in the presence of 20 mol% of *n*BuNH_2_ (**c**) and without nucleophiles (**d**). The chemical composition (**a**) and kinetic profiles (**c**,**d**) were obtained by ^1^H-NMR spectroscopy (500 MHz, 10 °C, 1 mM in C_6_D_6_).

**Fig. 5:**
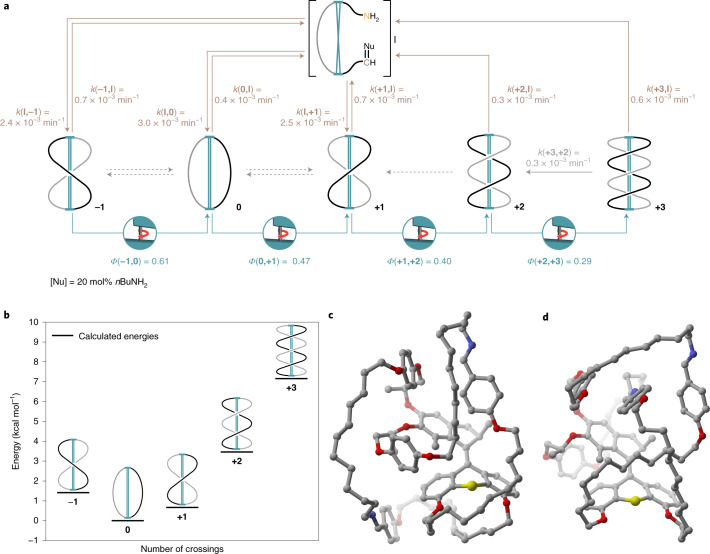
Energy, structure and detailed working mechanism of AMM **±n**. **a**, The full reaction scheme including all experimental rate constants/quantum yields (given as an average over at least two independent experiments) for the molecular machine operation at 10 °C shows the light-driven winding (blue; relative standard deviation ~10%), nucleophile-catalysed unwinding (brown; relative standard deviation ~35%) and sequence-specific unwinding (grey; relative standard deviation ~15%, solid arrow represents experimentally observed relaxation from **+3** to **+2**, while dotted arrows indicate that further, sequence-specific relaxation at 10 °C is not viable). An increasing number of crossings decreases the winding and increases the unwinding process. Note that the rates for the nucleophile-catalysed unwinding are all in a narrow range irrespective of the winding-induced strain. This can be rationalized when considering that the nucleophilic attack at the imine carbon atom is likely the rate-determining step in this reaction, while the subsequent ring-opening and unwinding steps are fast and thus do not affect the kinetics of the amine–imine exchange. **b**, Calculated energies of the most stable conformer of motors **−1** to **+3** in the gas phase relative to **0**. A higher number of crossings increases the electronic energy of the system. Experimentally, the system was found to reach up to **+3** crossings. **c**,**d**, Representative structures of one possible conformer of species **0** (**c**) and **+3** (**d**). The energies and structures in Fig. 5b–d were calculated at the M06-2X/def-SVP//GFN-xTB level of theory.

Next, we examined the unwinding of the system, both under thermal and nucleophile-assisted conditions (Fig. 4a and Supplementary Data Set 1). Full relaxation of the system at 10–40 °C can only be achieved in the presence of an external nucleophile, such as catalytic amounts of *n*-butyl amine (*n*BuNH_2_, Figs. 4a and 4c), under purely thermodynamic control as determined by ^1^H-NMR spectroscopy (Supplementary Fig. 27, for relaxation with water see Supplementary Figs. 33 and 34). The competing nucleophile can induce temporary ring-opening of the imine macrocycles by amine–imine exchange (forming open *n*-butyl imine **I** in 5%, Supplementary Fig. 27), leading to a topological reshuffling of the wound-up machine in a non-sequence-specific manner (Figs. 4b and 5a; Supplementary Figs. 27, 33 and 34), thus re-establishing the initial topological equilibrium. Interestingly, an increase in the number of crossings (and thus chemical strain) does not affect the reactivity of the imine groups towards *n*BuNH_2_ and therefore the rate for amine–imine exchange, as indicated by the rate constants (*k*) for nucleophile-induced ring-opening of the macrocycles (Fig. 5a). A similar behaviour was already observed for thiol–disulfide exchange^32^ and hydrolysis of esters^33^. However, the fact that ring-opening of **+3** and **+2** with *n*BuNH_2_ is an irreversible process (there is no measurable rate for the ring-closure, Fig. 5a) indicates that both topological isomers are high energy, strained species that are not populated in thermal equilibrium.

To gain further insight into the pure thermal stability of the ‘spring-loaded’ AMM, we studied the relaxation of a fully wound motor sample at different temperatures in the absence of an external nucleophile (Supplementary Figs. 26 and 28–32; trace amounts of nucleophiles, such as water, cannot be fully excluded). Under these conditions, the unwinding pathway via ring-opening is not feasible. The kinetic data associated with the various steps at 10 °C, both in the absence (grey) and presence (brown) of nucleophiles, are compiled in Fig. 5a (see also Supplementary Figs. 26–35). We found that bicyclic molecule **+3**, which is the most strained form, slowly decays at 10 °C (Fig. 5a, solid grey arrow; *k*(**+3**,**+2**) = 0.3 × 10^**−**3^ min^**−**1^) to selectively form less strained species **+2** after 60 h in ~60% yield (Figs. 4a and 4d, Supplementary Fig. 26). At this temperature, formation of species **−1**, **0**, or **+1** was not observed. Increasing the temperature stepwise from 10–60 °C led to an increase in the decay rate of **+3** by a factor of ~4 per 10 °C steps (Supplementary Fig. 32).

At 40 °C, complete unwinding was not observed even after one week, while full equilibration could be achieved within 42 h at 60 °C (Supplementary Figs. 29 and 30). The kinetic profiles of the relaxation processes clearly indicate a sequence-specific mechanism that can only be caused by an intramolecular reaction (there is no direct equilibrium between **−1** and **+1** under these conditions, Supplementary Figs. 28 and 29). Most importantly, an increase in the number of crossings increases the rate of unwinding and renders the relaxations from **+3** ⟶ **+2** and **+2** ⟶ **+1** irreversible, suggesting that potential energy is built up during the winding process (Figs. 5a and 5b). The potential energy accumulated during the illumination process can be released in a stepwise and sequence-specific manner when enough thermal energy is provided (Supplementary Figs. 26 and 28–30).

Together with an extensive computational analysis (Supplementary Data Set 2), the experimental and calculated data give a detailed picture of the machine’s working mechanism on the molecular scale (Fig. 5a–d and Supplementary Figs. 26–35, 37 and 41). The system at thermodynamic equilibrium consists of bridged bismacrocycles **−1**, **0** and **+1**, with an experimentally determined energy difference between molecule **0** (Fig. 5c) and singly twisted motors **+1** and **−1** of Δ*G*_exp_(**0**,**+1**) = 0.24 kcal mol^−1^ and Δ*G*_exp_(**0**,**−1**) = 0.39 kcal mol^−1^, respectively. Experimentally the system reaches up to three crossings, that is, **+3** (Fig. 5b). Species **+2** and **+3** (Fig. 5d) were not detected in the relaxed reaction mixture (Supplementary Fig. 2), as expected from the computational studies that predicted a steep increase in the potential energy per half-turn (Fig. 5b). Due to the irreversible nature of the transformation from **+3** and **+2** and from **+2** and **+1** the experimental energy difference between these species can only be estimated to be Δ*G*_exp_(**+2**,**+3**) ≥ 2 kcal mol^−1^ and Δ*G*_exp_(**+1,+2**) ≥ 2 kcal mol^−1^, respectively (under the conservative assumption that the absolute measurement error for NMR spectroscopy is ±3%). The experimentally observed quantum yields for the light-driven winding process diminish stepwise, going from *Φ*(**−1**,**0**) = 0.61 to *Φ*(**+2**,**+3**) = 0.29, meaning that the winding rate decreases with increasing strain (Fig. 5a). After illumination, the sequence-specific, intramolecular unwinding irreversibly forms species **+2** from **+3** (*k*(**+3**,**+2**) = 0.3 × 10^**−**3^ min^**−**1^) at 10 °C. Full relaxation at this temperature can only be achieved in the presence of nucleophiles via dynamic and non-sequence-specific ring-opening of the imine macrocycles via intermediate **I** (Fig. 5a top). Under these conditions, species **−1**, **0** and **+1** form with a comparable rate constant of *k* = 2.4–3.0 × 10^**−**3^ min^**−**1^ (for the kinetics of the non-catalysed, intramolecular unwinding see Supplementary Figs. 26 and 28–30), while ring-closure to **+2** and **+3** was not observed. A significant dependence of the ring-opening rate on the number of crossings in the system could not be detected.

This work demonstrates how the light-driven unidirectional rotary motion in AMM **±n** can decrease the overall kinetic (Fig. 5a) and thermodynamic (Fig. 5b) stability of the system during the winding process of the macrocycles. The experimental data clearly show that the small-molecule machine **±n** functions as a nanoratchet able to drive a coupled chemical equilibrium energetically uphill by light. We anticipate that our study will serve as an inspiration and blueprint for the design of AMMs of ever-increasing complexity which, for instance, can store light in the form of chemical energy or perform sophisticated synthetic tasks on the molecular scale.

## Methods

### Preparation of ±n samples

A stock solution of bisaldehyde and bisazide functionalized motor **S21** (1.0 mg, 0.60 µmol, 1.0 equiv.) in C_6_D_6_ was lyophilized in a J. Young NMR tube. Then, PPh_3_ (0.60 mg, 2.4 mmol, 4.0 equiv.) was added and the tube was put under high vacuum for 16 h. After that, the solids were dissolved in dry (distilled from CaH_2_) and degassed (three freeze–pump–thaw cycles) C_6_D_6_ or toluene-*d*8 (0.6 ml) inside a glovebox and activated molecular sieves (3 Å) were added. The sealed NMR tube was taken out of the glovebox and heated to 60 °C for 7 d in an oil bath under exclusion of light. Subsequently, the molecular sieves were removed inside a glovebox and the sample was used for further experiments without purification. Since the system is dynamic, the formation of the bridged bis-macrocyclic **±n** is concentration dependent. Increasing the concentration leads to formation of an insoluble polymer. The conversion of **S21** to **±n** was followed using ^1^H-NMR spectroscopy by observing the decrease of the aldehyde signal (~9.7 ppm) and increase of the imine signals (~8.1 ppm). Typically, bis-macrocyclic **±n** forms in 90–95%.

### Illumination of ±n samples for NMR spectroscopy

Typically, a J. Young NMR tube containing a 1 mM solution of equilibrated (*S,R,R*) or (*R,R,R*) **±n** in 0.6 ml C_6_D_6_ was illuminated with a hand-held UV lamp (365 nm, 6 W) on a shaker plate for 18 min at 8 °C (in a walkable fridge) or room temperature. The distance between lamp and NMR tube was kept constant throughout the experiment. To prevent any significant relaxation, illuminated samples were cooled with a 10 °C acetone bath before the respective measurement.

### CD spectroscopic measurements

CD spectra were recorded on a Jasco J-815 CD spectrometer. In a cuvette, 30 µl of a solution of (*S,R,R*) or (*R,R,R*) **±n** (1 mM in C_6_D_6_) was diluted with degassed and dry tetrahydrofuran (THF) to a total volume of 3 ml (concentration *c* = 10 μM). Benzene absorbs in the deep UV region and forces a cut-off at 270 nm. Spectra of illuminated samples were either recorded after in situ illumination with a hand-held UV lamp at 365 nm for 5 min (the distance between the cuvette and the lamp was ~2 cm) or by diluting a pre-illuminated sample at PSS with THF. Partial relaxation of the irradiated sample was achieved by heating the sample to 60 °C for 10 min.

### NMR spectroscopic measurements and kinetic analysis

All experiments were conducted on a Varian AVIII 500 NMR spectrometer that was precooled or prewarmed to the proper temperature. (*S,R,R*) **±n** samples were typically equilibrated for 5 min inside the instrument until the lock signal reached a constant value. The experimental data were subsequently fitted using COPASI v.4.29 (ref. ^34^). In order to obtain a fit that could give a realistic approximation of the irreversible and reversible reactions involved in each experiment, we simulated a reaction compartment of 0.6 ml (to match the volume of the solution of a typical NMR experiment) with concentration of the species involved of 1 mM. In all cases, the time unit used was minutes. The default Levenberg–Marquardt algorithm with a tolerance of 10^−6^ implemented in COPASI was used. The initial guess for the kinetic parameter estimation was to consider all species to be in equilibrium with one another. After every fitting run, visual inspection of the error associated to each kinetic constant provided indication of the relevance of a certain reaction. Kinetic constants with absolute values lower than 10^−6^ min^−1^ were approximated to 0 and the respective reaction deleted in the next iteration.

### IM–MS measurements

Ion mobility measurements were performed using a custom drift-tube instrumentation hosted in the Fritz Haber Institute of the Max Planck Society (Berlin, Germany) and adapted from a previous design^35^. The instrument is designed around a nanoelectrospray ionization source interfaced with a succession of radially confining entrance funnel, drift tube and exit funnel. This ensemble is prolonged by a quadrupole mass analyser under high vacuum and ended by an electron multiplier detector (ETP Ion Detect) for ion counting. In practice, samples were diluted to 10 μM in acetonitrile and nanoelectrospray ionization was used to generate ions using a needle voltage of 0.57 kV and a backing pressure of 0.8 bar (N_2_)_._ The ~160-cm-long drift tube was filled with helium buffer gas at a pressure of 4 mbar and subjected to a 2 kV direct current electric field for mobility separation. Ions were filtered for *m/z* = 1,612 Da, which correspond to the singly protonated molecular ion [M + H]^+^.

### SAXS measurements

SAXS measurements were performed at the Multipurpose X-ray Instrument for Nanostructure Analysis instrument at the University of Groningen. The instrument is built on a Cu rotating anode high brilliance X-ray source, providing X-ray photons with a wavelength of *λ* = 0.154 nm. The SAXS patterns were recorded using a 2D Vantec500 detector placed 24 cm away from the sample. SAXS 1D profiles were obtained by radially averaging the scattered intensity around the origin of the image (defined by the direct beam position on the detector) using MATLAB. Standard corrections for the detector distortion and sensitivity were applied. The scattering from the buffer solution was subtracted to obtain the neat SAXS signal of the sample. The 1D SAXS profiles are plotted against the modulus of the scattering vector defined as *q* = 4π sin*θ*/*λ*, where *θ* is half of the scattering angle. The probed scattering angle range was calibrated using the known positions of the diffraction peaks from a standard Silver Behenate sample (NIST).

## Online content

Any methods, additional references, Nature Research reporting summaries, source data, extended data, supplementary information, acknowledgements, peer review information; details of author contributions and competing interests; and statements of data and code availability are available at 10.1038/s41565-021-01021-z.

## Supplementary information

Supplementary Information Supplementary figures.

Supplementary Data Supplementary Data Set 1. Output files for kinetic analysis.

Supplementary Data Set 2. Gaussian output and xyz files for computed structures.

## Data Availability

All data needed to evaluate the conclusions in the paper are present in the paper and/or the Supplementary Information. Additional data related to this paper may be requested from the authors upon reasonable request.

## References

[1] Goodsell, D. S. *The Machinery of Life* (Springer, 2009).

[2] Schliwa, M. *Molecular Motors* (Wiley-VCH, 2006).

[3] Balzani V, Credi A, Raymo FM, Stoddart JF (2000). Artificial molecular machines. Angew. Chem. Int. Ed..

[4] Sauvage, J.-P. *Molecular Machines and Motors* (Springer, 2001).

[5] Balzani, V., Credi, A. & Venturi, M. *Molecular Devices and Machines: Concepts and Perspectives for the Nanoworld* (Wiley-VCH, 2008).

[6] Rowan SJ, Cantrill SJ, Cousins GRL, Sanders JKM, Stoddart JF (2002). Dynamic covalent chemistry. Angew. Chem. Int. Ed..

[7] Corbett PT (2006). Dynamic combinatorial chemistry. Chem. Rev..

[8] Lehn JM (2007). From supramolecular chemistry towards constitutional dynamic chemistry and adaptive chemistry. Chem. Soc. Rev..

[9] Belowich ME, Stoddart JF (2012). Dynamic imine chemistry. Chem. Soc. Rev..

[10] Koumura N, Geertsema EM, Van Gelder MB, Meetsma A, Feringa BL (2002). Second generation light-driven molecular motors. Unidirectional rotation controlled by a single stereogenic center with near-perfect photoequilibria and acceleration of the speed of rotation by structural modification. J. Am. Chem. Soc..

[11] Roke D, Wezenberg SJ, Feringa BL (2018). Molecular rotary motors: unidirectional motion around double bonds. Proc. Natl Acad. Sci. USA.

[12] Coskun A, Banaszak M, Astumian RD, Stoddart JF, Grzybowski BA (2012). Great expectations: can artificial molecular machines deliver on their promise?. Chem. Soc. Rev..

[13] Browne WR, Feringa BL (2006). Making molecular machines work. Nat. Nanotechnol..

[14] Baroncini M, Silvi S, Credi A (2020). Photo- and redox-driven artificial molecular motors. Chem. Rev..

[15] Bruns, C. J. & Stoddart, J. F. *The Nature of the Mechanical Bond* (John Wiley & Sons, 2016).

[16] Greb L, Lehn JM (2014). Light-driven molecular motors as four-step or two-step unidirectional rotors. J. Am. Chem. Soc..

[17] Kassem S (2017). Stereodivergent synthesis with a programmable molecular machine. Nature.

[18] García-López V, Liu D, Tour JM (2020). Light-activated organic molecular motors and their applications. Chem. Rev..

[19] Uhl E, Thumser S, Mayer P, Dube H (2018). Transmission of unidirectional molecular motor rotation to a remote biaryl axis. Angew. Chem. Int. Ed..

[20] Štacko P (2017). Locked synchronous rotor motion in a molecular motor. Science.

[21] Lewandowski B (2013). Sequence-specific peptide synthesis by an artificial small-molecule machine. Science.

[22] Cheng C (2015). An artificial molecular pump. Nat. Nanotechnol..

[23] Ragazzon G, Baroncini M, Silvi S, Venturi M, Credi A (2015). Light-powered autonomous and directional molecular motion of a dissipative self-assembling system. Nat. Nanotechnol..

[24] Ragazzon G, Baroncini M, Silvi S, Venturi M, Credi A (2015). Light-powered, artificial molecular pumps: a minimalistic approach. Beilstein J. Nanotechnol..

[25] Qiu Y (2019). A molecular dual pump. J. Am. Chem. Soc..

[26] Eelkema R (2006). Molecular machines: nanomotor rotates microscale objects. Nature.

[27] Li Q (2015). Macroscopic contraction of a gel induced by the integrated motion of light-driven molecular motors. Nat. Nanotechnol..

[28] Kathan M, Hecht S (2017). Photoswitchable molecules as key ingredients to drive systems away from the global thermodynamic minimum. Chem. Soc. Rev..

[29] Sell H (2019). Towards a light driven molecular assembler. Commun. Chem..

[30] Aprahamian I (2020). The future of molecular machines. ACS Cent. Sci..

[31] Fielden SDP, Leigh DA, Woltering SL (2017). Molecular knots. Angew. Chem. Int. Ed..

[32] Kucharski TJ (2009). Kinetics of thiol/disulfide exchange correlate weakly with the restoring force in the disulfide moiety. Angew. Chem. Int. Ed..

[33] Akbulatov S, Tian Y, Kapustin E, Boulatov R (2013). Model studies of the kinetics of ester hydrolysis under stretching force. Angew. Chem. Int. Ed..

[34] Hoops S (2006). COPASI–a COmplex PAthway SImulator. Bioinformatics.

[35] Warnke S (2015). Protomers of benzocaine: solvent and permittivity dependence. J. Am. Chem. Soc..

