# Author Correction: A light-fuelled nanoratchet shifts a coupled chemical equilibrium

**DOI:** 10.1038/s41565-022-01119-y

**Published:** 2022-03-24

**Authors:** Michael Kathan, Stefano Crespi, Niklas O. Thiel, Daniel L. Stares, Denis Morsa, John de Boer, Gianni Pacella, Tobias van den Enk, Piermichele Kobauri, Giuseppe Portale, Christoph A. Schalley, Ben L. Feringa

**Affiliations:** 1 Stratingh Institute for Chemistry, Center for Systems Chemistry and Zernike Institute for Advanced Materials, Faculty of Mathematics and Natural Sciences, University of Groningen, Groningen, the Netherlands; 2 Institut für Chemie und Biochemie der Freien Universität Berlin, Berlin, Germany; 3 Fritz-Haber-Institut der Max-Planck-Gesellschaft, Berlin, Germany; 4 Macromolecular Chemistry and New Polymeric Materials and Zernike Institute for Advanced Materials, Faculty of Mathematics and Natural Sciences, University of Groningen, Groningen, the Netherlands

**Keywords:** Chemistry, Photochemistry

Correction to: *Nature Nanotechnology* 10.1038/s41565-021-01021-z, published online 16 December 2021.

This Letter was originally published with an incorrect copyright status, and should have been Open Access; this has been amended and the Letter is now published under a Creative Commons license CC BY 4.0.

