# Supplementary material for: A light-fuelled nanoratchet shifts a coupled chemical equilibrium
Source: Nat Nanotechnol. 2021 Dec 16;17(2):159–65. doi: 10.1038/s41565-021-01021-z (PMC8956507; doi:10.1038/s41565-021-01021-z)

nature portfolio

https://doi.org/10.1038/s41565-021-01021-z

Supplementary information

A light-fuelled nanoratchet shifts a coupled chemical equilibrium

In the format provided by the
authors and unedited

Supplementary Information

A light-fueled nanoratchet shifts a coupled chemical equilibrium

Michael Kathan[1], Stefano Crespi[1], Niklas O. Thiel[1], Daniel L. Stares[2], Denis Morsa[3], John de Boer[1], Gianni Pacella[4], Tobias van den Enk[1], Piermichele Kobauri[1], Giuseppe Portale[4], Christoph A. Schalley[2]*, Ben L. Feringa[1]**

[1]Stratingh Institute for Chemistry, Center for Systems Chemistry and Zernike Institute for Advanced Materials, Faculty of Mathematics and Natural Sciences, University of Groningen, Nijenborgh 4, Groningen 9747 AG, The Netherlands
[2]Institut für Chemie und Biochemie der Freien Universität Berlin, Arnimallee 20, 14195 Berlin, Germany
[3]Fritz-Haber-Institut der Max-Planck-Gesellschaft, Faradayweg 4–6, 14195 Berlin, Germany
[4]Macromolecular Chemistry and New Polymeric Materials and Zernike Institute for Advanced Materials, Faculty of Mathematics and Natural Sciences, University of Groningen, Nijenborgh 4, Groningen 9747 AG, The Netherlands

*Corresponding authors. E-mail: b.l.feringa@rug.nl, m.p.k.kathan@rug.nl, c.schalley@fu-berlin.de

General Information

Chemicals were purchased from commercial sources and used without purification. If not stated otherwise, all reactions were carried out in flame-dried glassware under a nitrogen atmosphere using standard Schlenk techniques. Solutions and reagents were added with nitrogen-flushed disposable syringes/needles. For NMR experiments, solvents were added using glass syringes and stainless steel needles (stored at 80 °C). Analytical thin layer chromatography (TLC) was performed on silica gel 60 G/UV_{254} aluminium sheets from Merck (0.25 mm). Flash column chromatography was performed on silica gel Davisil LC60A (Merck type 9385, 230–400 mesh) or Reveleris X2 Flash Chromatography system (MPLC) using the indicated solvents. NMR spectra were recorded on a Varian Mercury Plus (^{1}H: 400 MHz, ^{13}C: 100 MHz), a Varian Unity Plus (^{1}H: 500 MHz, ^{13}C: 125 MHz) or a Bruker Innova (^{1}H: 600 MHz, ^{13}C: 151 MHz). Chemical shifts are in parts per million (ppm) relative to TMS. For the calibration of the chemical shift, the residual solvent resonance was used as the internal standard. Data are as follows: chemical shift (δ in ppm), multiplicity (br = broad, s = singlet, d = doublet, t = triplet, p = pentet, m = multiplet), coupling constants (*J* in Hz), integration. High resolution mass spectra (HRMS) were recorded on an LTQ Orbitrap XL. Ion mobility (IM) measurements were performed using a custom drift-tube instrumentation hosted in the Fritz Haber Institute of the Max Planck Society (Berlin, Germany). CD spectra were measured on a Jasco J-815 CD spectrometer. SAXS measurements were performed at the Multipurpose X-ray Instrument for Nanostructure Analysis (MINA) instrument at the University of Groningen. Illuminations were carried out using a UV lamp from Vilber Lourmat (6W, λ_{irr} = 365 nm).

The following compounds were prepared according to literature procedures: 1,12-dodecanediol mono(4-methylbenzenesulfonate) **S1**[1], 12-azidododecyl 4-methylbenzenesulfonate **S4**[2], 5,6-dihydroxy-2,7-dimethyl-indane-1-one **S6**[3] and *N*,*N*-diethyl-3-methoxy-2-((2-methoxyphenyl)thio)benzamide **S9**[4].

The following compounds were prepared according to modified literature procedures: **S2**[5], **S7**–**S8**[3], **S10**–**S16**[3,4].

Spectra of all compounds described in the synthesis section can be found in the spectra appendix at the end of the Supplementary Information.

The absolute measurement error for NMR spectroscopic measurements involving molecular machine **±n** was estimated to be ±3%. The average relative standard deviation of the fit over all individual experiments amounts to < 0.7%. This confirms the proposed kinetic models and the related mechanisms for each individual experiment. The values given in the main manuscript are an average over at least two individual experiments. The relative standard deviation between these individual experiments was estimated to be ±10% for quantum yields; ±15% for intramolecular rate constants; ±15% for experimental Gibbs free energies at 25 °C; ±35% for intermolecular rate constants. For further information see also Supplementary Data Set 1.

Synthesis

Synthesis of side-arms

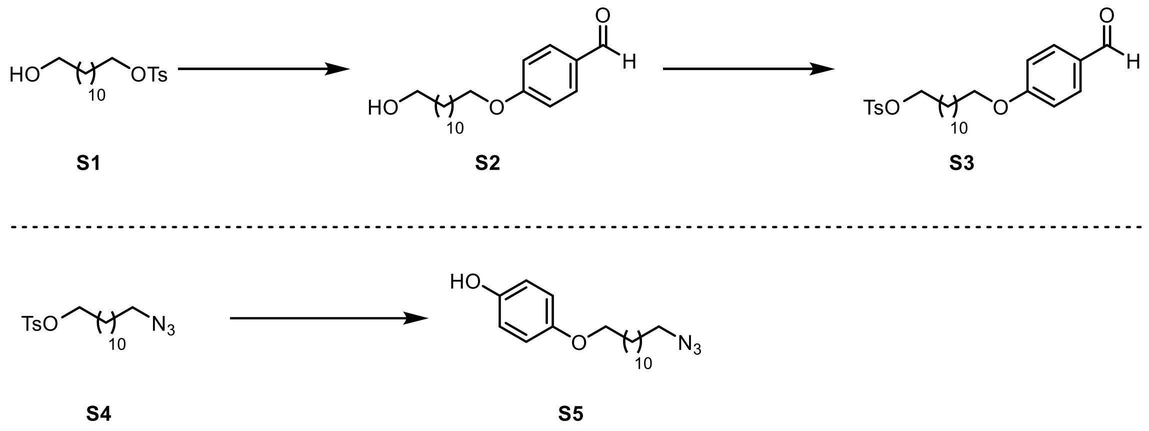

Scheme 1 | Synthesis of side-arms. Synthesis of aldehyde linker **S3** (top) and azide linker **S5** (bottom).

4-((12-hydroxydodecyl)oxy)benzaldehyde (S2)[5]

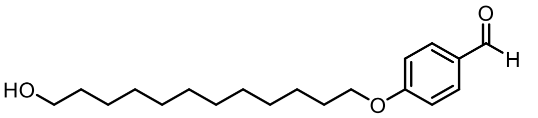

S2
$C_{19}H_{30}O_3$
Mw = 306.45 g/mol

In a flame-dried 3-necked flask equipped with a stirring bar and a reflux condenser, K_2CO_3 (1.74 g, 12.6 mmol, 3.00 eq.) and 4-hydroxybenzaldehyde (1.03 g, 8.42 mmol, 2.00 eq.) were suspended in dry MeCN (21 mL). Alkyl tosylate **S1** (1.50 g, 4.21 mmol, 1.00 eq.) was added and the mixture was heated to 90 °C for 6 h. Subsequently, the mixture was cooled to room temperature and all volatiles were removed under reduced pressure. To the residue, H_2O (20 mL) and ethyl acetate (20 mL) were added. The phases were separated and the aqueous layer was extracted with ethyl acetate (3x10 mL) and the combined organic layers were dried over $MgSO_4$. All volatiles were removed under reduced pressure. The title compound **S2** was obtained as an off-white solid (1.20 g, 3.92 mmol, 93%) and was used without further purification.

^{1}H-NMR (400 MHz, $CDCl_3$): δ = 1.21–1.38 (m, 14H), 1.46 (p, *J* = 7.0, 6.3 Hz, 2H), 1.51–1.62 (m, 2H), 1.74–1.86 (m, 2H), 3.63 (t, *J* = 6.6 Hz, 2H), 4.03 (t, *J* = 6.5 Hz, 2H), 6.95–7.02 (m, 2H), 7.78–7.86 (m, 2H), 9.87 (s, 1H) ppm. The signal for *OH* could not be detected.

^{13}C-NMR (101 MHz, $CDCl_3$): δ = 25.9, 26.1, 29.2, 29.5, 29.6, 29.7, 29.7, 29.7, 32.9, 63.2, 68.3, 68.6, 114.9, 129.9, 132.1, 164.4, 191.0 ppm.

HRMS-ESI (ESI+): *m/z* calculated for $C_{19}H_{31}O_3^+$ ($[M+H]^+$): 307.2268, found 307.2270.

The analytical data is in accordance with the literature.

12-(4-formylphenoxy)dodecyl 4-methylbenzenesulfonate (S3)

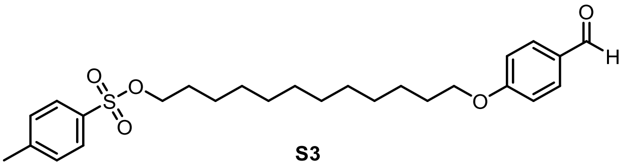

S3
$C_{26}H_{36}O_5S$
Mw = 460.63 g/mol

In a flame-dried Schlenk-tube, 4-toluenesulfonyl chloride (560 mg, 2.94 mmol, 1.50 eq.), Et_3N (0.55 mL, 3.9 mmol, 2.0 eq.) and alcohol **S2** (600 mg, 1.96 mmol, 1.00 eq.) were dissolved in CH_2Cl_2 (9.8 mL). 4-(Dimethylamino)pyridine (24 mg, 0.20 mmol, 0.10 eq.) was added as a solid and the mixture was stirred for 14 h at room temperature. Subsequently, H_2O (10 mL) was added and the phases were separated. The aqueous layer was extracted with CH_2Cl_2 (2x8 mL) and the combined organic layers were dried over $MgSO_4$. All volatiles were removed under reduced pressure. The crude product was purified by MPLC (SiO_2; *n*-pentane/CH_2Cl_2 gradient 60:40 ⟶ 0:100). The title compound **S3** was obtained as a colorless solid (0.74 g, 1.6 mmol, 82%).

^{1}H-NMR (400 MHz, $CDCl_3$): δ = 1.17–1.40 (m, 14H), 1.40–1.51 (m, 2H), 1.57–1.68 (m, 2H), 1.80 (p, *J* = 6.7 Hz, 2H), 2.44 (s, 3H), 4.02 (dt, *J* = 7.9, 6.5 Hz, 4H), 6.94–7.02 (m, 2H), 7.33 (d, *J* = 8.0 Hz, 2H), 7.75–7.86 (m, 4H), 9.87 (s, 1H) ppm.

^{13}C-NMR (101 MHz, $CDCl_3$): δ = 21.8, 25.5, 26.1, 29.0, 29.0, 29.2, 29.4, 29.5, 29.6, 29.6, 29.6, 68.5, 70.8, 114.9, 128.0, 129.9, 129.9, 132.1, 133.4, 144.7, 164.4, 190.9 ppm.

HRMS-ESI (ESI+): *m/z* calculated for $C_{26}H_{37}O_5S^+$ ($[M+H]^+$): 461.2356, found 461.2359.

4-((12-azidododecyl)oxy)phenol (S5)

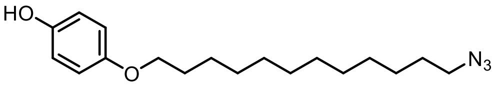

S5
$C_{18}H_{29}N_3O_2$
Mw = 319.45 g/mol

In a flame-dried 3-necked flask equipped with a stirring bar and a reflux condenser, K_2CO_3 (804 mg, 5.82 mmol, 3.00 eq.) and hydroquinone (2.14 g, 19.4 mmol, 10.0 eq.) were suspended in dry MeCN (9.7 mL). **S4** (740 mg, 1.94 mmol, 1.00 eq.) was added and the mixture was heated to 90 °C for 16 h. Subsequently, the mixture was cooled to room temperature and all volatiles were removed under reduced pressure. Then, H_2O (15 mL) and ethyl acetate (15 mL) were added to the residue. The phases were separated and the aqueous layer was extracted with ethyl acetate (3x8 mL). The combined organic layers were dried over $MgSO_4$ and all volatiles were removed under reduced pressure. The crude product was purified by MPLC (SiO_2; *n*-pentane/CH_2Cl_2 gradient 70:30 ⟶ 0:100). The title compound **S5** was obtained as an off-white solid (380 mg, 1.19 mmol, 61%).

^{1}H-NMR (400 MHz, $CDCl_3$): δ = 1.21–1.40 (m, 14H), 1.40–1.47 (m, 2H), 1.60 (p, *J* = 7.0 Hz, 2H), 1.69–1.81 (m, 2H), 3.26 (t, *J* = 7.0 Hz, 2H), 3.89 (t, *J* = 6.6 Hz, 2H), 4.36 (br s, 1H), 6.70–6.82 (m, 4H) ppm.

^{13}C-NMR (101 MHz, $CDCl_3$): δ = 26.2, 26.8, 29.0, 29.3, 29.5, 29.5, 29.6, 29.6, 29.6, 29.7, 51.6, 68.9, 115.8, 116.1, 149.5, 153.4 ppm.

HRMS-ESI (ESI^-): *m/z* for $C_{18}H_{28}N_3O_2^-$ [$(M-H)^-$]: calc. 318.2187, found 318.2187.

Synthesis of the motor core

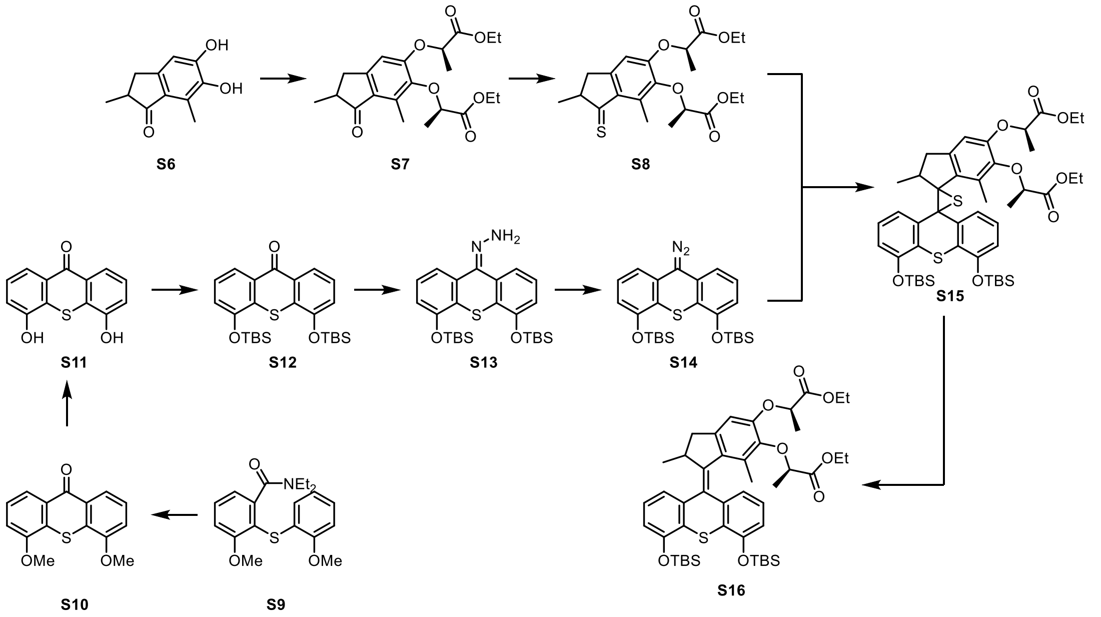

Scheme 2 | Synthesis of the motor core.

Diethyl 2,2'-((2,7-dimethyl-1-oxo-2,3-dihydro-1*H*-indene-5,6-diyl)bis(oxy))(2*R*,2'*R*)-dipropionate (S7)[3]

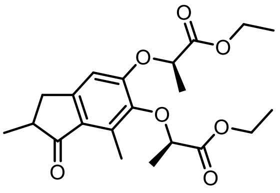

S7
$C_{21}H_{28}O_7$
Mw = 392.45 g/mol

Under a nitrogen atmosphere, ketone **S6** (9.00 g, 0.05 mol, 1.00 eq.), PPh_3 (65.9 g, 0.25 mol, 5 eq.), and (*S*)-lactic ester (28.7 mL, 0.25 mol, 5.00 eq.) were dissolved in dry THF (180 mL) and DIAD (50.8 g, 0.25 mol, 5.00 eq.) in dry THF (180 mL) was slowly added at 0 °C. The reaction mixture was stirred at room temperature for 16 h. Subsequently, all volatiles were removed under reduced pressure and 400 mL *n*-pentane/Et_2O (7:3) were added. Then, the mixture was sonicated and the precipitate removed by filtration. The filtrate was purified by MPLC (SiO_2; *n*-pentane/ethyl acetate gradient 95:05 ⟶ 85:15) and the title compound **S7** was obtained as a colorless oil (6.00 g, 16.0 mmol, 32%).

^{1}H-NMR (300 MHz, $CDCl_3$): δ = 1.18–1.28 (m, 9H), 1.55–1.71 (m, 6H), 2.45–2.70 (m, 5H), 3.18 (ddd, *J* = 16.7, 7.6, 2.8 Hz, 1H), 4.06–4.29 (m, 4H), 4.77–4.91 (m, 2H), 6.54 (s, 1H) ppm.

The analytical data is in accordance with the literature.

(4,5-bis((*tert*-butyldimethylsilyl)oxy)-9*H*-thioxanthen-9-ylidene)hydrazine (S8)[3]

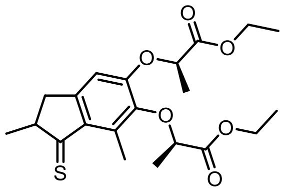

S8
$C_{21}H_{28}O_6S$
Mw = 408.51 g/mol

Under a nitrogen atmosphere, indanone **S7** (350 mg, 0.892 mmol, 1.0 eq.) was dissolved in dry toluene (13 mL) and P_4S_{10} (3.96 g, 17.8 mmol, 10.0 eq.) was added at 25 °C. After stirring at 80 °C for 2 h, the reaction mixture was quickly purified by flash column chromatography (SiO_2; first *n*-pentane, then ethyl acetate) under inert atmosphere. TLC (SiO_2; *n*-pentane/ethyl acetate 8:2) indicates full conversion of the starting material. Removal of the solvent yields product **S8** as a purple oil, which was directly used in the next reaction without further purification to avoid decomposition.

4,5-dimethoxy-9*H*-thioxanthen-9-one (S10)[3]

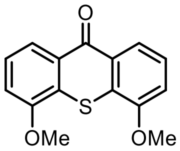

S10
$C_{15}H_{12}O_3S$
Mw = 272.32 g/mol

In a flame-dried 3-necked flask, a freshly made LDA solution (1.0 L, 0.50 M, 5.0 eq.) in dry THF was prepared at –78 °C and stirred at room temperature for 30 min. After cooling to 0 °C with an ice bath, a solution of amide **S9** (33.7 g, 98.0 mmol, 1.00 eq.) in dry THF (450 mL) was added dropwise to the LDA solution. After completion of the addition, the mixture was stirred at room temperature for 1 h. After full conversion was observed by TLC (*n*-pentane:ethyl acetate = 7:3), aq. NH_4Cl-solution (1M, 500 mL) was slowly added. The phases were separated and the aqueous layer was extracted with CH_2Cl_2 (2x500 mL). The combined organic layers were dried over $MgSO_4$ and all volatiles were removed under reduced pressure. The crude product was purified first by trituration in *n*-pentane/ethyl acetate and then recrystallization from ethyl acetate to afford title compound **S10** as a yellow powder (16.9 g, 62.0 mmol, 63%).

^{1}H-NMR (400 MHz, DMSO-d_6): δ = 4.03 (s, 6H), 7.44 (dd, *J* = 8.0, 1.2 Hz, 2H), 7.55 (t, *J* = 8.0 Hz, 2H), 8.06 (dd, *J* = 8.1, 1.2 Hz, 2H) ppm.

^{13}C-NMR (101 MHz, DMSO-d_6): δ = 56.7, 113.3, 120.5, 126.2, 126.7, 129.0, 154.5, 178.9 ppm.

HRMS-ESI (ESI+): *m/z* calculated for $C_{15}H_{13}O_3S$ ($[M+H]^+$): 273.0580, found 273.0584.

The analytical data is in accordance with the literature.

4,5-bis((*tert*-butyldimethylsilyl)oxy)-9*H*-thioxanthen-9-one (S12)[3]

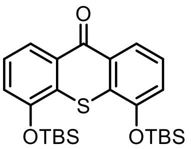

S12

$C_{25}H_{36}O_3SSi_2$

Mw = 472.79 g/mol

Cleavage of methyl ethers:
In a flame-dried 3-necked flask, thioxanthon **S10** (16.8 g, 62.0 mmol, 1.00 eq.) was dissolved in boiling CH_2Cl_2 (550 mL). The homogeneous solution was cooled to 0 °C with an ice bath and BBr_3 (29.2 mL, 308 mmol, 5.00 eq.) was added slowly. After the addition was complete, the mixture was stirred overnight and allowed to slowly warm to room temperature. Next, the mixture was cooled to 0 °C with an ice bath and MeOH (100 mL) was added slowly. After stirring for 3 h, all volatiles were removed under reduced pressure. The residue was suspended in MeOH (100 mL), which was evaporated again. Next, the crude solid was washed on a glass frit with H_2O (ca. 100 mL). Dissolving in acetone (400 mL) and drying over $MgSO_4$ delivered **S11** which was used without further purification.

TBS-protection:
In a flame-dried 3-necked-flask crude bisphenol **S11**, imidazole (12.6 g, 185 mmol, 3.00 eq.) and 4-(dimethylamino)pyridine (0.76 g, 6.2 mmol, 0.100 eq.), were dissolved in dry THF (250 mL). A solution of TBSCl (27.9 g, 185 mmol, 3.00 eq.) in dry THF (150 mL) was added and the mixture was stirred at room temperature for 16 h. Subsequently, the reaction mixture was filtered over a pad of silica gel and washed with CH_2Cl_2 (ca. 100 mL). The crude product was purified by recrystallization from EtOH and title compound **S12** was obtained as yellow needles (22 g, 47 mmol, 75% over two steps).

^{1}H-NMR (400 MHz, $CDCl_3$): δ = 0.35 (s, 12H), 1.11 (s, 18H), 7.10 (dd, *J* = 7.9, 1.3 Hz, 2H), 7.35 (t, *J* = 8.0 Hz, 2H), 8.26 (dd, *J* = 8.1, 1.3 Hz, 2H) ppm.

^{13}C-NMR (101 MHz, $CDCl_3$): δ = -4.0, 18.6, 26.0, 102.5, 119.8, 122.2, 125.8, 130.5, 130.7, 151.5, 180.8 ppm.

HRMS-ESI (ESI+): *m/z* calculated for $C_{25}H_{37}O_3SSi_2^+$ ($[M+H]^+$): 473.1996, found 473.1999.

The analytical data is in accordance with the literature.

(4,5-bis((*tert*-butyldimethylsilyl)oxy)-9*H*-thioxanthen-9-ylidene)hydrazine (S13)[3]

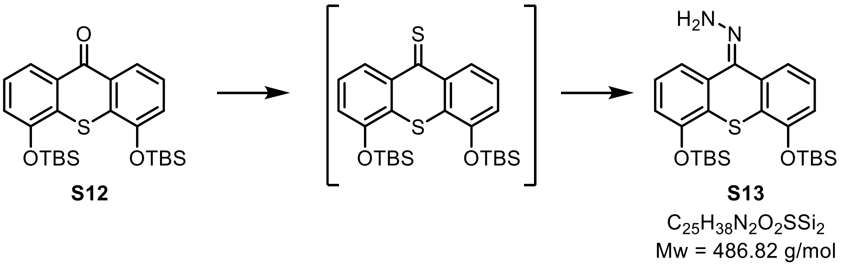

Thioketone formation:
Under a nitrogen atmosphere in a flame-dried 3-necked flask equipped with a stirring bar and a reflux condenser, thioxanthone **S12** (2.0 g, 4.0 mmol, 1.0 eq.) was dissolved in dry toluene (20 mL). Lawesson's reagent (5.1 g, 13 mmol, 3.0 eq.) was added and the mixture was heated to 80 °C for 2 h. Subsequently, the mixture was cooled to room temperature and the crude product was purified by flash column chromatography (*n*-pentane/ethyl acetate = 4:1) and the thioketone was immediately converted further.

Hydrazone formation:
In a round-bottom flask, the thioketone was dissolved in THF (15 mL) and stirred at room temperature. Next, hydrazine (2.1 mL, 4.2 mmol, 10 eq.) was added and the mixture was stirred at room temperature for 2 h. During this time, the color of the reaction mixture changed from green to almost colorless. Then, all volatiles were removed under reduced pressure and the crude product was purified by MPLC (SiO_2; *n*-pentane/ethyl acetate gradient 100:0 ⟶ 80:20). The title compound **S13** (2.0 g, 4.0 mmol, 99%) was obtained as a slightly yellow oil which solidified upon standing.

^{1}H-NMR (400 MHz, $CDCl_3$): δ = 0.28 (s, 6H), 0.31 (s, 6H), 1.07 (s, 10H), 1.09 (s, 9H), 5.83 (s, 2H), 6.78 (dd, *J* = 7.9, 1.1 Hz, 1H), 6.84 (dd, *J* = 8.0, 1.1 Hz, 1H), 7.16 (t, *J* = 7.9 Hz, 1H), 7.19 (d, *J* = 8.0 Hz, 1H), 7.43 (dd, *J* = 7.8, 1.2 Hz, 1H), 7.63 (dd, *J* = 7.9, 1.1 Hz, 1H) ppm.

The analytical data is in accordance with the literature.

(4,5-bis((tert-butyldimethylsilyl)oxy)-9*H*-thioxanthen-9-ylidene)hydrazine (S14)[3]

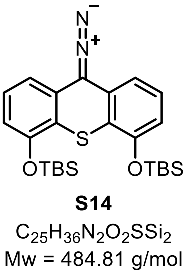

Under an argon atmosphere and exclusion of light, hydrazone **S13** (478 mg, 0.981 mmol, 1.0 eq.) was dissolved in dry THF (13 mL). Then, MnO_2 (1.55 g, 17.8 mmol, 18 eq.; manganese(IV) oxide activated, technical grade ≥ 90%, FLUKA Cat. 63548) and Na_2SO_4 (633 mg, 4.46 mmol, 4.5 eq.) were added at 0 °C. After stirring at 0 °C for 10 min, the reaction mixture was quickly filtered with a cannula (Whatman technique) under strict exclusion of light and air and the residue was washed two more times with dry and cold THF (2 mL, 0 °C). The green filtrate was kept at 0 °C and immediately used for the next reaction.

Diethyl 2,2'-((4'',5''-bis((*tert*-butyldimethylsilyl)oxy)-2,7-dimethyl-2,3-dihydrodispiro-[indene-1,2'-thiirane-3',9''-thioxanthene]-5,6-diyl)bis(oxy))(2*R*,2'*R*)-dipropionate (*S*15)[3]

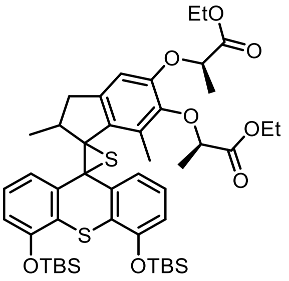

S15

$C_{47}H_{68}O_8S_2Si_2$

Mw = 881.34 g/mol

Under an argon atmosphere and exclusion of light, thioketone **S8** was dissolved in dry THF (2 mL), cooled to 0 °C, and slowly added to a solution of diazo **S14** in dry THF at 0 °C. After three vacuum-argon cycles, the reaction mixture was allowed to warm up to room temperature while stirring for 16 h. Purification by MPLC (SiO_2; *n*-pentane/ethyl acetate gradient 100:0 ⟶ 90:10) affords diasteromers (*R,R,R)*-**S15** (254 mg, 0.294 mmol, 33%; elutes at 6% ethyl acetate) and *(S,R,R)*-**S15** (312 mg, 0.361 mmol, 35%; elutes at 7% ethyl acetate) with excellent separation.

(*R,R,R*)-**S15**

^{1}H-NMR (300 MHz, $CDCl_3$): δ = –0.08 (s, 3H), 0.08 (s, 3H), 0.23 (s, 3H), 0.25 (s, 3H), 0.91 (d, *J* = 6.9 Hz, 3H), 0.96 (s, 9H), 1.04 (s, 9H), 1.07 (s, 1H), 1.22 (t, *J* = 7.1 Hz, 3H), 1.30 (t, *J* = 7.1 Hz, 3H), 1.42 (d, *J* = 6.8 Hz, 3H), 1.52 (d, *J* = 6.7 Hz, 3H), 2.02–2.10 (m, 1H), 2.14 (s, 3H), 3.18 (dd, *J* = 14.8, 6.4 Hz, 1H), 4.10–4.29 (m, 5H), 4.60 (q, *J* = 6.7 Hz, 1H), 6.34 (s, 1H), 6.60 (d, *J* = 8.0 Hz, 1H), 6.73 (d, *J* = 7.9 Hz, 1H), 7.01 (t, *J* = 7.9 Hz, 1H), 7.10 (t, *J* = 7.9 Hz, 1H), 7.24 (d, *J* = 7.9 Hz, 1H), 7.39 (d, *J* = 8.1 Hz, 1H) ppm.

(*S,R,R*)-**S15**

^{1}H-NMR (300 MHz, $CDCl_3$): δ = –0.12 (s, 3H), 0.06 (s, 3H), 0.23 (s, 3H), 0.25 (s, 3H), 0.94 (s, 9H), 1.05 (s, 9H), 1.14–1.29 (m, 11H), 1.52 (d, *J* = 6.8 Hz, 3H), 2.04 (d, *J* = 15.0 Hz, 2H), 2.12 (s, 3H), 3.20 (dd, *J* = 15.0, 6.4 Hz, 1H), 4.05–4.26 (m, 5H), 4.58 (dq, *J* = 8.2, 6.8 Hz, 2H), 6.59 (dd, *J* = 8.0, 1.2 Hz, 1H), 6.73 (dd, *J* = 8.0, 1.3 Hz, 1H), 7.00 (t, *J* = 7.8 Hz, 1H), 7.10 (t, *J* = 7.9 Hz, 1H), 7.24 (dd, *J* = 7.7, 1.2 Hz, 1H), 7.38 (dd, *J* = 7.7, 1.3 Hz, 1H) ppm.

The analytical data is in accordance with the literature.

Diethyl 2,2'-((1-(4,5-bis((*tert*-butyldimethylsilyl)oxy)-9H-thioxanthen-9-ylidene)-2,7-dimethyl-2,3-dihydro-1H-indene-5,6-diyl)bis(oxy))(2*R*,2'*R*)-dipropionate[3]

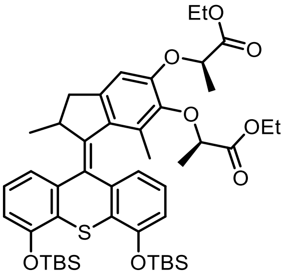

S16
$C_{47}H_{68}O_8SSi_2$
Mw = 832.39 g/mol

Under an argon atmosphere, episulfide **S15** (210 mg, 0.242 mmol, 1 eq.) and PPh_3 (1.27 g, 4.82 mmol, 20 eq.) were dissolved in toluene (3 mL) and the solution was stirred at 120 °C for 3 d. Purification by MPLC (SiO_2; *n*-pentane/ethyl acetate gradient 100:0 ⟶ 90:10) afforded product **S16** as a colorless solid (190 mg, 0.227 mmol, 94%).

(*R,R,R*)-**S16**

^{1}H-NMR (600 MHz, $CDCl_3$): δ = 0.25 (s, 3H), 0.27 (s, 3H), 0.27 (s, 3H), 0.29 (s, 3H), 0.61 (d, *J* = 6.8 Hz, 3H), 0.98 (s, 1H), 1.09 (d, *J* = 1.9 Hz, 17H), 1.19 (s, 3H), 1.28 (dt, *J* = 9.3, 7.1 Hz, 6H), 1.52 (d, *J* = 6.8 Hz, 3H), 1.62 (d, *J* = 6.8 Hz, 3H), 2.33 (d, *J* = 14.8 Hz, 1H), 3.29–3.35 (m, 1H), 4.10 (p, *J* = 6.7 Hz, 1H), 4.18 (dd, *J* = 7.2, 2.3 Hz, 1H), 4.19–4.30 (m, 3H), 4.44 (q, *J* = 6.8 Hz, 1H), 4.77 (q, *J* = 6.7 Hz, 1H), 6.56 (s, 1H), 6.64 (dd, *J* = 7.9, 1.2 Hz, 1H), 6.66 (dd, *J* = 7.7, 1.2 Hz, 1H), 6.69 (dd, *J* = 8.0, 1.1 Hz, 1H), 6.89 (t, *J* = 7.8 Hz, 1H), 7.12 (t, *J* = 7.8 Hz, 1H), 7.29 (dd, *J* = 7.7, 1.2 Hz, 1H) ppm.

^{13}C-NMR (151 MHz, $CDCl_3$): δ = –4.26, –4.18, –3.86, –3.81, 14.28, 14.39, 14.57, 18.53, 18.56, 18.66, 18.87, 19.11, 26.07, 38.15, 39.67, 60.83, 61.32, 73.05, 77.61, 108.31, 115.68, 116.49, 120.45, 121.14, 126.23, 126.64, 127.29, 127.73, 128.32, 131.03, 133.61, 138.48, 141.90, 142.47, 144.99, 145.58, 150.32, 152.36, 152.87, 172.32, 172.57 ppm.

(*S,R,R*)-**S16**

^{1}H-NMR (600 MHz, $CDCl_3$): δ = 0.25 (s, 3H), 0.27 (d, *J* = 1.5 Hz, 6H), 0.29 (s, 3H), 0.62 (d, *J* = 6.8 Hz, 3H), 1.09 (d, *J* = 4.4 Hz, 18H), 1.17–1.26 (m, 10H), 1.45 (d, *J* = 6.8 Hz, 3H), 1.61 (d, *J* = 6.8 Hz, 4H), 2.32 (d, *J* = 14.8 Hz, 1H), 3.33 (ddd, *J* = 14.9, 6.6, 1.2 Hz, 1H), 4.07–4.17 (m, 3H), 4.17–4.29 (m, 2H), 4.75 (qd, *J* = 6.7, 4.6 Hz, 2H), 6.53 (s, 1H), 6.62–6.64 (m, 2H), 6.69 (dd, *J* = 8.0, 1.1 Hz, 1H), 6.86 (t, *J* = 7.8 Hz, 1H), 7.12 (t, *J* = 7.8 Hz, 1H), 7.29 (dd, *J* = 7.7, 1.2 Hz, 1H) ppm.

^{13}C-NMR (151 MHz, $CDCl_3$): δ = –4.23, –4.18, –3.87, –3.79, 14.22, 14.26, 14.55, 18.56, 18.68, 19.09, 26.08, 38.15, 39.61, 60.82, 61.36, 73.12, 77.08, 108.25, 115.67, 116.53, 120.40, 121.12, 126.23, 126.58, 127.32, 127.83, 128.29, 131.05, 133.59, 138.48, 141.94, 142.13, 144.60, 145.65, 149.76, 152.36, 152.89, 172.17, 172.62 ppm.

MS-ESI (ESI+): *m/z* calculated for $C_{47}H_{68}O_6SSi_2H^+$ ($[M+H]^+$): 832.39, found 832.39.

The analytical data is in accordance with the literature.

Postfunctionalization of the motor core

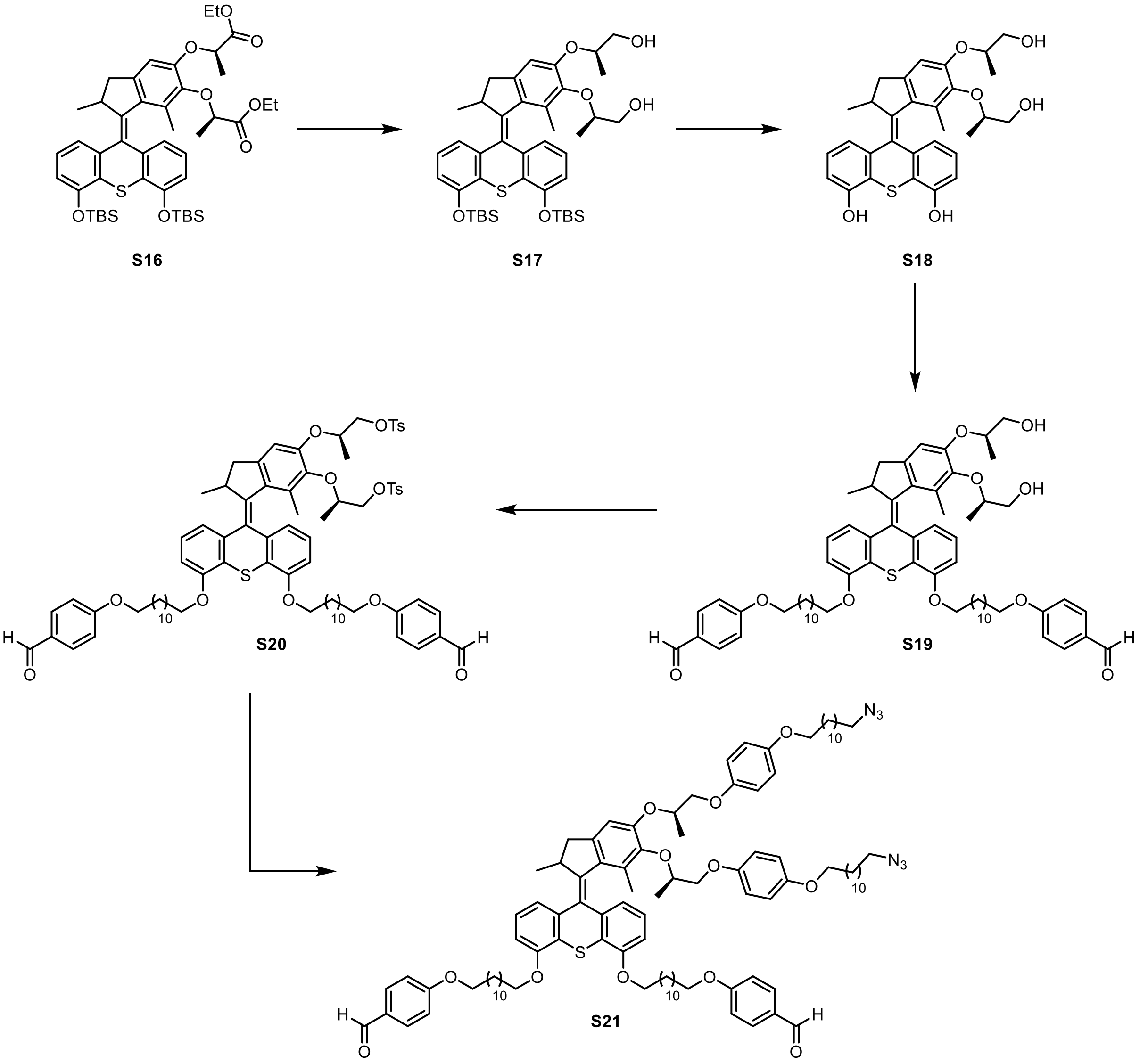

Scheme 3 | Postfunctionalization of the motor core.

(2*R*,2'*R*)-2,2'-((1-(4,5-bis((*tert*-butyldimethylsilyl)oxy)-9*H*-thioxanthen-9-ylidene)-2,7-dimethyl-2,3-dihydro-1*H*-indene-5,6-diyl)bis(oxy))bis(propan-1-ol) (*S*17)

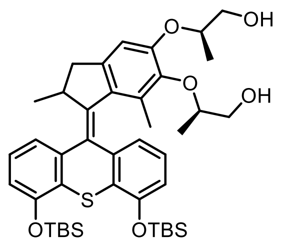

S17
$C_{42}H_{60}O_6SSi_2$
Mw = 749.17 g/mol

Under a nitrogen atmosphere, fully protected motor **S16** (179 mg, 0.215 mmol, 1.00 eq.) was dissolved in dry CH_2Cl_2 (8.2 mL) and diisobutylaluminium hydride (1 M in hexanes, 2.1 mL, 10 eq.) was slowly added at 0 °C. After stirring for 1.5 h at 0 °C, additional 2 eq. of diisobutylaluminium hydride were added after 30 min and 1 h, whereby completion of the reaction was monitored by TLC (SiO_2; *n*-pentane/ethyl acetate 1:1). Then, the reaction was quenched with aq. saturated Rochelle's salt and the mixture stirred for 30 min until a clear phase separation was observed. The reaction mixture was diluted with CH_2Cl_2, washed with water, and dried over $MgSO_4$. Typically, the crude mixture was filtered over a pad of silica gel with CH_2Cl_2/methanol (10:1) and used for the next step without further purification. In order to fully characterize the compound, the crude reaction mixture was once purified by MPLC (SiO_2; *n*-pentane/ethyl acetate gradient 100:0 ⟶ 0:100), which provided product **S17** as a colorless solid (156 mg, 0.21 mmol, 97%).

(*R,R,R*)-**S17**

^{1}H-NMR (600 MHz, C_6D_6): δ = 0.22 (s, 3H), 0.23 (s, 3H), 0.24 (s, 3H), 0.25 (s, 3H), 0.77 (d, *J* = 6.8 Hz, 3H), 0.84 (d, *J* = 6.4 Hz, 3H), 1.15 (s, 9H), 1.17 (s, 9H), 1.20 (d, *J* = 6.3 Hz, 3H), 1.56 (s, 3H), 2.30 (d, *J* = 14.7 Hz, 1H), 3.10 (br s, 2H), 3.33–3.42 (m, 2H), 3.46 (ddd, *J* = 12.4, 8.5, 3.7 Hz, 2H), 3.64 (dd, *J* = 12.2, 2.8 Hz, 1H), 4.04–4.11 (m, 1H), 4.24 (p, *J* = 6.7 Hz, 1H), 4.35 (pd, *J* = 6.5, 3.0 Hz, 1H), 6.62 (dd, *J* = 8.0, 1.2 Hz, 1H), 6.64 (s, 1H), 6.71–6.78 (m, 2H), 6.93 (dd, *J* = 7.6, 1.2 Hz, 1H), 7.06 (t, *J* = 7.9 Hz, 1H), 7.39 (dd, *J* = 7.7, 1.1 Hz, 1H) ppm.

^{13}C-NMR (151 MHz, C_6D_6): δ = −4.2, −4.1, −3.9, −3.8, 15.2, 15.7, 16.7, 18.7, 18.7, 19.2, 26.2, 26.2, 38.5, 39.8, 65.7, 66.8, 76.1, 78.4, 109.5, 116.1, 116.8, 120.9, 121.6, 126.6, 127.1, 128.6, 128.6, 128.9, 131.4, 133.3, 139.2, 142.3, 142.8, 145.0, 146.2, 151.0, 152.9, 153.4 ppm. Signals for some carbon atoms are missing due to signal overlap with C_6D_6.

(*S,R,R*)-**S17**

^{1}H-NMR (600 MHz, C_6D_6): δ = 0.23 (dd, *J* = 11.6, 8.6 Hz, 12H), 0.76 (d, *J* = 6.8 Hz, 3H), 1.02 (d, *J* = 6.4 Hz, 3H), 1.15 (d, *J* = 9.3 Hz, 18H), 1.25 (d, *J* = 6.2 Hz, 3H), 1.47 (s, 3H), 2.30 (d, *J* = 14.7 Hz, 1H), 3.35–3.41 (m, 1H), 3.43 (dd, *J* = 12.3, 3.9 Hz, 1H), 3.48 (dd, *J* = 11.8, 6.8 Hz, 1H), 3.58 (dd, *J* = 11.8, 2.7 Hz, 1H), 3.67 (dd, *J* = 12.3, 2.6 Hz, 1H), 3.82 (s, 2H), 4.05 (tq, *J* = 9.0, 3.0 Hz, 1H), 4.12 (pd, *J* = 6.4, 2.5 Hz, 1H), 4.20 (p, *J* = 6.7 Hz, 1H), 6.61 (dd, *J* = 7.9, 1.2 Hz, 1H), 6.63 (s, 1H), 6.67–6.74 (m, 2H), 6.84 (dd, *J* = 7.6, 1.2 Hz, 1H), 7.05 (t, *J* = 7.9 Hz, 1H), 7.37 (dd, *J* = 7.8, 1.1 Hz, 1H) ppm.

^{13}C-NMR (151 MHz, C_6D_6): δ = −4.2, −4.1, −4.0, −3.9, 15.3, 15.6, 16.7, 18.7, 19.1, 26.2, 38.5, 40.1, 65.4, 66.2, 76.5, 80.2, 110.5, 116.1, 116.8, 120.7, 121.8, 126.5, 126.7, 128.3, 128.7, 129.0, 131.8, 133.8, 139.0, 142.6, 142.6, 146.0, 146.1, 151.1, 152.9, 153.5 ppm. Signals for some carbon atoms are missing due to signal overlap with C_6D_6.

HRMS-ESI (ESI+): *m/z* calculated for $C_{42}H_{60}O_6SSi_2Na^+$ ([M+Na]$^+$): 771.3541, found 771.3523.

9-(5,6-bis(((*R*)-1-hydroxypropan-2-yl)oxy)-2,7-dimethyl-2,3-dihydro-1*H*-inden-1-ylidene)-9*H*-thioxanthene-4,5-diol (*S18*)

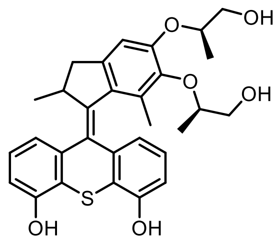

S18
$C_{30}H_{32}O_6S$
Mw = 520.64 g/mol

Under a nitrogen atmosphere, bis-TBS motor **S17** (159 mg, 0.21 mmol, 1.0 eq.) was dissolved in dry THF (14 mL) and TBAF (1M in THF, 0.67 mL, 3.1 eq.) was slowly added at 0 °C. After stirring for 5 min at 0 °C, the reaction mixture was quenched with saturated aq. NH_4Cl, diluted with CH_2Cl_2, washed with water, and dried over $MgSO_4$. Then, the mixture was filtered over a pad of silica gel with CH_2Cl_2/methanol (10:1) and the crude product **S18** (110 mg, 0.2 mmol, 99%) was used for the next step without further purification to avoid decomposition.

4,4'-((((9-(5,6-bis(((*R*)-1-hydroxypropan-2-yl)oxy)-2,7-dimethyl-2,3-dihydro-1*H*-inden-1-ylidene)-9*H*-thioxanthene-4,5-diyl)bis(oxy))bis(dodecane-12,1-diyl))bis(oxy))dibenzaldehyde (S19)

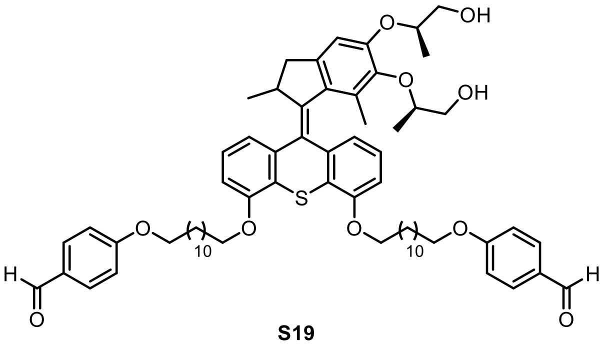

S19
$C_{68}H_{88}O_{10}S$
Mw = 1097.50 g/mol

Under a nitrogen atmosphere, deprotected motor **S18** (110 mg, 0.20 mmol, 1.0 eq.), tosylate **S3** (584 mg, 1.3 mmol, 6.0 eq.), and freshly grinded K_2CO_3 (175 mg, 1.3 mmol, 6.0 eq.) were stirred in DMF (7.5 mL) at 60 °C for 3 days. The reaction mixture was diluted with CH_2Cl_2, washed with water, and dried over $MgSO_4$. Purification by MPLC (SiO_2; *n*-pentane/ethyl acetate gradient 100:0 ⟶ 30:70) afforded product **S19** as a colorless oil (0.11 g, 0.10 mmol, 47% over three steps).

(*R,R,R*)-**S19**

^{1}H-NMR (600 MHz, C_6D_6): δ = 0.80 (d, *J* = 6.8 Hz, 3H), 0.82 (d, *J* = 6.4 Hz, 3H), 1.21 (d, *J* = 6.3 Hz, 3H), 1.23–1.38 (m, 30H), 1.41–1.52 (m, 4H), 1.52–1.60 (m, 7H), 1.66–1.78 (m, 4H), 2.32 (d, *J* = 14.8 Hz, 1H), 3.36–3.49 (m, 4H), 3.52 (t, *J* = 6.4 Hz, 4H), 3.62–3.71 (m, 2H), 3.74 (dt, *J* = 9.1, 6.5 Hz, 1H), 3.82 (ddt, *J* = 21.9, 9.0, 6.4 Hz, 2H), 4.09 (dt, *J* = 6.4, 3.4 Hz, 1H), 4.26–4.39 (m, 2H), 6.44–6.49

(m, 1H), 6.57 (d, *J* = 7.5 Hz, 1H), 6.64–6.69 (m, 5H), 6.84 (t, *J* = 7.9 Hz, 1H), 6.96 (dd, *J* = 7.7, 1.0 Hz, 1H), 7.45 (d, *J* = 7.6 Hz, 1H), 7.57–7.62 (m, 4H), 9.72 (s, 2H) ppm.

^{13}C-NMR (151 MHz, C_6D_6): δ = 15.0, 15.3, 16.4, 18.9, 26.0, 26.1, 26.1, 29.0, 29.2, 29.3, 29.4, 29.5, 29.7, 29.7, 29.8, 38.3, 39.6, 65.3, 66.5, 67.9, 68.6, 75.7, 78.0, 109.0, 114.5, 120.0, 120.6, 125.4, 125.7, 126.3, 126.8, 127.5, 127.6, 127.7, 127.8, 127.8, 128.0, 130.3, 131.1, 131.6, 133.0, 138.5, 141.9, 142.1, 144.6, 145.9, 150.6, 156.1, 156.5, 163.8, 189.4 ppm.
Signals for some carbon atoms are missing due to signal overlap with C_6D_6.

(*S*,*R*,*R*)-**S19**

^{1}H-NMR (600 MHz, C_6D_6): δ = 0.77 (d, *J* = 6.8 Hz, 3H), 1.05 (d, *J* = 6.4 Hz, 3H), 1.23–1.37 (m, 33H), 1.39–1.52 (m, 7H), 1.57 (dq, *J* = 13.7, 6.6 Hz, 4H), 1.62–1.77 (m, 4H), 2.32 (d, *J* = 14.8 Hz, 1H), 3.38–3.46 (m, 2H), 3.49 (dd, *J* = 11.8, 6.5 Hz, 1H), 3.53 (td, *J* = 6.4, 1.1 Hz, 4H), 3.61 (dd, *J* = 11.8, 2.6 Hz, 1H), 3.64–3.70 (m, 2H), 3.74 (dt, *J* = 9.0, 6.4 Hz, 1H), 3.81 (ddt, *J* = 13.0, 8.9, 6.4 Hz, 2H), 4.07 (dq, *J* = 9.8, 6.2, 4.9 Hz, 1H), 4.17 (pd, *J* = 6.3, 2.5 Hz, 1H), 4.27 (p, *J* = 6.7 Hz, 1H), 6.45–6.50 (m, 1H), 6.57 (d, *J* = 7.8 Hz, 1H), 6.66 (s, 1H), 6.68 (d, *J* = 8.6 Hz, 4H), 6.83 (t, *J* = 7.9 Hz, 1H), 6.90 (dd, *J* = 7.7, 1.0 Hz, 1H), 7.42 (d, *J* = 7.7 Hz, 1H), 7.58–7.62 (m, 4H), 9.72 (s, 2H) ppm.

^{13}C-NMR (151 MHz, C_6D6): δ = 15.4, 15.7, 16.8, 19.2, 26.3, 26.4, 26.5, 29.4, 29.6, 29.6, 29.8, 29.9, 30.1, 30.1, 30.1, 38.6, 40.1, 65.4, 66.1, 68.3, 69.0, 69.1, 76.7, 80.0, 109.1, 109.2, 110.7, 114.9, 120.3, 121.1, 125.5, 126.2, 126.6, 126.7, 128.4, 128.6, 128.8, 130.6, 131.9, 132.0, 133.9, 138.6, 142.4, 142.6, 145.9, 146.2, 151.2, 156.4, 156.9, 164.2, 189.9 ppm.
Signals for some carbon atoms are missing due to signal overlap with C_6D_6.

HRMS-ESI (ESI+): *m/z* calculated for $C_{68}H_{88}O_{10}SNa^+$ ([M+Na]$^+$): 1119.5990, found 1119.5973.

(2*R*,2'*R*)-((1-(4,5-bis((12-(4-formylphenoxy)dodecyl)oxy)-9*H*-thioxanthen-9-ylidene)-2,7-dimethyl-2,3-dihydro-1*H*-indene-5,6-diyl)bis(oxy))bis(propane-2,1-diyl) bis(4-methylbenzenesulfonate) (*S*20)

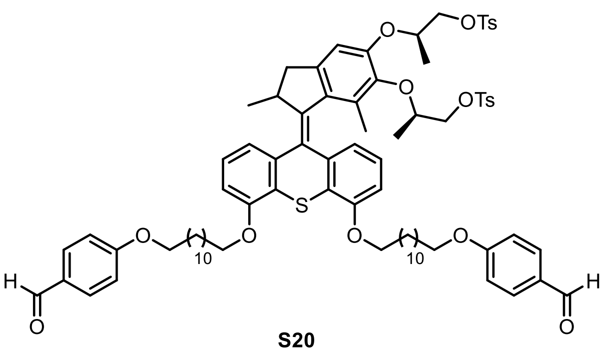

S20
$C_{82}H_{100}O_{14}S_3$
Mw = 1405.87 g/mol

Under a nitrogen atmosphere, bis-alcohol **S19** (110 mg, 0.10 mmol, 1.0 eq.), Et_3N (0.14 mL, 1.0 mmol, 10 eq.), 4-dimethylaminopyridine (12 mg, 0.10 mmol, 1.0 eq.), and 4-toluenesulfonyl chloride (191 mg, 1.00 mmol, 10.0 eq.) were stirred in dry CH_2Cl_2 (3.4 mL) at room temperature for 16 h. The reaction mixture was diluted with CH_2Cl_2, washed with water, and dried over $MgSO_4$. Purification by flash column chromatography (SiO_2; first *n*-pentane/CH_2Cl_2 8:2, then ethyl acetate) provided product **S20** as a colorless oil (140 mg, 99%).

(*R,R,R*)-**S20**

^{1}H-NMR (600 MHz, C_6D_6): δ = 0.71 (d, *J* = 6.8 Hz, 3H), 0.91 (d, *J* = 6.3 Hz, 3H), 1.07 (d, *J* = 6.3 Hz, 3H), 1.21–1.41 (m, 37H), 1.42–1.52 (m, 7H), 1.53–1.60 (m, 4H), 1.67–1.80 (m, 4H), 1.87 (s, 3H), 1.90 (s, 3H), 2.25 (d, *J* = 14.8 Hz, 1H), 3.34 (dd, *J* = 14.8, 6.2 Hz, 1H), 3.53 (t, *J* = 6.5 Hz, 5H), 3.69–3.85 (m, 4H), 3.92–4.04 (m, 5H), 4.15 (dd, *J* = 10.1, 4.5 Hz, 1H), 4.24 (ddd, *J* = 10.2, 7.6, 5.2 Hz, 2H), 4.37 (pd, *J* = 6.2, 4.4 Hz, 1H), 6.53–6.59 (m, 2H), 6.68 (d, *J* = 8.4 Hz, 4H), 6.73–6.83 (m, 5H), 6.92 (dd, *J* = 7.7, 1.1 Hz, 1H), 7.42 (d, *J* = 7.7 Hz, 1H), 7.60 (d, *J* = 8.6 Hz, 4H), 7.80 (dd, *J* = 8.3, 3.0 Hz, 4H), 9.72 (s, 2H) ppm.

^{13}C-NMR (151 MHz, C_6D_6): δ = 15.1, 16.4, 17.4, 19.1, 21.2, 21.3, 26.3, 26.3, 26.5, 26.5, 29.4, 29.6, 29.8, 29.8, 29.9, 29.9, 30.1, 30.1, 38.6, 39.9, 68.3, 69.0, 69.1, 71.7, 71.8, 72.6, 75.3, 109.1, 109.9, 110.5, 114.9, 120.3, 120.9, 125.7, 125.9, 126.7, 127.3, 129.1, 129.9, 130.0, 130.7, 131.7, 131.9, 134.0, 134.3, 134.3, 138.7, 142.0, 142.4, 144.4, 144.7, 145.3, 145.9, 150.6, 156.4, 157.0, 164.2, 189.8 ppm.
Signals for some carbon atoms are missing due to signal overlap with C_6D_6.

(*S,R,R*)-**S20**

^{1}H-NMR (600 MHz, C_6D_6): δ = 0.71 (d, *J* = 6.7 Hz, 3H), 0.98 (d, *J* = 6.4 Hz, 3H), 1.09 (d, *J* = 6.4 Hz, 3H), 1.24–1.37 (m, 30H), 1.38 (s, 3H), 1.47 (dtd, *J* = 15.4, 12.2, 9.1, 4.5 Hz, 4H), 1.53–1.61 (m, 4H), 1.70 (ddp, *J* = 19.7, 13.1, 6.1 Hz, 4H), 1.83 (s, 3H), 1.87 (s, 3H), 2.25 (d, *J* = 14.8 Hz, 1H), 3.33 (dd, *J* = 14.7, 6.1 Hz, 1H), 3.53 (td, *J* = 6.4, 1.5 Hz, 4H), 3.70 (ddt, *J* = 36.7, 9.2, 6.6 Hz, 2H), 3.76–3.86 (m, 2H), 3.99 (dd, *J* = 10.6, 4.2 Hz, 1H), 4.03 (dd, *J* = 10.2, 5.3 Hz, 1H), 4.12 (dd, *J* = 10.6, 5.8 Hz, 1H), 4.23 (dq, *J* = 10.3, 5.8, 4.8 Hz, 2H), 4.27–4.34 (m, 1H), 4.39 (h, *J* = 6.3 Hz, 1H), 6.47 (dd, *J* = 7.7, 1.5 Hz, 1H), 6.51 – 6.58 (m, 2H), 6.68 (d, *J* = 8.8 Hz, 4H), 6.75 (dd, *J* = 17.4, 7.9 Hz, 4H), 6.84 – 6.92 (m, 2H), 7.39 (d, *J* = 7.7 Hz, 1H), 7.60 (d, *J* = 8.5 Hz, 4H), 7.77–7.82 (m, 4H), 9.72 (s, 2H).

^{13}C-NMR (151 MHz, C_6D_6): δ = 15.2, 16.2, 17.2, 19.1, 21.2, 21.2, 26.3, 26.4, 26.5, 29.4, 29.6, 29.7, 29.8, 29.9, 30.1, 30.1, 30.1, 30.1, 30.1, 38.6, 39.9, 68.3, 69.0, 69.0, 71.9, 72.1, 72.7, 75.5, 109.1, 109.3,

110.4, 114.9, 120.3, 121.0, 125.6, 126.0, 126.7, 127.0, 128.3, 128.9, 129.9, 130.0, 130.7, 131.8, 131.9, 134.0, 134.1, 138.6, 142.2, 142.3, 144.4, 144.5, 145.3, 146.0, 150.2, 156.4, 156.9, 164.2, 164.2, 189.8 ppm.
Signals for some carbon atoms are missing due to signal overlap with C_6D_6.

HRMS-ESI (ESI+): *m/z* calculated for $C_{82}H_{100}O_{14}S_3Na^+$ ($[M+Na]^+$): 1427.6167, found 1427.6151.

2.3.5 4,4'-((((9-(5,6-bis(((*R*)-1-(4-((12-azidododecyl)oxy)phenoxy)propan-2-yl)oxy)-2,7-dimethyl-2,3-dihydro-1*H*-inden-1-ylidene)-9*H*-thioxanthene-4,5-diyl)bis(oxy))bis(dodecane-12,1-diyl))bis(oxy))dibenzaldehyde (S21)

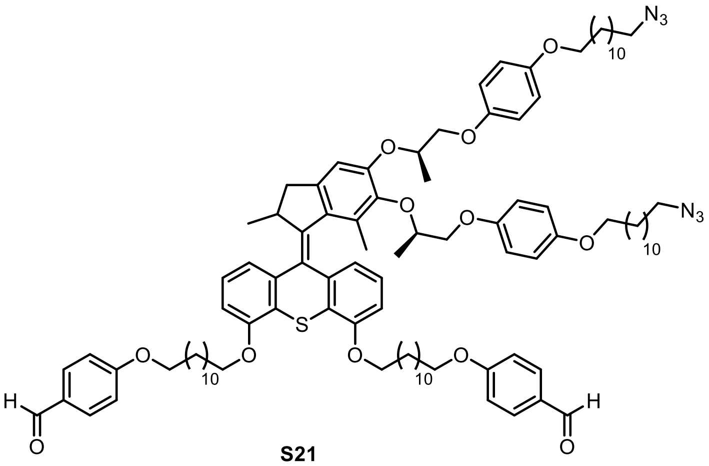

S21
$C_{104}H_{142}N_6O_{12}S$
Mw = 1700.37 g/mol

Under a nitrogen atmosphere, bis-tosylate **S20** (30 mg, 21 μmol, 1.0 eq.), phenol **S5** (0.20 g, 0.64 mmol, 30.0 eq.), and Cs_2CO_3 (0.21 g, 0.64 mmol, 30.0 eq.) were stirred in a mixture of DMF/THF (1 mL, 7:3) at 55 °C for 9 d. The reaction mixture was diluted with CH_2Cl_2, washed with water, dried over $MgSO_4$, and filtered over a pad of silica gel with ethyl acetate. After purification by MPLC (SiO_2; first *n*-pentane/CH_2Cl_2 1:1, then *n*-pentane/ethyl acetate gradient 100:0 ⟶ 80:20), product **S21** was obtained as an off-white solid (18 mg, 11 μmol, 50%).

(*R,R,R*)-**S21**

^{1}H-NMR (600 MHz, C_6D_6): δ = 0.78 (d, *J* = 6.7 Hz, 3H), 1.05–1.15 (m, 8H), 1.16–1.38 (m, 57H), 1.39–1.60 (m, 15H), 1.65 (s, 3H), 1.67–1.75 (m, 7H), 2.33 (d, *J* = 14.8 Hz, 1H), 2.71 (t, *J* = 6.8 Hz, 4H), 3.43 (dd, *J* = 14.9, 6.1 Hz, 1H), 3.52 (t, *J* = 6.5 Hz, 4H), 3.65–3.79 (m, 9H), 3.79–3.91 (m, 1H), 3.96 (dd, *J* = 9.7, 5.8 Hz, 1H), 4.05 (dd, *J* = 9.3, 4.0 Hz, 1H), 4.29 (p, *J* = 6.7 Hz, 1H), 4.57–4.62 (m, 1H), 4.79–4.85 (m, 1H), 6.41–6.48 (m, 1H), 6.56 (d, *J* = 8.1 Hz, 1H), 6.67 (d, *J* = 8.7 Hz, 4H), 6.79–6.92 (m, 10H), 6.95–7.01 (m, 1H), 7.39–7.46 (m, 1H), 7.56–7.63 (m, 4H), 9.72 (s, 2H) ppm.

^{13}C-NMR (151 MHz, C_6D_6): δ = 15.2, 17.4, 18.5, 19.2, 26.3, 26.5, 26.5, 26.6, 27.0, 29.0, 29.4, 29.5, 29.6, 29.6, 29.8, 29.9, 29.9, 29.9, 29.9, 30.0, 30.0, 30.0, 30.1, 30.1, 30.2, 38.8, 40.1, 51.4, 68.3, 68.5, 68.5, 69.0, 69.0, 71.7, 71.7, 73.4, 76.6, 109.0, 109.4, 110.0, 114.9, 115.6, 115.7, 116.0, 116.0, 120.4, 121.0, 125.8, 126.1, 126.6, 127.1, 127.6, 128.7, 130.7, 131.8, 131.9, 133.5, 138.9, 142.2, 142.4, 146.1, 146.4, 151.7, 153.4, 153.8, 154.1, 154.3, 156.4, 156.9, 164.1, 189.8 ppm. Signals for some carbon atoms are missing due to signal overlap with C_6D_6.

(S,R,R)-**S21**

^{1}H-NMR (600 MHz, C_6D_6): δ = 0.80 (d, J = 6.8 Hz, 3H), 1.08–1.12 (m, 7H), 1.19 (m, 5H), 1.20–1.35 (m, 42H), 1.37–1.52 (m, 13H), 1.52–1.60 (m, 4H), 1.63–1.76 (m, 11H), 2.33 (d, J = 14.8 Hz, 1H), 2.72 (td, J = 6.9, 2.8 Hz, 4H), 3.43 (dd, J = 14.8, 6.1 Hz, 2H), 3.52 (t, J = 6.4 Hz, 4H), 3.63–3.77 (m, 7H), 3.77–3.83 (m, 3H), 3.93 (dd, J = 9.4, 6.5 Hz, 1H), 3.99 (dd, J = 9.6, 5.4 Hz, 1H), 4.21 (dd, J = 9.4, 4.6 Hz, 1H), 4.29 (p, J = 6.7 Hz, 2H), 4.61–4.67 (m, 2H), 4.75–4.83 (m, 2H), 6.47 (d, J = 7.4 Hz, 1H), 6.56 (d, J = 7.8 Hz, 1H), 6.67 (d, J = 8.1 Hz, 4H), 6.77–6.90 (m, 11H), 7.00 (dd, J = 7.7, 0.9 Hz, 2H), 7.14 (d, J = 7.9 Hz, 1H), 7.43 (d, J = 7.7 Hz, 1H), 7.57–7.63 (m, 4H), 9.72 (s, 2H) ppm.

^{13}C-NMR (151 MHz, C_6D_6): δ = 15.1, 16.9, 18.1, 18.9, 26.0, 26.1, 26.1, 26.2, 26.6, 28.7, 29.0, 29.1, 29.2, 29.3, 29.4, 29.5, 29.5, 29.5, 29.6, 29.6, 29.7, 29.7, 29.7, 38.3, 39.6, 51.0, 67.9, 68.1, 68.1, 68.6, 71.5, 71.9, 72.8, 76.3, 108.7, 108.9, 109.9, 114.5, 115.3, 115.4, 115.5, 115.6, 120.0, 120.8, 125.4, 125.7, 126.3, 126.6, 128.3, 130.3, 131.5, 131.6, 133.3, 138.4, 141.6, 142.0, 145.9, 146.0, 150.9, 153.0, 153.3, 153.7, 153.9, 156.0, 156.5, 163.8, 189.4 ppm.
Signals for some carbon atoms are missing due to signal overlap with C_6D_6.

HRMS-ESI (ESI+): m/z calculated for $C_{104}H_{142}N_6O_{12}SNa^+$ ($[M+Na]^+$): 1723.0332, found 1723.0283.

General procedure for the formation of (*S,R,R*)- and (*R,R,R*)-±n

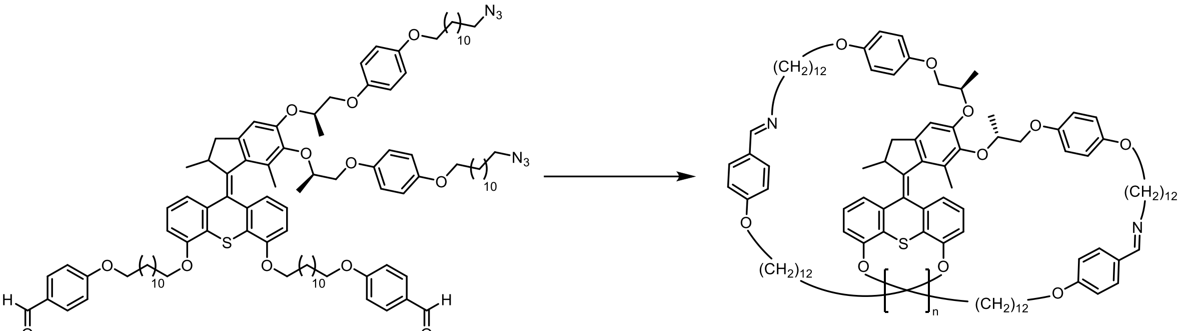

S21 → **±n**

A stock solution of motor **S21** (1.0 mg, 0.60 µmol, 1.0 eq.) in C_6D_6 was lyophilized in a J. Young NMR tube. Then, PPh_3 (0.6 mg, 2.4 mmol, 4.0 eq.) was added and the tube was put under high vacuum for 16 h. After that, the solids were dissolved in dry (distilled from CaH_2) and degassed (three freeze-pump-thaw cycles) C_6D_6 or toluene-*d*8 (0.6 mL) inside a glovebox and activated molecular sieves (3 Å) were added. The sealed NMR tube was taken out of the glovebox and heated to 60 °C for 7 d in an oil bath under exclusion of light. Subsequently, the molecular sieves were removed inside a glovebox and the sample was used for further experiments without purification. Since the system is dynamic, the formation of the bridged bis-macrocyclic **±n** is concentration dependent. Increasing the concentration leads to formation of an insoluble polymer. The conversion of **S21** to **±n** was followed using ^{1}H-NMR spectroscopy by observing the decrease of the aldehyde signal (ca. 9.7 ppm) and increase of imine signals (ca. 8.1 ppm). Typically, bis-macrocyclic **±n** forms in 90-95%.

Note that PPh_3 does not engage in any kind of exchange reaction with the imines. Therefore, no influence of PPh_3 on the relaxation rate of wound **±n** was observed.

Diastereomer (*S,R,R*)-**±n** and its machine-like function was characterized and investigated by HRMS, IMS, CD and NMR spectroscopy, SAXS, and computational studies. Our data supports that the formation of oligomers under our experimental conditions is negligible.

HRMS-ESI (ESI+): *m/z* calculated for $C_{104}H_{143}N_2O_{10}S$ $[M+H]^+$: 1613.0492, found: 1613.0484

Comparison of ^{1}H-NMR spectra of S21, equilibrated, illuminated, and partially relaxed ±n samples in C_6D_6 at 10 °C

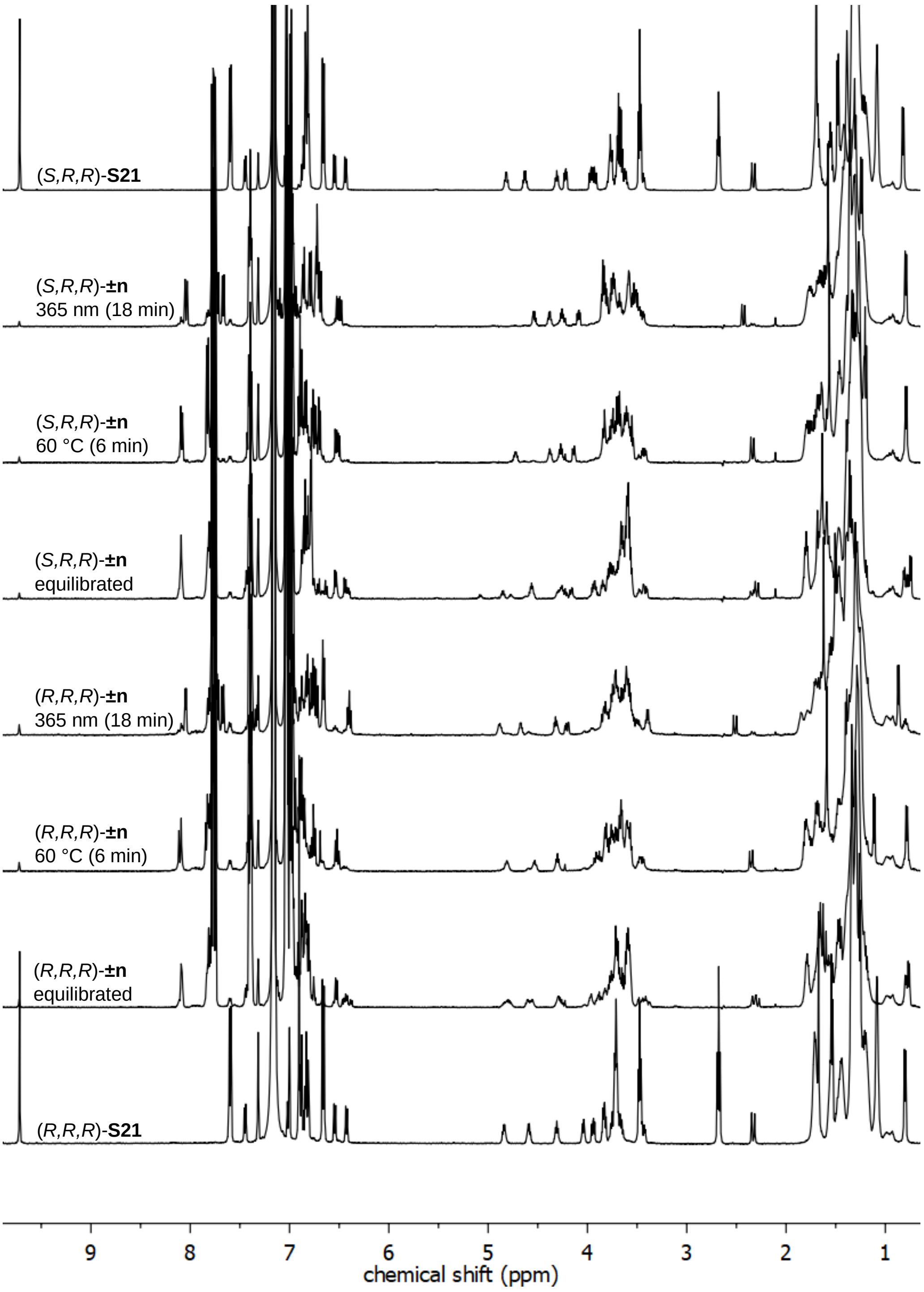

Fig. 1 | Comparison of 1H-NMR spectra of S21 and ±n samples at different relaxation stages. C_6D_6, c = 1 mM, 10 °C, 500 MHz.

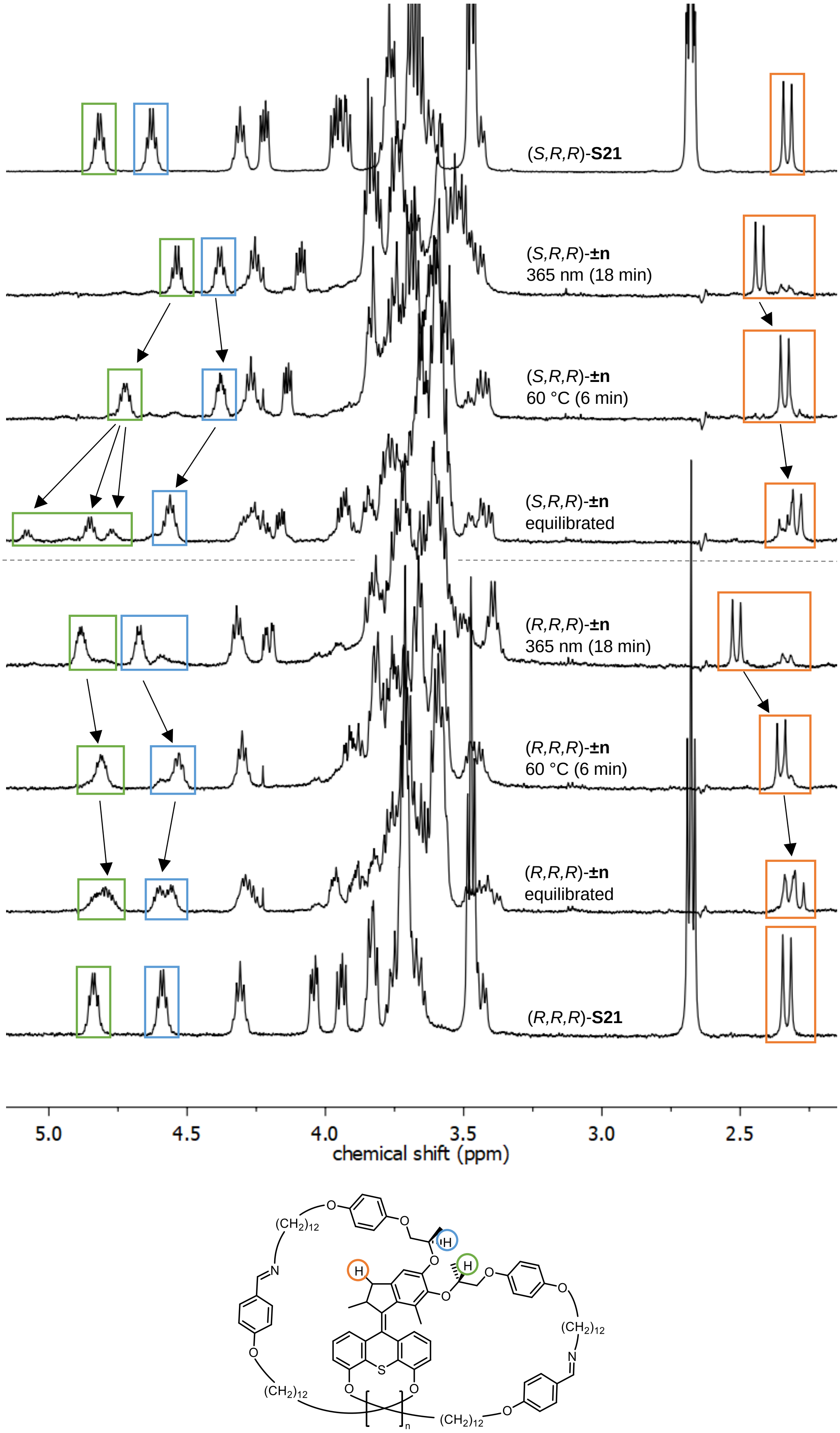

Supplementary Fig. 2 | Comparison of ^{1}H-NMR spectra of S21 and ±n samples at different relaxation stages from 5.2 to 2.2 ppm. Arrows indicate interconversion of key signals. C_6D_6, c = 1 mM, 10 °C, 500 MHz.

2D NMR spectra of equilibrated (*R,R,R*)-±n sample

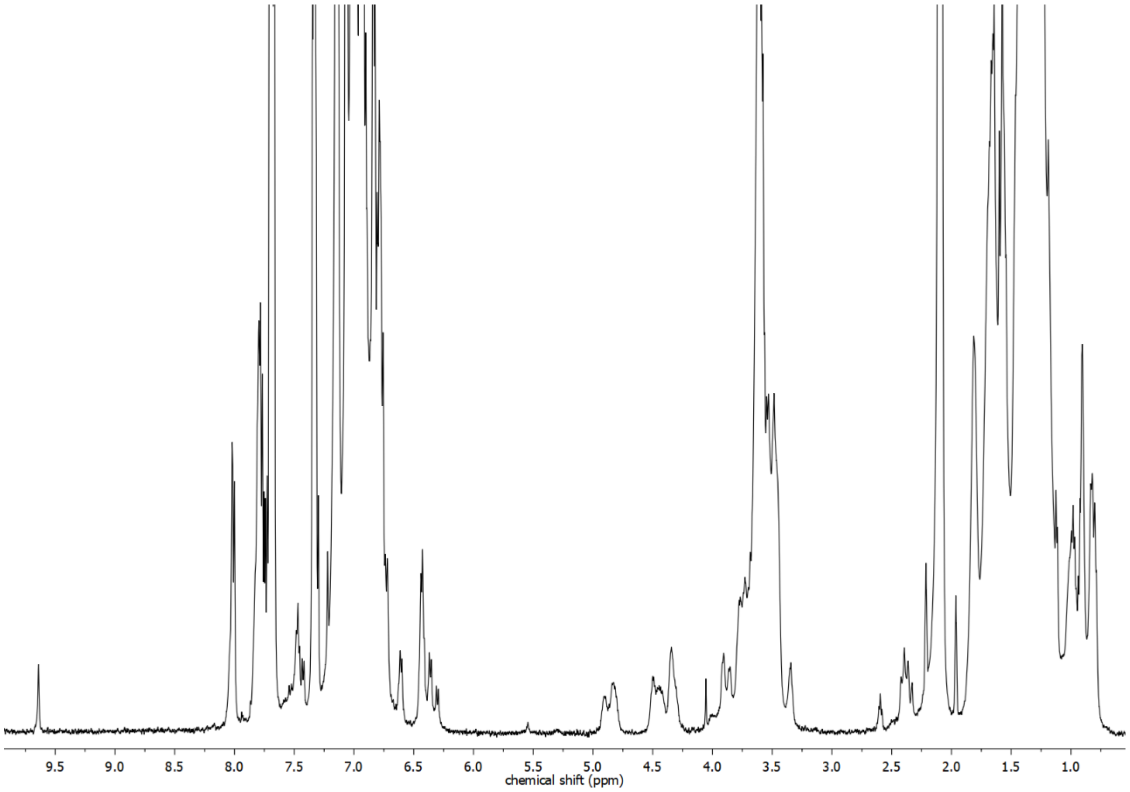

Supplementary Fig. S3 | ^{1}H-NMR spectrum of an equilibrated (*R,R,R*)-±n sample. Toluene-*d*8, c = 1 mM, −40 °C, 500 MHz.

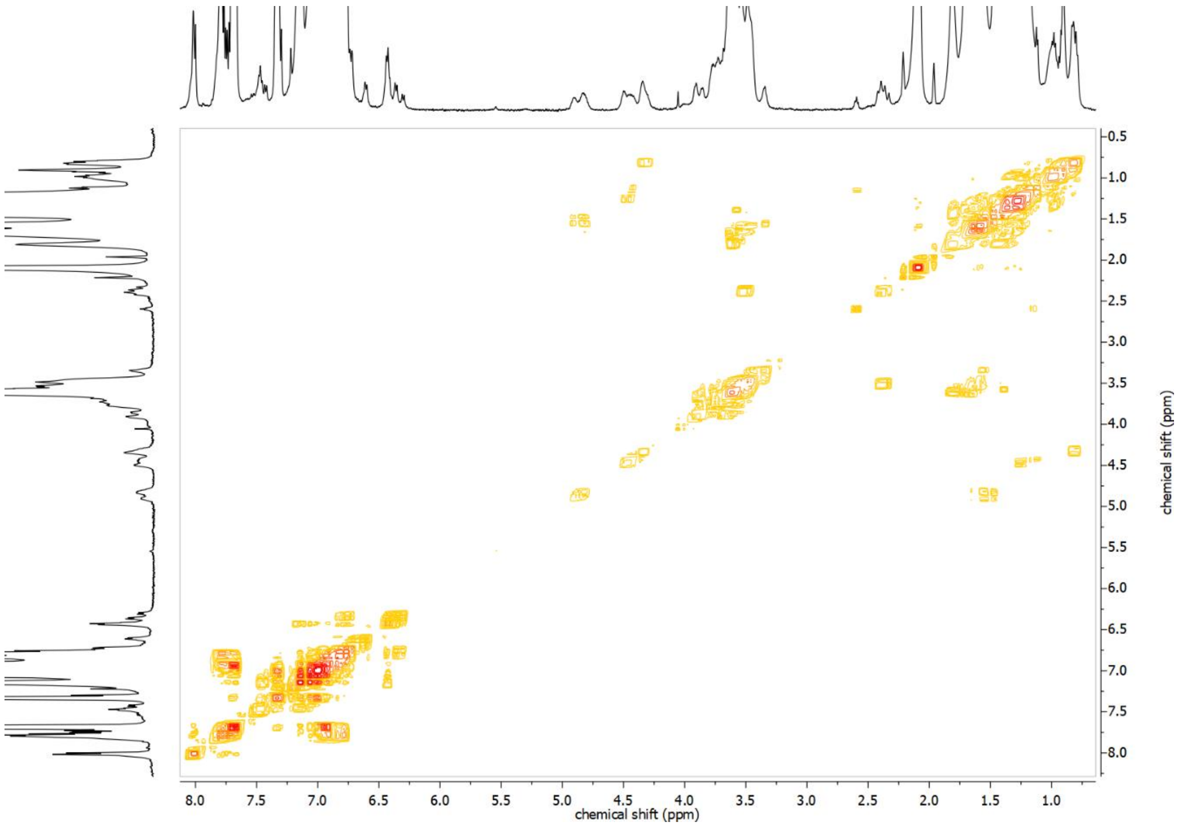

Supplementary Fig. 4 | ^{1}H, ^{1}H COSY NMR spectrum of an equilibrated (*R,R,R*)-±n sample. Toluene-*d*8, c = 1 mM, −40 °C, 500 MHz.

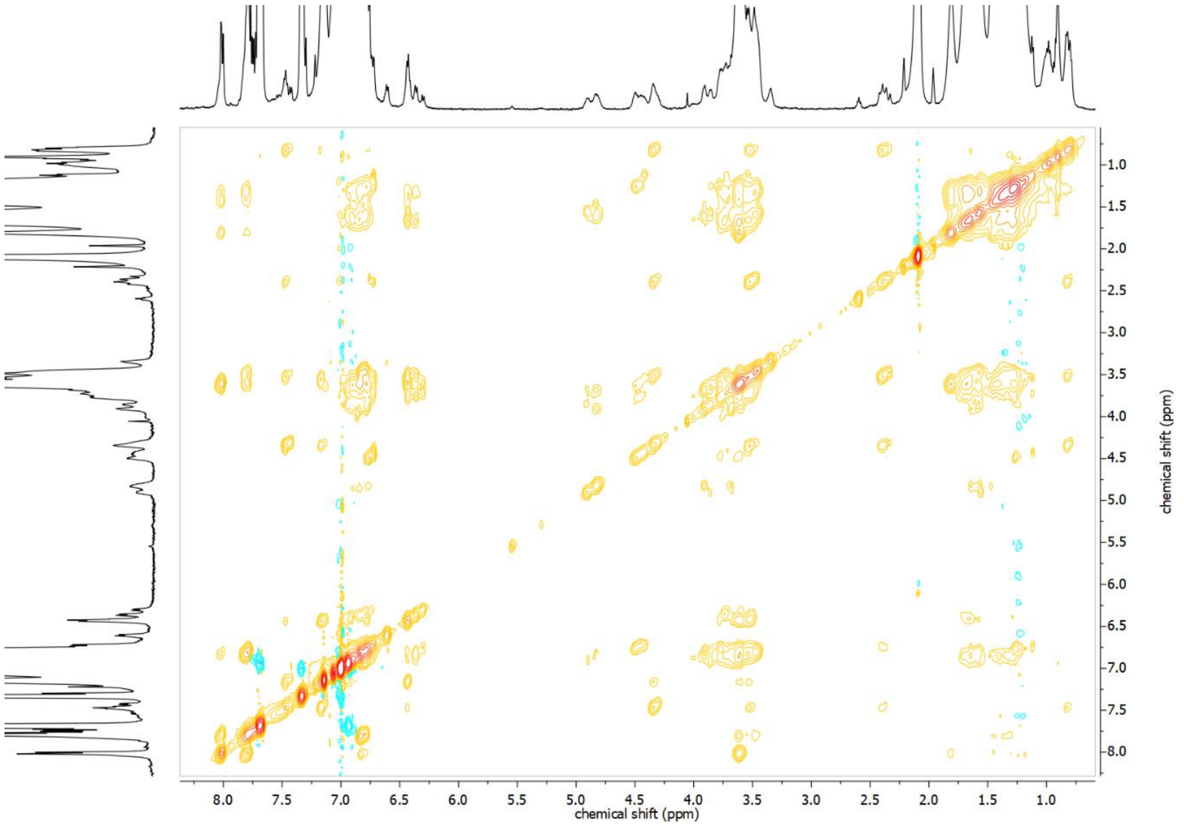

Supplementary Fig. 5 | ^{1}H, ^{1}H NOESY NMR spectrum of an equilibrated (*R,R,R*)-±n sample. Toluene-*d*8, c = 1 mM, −40 °C, 500 MHz.

NMR spectra of illuminated (*R,R,R*)-±n sample

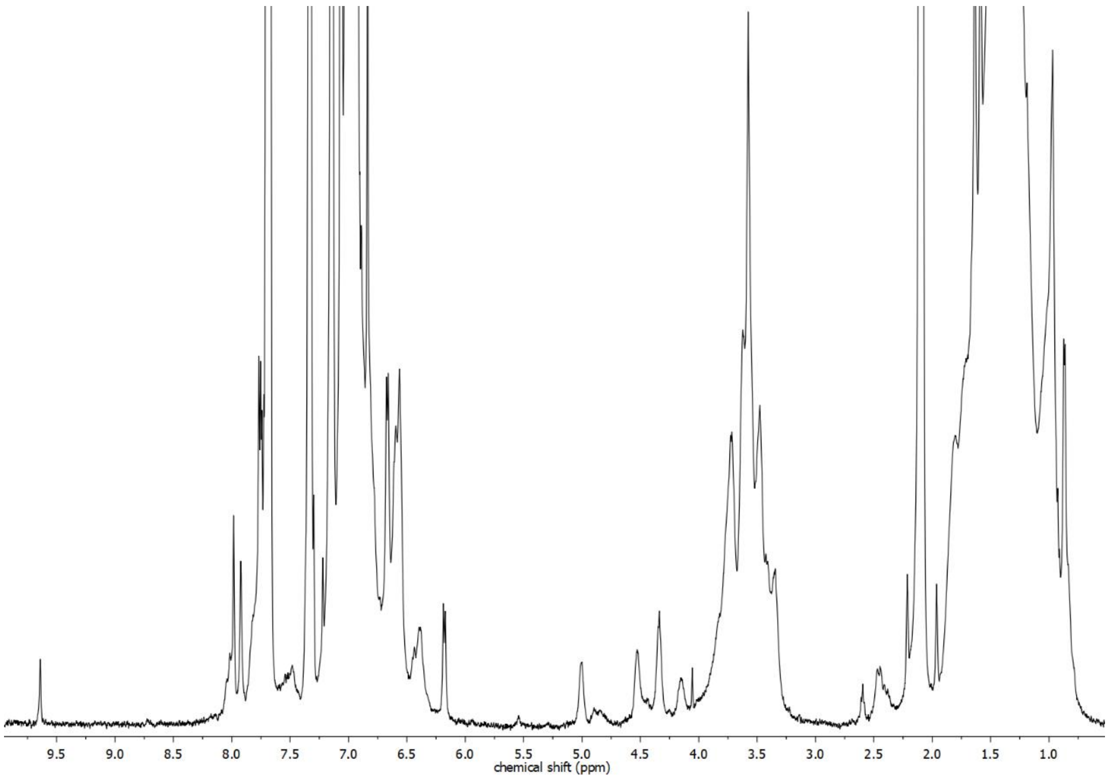

Supplementary Fig. 6 | ^{1}H-NMR spectrum after illumination of (*R,R,R*)-±n. Toluene-*d*8, c = 1 mM, −40 °C, 500 MHz, λ_{irr} = 365 nm (18 min).

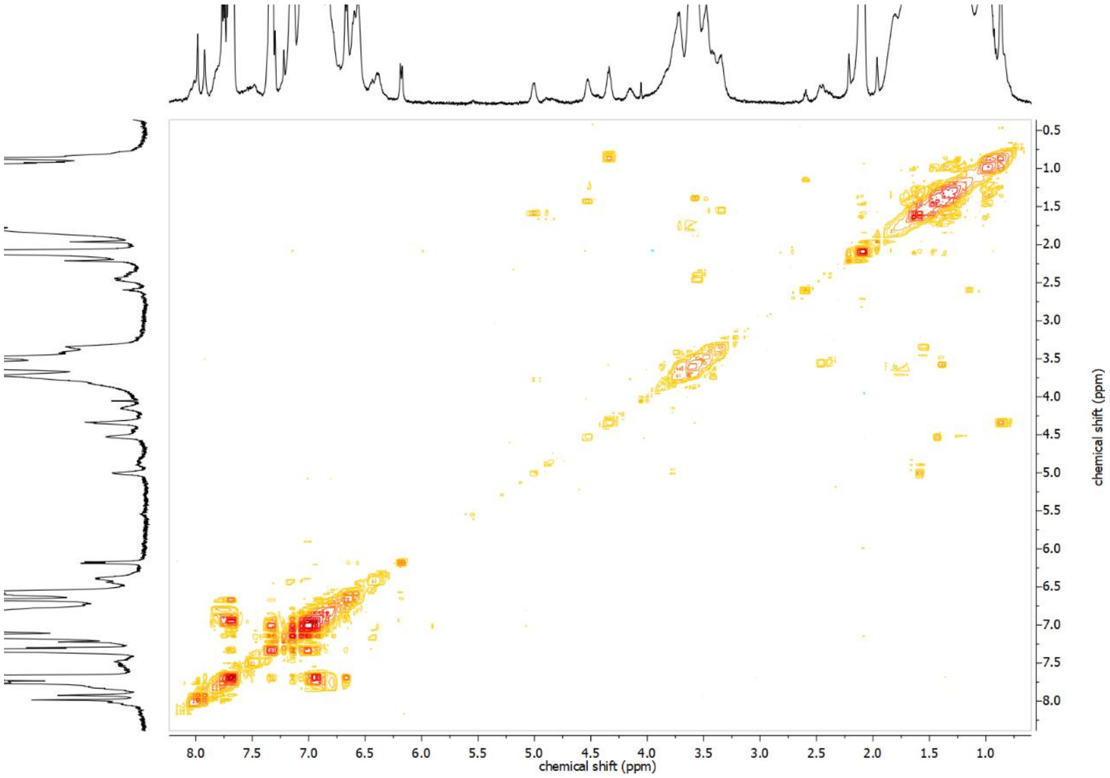

Supplementary Fig. 7 | ¹H, ¹H COSY NMR spectrum after illumination of (*R,R,R*)-±n. Toluene-*d*8, c = 1 mM, −40 °C, 500 MHz, λ_{irr} = 365 nm (18 min).

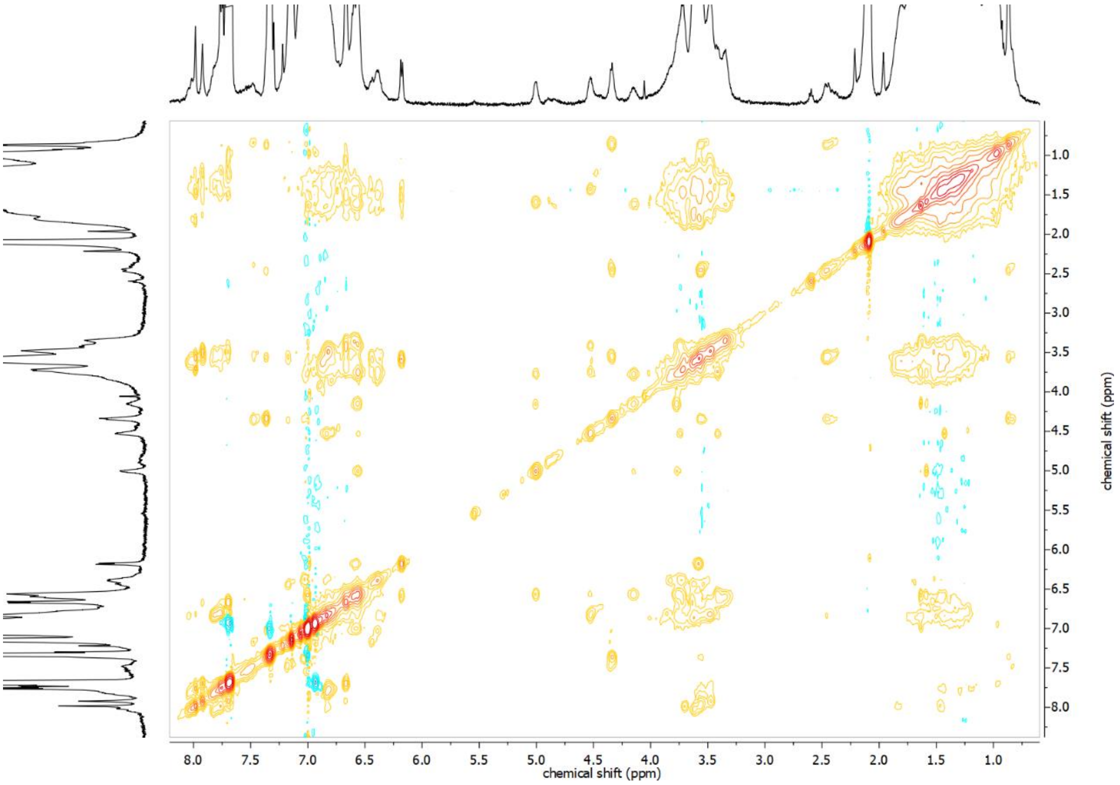

Supplementary Fig. 8 | ¹H, ¹H NOESY NMR spectrum after illumination of (*R,R,R*)-±n. Toluene-*d*8, c = 1 mM, −40 °C, 500 MHz, λ_{irr} = 365 nm (18 min).

NMR spectra of partially relaxed (*R*,*R*,*R*)-±n sample

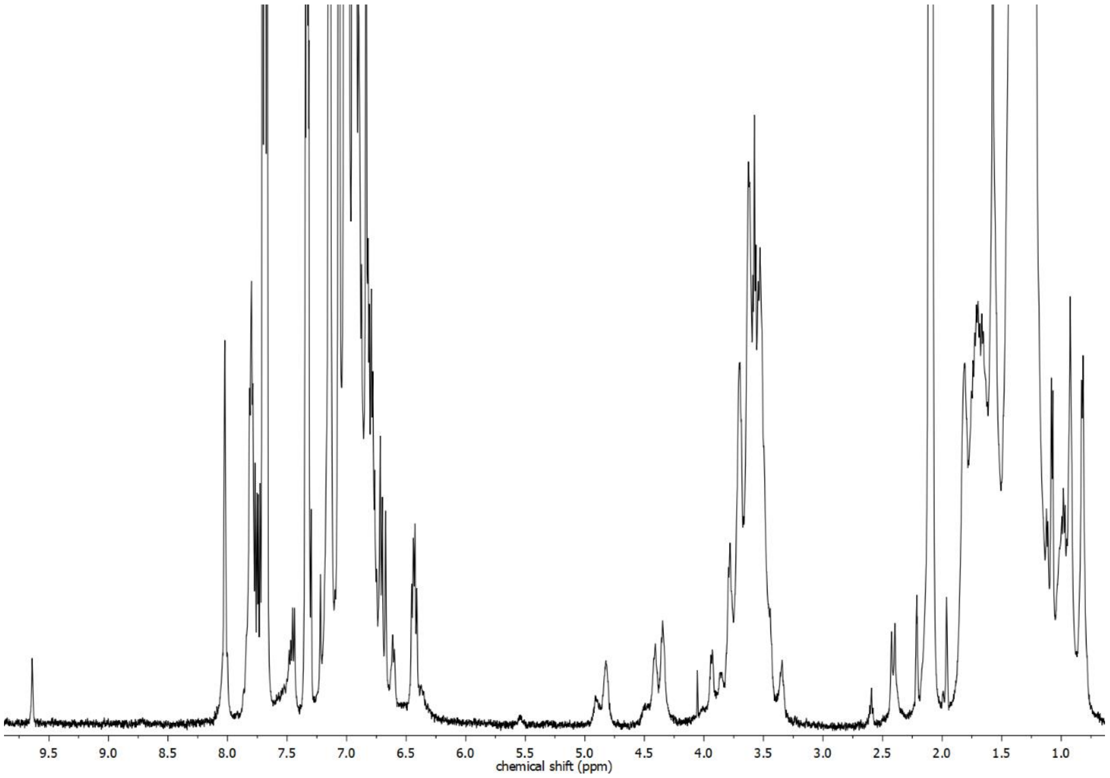

Supplementary Fig. 9 | ^{1}H-NMR spectrum of (*R*,*R*,*R*)-±n sample after 6 min at 60 °C. Toluene-*d*8, c = 1 mM, −40 °C, 500 MHz.

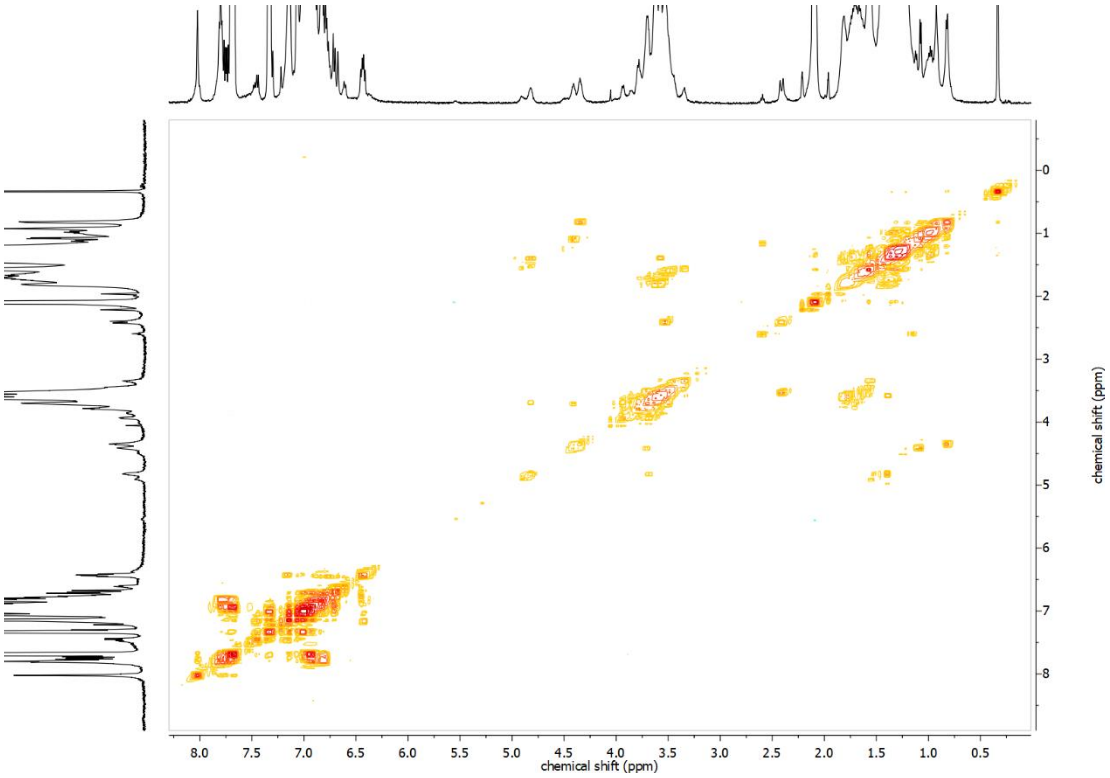

Supplementary Fig. 10 | ^{1}H,^{1}H COSY NMR spectrum of (*R*,*R*,*R*)-±n sample after 6 min at 60 °C. Toluene-*d*8, c = 1 mM, −40 °C, 500 MHz.

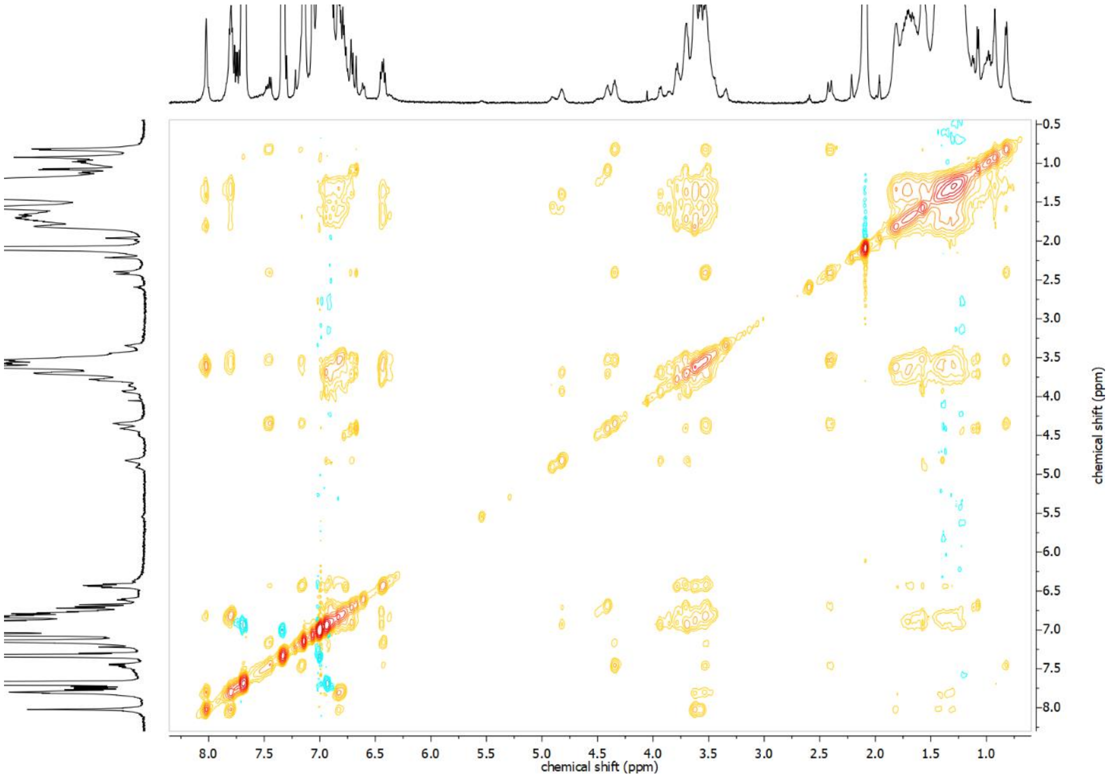

Supplementary Fig. 11 | ¹H,¹H NOESY NMR spectrum of (*R,R,R*)-±n sample after 6 min at 60 °C. Toluene-*d*8, c = 1 mM, −40 °C, 500 MHz.

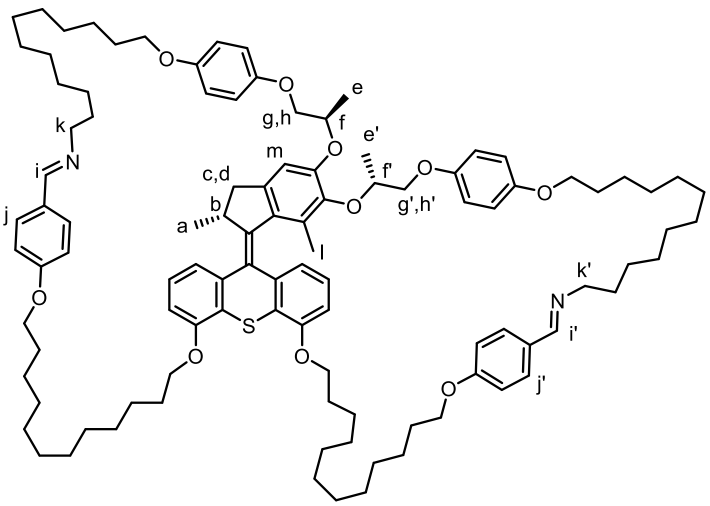

Supplementary Tab. 1 | Chemical shifts of important proton signal of (*R,R,R*)-±n. Toluene-*d*8, c = 1 mM, −40 °C, 500 MHz.			
**Protons**	**Equilibrated**	**Illuminated**	**Partially relaxed**
**a**	0.82	0.87	0.82
**b**	4.33	4.34	4.34
**c,d**	2.39, 3.51	2.44, 3.55	2.40, 3.53
**e,e'**	1.28, 1.50	1.43, 1.59	1.08, 1.40
**f,f'**	4.44, 4.50, 4.82, 4.91	4.53, 5.00	4.41, 4.82
**g,g',h,h'**	3.56, 3.60, 3.69, 3.75, 3.85, 3.90	3.41, 3.74, 3.77, 4.16	3.52, 3.69, 3.72, 3.93
**i,i'**	8.00, 8.02	7.92, 7.98	8.00
**j,j'**	7.79	7.69, 7.76	7.80
**k, k'**	3.61	3.49, 3.59	3.60
**l**	1.61	1.64	-
**m**	6.75	6.83	6.69

NMR spectra of equilibrated (*S*,*R*,*R*)-±n sample

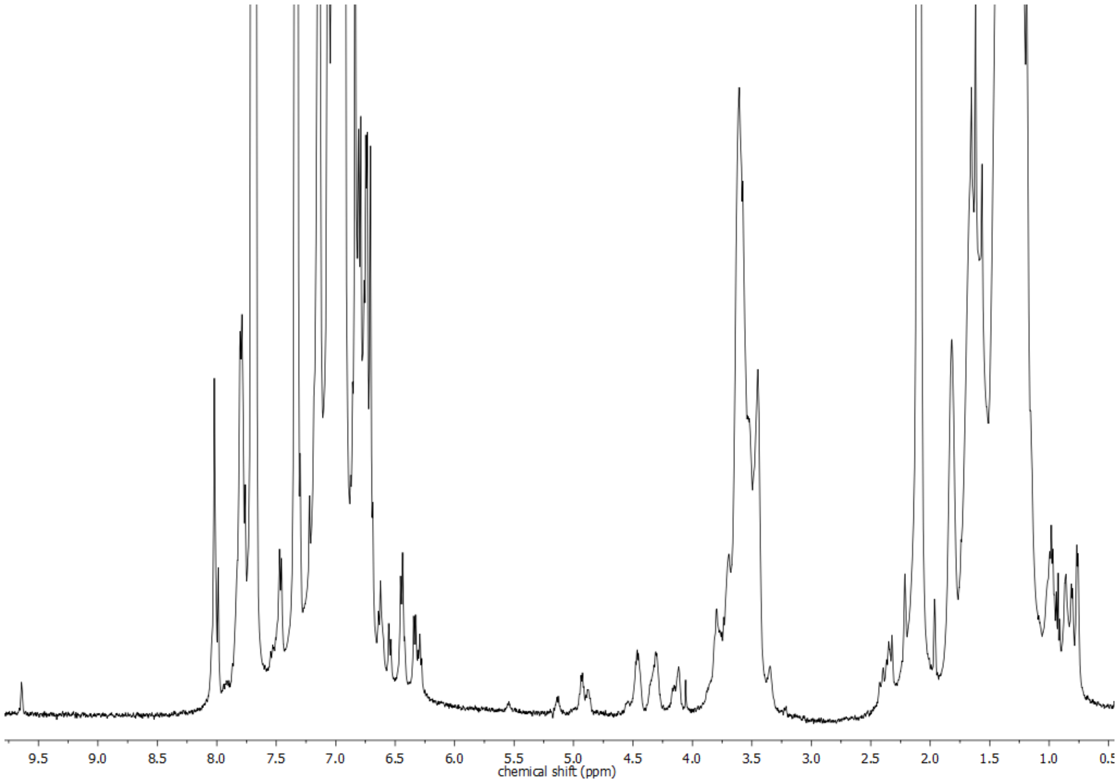

Supplementary Fig. 12 | ^{1}H-NMR spectrum of an equilibrated (*S*,*R*,*R*)-±n sample. Toluene-*d*8, c = 1 mM, −40 °C, 500 MHz.

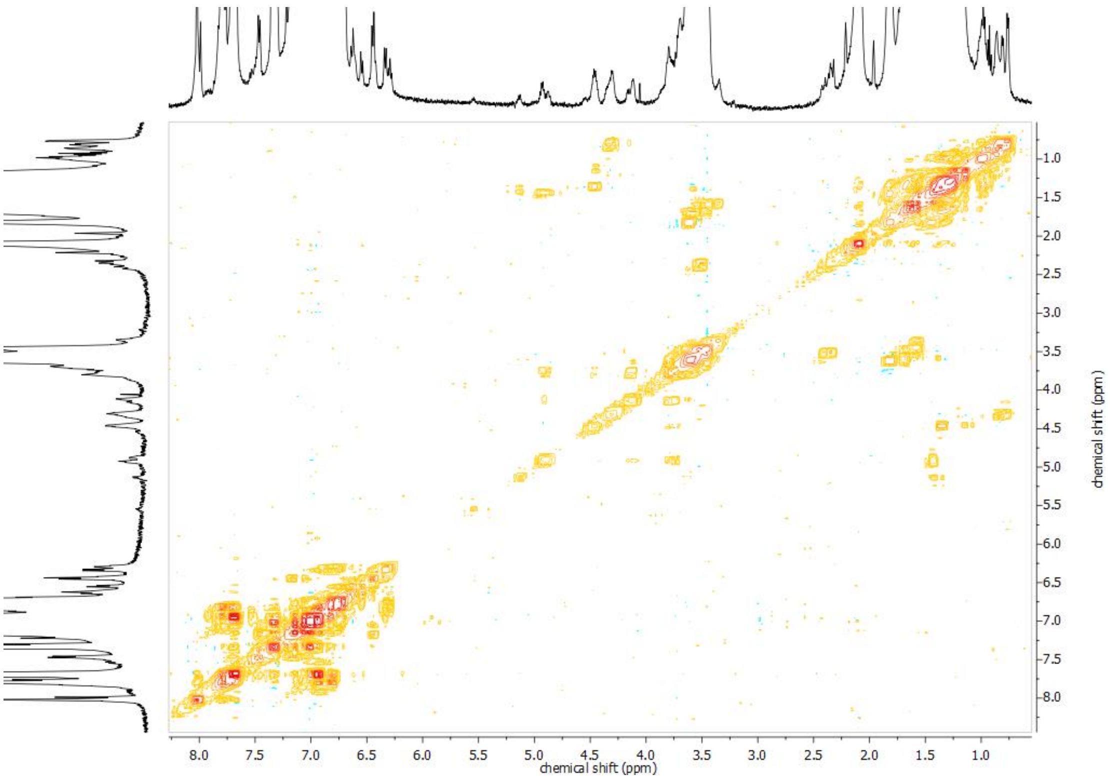

Supplementary Fig. 13 | ^{1}H, ^{1}H COSY NMR spectrum of an equilibrated (*S*,*R*,*R*)-±n sample. Toluene-*d*8, c = 1 mM, −40 °C, 500 MHz.

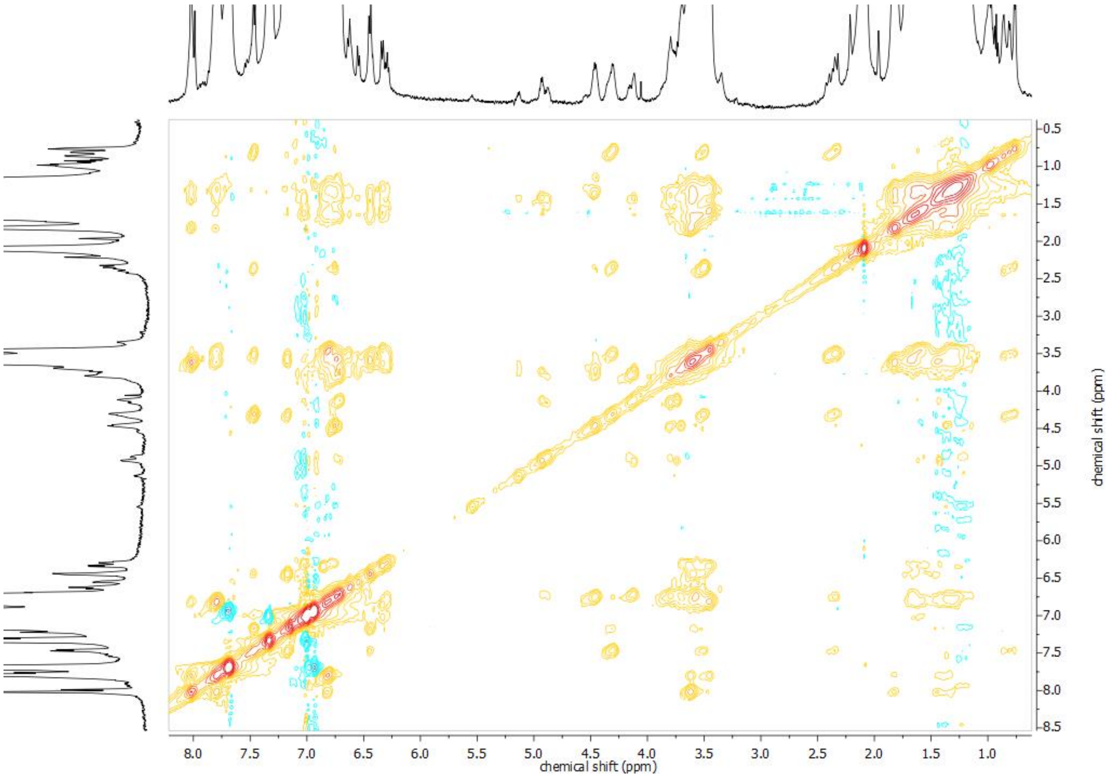

Supplementary Fig. 14 | ^{1}H, ^{1}H NOESY NMR spectrum of an equilibrated (*S,R,R*)-±n sample. Toluene-*d*8, c = 1 mM, −40 °C, 500 MHz.

NMR spectra of illuminated (*S,R,R*)-±n sample

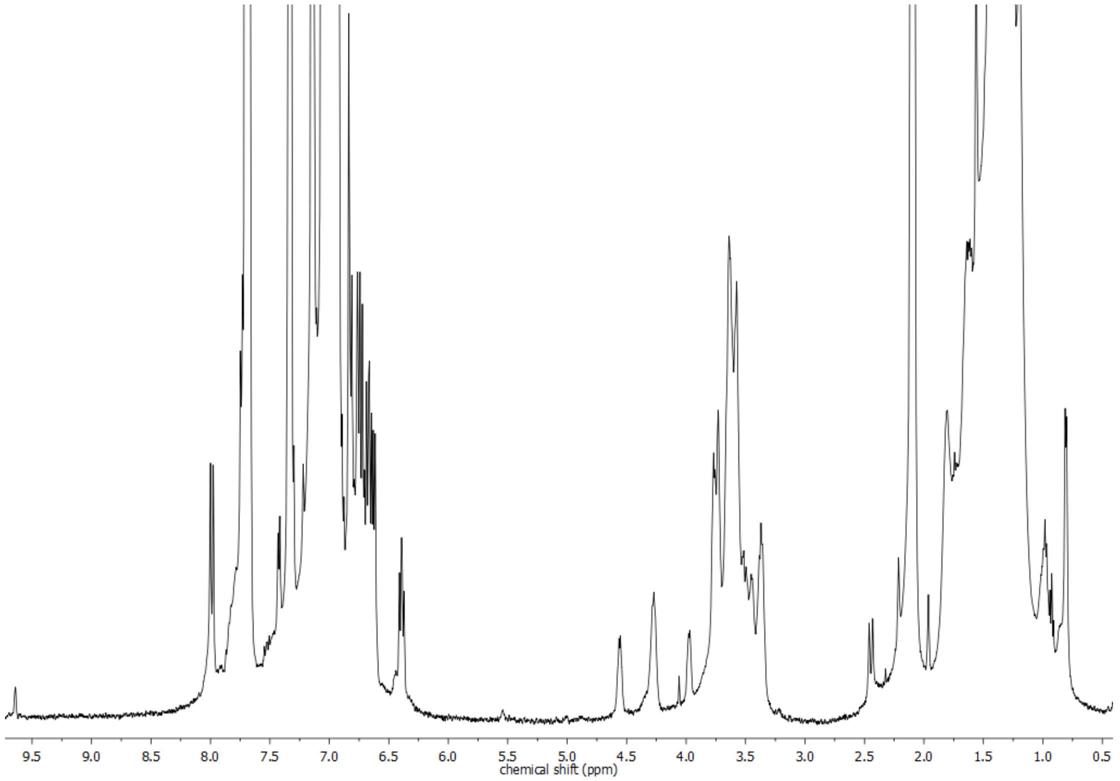

Supplementary Fig. 15 | ^{1}H-NMR spectrum after illumination of (*S,R,R*)-±n. Toluene-*d*8, c = 1 mM, −40 °C, 500 MHz, λ_{irr} = 365 nm (18 min).

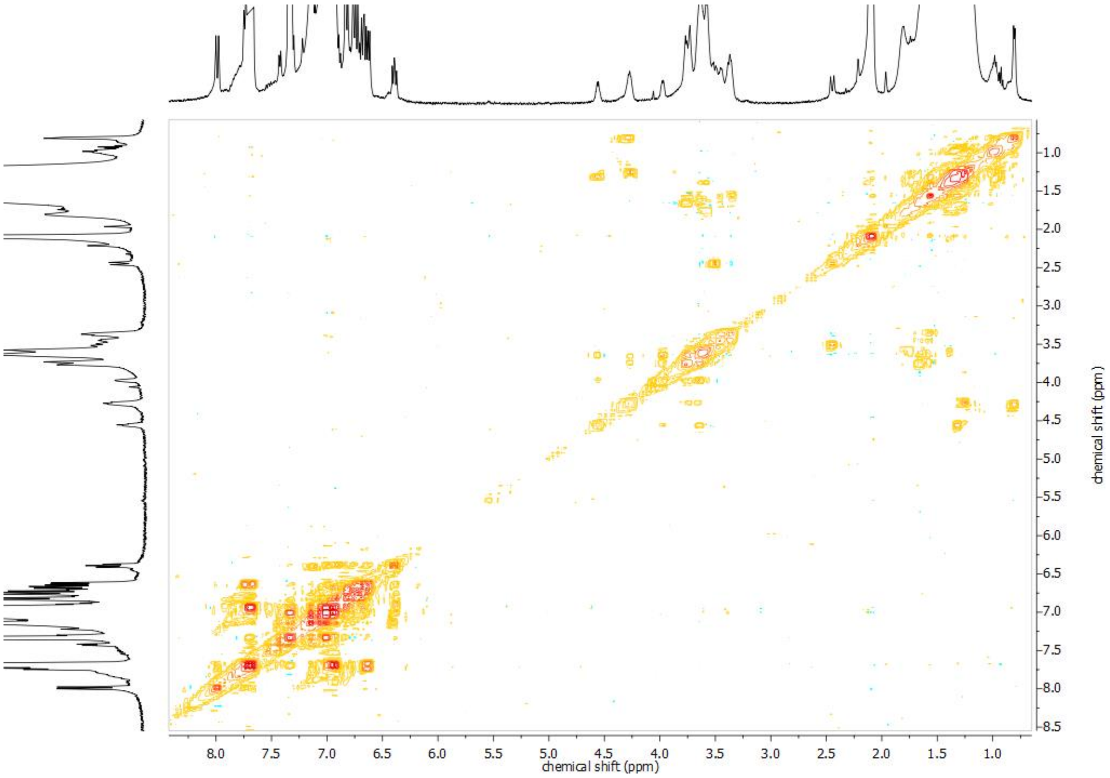

Supplementary Fig. 16 | ^{1}H, ^{1}H COSY NMR spectrum after illumination of (*S,R,R*)-±n. Toluene-*d*8, c = 1 mM, −40 °C, 500 MHz, λ_{irr} = 365 nm (18 min).

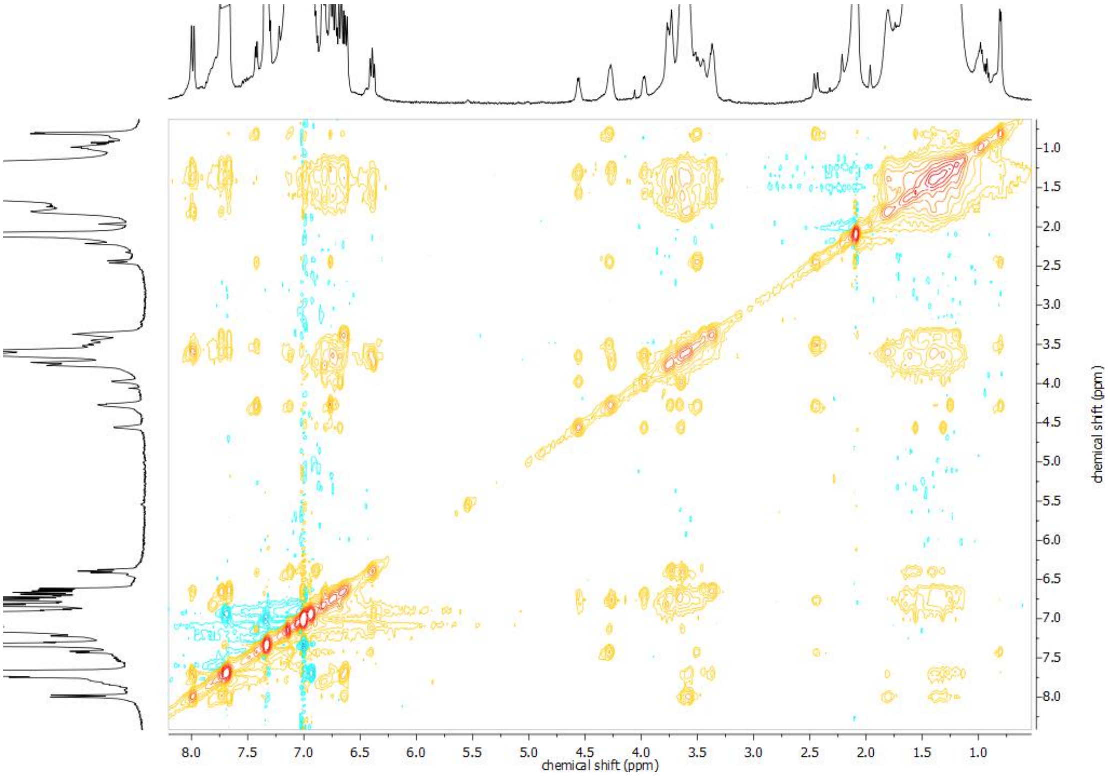

Supplementary Fig. 17 | ^{1}H, ^{1}H NOESY NMR spectrum after illumination of (*S,R,R*)-±n. Toluene-*d*8, c = 1 mM, −40 °C, 500 MHz, λ_{irr} = 365 nm (18 min).

NMR spectra of partially relaxed (*S*,*R*,*R*)-±n sample

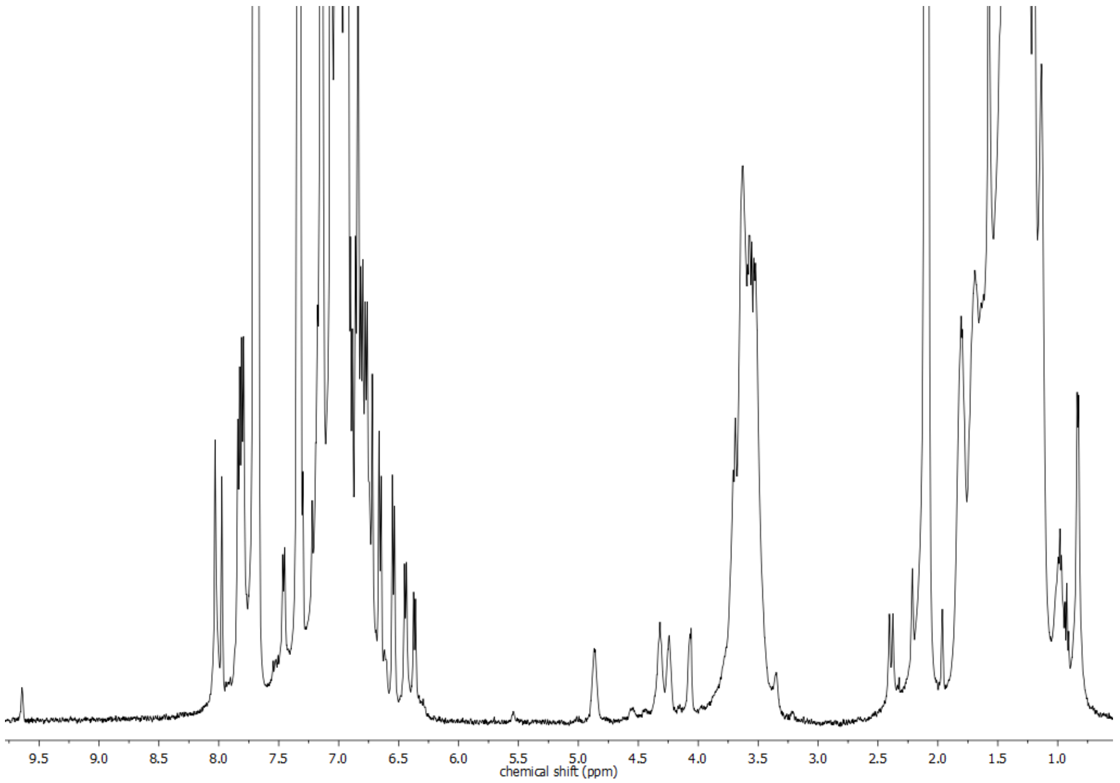

Supplementary Fig. 18 | ^{1}H-NMR spectrum of (*S*,*R*,*R*)-±n sample after 6 min at 60 °C. Toluene-*d*8, c = 1 mM, −40 °C, 500 MHz.

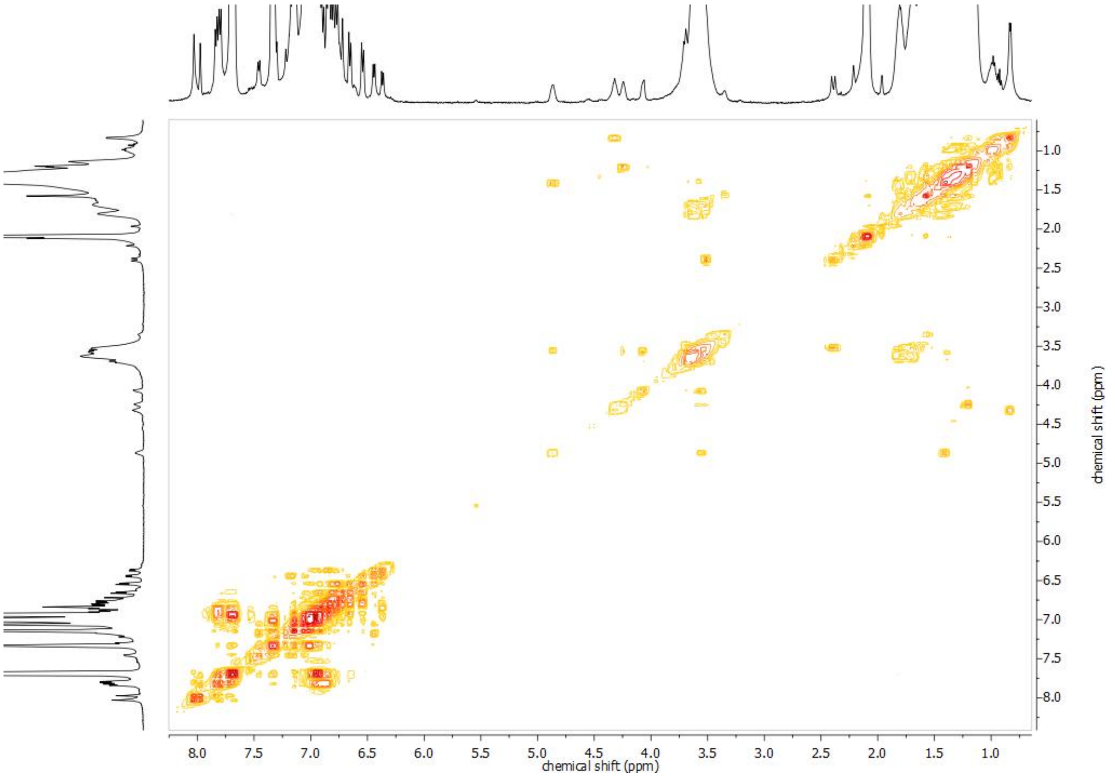

Supplementary Fig. 19 | ^{1}H,^{1}H COSY NMR spectrum of (*S*,*R*,*R*)-±n sample after 6 min at 60 °C. Toluene-*d*8, c = 1 mM, −40 °C, 500 MHz.

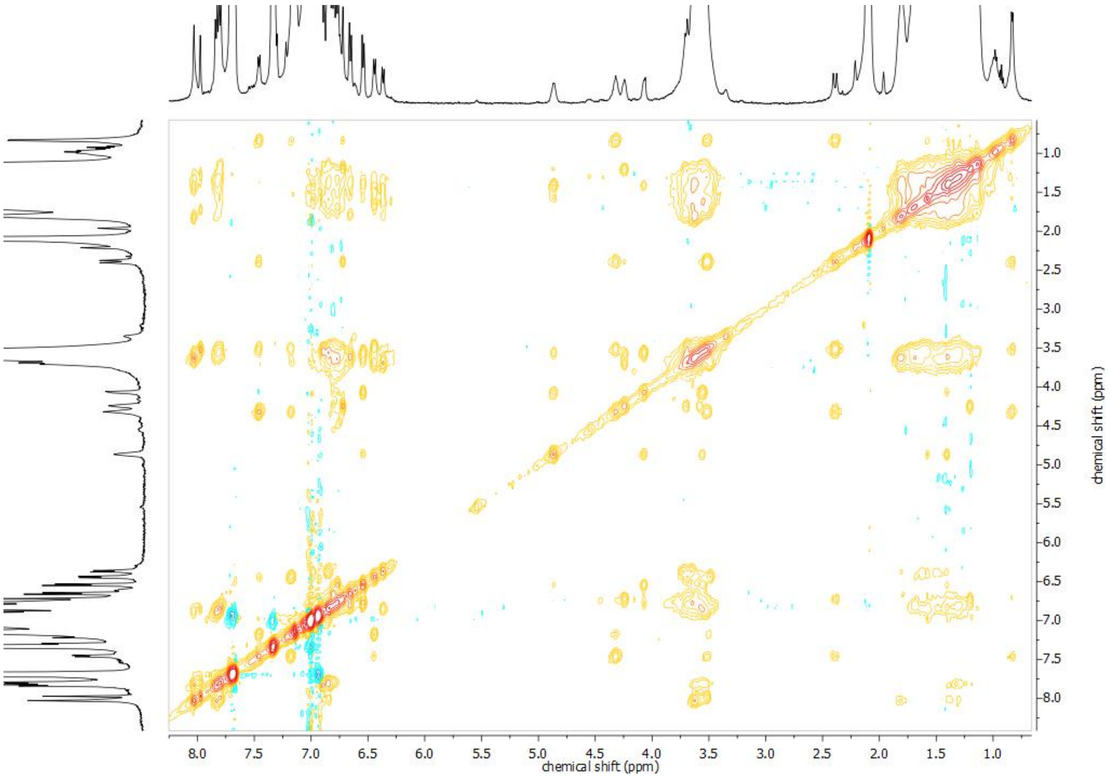

Supplementary Fig. 20 | ^{1}H, ^{1}H NOESY NMR spectrum of (*S,R,R*)-±n sample after 6 min at 60 °C. Toluene-*d*8, c = 1 mM, −40 °C, 500 MHz.

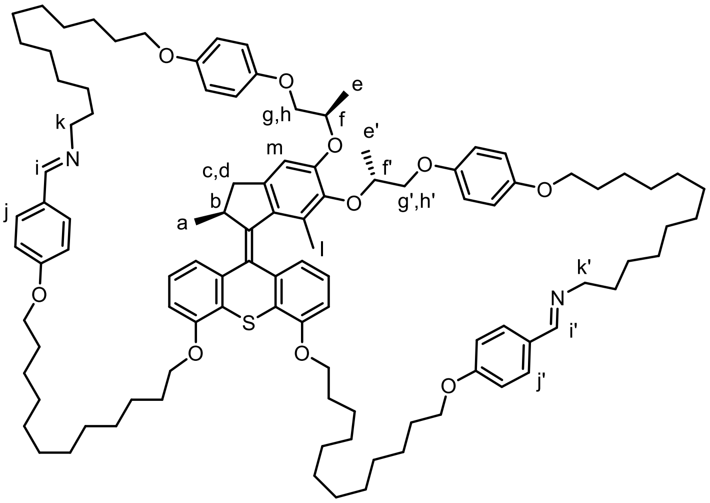

Supplementary Tab. 2 | Chemical shifts of important proton signal of (*S,R,R*)-±n. Toluene-*d*8, c = 1 mM, −40 °C, 500 MHz.

**Protons**	**Equilibrated**	**Illuminated**	**Partially relaxed**
**a**	0.76, 0.81, 0.86	0.80	0.83
**b**	4.32	4.30	4.32
**c,d**	2.36, 3.50	2,45, 3.50	2.39, 3.52
**e,e’**	1.35, 1.43	1.25, 1.31	1.21, 1.41
**f,f’**	4.46, 4.87, 4.93, 5.13	4.26, 4.55	4.24, 4.86
**g,g’,h,h’**	3.72, 3.78, 3.76, 4.13, 4.16	3.65, 3.74, 3.64, 3.97	3.59, 3.70, 3.56, 4.08
**i,i’**	8.02	7.98, 8.00	7.98, 8.03
**j,j’**	7.80	7.67, 7.74	7.80, 7.83
**k, k’**	3.47, 3.61	3.59	3.53, 3.62
**l**	1.56, 1.62, 1.66	1.55	1.58
**m**	6.71, 6.75	6.77	6.71

Light-driven winding

General illumination conditions

Typically, a J. Young NMR tube containing a 1 mM solution of equilibrated (*S,R,R*) or (*R,R,R*)-**±n** in 0.6 mL C_6D_6 was illuminated with a lamp for TLC control (365 nm, 6 W) on a shaker plate for 18 min at 8 °C (in a walkable fridge) or room temperature. The distance between lamp and NMR tube was kept constant throughout the experiment. To prevent any significant relaxation, illuminated samples were cooled with a 10 °C acetone bath before the respective measurement.

Stability towards oxygen

Under a nitrogen atmosphere, a sample can be irradiated and relaxed at elevated temperatures several times without noticeable fatigue. Note, that trace amounts of oxygen cannot be fully avoided, which over time has a noticeable impact on the highly dilute samples. First, residual PPh_3 acts as an oxygen scavenger and is oxidized to $OPPh_3$, which is then followed by photo-decomposition of (*S,R,R*) or (*R,R,R*)-**±n** upon irradiation with UV light of 365 nm. A similar behavior was observed for a comparable system by Giuseppone and co-workers [6].

Control experiments

To check if bis-macrocyclization of the motor is necessary for the winding mechanism, a 1 mM solution of bis-azide (*S,R,R*)-**S21** in C_6D_6 was illuminated for 18 min with UV light of 365 nm. No noticeable change in the ^{1}H-NMR spectrum was observed. Furthermore, we investigated the influence of different macrocyclization stages of (*S,R,R*)-**S21** on the winding process. Therefore, we illuminated (λ_{irr} = 365 nm, 18 min) a sample that was prepared according to the general procedure for imine macrocyclization after a reaction time of 6.5 h (7% aldehyde conversion) and 27 h (50% aldehyde conversion). This lead to the formation of (*S,R,R*)-**+3** in 0% and 10% yield, respectively (Supplementary Fig. 21).

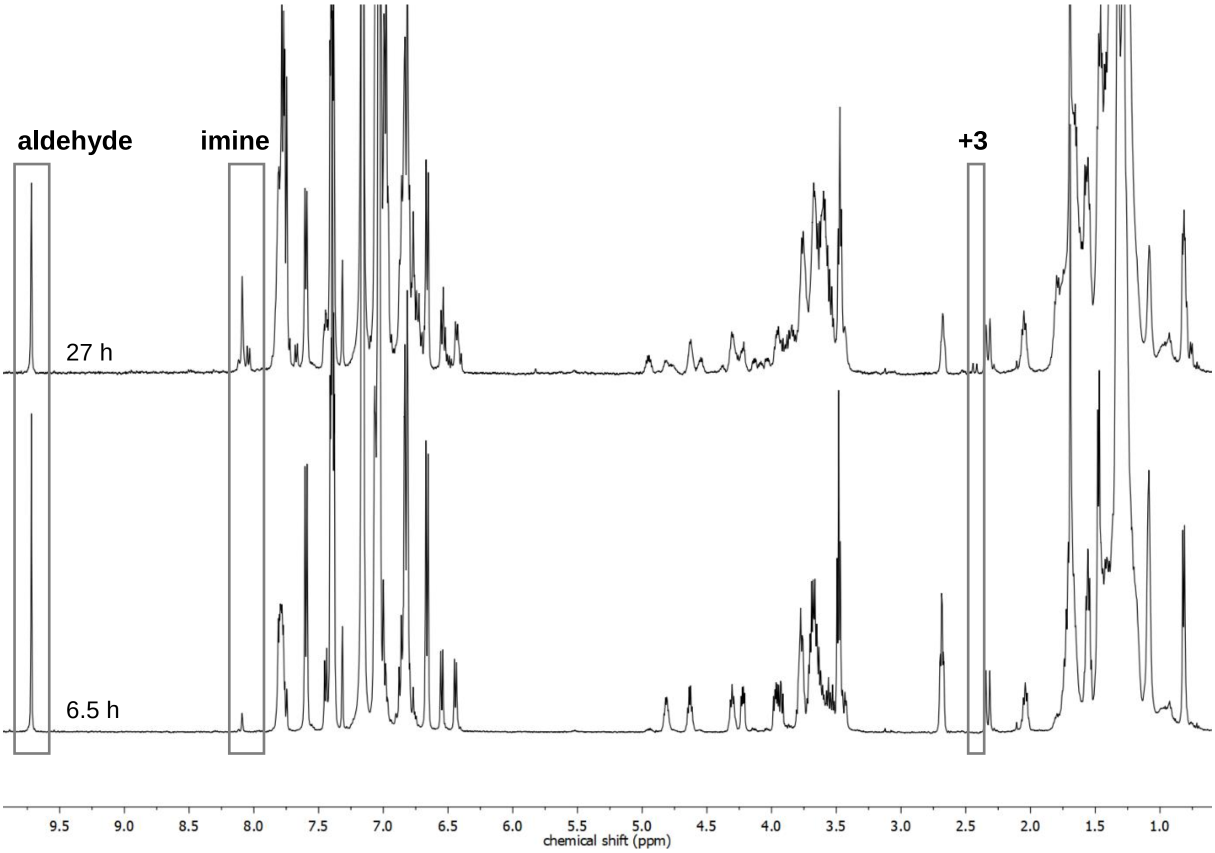

Supplementary Fig. 21 | ^{1}H-NMR spectra after illumination of partially macrocyclized bis-azide (*S,R,R*)-S21. After a reaction time of 6.5 h (7% aldehyde conversion, bottom) and 27 h (50% aldehyde conversion, top), (*S,R,R*)-**+3** forms in 0% (bottom) and 10% (top), respectively upon UV light illumination. C_6D_6, c = 1 mM, 10 °C, 500 MHz, λ_{irr} = 365 nm (18 min).

We also prepared the *n*-butyl bis-imine of compound (*S,R,R*)-**S21** in C_6D_6 (0.6 mL, 1 mM) by *in-situ* condensation with *n*-butyl imine (2 eq.). Almost quantitative conversion was observed after three d at 60 °C in presence of 3 Å molecular sieves. Also in this case, no change was observed after illumination with UV light of 365 nm for 18 min (Supplementary Fig. 22).

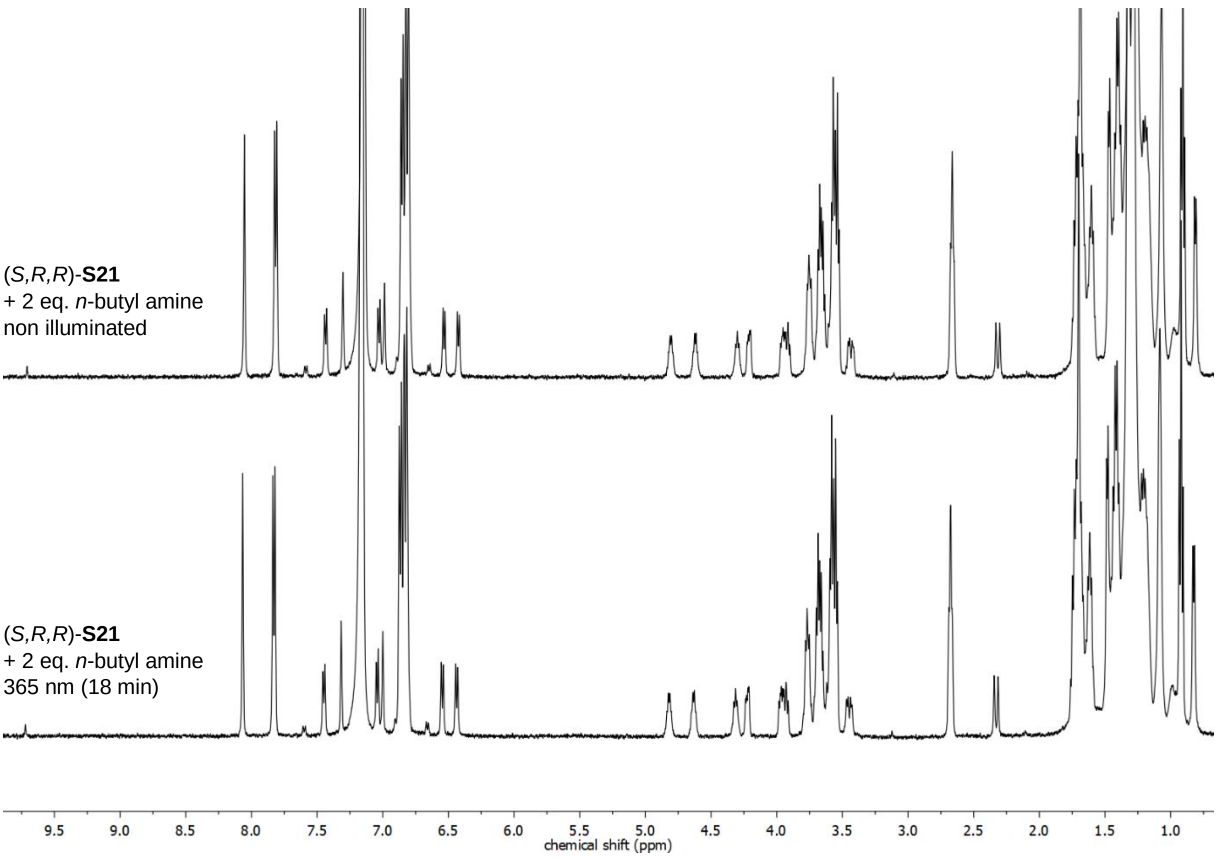

Supplementary Fig. 22 | ^{1}H-NMR spectra before and after illumination of (*S,R,R*)-S21 bis-*n*-butyl amine. No winding of the motor was observed. C_6D_6, c = 1 mM, 10 °C, 500 MHz, λ_{irr} = 365 nm (18 min).

CD Spectroscopy

In a cuvette, 30 µL of a solution of (*R,R,R*)- or (*S,R,R*)-**±n** (1 mM in C_6D_6) was diluted with degassed and dry THF to a total volume of 3 mL (c = 10 µM). Benzene absorbs in the deep UV region and forces a cut-off at 270 nm. Spectra of illuminated samples were either recorded after *in-situ* illumination with a TLC lamp at 365 nm for 5 min (the distance between the cuvette and the lamp was ~2 cm) or by diluting a pre-illuminated sample at PSS with THF. Partial relaxation of the irradiated sample was achieved by heating the sample to 60 °C for 10 min. Subsequent *in-situ* illumination showed that the process is reversible (Figs. 23 and24).

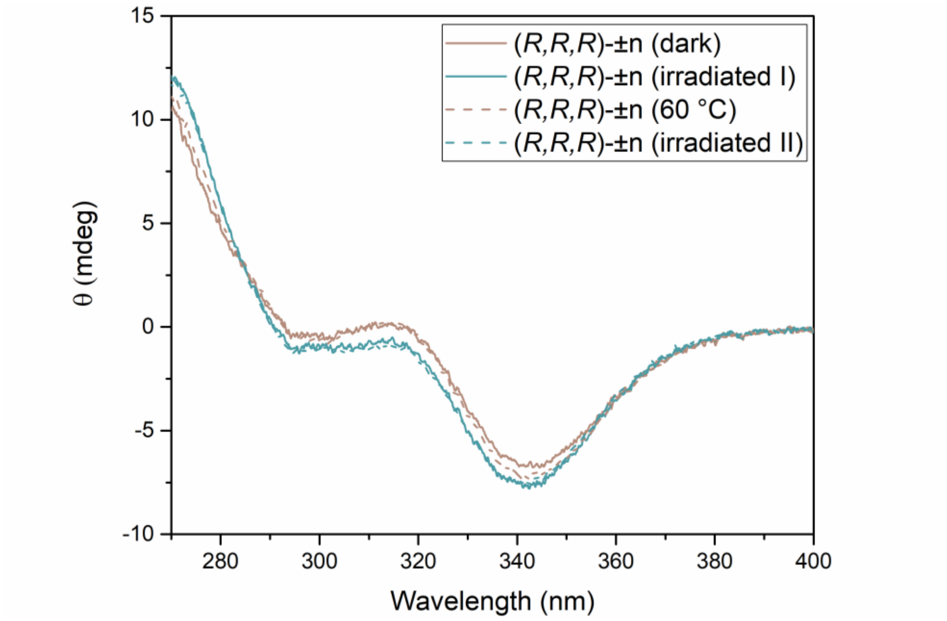

Supplementary Fig. 23 | CD spectra of (*R,R,R*)-±n. Fully relaxed (brown, solid line), illuminated (blue, solid line), relaxed at 60 °C for 10 min (brown, dotted line), and illuminated (blue, dotted line). THF, c = $1 \cdot 10^{-5}$ M, 0 °C, λ_{irr} = 365 nm (18 min).

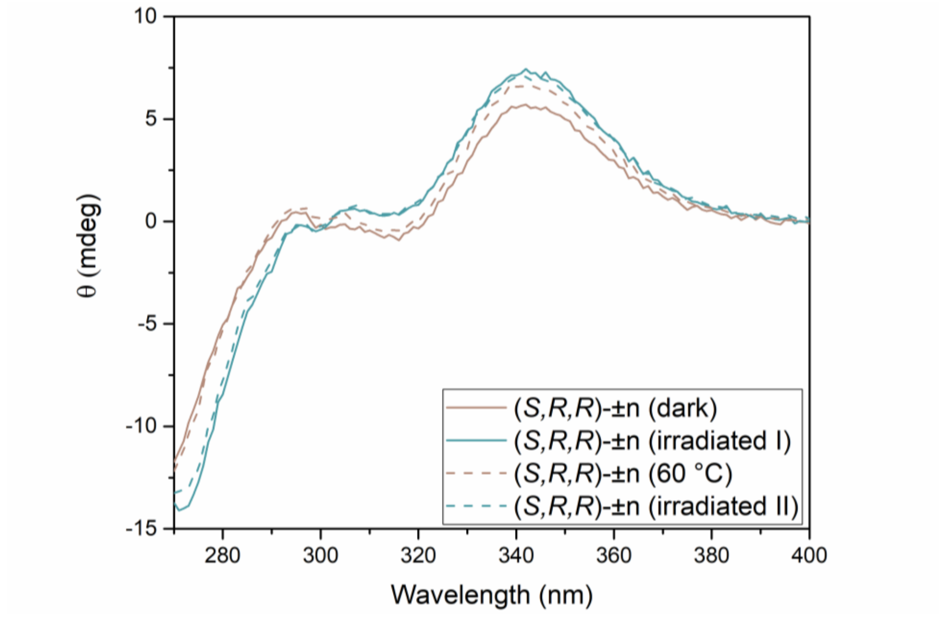

Supplementary Fig. 24 | CD spectra of (*S,R,R*)-±n. Fully relaxed (brown, solid line), illuminated (blue, solid line), relaxed at 60 °C for 10 min (brown, dotted line), and illuminated (blue, dotted line). THF, c = $1{\cdot}10^{-5}$ M, 0 °C, λ_{irr} = 365 nm (18 min).

CD spectra for fully protected motors (*R*,*R*,*R*)-**S16** and (*S*,*R*,*R*)-**S16** were recorded as reference spectra (Supplementary Fig. 25).

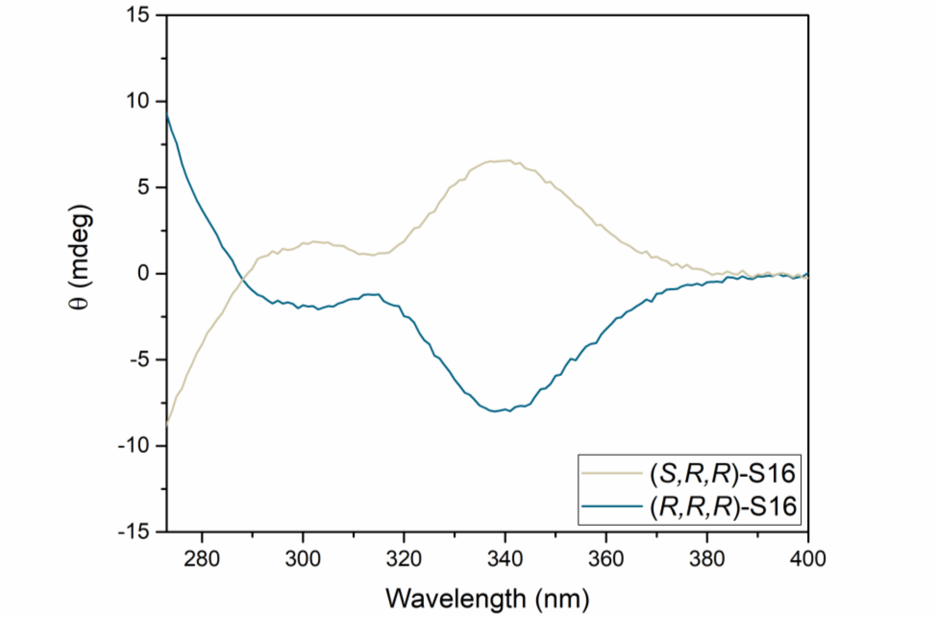

Supplementary Fig. 25 | CD spectra of (*R*,*R*,*R*)-S16 and (*S*,*R*,*R*)-S16. THF, c = 1 · 10^{-5} M, 0 °C.

NMR Experiments and Kinetic Analysis

General

All experiments were conducted on a Varian AVIII 500 NMR spectrometer that was pre-cooled or -warmed to the proper temperature. (*S,R,R*)-**±n** samples were typically equilibrated for 5 min inside the instrument until the lock signal reached a constant value. All samples were prepared according to the general procedure and were usually equilibrated at 60 °C. The experimental data were subsequently fitted using COPASI 4.29[7]. In order to obtain a fit that could give a realistic approximation of the irreversible and reversible reactions involved in each experiment, we simulated a reaction compartment of 0.6 mL (to match the volume of the solution of a typical NMR experiment) with concentration of the species involved of 1 mM. In all cases, the time unit used was minutes. The default Levenberg-Marquardt algorithm with a tolerance of $1 \cdot 10^{-6}$ implemented in COPASI was used. The initial guess for the kinetic parameter estimation was to consider all species in equilibrium with one another. After every fitting run, visual inspection of the error associated to each kinetic constant provided indication of the relevance of a certain reaction. Kinetic constants with absolute values lower than 10^{-6} min^{-1} were approximated to 0 and the respective reaction deleted in the next iteration. In the following section, the fitting of the experimental values, along with the kinetic model and the associated constants will be provided. For clarity, only a representative example of each dataset is presented. For all the output files of our kinetic simulations see Supplementary Data Set 1.

Relaxation kinetics of a (*S,R,R*)-±n sample at 10 °C without nucleophile.

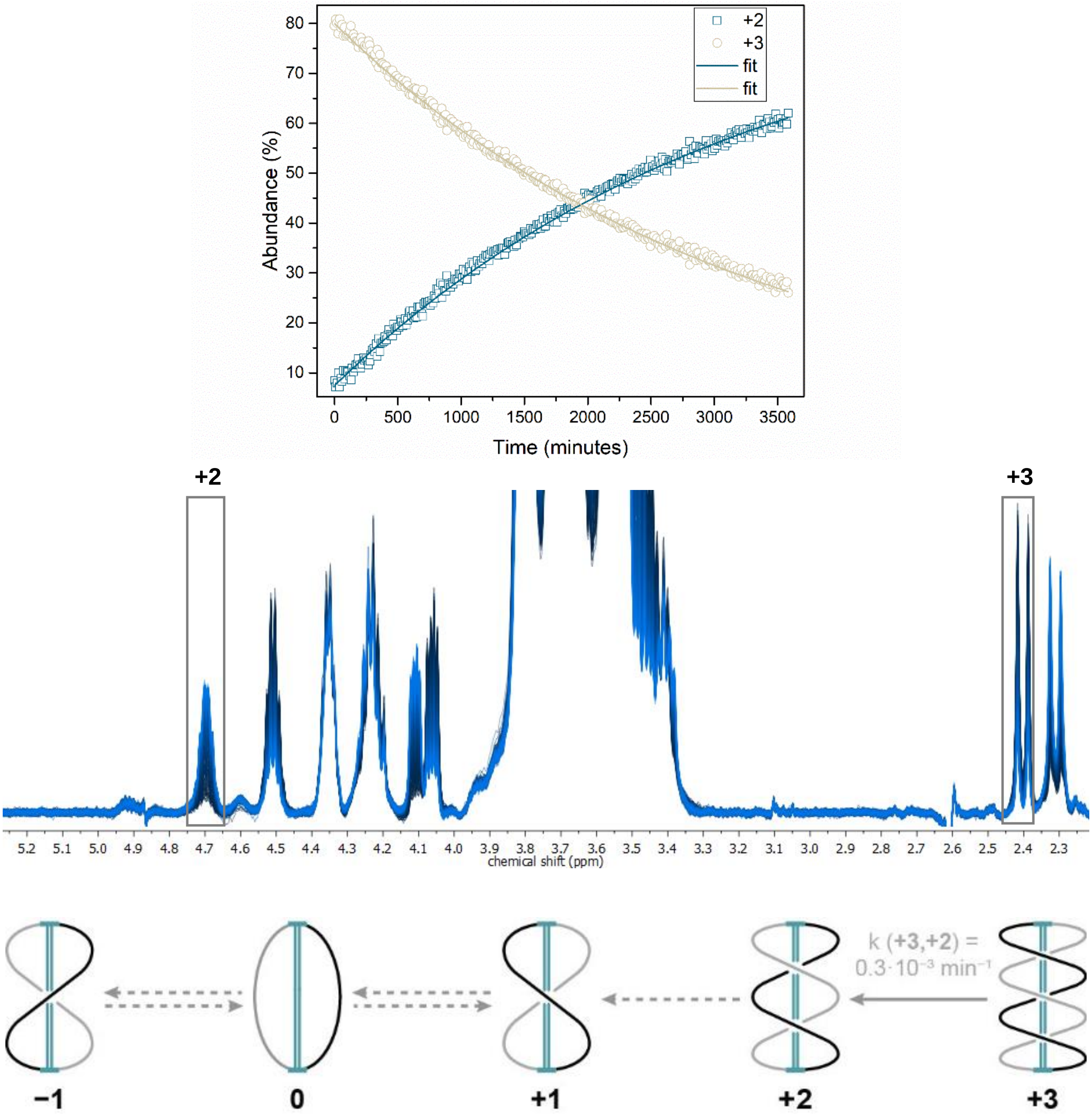

Supplementary Fig. 26 | Relaxation of a (*S,R,R*)-±n sample at 10 °C without nucleophile. Kinetic trace of a representative example with fit (top), NMR stack (middle) and proposed mechanism with average rate constants (bottom). C_6D_6, c = 1 mM, 500 MHz.

Relaxation kinetics of a (*S,R,R*)-±n sample at 10 °C with 20 mol% *n*-butyl amine

The amine (as a stock solution in deuterated benzene) was added inside a glovebox to a pre-illuminated (*S,R,R*)-**±n** sample (according to the general procedure). The same samples as for the relaxation experiment at 10 °C without nucleophile was used.

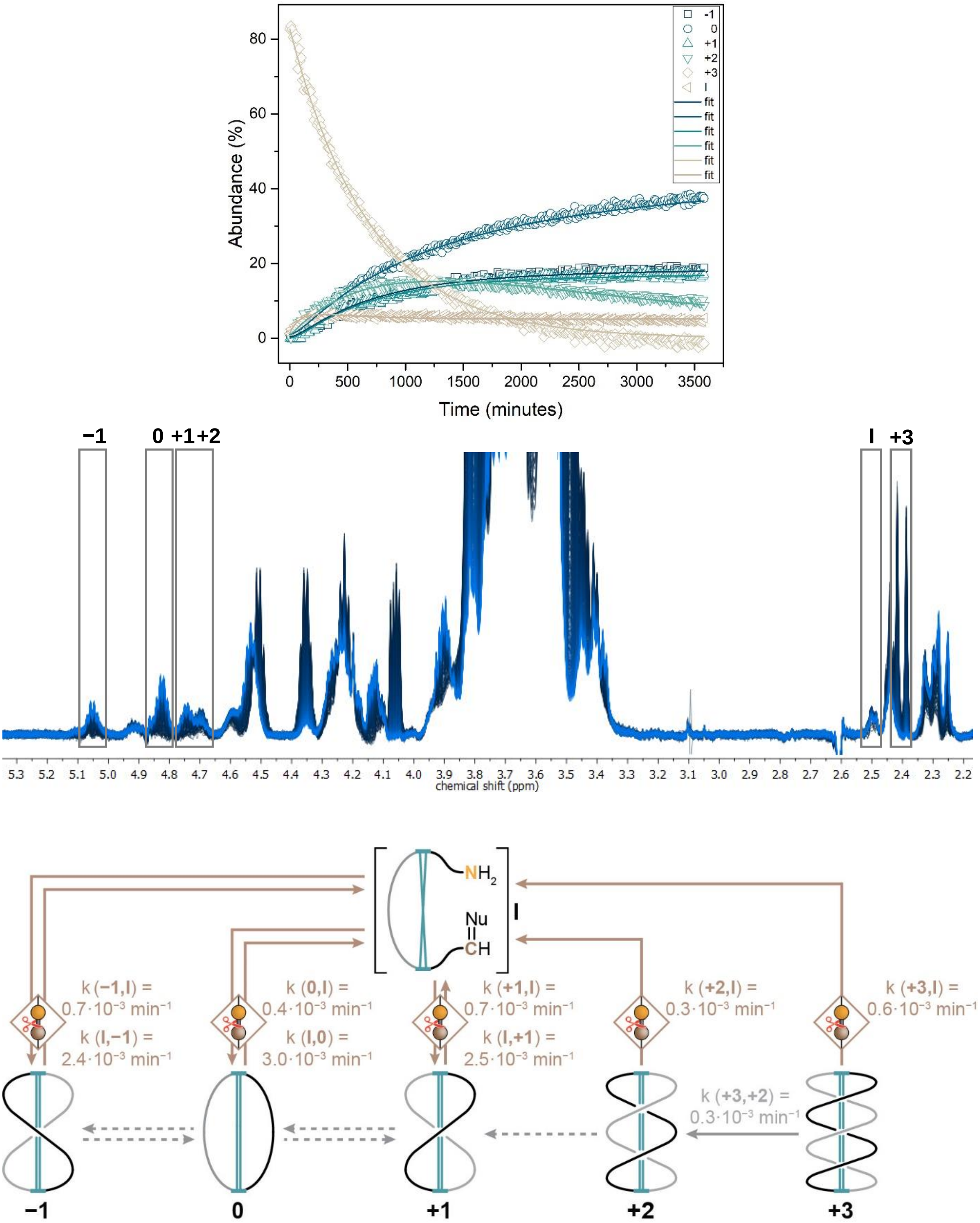

Supplementary Fig. 27 | Relaxation of a (*S,R,R*)-±n sample at 10 °C with 20 mol% *n*-butyl amine. Kinetic trace of a representative example with fit (top), NMR stack (middle) and proposed mechanism with average rate constants (bottom). C_6D_6, c = 1 mM, 500 MHz.

Relaxation kinetics of a (*S*,*R*,*R*)-±n sample at 60 °C without nucleophile

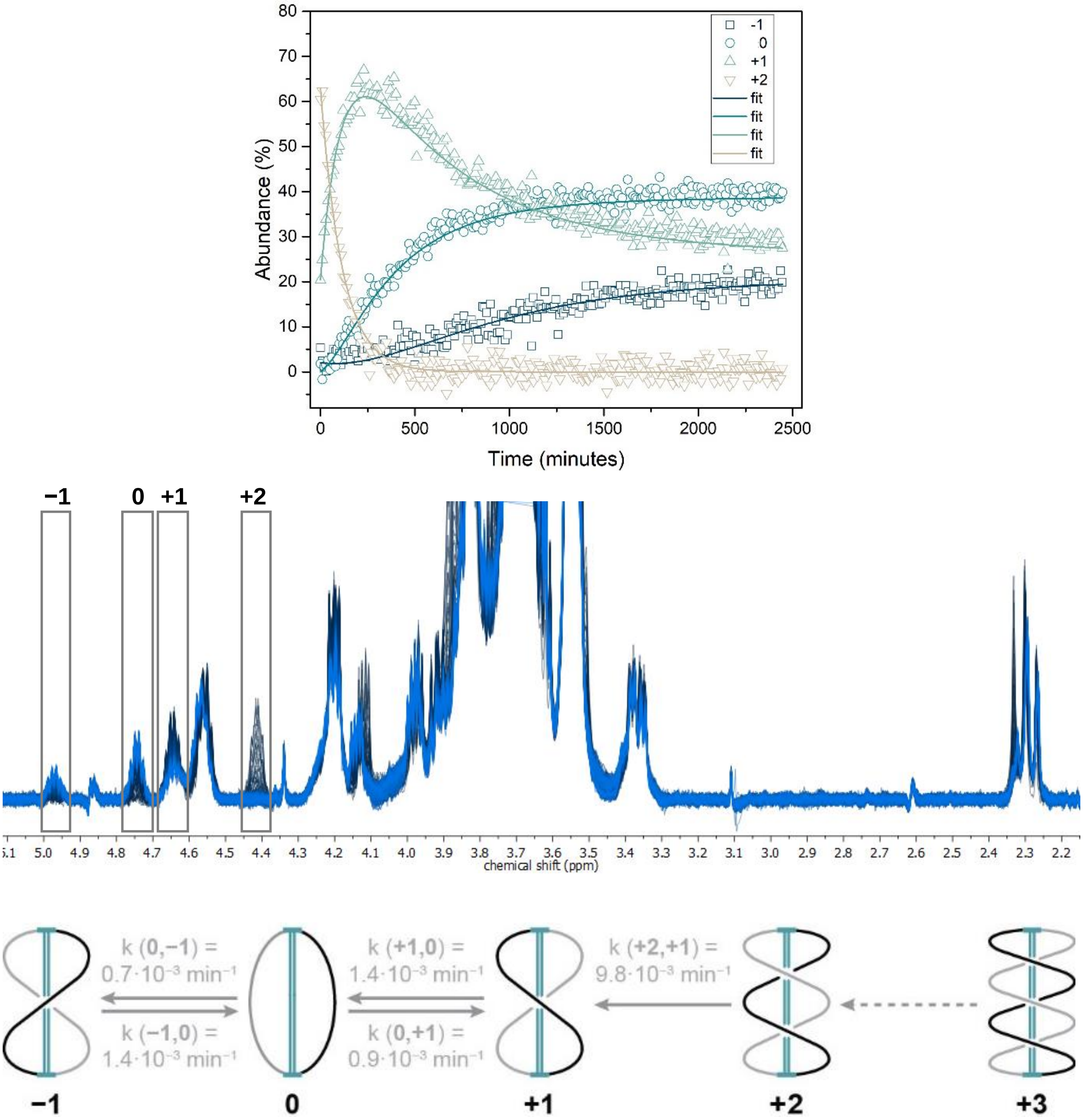

Supplementary Fig. 28 | Relaxation of a (*S*,*R*,*R*)-±n sample at 60 °C without nucleophile. Kinetic trace of a representative example with fit (top), NMR stack (middle) and proposed mechanism with average rate constants (bottom). C_6D_6, c = 1 mM, 500 MHz.

Relaxation kinetics of a (*S*,*R*,*R*)-±n sample at 40 °C without nucleophile

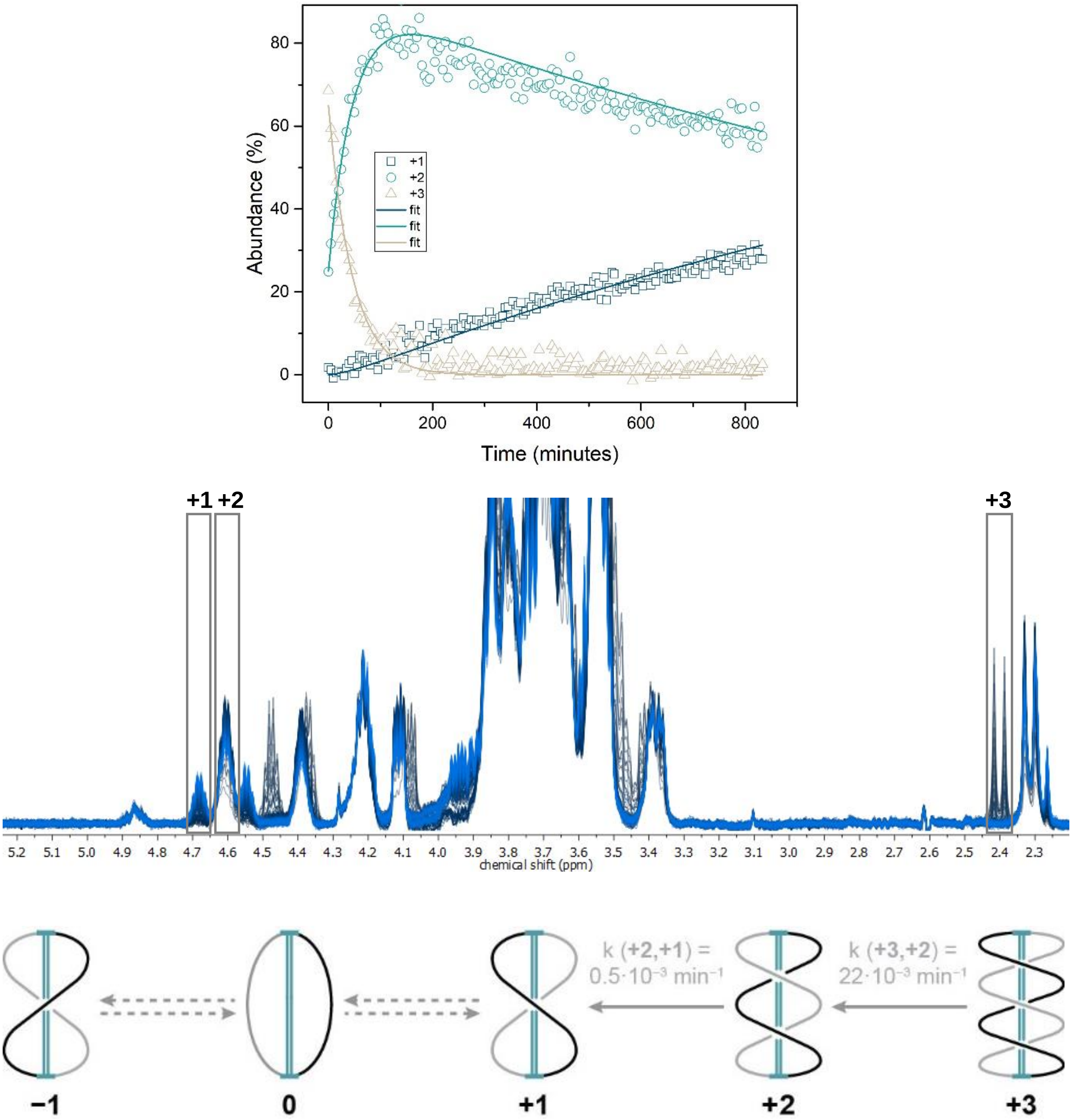

Supplementary Fig. 29 | Relaxation of a (*S*,*R*,*R*)-±n sample at 40 °C without nucleophile. Kinetic trace of a representative example with fit (top), NMR stack (middle) and proposed mechanism with average rate constants (bottom). C_6D_6, c = 1 mM, 500 MHz.

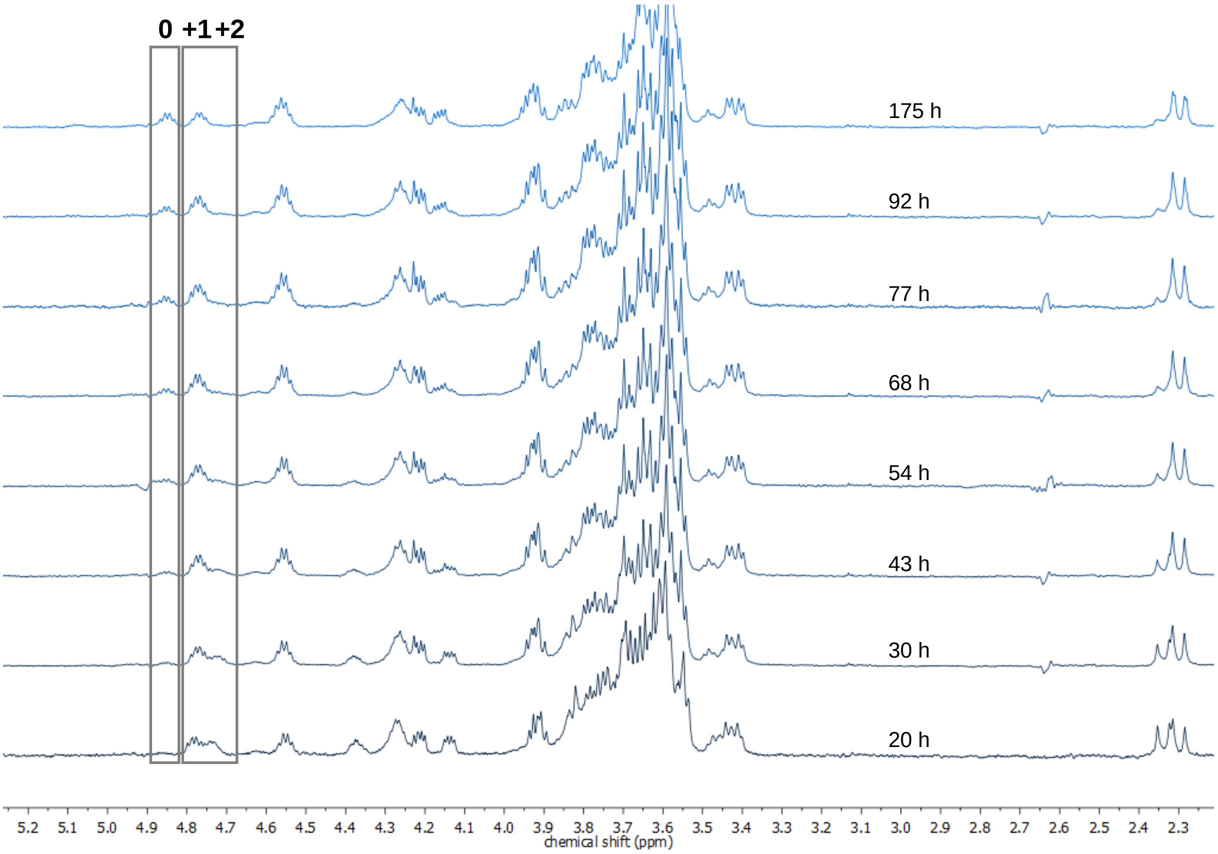

Supplementary Fig. 30 | ^{1}H-NMR spectra showing the relaxation of a (*S*,*R*,*R*)-±n sample at 40 °C without nucleophile over one week. C_6D_6, c = 1 mM, 500 MHz.

Relaxed (*S*,*R*,*R*)-±n sample at 10–60 °C without nucleophile and at 40 °C with 20 mol% *n*-butyl amine

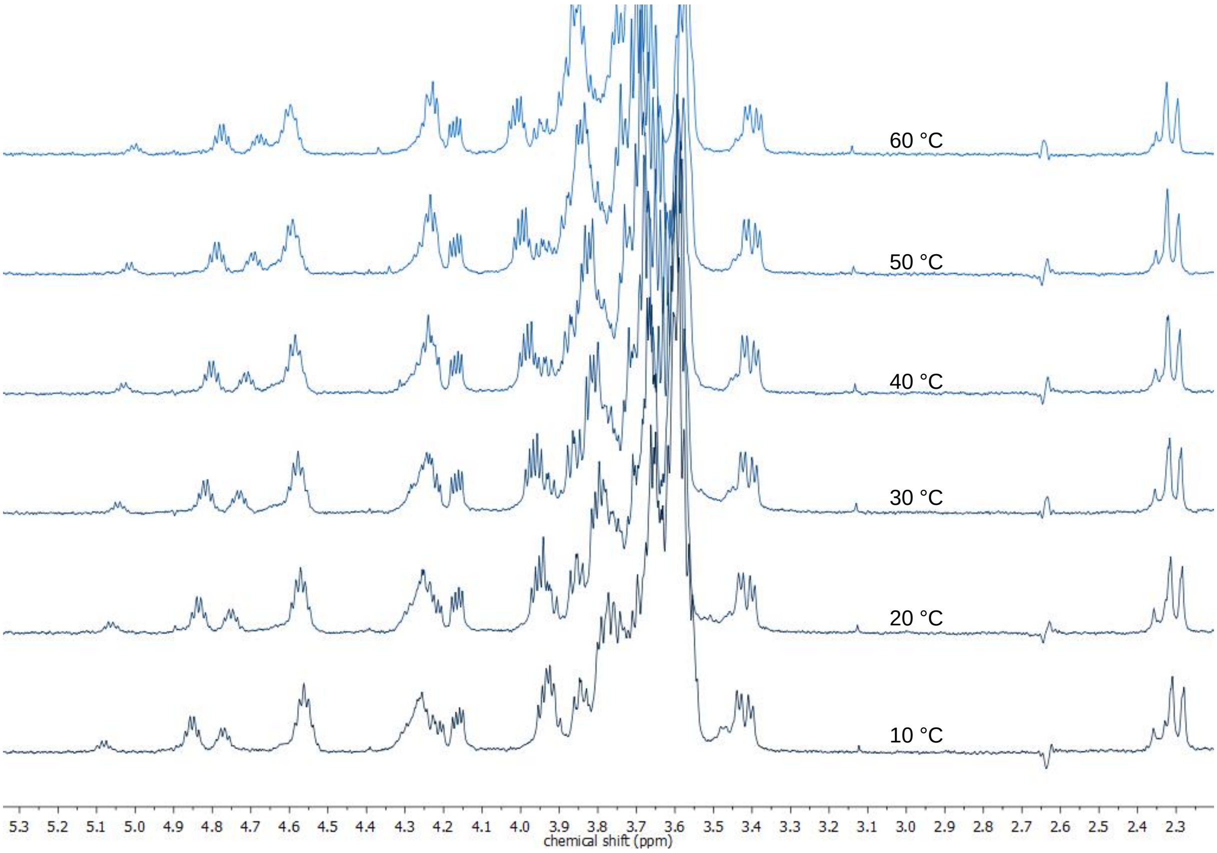

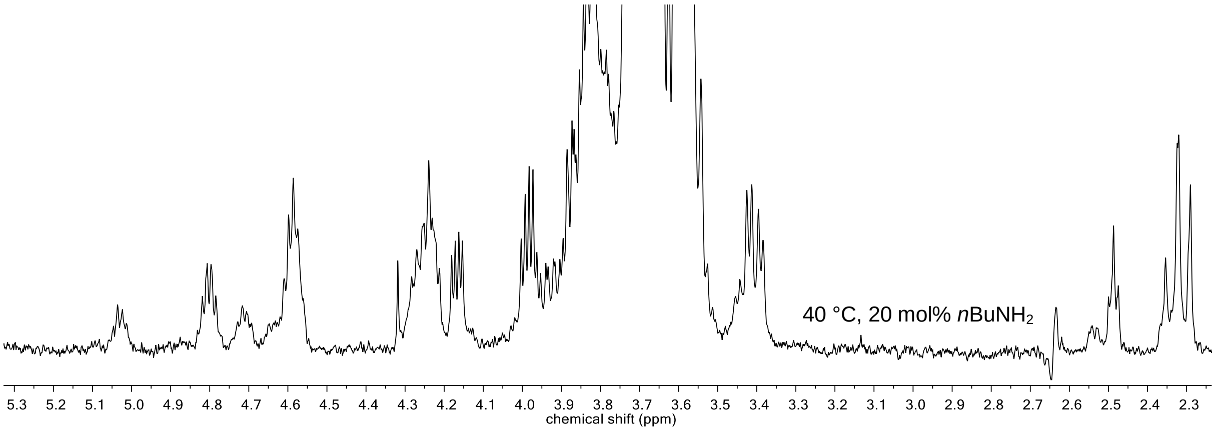

Supplementary Fig. 31 | ^{1}H-NMR spectra of an equilibrated (*S*,*R*,*R*)-±n samples. Without nucleophile at 10–60 °C (top) and with 20 mol% *n*-butyl amine at 40 °C (bottom). C_6D_6, c = 1 mM, 500 MHz.

Relaxation kinetics of a (*S,R,R*)-±n sample at 10–40 °C without nucleophile

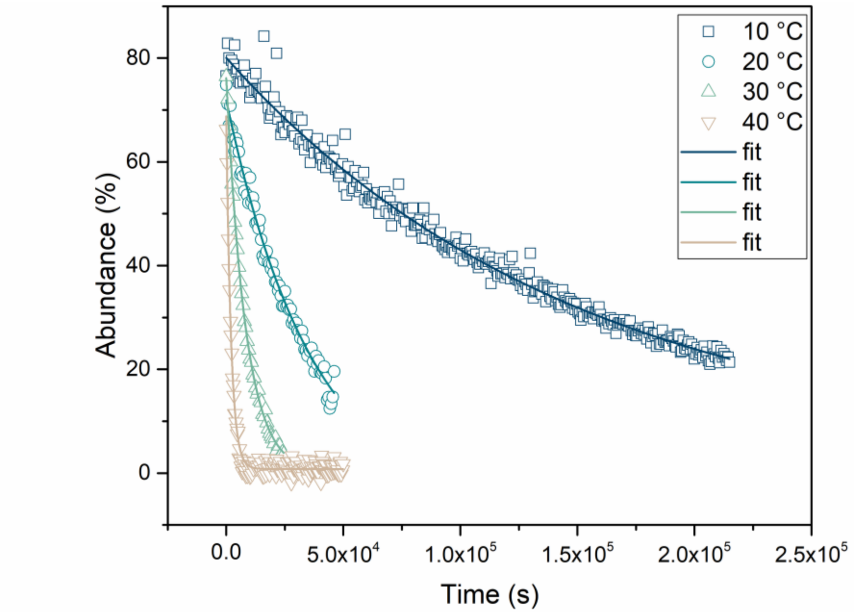

Supplementary Fig. 32 | Temperature dependent relaxation of (*S,R,R*)-+3 without nucleophile. The first order decay of (*S*,*R*,*R*)-**+3** accelerates by a factor of ~4 per 10 °C. C_6D_6, c = 1 mM, 500 MHz.

Relaxation of a (*S,R,R*)-±n sample in presence of water

Water (as a stock solution in deuterated THF) was added to a relaxed (*S,R,R*)-**±n** sample under an inert atmosphere. The same sample was used for all experiments. Water does not significantly increase the decay rate of (*S,R,R*)-**+3** (k (+3,+2) = 0.2–0.3·10^{-3} min^{-1}) at 10 °C. However, an illuminated sample (according to the general procedure) containing 20 eq. of water relaxed to isomers (*S,R,R*)-**−1**, **0**, **+1**, and **+2** at room temperature after 3 d. A similar sample without nucleophile forms almost exclusively (*S,R,R*)-**+2** under the same conditions.

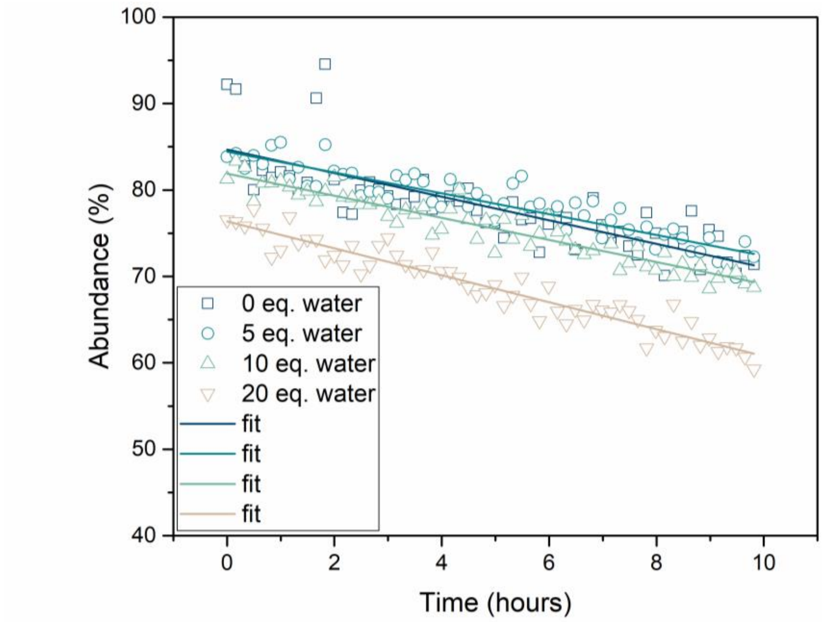

Supplementary Fig. 33 | Decay rate of (*S,R,R*)-+3 in presence of water. The amount of water has no significant effect on the decay rate of (*S,R,R*)-**+3**. The first order rate constant k (+3,+2) = 0.2–0.3·10^{-3} min^{-1} was determined by initial slope approximation. C_6D_6, c = 1 mM, 10 °C, 500 MHz.

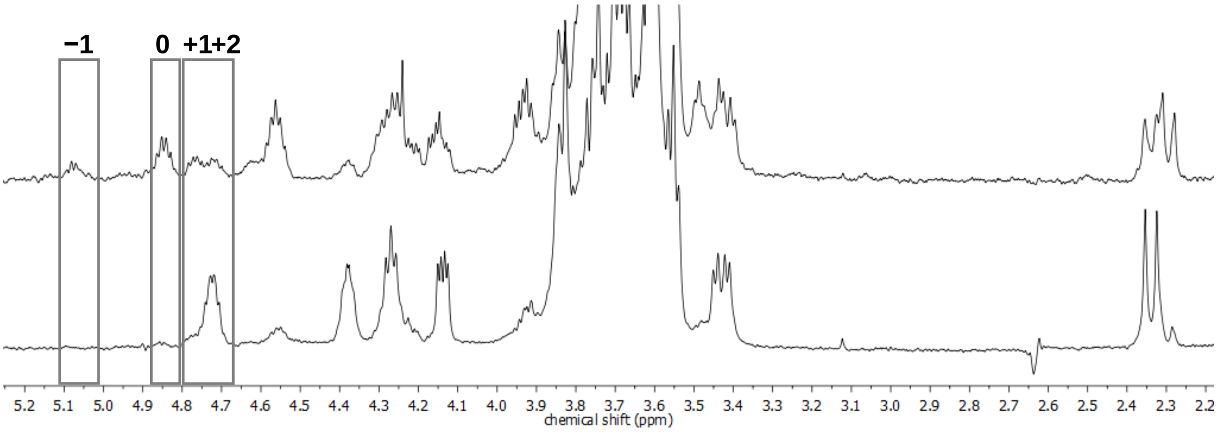

Supplementary Fig. 34 | ^{1}H-NMR spectra of irradiated (*S,R,R*)-±n samples at room temperature after 72 h with and without water. With 20 eq. water (top) and without external nucleophile (bottom). C_6D_6, c = 1 mM, 500 MHz.

Irradiation kinetics of a (*S*,*R*,*R*)-±n sample at 10 °C without nucleophile

A relaxed (*S*,*R*,*R*)-**±n** was illuminated at 8 °C inside a walkable fridge according to the general procedure. After each irradiation step, the sample was cooled to 10 °C with an acetone bath to prevent significant relaxation during transportation to the NMR instrument. Further experimental details for the quantum yield determination are given at page 56 of the Supplementary Information.

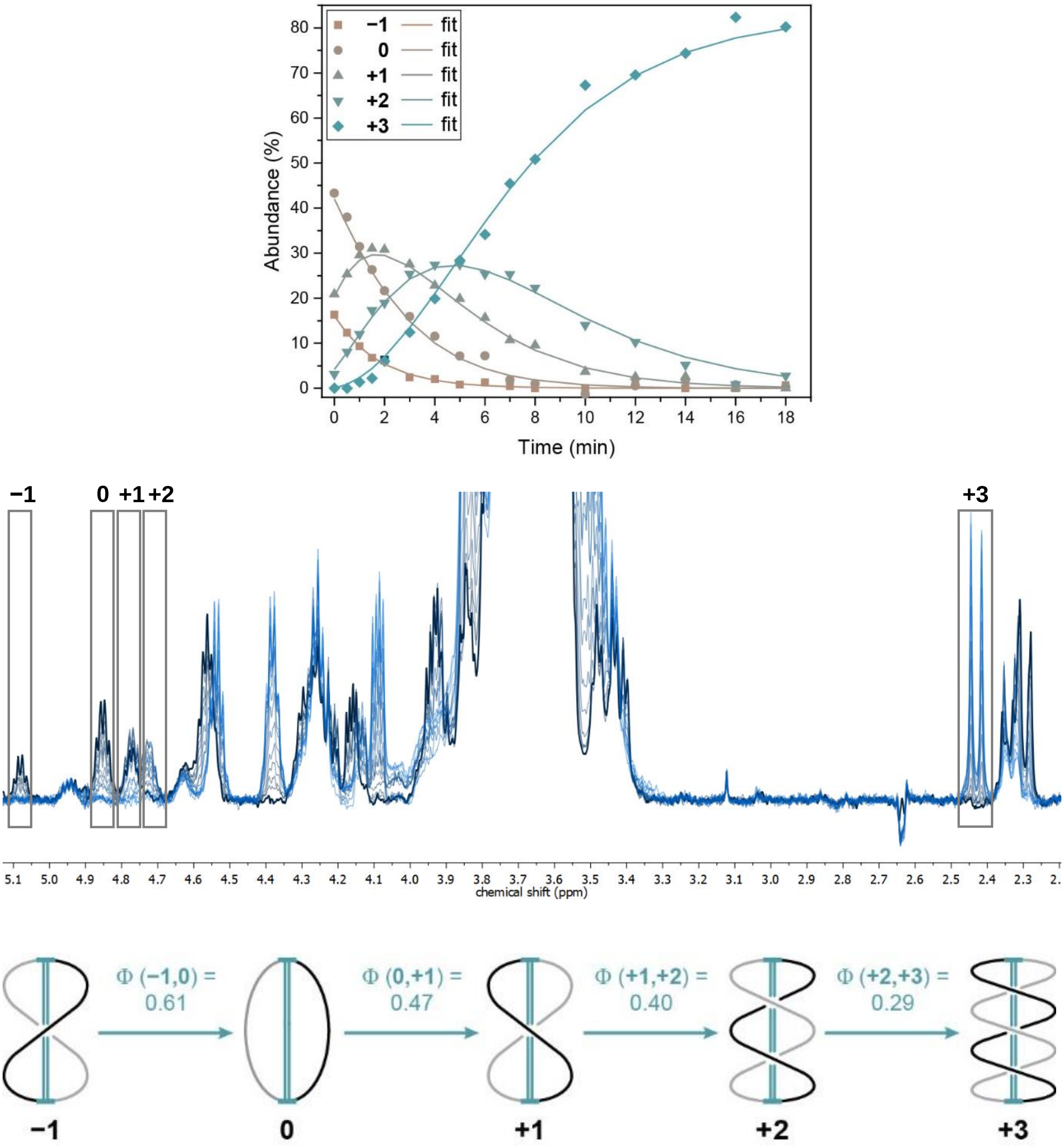

Supplementary Fig. 35 | Irradiation kinetics of a relaxed (*S*,*R*,*R*)-±n sample. Kinetic trace of a representative example with fit (top), NMR stack (middle) and proposed mechanism with average quantum yields (bottom). C_6D_6, c = 1 mM, 10 °C, 500 MHz.

Mass Spectrometry

Ion mobility (IM) measurements were performed using a custom drift-tube instrumentation hosted in the Fritz Haber Institute of the Max Planck Society (Berlin, Germany) and adapted from a previous design[8]. The instrument is designed around a nanoelectrospray ionization (nESI) source interfaced with a succession of radially-confining entrance funnel, drift tube and exit funnel. This ensemble is prolonged by a quadrupole mass analyzer under high vacuum and ended by an electron multiplier detector (ETP Ion Detect) for ion counting. In practice, samples were diluted to 10 μM in acetonitrile and nESI was used to generate ions using a needle voltage of 0.57 kV and a backing pressure of 0.8 bar (N_2). The ~160 cm long drift tube was filled with helium buffer gas at a pressure of 4 mbar and subjected to a 2 kV direct current (DC) electric field for mobility separation. Ions were filtered for *m/z* = 1612 Da, which correspond to the singly-protonated molecular ion $[M+H]^+$.

Experimental collision cross sections ($^{DT}CCS_{He}$) Ω were determined from the reduced mobility coefficient K_0 using Eq. 1[9] after measuring the arrival time distributions (ATD) for DC voltages ranging from 1.3 kV to 2.3 kV. The contribution of each peak was extracted by fitting the ATD using multiple Gaussian functions (OriginPro 2020, OriginLab).

$$K_0 = \frac{3}{16}\frac{q}{N_0}\left(\frac{2\pi}{\mu k_B T}\right)^{1/2}\frac{1}{\Omega}$$

where q is the ion charge, N_0 is the standard gas number density, μ is the reduced mass of the ion-gas colliding partners, k_B is the Boltzmann constant and T is the temperature.

Theoretical collision cross section (^{TM}CCS) were calculated using the trajectory method (TM) implemented in the MobCal software suite [10] as the average of 500 individual candidate structures randomly picked along the dynamics (Supplementary Data Set 2). The CCS values for all experimentally observed topological isomers of (*S,R,R*)-**±n** amount to: $^{TM}CCS_{He}$ (−1) = 344 Å^2, $^{TM}CCS_{He}$ (0) = 352 Å^2, $^{TM}CCS_{He}$ (+1) = 336 Å^2, $^{TM}CCS_{He}$ (+2) = 328 Å^2, $^{TM}CCS_{He}$ (+3) = 348 Å^2.

Supplementary Fig. 36a shows the evolution of the ATD for increasing durations of irradiation at λ_{irr} = 365 nm. The transition is characterized by a progressive narrowing of the distribution toward the emergence of a single peak associated with **+3** after 15 min. Supplementary Fig. 36b shows the evolution of the ATD for increasing injection voltages applied on the irradiated sample. The initial single peak corresponding to **+3** at low voltage is progressively extinguished in favor of three distinct contributions corresponding to **−1, 0** and **+1** at high voltages. This latter distribution agrees with the distribution of the non-irradiated sample, thereby validating the reversibility of the reaction pathway.

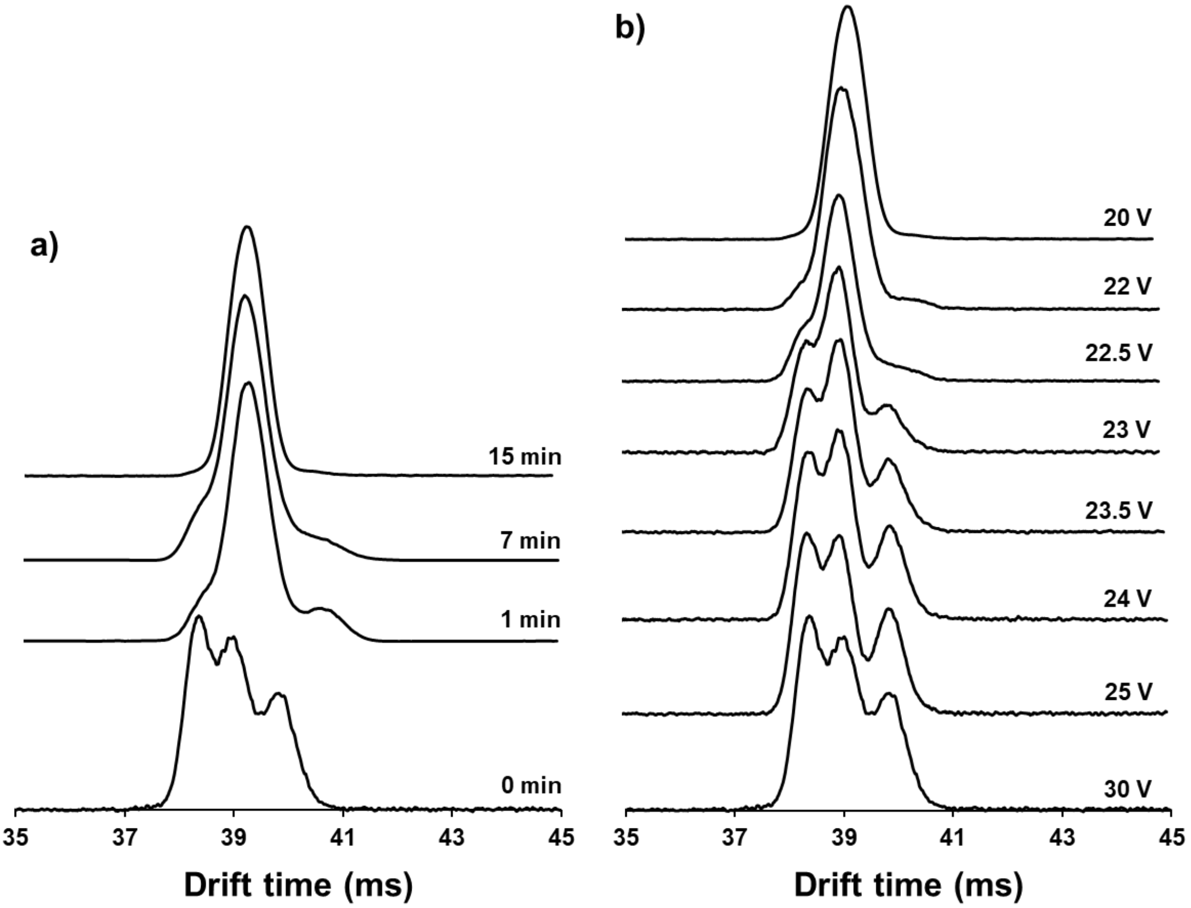

Supplementary Fig. 36 | ATDs of (*S,R,R*)-±n recorded on the drift tube instrumentation. a, ATDs for increasing durations of irradiation at λ_{irr} = 375 nm and **b,** increasing injection voltages from 20 V to 30 V applied on the irradiated sample.

Computational Analysis

The full thermal rotational path of the motor was probed at the ωB97X-D/def2-TZVP//ωB97X-D/def2-SVP level of theory as implemented in the Gaussian 16, Version B.01 software package[11]. The values of the Gibbs free energies (in kcal/mol) of each species are given in Supplementary Fig. 37. Two different pathways for the thermal helix inversion (THI) were considered (populating either intermediate **2** or **4**). To have a better overview of the unidirectionality of the motor, the thermal *E*-*Z* barrier (TEZ) was also computed using the broken-symmetry approach at the same level of theory used for calculating the thermal helix inversion step. A difference of more than 6 kcal/mol between the THI and TEZ barriers confirms the unidirectionality of the thermal step of the motor rotation.

The structures of the macrocyclic compounds in different topological isomers were modelled in the respective stable states of the motor core. All the structures were pre-screened using the CREST driver in the xTB software[12–14] using the GFN force field[15]. In this way, the most stable conformers for each structure were picked *via* the default series of metadynamics and dynamics runs implemented in the driver. The conformers obtained following this procedure were re-optimized at the GFN2-xTB level with very tight optimization criteria. The energy was then computed with a single-point calculation at the M06-2x/def2-SVP level, as implemented in the Gaussian 16, Version B.01 software package[11]. The energy profile of the isomerization process afforded a stepwise increase of the overall energy with a global minimum at the topological isomer **0**. The experimentally observed energy differences between the states amount to ΔG_{exp} (+3,+2) ≥ 2.0 kcal/mol, ΔG_{exp} (+2,+1) ≥ 2.0 kcal/mol, ΔG_{exp} (**+1**,**0**) = 0.24 kcal/mol and ΔG_{exp} (**−1**,**0**) = 0.39 kcal/mol by assuming that 3% of **±n** cannot be reliably detected by ^{1}H-NMR spectroscopy. These values are comparable with the computed electronic energy differences presented in Fig. 5b of the main text.

The CD spectra of (*S,R,R*)-**±n** with progressive number of turns was calculated at the sTDA-xTB level on the optimized structures. The results for the motor core with *S*-chirality are in Supplementary Fig. 38. The results furnish a qualitative flavor of the CD signs associated to metastable and stable states. From the computations, all the stable states show a positive Cotton effect in the most red-shifted band. The metastable states possess opposite helicity and consequently, opposite sign. Each metastable state was optimized following the same procedure previously discussed for the stable states. Every metastable state generated from a certain stable state was dubbed with an additional ".5" in the name (e.g. **+1.5** is generated by photochemical isomerization of **+1**).

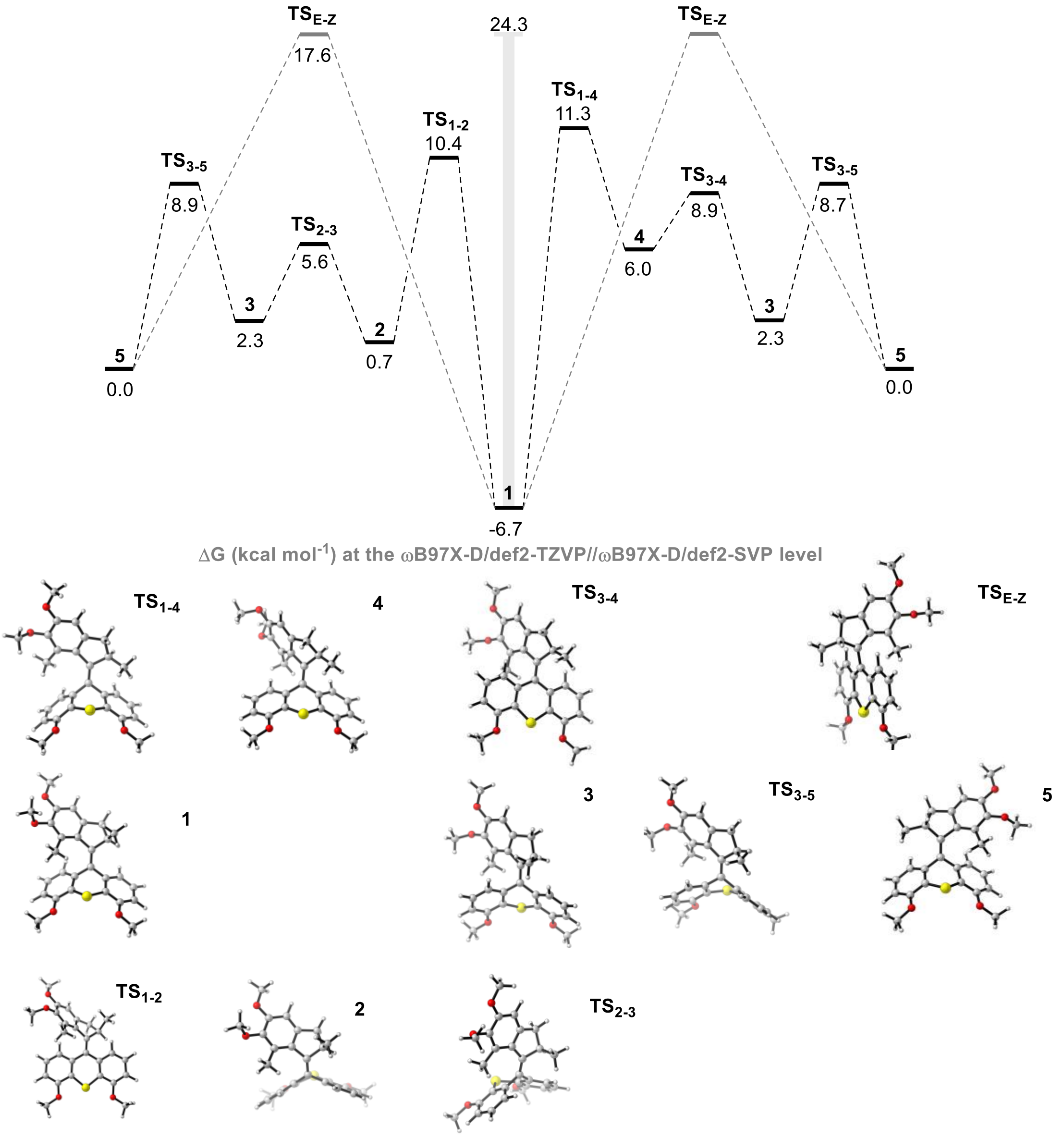

Supplementary Fig. 37 | Full thermal isomerization pathways of the motor core at the ωB97X-D/def2-TZVP//ωB97X-D/def2-SVP level.

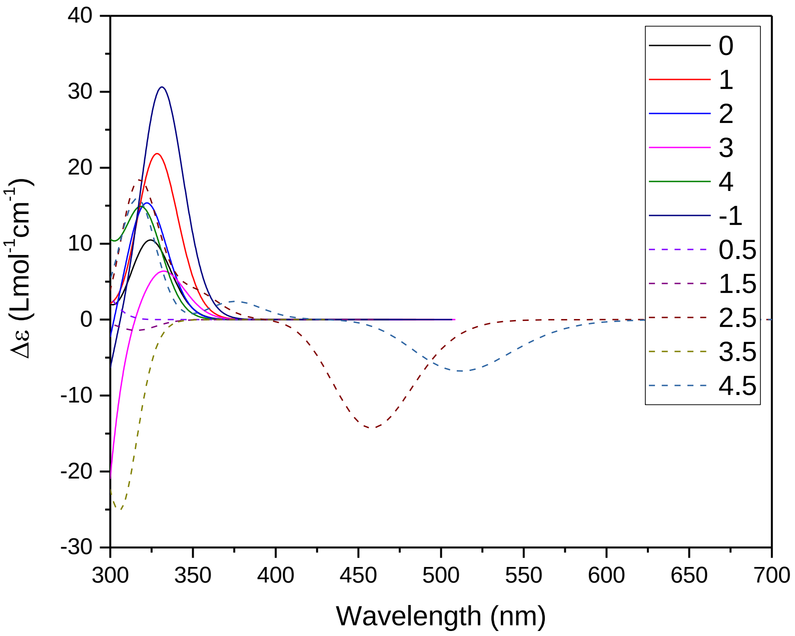

Supplementary Fig. 38 | Simulated CD spectra of the core of the different motors computed at the TDA(30 states)-ωB97X-D/def2-SVP level.

The dynamic behavior of the different topological isomer of the protonated macrocycle in vacuum were also computed, to provide a theoretical interpretation for the IM-MS results. The different stable isomers were optimized with the xTB software[12–14] using the GFN force field[15]. A molecular dynamics simulation of 1 ns was then run using the default values recommended from the developers (298.15 K, Berendsen thermostat, SHAKE algorithm for all bonds, hydrogen mass = 4, timestep of 2 fs). 500 geometries were then sampled randomly and used to calculate the collision cross-section values. Cartesian coordinates of all structures can be found in Supplementary Data Set 2.

SAXS Measurements

General

SAXS measurements were performed at the Multipurpose X-ray Instrument for Nanostructure Analysis (MINA) instrument at the University of Groningen. The instrument is built on a Cu rotating anode high brilliance X-ray source, providing X-ray photons with wavelength of λ = 0.154 nm. The SAXS patterns were recorded using a 2D Vantec500 detector placed 24 cm away from the sample. SAXS 1D profiles were obtained by radially averaging the scattered intensity around the origin of the image (defined by the direct beam position on the detector) using MATLAB. Standard corrections for the detector distortion and sensitivity were applied. The scattering from the buffer solution was subtracted to obtain the neat SAXS signal of the sample. The 1D SAXS profiles are plotted against the modulus of the scattering vector defined as $q = 4\pi \sin\theta/\lambda$, where θ is half of the scattering angle. The probed scattering angle range was calibrated using known position of diffraction peaks from a standard Silver Behenate sample (NIST).

Sample preparation

A relaxed (*S,R,R*)-**±n** sample (1 mM solution in toluene-*d*8) was contained in a glass capillary of 1.5 mm diameter (wall size of 0.01 mm), flame-sealed to avoid solvent evaporation and placed in the X-ray vacuum chamber to remove air absorption and scattering. The capillary temperature was stabilized at 23 °C. After the measurement, the same solution was illuminated inside the capillary with UV light (λ_{irr} = 365 nm, 18 min) and immediately measured. To probe reversible conformation change of the molecule, the sample was allowed to relax at 60 °C for 16 h and then measured. We also measured a sample that was pre-illuminated according to the general procedure, which lead to similar results.

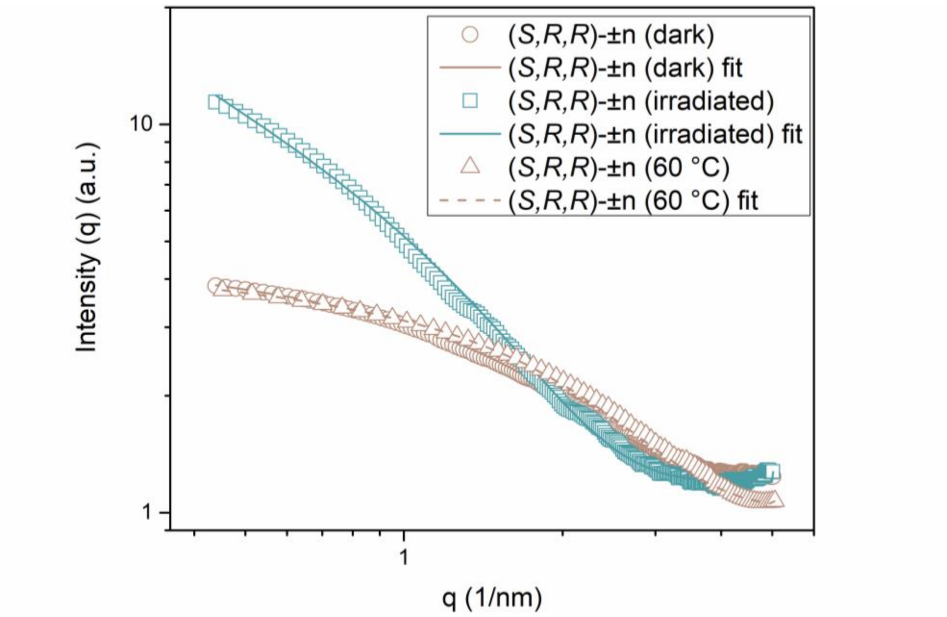

Supplementary Fig. 39 | Scattering intensities of a (*S,R,R*)-±n sample. At equilibrium (brown circles), after illumination with 365 nm for 18 min (blue squares), and after relaxation at 60 °C for 16 h (brown triangles). 23 °C, c = 1 mM, toluene-*d*$_8$.

Data analysis

As the shape of the nanoratchet in solution is not the one of a simple object (sphere, cylinder, *etc.*), SAXS profiles were further analyzed to estimate the dimensions of the nanoobject in solution using a model-independent approach. In this case, a generalized Guinier equation was used.

$$I(q) = \begin{Bmatrix} 1 & \text{for } \alpha = 0 \\ \alpha\pi q^{-\alpha} & \text{for } \alpha \neq 0 \end{Bmatrix} A \exp\left(\frac{R_\alpha^2 q^2}{3-\alpha}\right)$$

where α is the shape factor (0 for sphere, 1 for rod, and 3 for disk), R_α is the radius of gyration and A is a scaling pre-factor that depends on quantities specific of the sample (volume, contrast, and concentration) and specific of the experimental configuration (photon flux, detector sensitivity, and solid angle defined by the detector). The advantage of this approach is that no a-priori assumption is made on the shape of the nanoobject in solution, that can be inferred by the fitted value of α.

The best fitting curves obtained by this method are in Fig. 2 of the main manuscript and the fitting results are summarized in Supplementary Tab. 3. Data fitting was performed using analytical models *via* the SASFIT program[16].

Supplementary Tab. 3 | SAXS profile fitting results.

	A	α	R_α (nm)
**Equilibrated**	3.9	0.19	0.86
**Illuminated**	2.1	0.85	0.88
**60 °C (16 h)**	3.8	0.15	0.79

Our SAXS analysis clearly suggests that the pristine molecules in solution adopt a close-to-spheroidal configuration as α = 0.19 is close to 0 which is expected for a perfect sphere. On the contrary, winding causes a clear shape change towards an elongated-like conformation as α = 0.85 is close to 1 which is expected for a perfect cylinder. In this case, an estimation of the cross-sectional radius of gyration for a rod-like conformation (or of the short semi-axis for an ellipsoidal conformation) can be obtained by the cross-sectional Guinier analysis[17], *i.e.* fit of the linear part in the plot $\log(qI(q))$ vs q^2 in the range of points that satisfy the relationship $qR_c < 1$. The value of the slope is related to the cross-sectional radius as $slope = R_c^2/2$. For the irradiated sample (see Supplementary Fig. 40), we get an estimated $R_c \sim 0.7$ nm.

The larger dimension of the elongated nanoobject can be estimated assuming either an ellipsoidal shape ($R_\alpha^2 = \frac{a^2}{5} + \frac{2b^2}{5}$, with a > b being the semi-axes) or a cylindrical shape ($R_\alpha^2 = \frac{R_c^2}{2} + \frac{L^2}{12}$). We thus estimate $a \sim 1.7$ nm and $L \sim 2.5$ nm. These values should be considered with care as they are based on the assumption of a well-defined geometrical shape, but can be nevertheless considered as the lower and an upper limits of the larger axis of the wound nanoobject in solution.

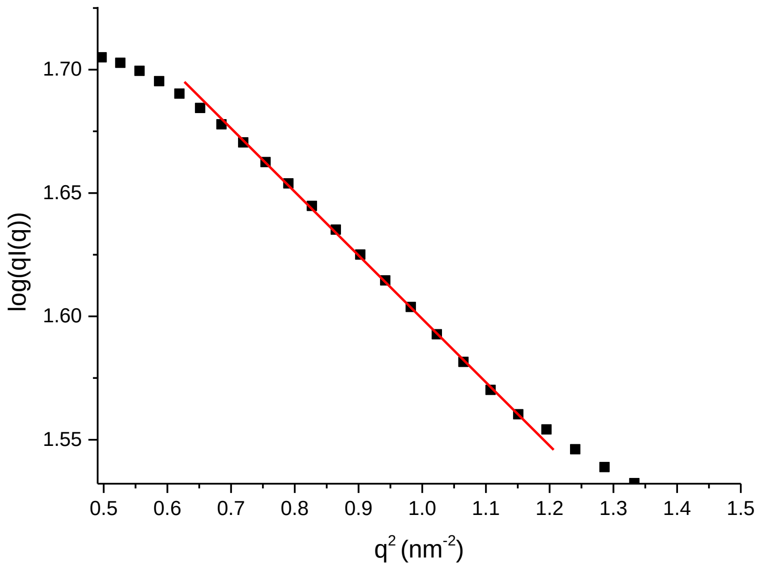

Supplementary Fig. 40 | Guinier plot for the irradiated sample. Red line is the linear fit that gives a slope of −0.25.

Quantum Yield Determination

The quantum yields for every single winding step of molecular machine (*S,R,R*)-**±n** were determined by NMR spectroscopy with *ortho*-nitrobenzaldehyde (**NBA**) as an actinometer, following a literature procedure from Ji *et al.*[18] The following equation was used for the fit:

$$-\frac{d[\mathbf{NBA}]}{dt} = \Phi_{\boldsymbol{NBA}} I_0 (1 - 10^{-\varepsilon_{\boldsymbol{NBA}} b[\mathbf{NBA}]})$$

The molar extinction coefficient of NBA is ε_{NBA} (365nm) = 265 M^{-1} cm^{-1} and its quantum yield is Φ_{NBA} = 0.5[18]. The light intensity in our irradiation setup was measured at 365 nm and corresponds to a molar photon flux I_0 (365nm) = 4.252 mM min^{-1}; the path length corresponds to b = 0.027 cm (Supplementary Fig. 41 left).

The molar extinction coefficient ε was determined for the motor core (*S,R,R*)-**S16** (Supplementary Fig. 41 right) and corresponds to ε (365nm) = 3891 M^{-1} cm^{-1}. Based on UV/vis and CD spectroscopic experiments, we can assume that ε (365nm) is similar for all topological isomers.

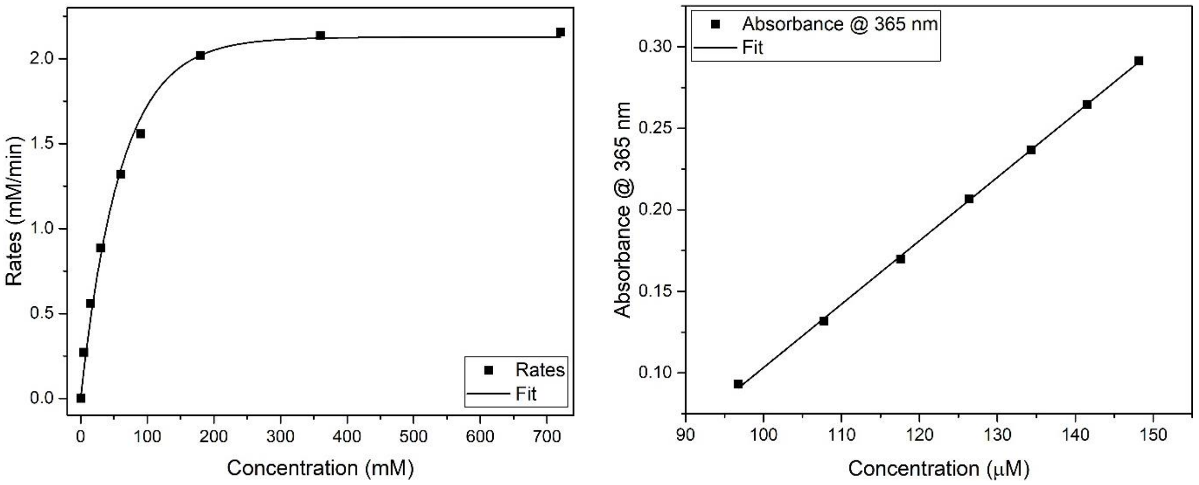

Supplementary Fig. 41 | Determination of the light intensitiy (I_0), path length (b) and molar extinction coefficient (ε) at 365 nm. The photochemical conversion rates of *ortho*-nitrobenzaldehyde to *ortho*-nitrosobenzoic acid at various concentrations give I_0 and b (left), whereas ε was determined by measuring the absorbance of motor core (*S*,*R*,*R*)-**S16** at different concentrations (right).

The quantum yield Φ was then determined by following the temporal change of a fully relaxed sample of (*S,R,R*)-**±n** upon illumination with 365 nm by NMR spectroscopy (Supplementary Fig. 35). The kinetic profile was fitted with COPASI 4.29[7] by applying the following equation:

$$-\frac{d[\pm\mathbf{n}]}{dt} = \Phi I_0 (1 - 10^{-\varepsilon b[\pm\mathbf{n}]})$$

Strictly speaking, the quantum yield values provided are apparent quantum yields connecting a stable state **±n** of the motor with the successive having an increased crossing number, **±n+1**. The metastable form connecting these two states (and the associated ultrafast thermal reaction that populates **±n+1**) is neglected because it is impossible to observe under our experimental conditions.

Energetic Considerations of the Winding Mechanism

Our experiments show that AMM **±n** works by an energy ratchet mechanism and the winding process is driven by light energy (only the (*S,R,R*) isomer is considered in this discussion).

Experimentally, the Gibbs free energy (G) of – and thus strain in – the system increases with increasing amount of crossings ($\boldsymbol{N}$), whereas the state with zero crossings ($\boldsymbol{N} = 0$) has the lowest energy. At thermal equilibrium, three states are populated, namely $\mathbf{-1}$, $\mathbf{0}$ and $\mathbf{+1}$. States with $\boldsymbol{N} > +1$ or $\boldsymbol{N} < -1$ were not observed.

The system can thermally equilibrate either by intermolecular nucleophile-imine exchange or intramolecular, thermal double bond isomerization. While the rate constant (k) of the former is independent of $\boldsymbol{N}$, the rate constant of the latter decreases with increasing $\boldsymbol{N}$.

Light-driven winding increases $\boldsymbol{N}$ stepwise by +1 and occurs by a photochemical *E/Z* isomerization, forming a metastable isomer (experimentally not observed) that relaxes by a fast thermal helix inversion (THI). The quantum yield (Φ) of the double bond isomerization decreases with increasing $\boldsymbol{N}$ and is therefore dependent on strain in the system. Experimentally, the system reaches up to +3 crossings.

With these observations in mind, we can derive the conditions which limit the number of crossings in our light-driven molecular machine (assuming that no competing nucleophile is present), considering a simple model for our nanoratchet (Supplementary Fig. 42).

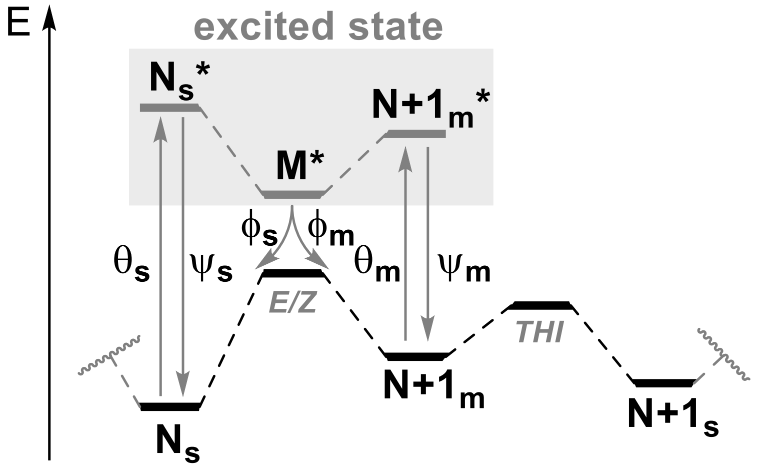

Supplementary Fig. 42 | Schematic representation describing the energetics of the winding mechanism. θ, ψ and ϕ are transition constants; horizontal lines represent qualitative energy levels of different winding states.

For a generic molecular motor to operate, an equilibrium between its states $\boldsymbol{N_s}$ (a stable state that was subjected to N turns), $\boldsymbol{N+1_m}$ (a metastable state that was subjected to N+1 turns) and $\boldsymbol{N+1_s}$ (a stable state that was subjected to N+1 turns) must be established, where ultimately the probabilities to find the system in a certain stable state will be regulated by the Boltzmann equation:

$$\left(\frac{p_{\mathbf{N+1_s}}}{p_{\mathbf{N_s}}}\right) = e^{\left(\frac{G_{\mathbf{N_s}} - G_{\mathbf{N+1_s}}}{k_B T}\right)}$$

$p_{\mathbf{N+1_s}}$ and $p_{\mathbf{N_s}}$ are the steady-state levels for $\boldsymbol{N+1_s}$ and $\boldsymbol{N_s}$, $G_{\mathbf{N_s}}$ and $G_{\mathbf{N+1_s}}$ are the free energies of $\boldsymbol{N+1_s}$ and $\boldsymbol{N_s}$, k_B is Boltzmann's constant and T is the temperature in Kelvin. This scenario can be described in more detail by considering the equilibria involving these three species on the same

potential energy surface and the possibility to populate the respective excited states of species $\boldsymbol{N_s}$ and $\boldsymbol{N+1_m}$, namely $\boldsymbol{N_s}^*$ and $\boldsymbol{N+1_m}^*$. The diabatic transition from ground to the excited state is regulated by the Bose-Einstein equations for absorption (transition constant θ), stimulated and spontaneous emission (transition constant ψ)[19]. It is known that for Feringa-type molecular motors the population of the productive excited state involved in the isomerization leads to the (almost barrierless) formation of a so-called dark-state $\boldsymbol{M}^*$[20,21]. When an equilibrium is reached between the different species and if we consider the two surfaces separated, the ground state will have:

$$\left(\frac{p_{\mathrm{N+1_m}}}{p_{\mathrm{N_s}}}\right) = e^{\left(\frac{G_{\mathrm{N_s}} - G_{\mathrm{N+1_m}}}{k_B T}\right)} \text{ and } \left(\frac{p_{\mathrm{N+1_s}}}{p_{\mathrm{N+1_m}}}\right) = e^{\left(\frac{G_{\mathrm{N+1_m}} - G_{\mathrm{N+1_s}}}{k_B T}\right)}$$

while at the excited state:

$$\left(\frac{p_{\mathrm{M^*}}}{p_{\mathrm{N_s^*}}}\right) = e^{\left(\frac{G_{\mathrm{N_s^*}} - G_{\mathrm{M^*}}}{k_B T}\right)} \text{ and } \left(\frac{p_{\mathrm{M^*}}}{p_{\mathrm{N+1_s^*}}}\right) = e^{\left(\frac{G_{\mathrm{N+1_m^*}} - G_{\mathrm{M^*}}}{k_B T}\right)}$$

The population of $\boldsymbol{M}^*$ leads to the non-adiabatic formation[20,21] of $\boldsymbol{N_s}$ and $\boldsymbol{N+1_m}$ with transition constants ϕ_s and ϕ_m, given the approximation that the directionality of the motion at the excited state does not depend on the state initially populated, but only by the characteristics of $\boldsymbol{M}^*$[I].

Given these premises, we can for example consider that at the stationary state the probability to photochemically populate the state $\boldsymbol{N+1_m}$ starting from $\boldsymbol{N_s}$ *via* $\boldsymbol{M}^*$ will be the product of the probabilities associated with each step involving these species[II]:

$$\mathrm{P}^{h\nu}_{\mathrm{N_s \to N+1_m}} = \frac{p_{\mathrm{N_s}} \theta_{\mathrm{s}} \cdot p_{\mathrm{N_s^*}} k_{\mathrm{N_s^* \to M^*}} \cdot p_{\mathrm{M^*}} \phi_{\mathrm{m}}}{p_{\mathrm{N_s^*}} \psi_{\mathrm{s}}} = p_{\mathrm{N_s}} \cdot \mathrm{P}'^{h\nu}_{\mathrm{N_s \to N+1_m}}$$

In this way, we can consider that at the stationary state, the sum of probabilities that lead from $\boldsymbol{N_s}$ and $\boldsymbol{N+1_s}$ should equal the sum of the ones that form $\boldsymbol{N+1_s}$ from $\boldsymbol{N_s}$. Thus:

$$1 = \frac{\mathrm{P}^{h\nu}_{\mathrm{N_s \to N+1_m}} \cdot \mathrm{P}_{\mathrm{N+1_m \to N+1_s}} + \mathrm{P}_{\mathrm{N_s \to N+1_m}} \cdot \mathrm{P}_{\mathrm{N+1_m \to N+1_s}}}{\mathrm{P}^{h\nu}_{\mathrm{N+1_m \to N_s}} \cdot \mathrm{P}_{\mathrm{N+1_s \to N+1_m}} + \mathrm{P}_{\mathrm{N+1_m \to N_s}} \cdot \mathrm{P}_{\mathrm{N+1_s \to N+1_m}}}$$

which can be rewritten as:

$$1 = \frac{p_{\mathrm{N_s}} \cdot \mathrm{P}'^{h\nu}_{\mathrm{N_s \to N+1_m}} \cdot p_{\mathrm{N+1_m}} k_{\mathrm{N+1_m \to N+1_s}} + p_{\mathrm{N_s}} k_{\mathrm{N_s \to N+1_m}} \cdot p_{\mathrm{N+1_m}} k_{\mathrm{N+1_m \to N+1_s}}}{p_{\mathrm{N+1_m}} \cdot \mathrm{P}'^{h\nu}_{\mathrm{N+1_m \to N_s}} \cdot p_{\mathrm{N+1_s}} k_{\mathrm{N+1_s \to N+1_m}} + p_{\mathrm{N+1_m}} k_{\mathrm{N+1_m \to N_s}} \cdot p_{\mathrm{N+1_s}} k_{\mathrm{N+1_s \to N+1_m}}}$$

and simplified to:

$$1 = \frac{p_{\mathrm{N_s}} k_{\mathrm{N+1_m \to N+1_s}} \cdot (\mathrm{P}'^{h\nu}_{\mathrm{N_s \to N+1_m}} + k_{\mathrm{N_s \to N+1_m}})}{p_{\mathrm{N+1_s}} k_{\mathrm{N+1_s \to N+1_m}} \cdot (\mathrm{P}'^{h\nu}_{\mathrm{N+1_m \to N_s}} + k_{\mathrm{N+1_m \to N_s}})}$$

finally obtaining:

[I] The excited state vibrationally coherent population of conical intersections is deliberately omitted in this treatment.

[II] We will not consider here the possibility of an up-hill population of $\boldsymbol{N+1_m}^*$ (or $\boldsymbol{N_s}^*$) from $\boldsymbol{M}^*$ and subsequent light emission to afford $\boldsymbol{N+1_m}$ (or $\boldsymbol{N_s}$).

$$\left(\frac{p_{\mathbf{N+1_s}}}{p_{\mathbf{N_s}}}\right) = \frac{k_{\mathbf{N+1_m \to N+1_s}}}{k_{\mathbf{N+1_s \to N+1_m}}} \cdot \frac{(\mathrm{P'}^{hv}_{\mathbf{N_s \to N+1_m}} + k_{\mathbf{N_s \to N+1_m}})}{(\mathrm{P'}^{hv}_{\mathbf{N+1_m \to N_s}} + k_{\mathbf{N+1_m \to N_s}})}$$

Thus, when $k_{\mathbf{N_s \to N+1_m}} \ll \mathrm{P'}^{hv}_{\mathbf{N_s \to N+1_m}}$ and $k_{\mathbf{N+1_m \to N_s}} \ll \mathrm{P'}^{hv}_{\mathbf{N+1_m \to N_s}}$[III], we have:

$$\left(\frac{p_{\mathbf{N+1_s}}}{p_{\mathbf{N_s}}}\right) = e^{\left(\frac{G_{\mathbf{N+1_m}} - G_{\mathbf{N+1_s}}}{k_B T}\right)} \cdot \frac{\mathrm{P'}^{hv}_{\mathbf{N_s \to N+1_m}}}{\mathrm{P'}^{hv}_{\mathbf{N+1_m \to N_s}}}$$

We can simplify this equation by assuming that the ratio of the photochemical probabilities is the photostationary state (PSS)[22]:

$$\frac{\mathrm{P'}^{hv}_{\mathbf{N_s \to N+1_m}}}{\mathrm{P'}^{hv}_{\mathbf{N+1_m \to N_s}}} = \frac{\Phi_{\mathbf{N_s \to N+1_m}} \varepsilon_{\mathbf{N_s}}}{\Phi_{\mathbf{N+1_m \to N_s}} \varepsilon_{\mathbf{N+1_m}}}$$

thus:

$$\left(\frac{p_{\mathbf{N+1_s}}}{p_{\mathbf{N_s}}}\right) = e^{\left(\frac{G_{\mathbf{N+1_m}} - G_{\mathbf{N+1_s}}}{k_B T}\right)} \cdot \frac{\Phi_{\mathbf{N_s \to N+1_m}} \varepsilon_{\mathbf{N_s}}}{\Phi_{\mathbf{N+1_m \to N_s}} \varepsilon_{\mathbf{N+1_m}}} = \frac{k_{\mathbf{N+1_m \to N+1_s}}}{k_{\mathbf{N+1_s \to N+1_m}}} \cdot \frac{\Phi_{\mathbf{N_s \to N+1_m}} \varepsilon_{\mathbf{N_s}}}{\Phi_{\mathbf{N+1_m \to N_s}} \varepsilon_{\mathbf{N+1_m}}}$$

$\Phi_{\mathbf{N_s \to N+1_m}}$ describes the quantum yield of the $\boldsymbol{N_s} \to \boldsymbol{N} + \mathbf{1}_{\boldsymbol{m}}$ process starting from $\boldsymbol{N_s}$ upon light absorption and incorporates both the Bose-Einstein terms and the non-adiabatic terms. We can also explicitly consider the probability of the diabatic transition as proportional to the absorptivity of $\boldsymbol{N_s}$ ($\varepsilon_{\mathbf{s}}$).

This equation can be zeroed in the following extreme cases, consequently impeding winding and thus the population of $\boldsymbol{N} + \mathbf{1}_{\boldsymbol{s}}$:

1. $G_{\mathbf{N+1_m}} \ll G_{\mathbf{N+1_s}}$, hence the metastable state ensuing from the photochemical step ($\boldsymbol{N} + \mathbf{1}_{\boldsymbol{m}}$) is more stable than the next "stable" state ($\boldsymbol{N} + \mathbf{1}_{\boldsymbol{s}}$) that is generated from the thermal helix inversion (THI) step and/or $k_{\mathbf{N+1_s \to N+1_m}} \gg k_{\mathbf{N+1_m \to N+1_s}}$, hence further winding is kinetically prevented;

2. $\Phi_{\mathbf{N_s \to N+1_m}} \ll \Phi_{\mathbf{N+1_m \to N_s}}$, given similar molar absorptivities $\varepsilon_{\mathbf{s}}$ and $\varepsilon_{\mathbf{m}}$, hence the photochemical population of $\boldsymbol{N} + \mathbf{1}_{\boldsymbol{m}}$ will not be feasible;

3. $\varepsilon_{\mathbf{s}} \ll \varepsilon_{\mathbf{m}}$ at a given wavelength of irradiation, preventing the excitation of $\boldsymbol{N_s}$ and the formation of $\boldsymbol{N} + \mathbf{1}_{\boldsymbol{m}}$.

III Conditions typical of a photochemically-fueled motor with an E/Z barrier that prevents the thermal population of $\boldsymbol{N} + \mathbf{1}_{\boldsymbol{m}}$ from $\boldsymbol{N_s}$.

References

1. Ballot, S. & Noiret, N. Synthesis of new C60 based phosphines. *Tetrahedron Lett.* **44**, 8811–8814 (2003).

2. Konken, C. P. *et al.* Development of symmetric O-BODIPYs with different optical properties as building blocks for the synthesis of ligands for multimodal imaging. *Dye. Pigment.* **158**, 88–96 (2018).

3. Li, Q. *et al.* Gram scale synthesis of functionalized and optically pure Feringa's motors. *Tetrahedron* **73**, 4874–4882 (2017).

4. Van Dijken, D. J., Chen, J., Stuart, M. C. A., Hou, L. & Feringa, B. L. Amphiphilic Molecular Motors for Responsive Aggregation in Water. *J. Am. Chem. Soc.* **138**, 660–669 (2016).

5. Tian, Y. K., Shi, Y. G., Yang, Z. S. & Wang, F. Responsive supramolecular polymers based on the bis[alkynylplatinum(II)] terpyridine molecular tweezer/arene recognition motif. *Angew. Chemie - Int. Ed.* **53**, 6090–6094 (2014).

6. Li, Q. *et al.* Macroscopic contraction of a gel induced by the integrated motion of light-driven molecular motors. *Nat. Nanotechnol.* **10**, 161–165 (2015).

7. Hoops, S. *et al.* COPASI--a COmplex PAthway SImulator. *Bioinformatics* **22**, 3067–3074 (2006).

8. Warnke, S. *et al.* Protomers of benzocaine: Solvent and permittivity dependence. *J. Am. Chem. Soc.* **137**, 4236–4242 (2015).

9. Mason, E. A. & McDaniel, E. W. *Transport Properties of Ions in Gases*. *Transport Properties of Ions in Gases* (Wiley, 1988). doi:10.1002/3527602852

10. Mesleh, M. F., Hunter, J. M., Shvartsburg, A. A., Schatz, G. C. & Jarrold, M. F. Structural Information from Ion Mobility Measurements: Effects of the Long-Range Potential. *J. Phys. Chem.* **100**, 16082–16086 (1996).

11. Frisch, M. J. *et al.* Gaussian 16, Revision B.01. Gaussian, Inc., Wallingford CT 2016.

12. Grimme, S., Bannwarth, C. & Shushkov, P. A Robust and Accurate Tight-Binding Quantum Chemical Method for Structures, Vibrational Frequencies, and Noncovalent Interactions of Large Molecular Systems Parametrized for All spd-Block Elements (Z = 1-86). *J. Chem. Theory Comput.* **13**, 1989–2009 (2017).

13. Bannwarth, C., Ehlert, S. & Grimme, S. GFN2-xTB - An Accurate and Broadly Parametrized Self-Consistent Tight-Binding Quantum Chemical Method with Multipole Electrostatics and Density-Dependent Dispersion Contributions. *J. Chem. Theory Comput.* **15**, 1652–1671 (2019).

14. Pracht, P., Caldeweyher, E., Ehlert, S. & Grimme, S. A Robust Non-Self-Consistent Tight-Binding Quantum Chemistry Method for Large Molecules. *ChemRxiv* (2019). doi:10.26434/chemrxiv.8326202.v1

15. Spicher, S. & Grimme, S. Robust Atomistic Modeling of Materials, Organometallic, and Biochemical Systems. *Angew. Chemie Int. Ed.* **59**, 15665–15673 (2020).

16. Breßler, I., Kohlbrecher, J. & Thünemann, A. F. SASfit: A tool for small-angle scattering data analysis using a library of analytical expressions. *J. Appl. Crystallogr.* **48**, 1587–1598 (2015).

17. Glatter, O. & Kratky, O. *Small angle X-ray scattering*. (Academic Press, 1982).

18. Ji, Y., DiRocco, D. A., Hong, C. M., Wismer, M. K. & Reibarkh, M. Facile Quantum Yield Determination via NMR Actinometry. *Org. Lett.* **20**, 2156–2159 (2018).

19. Astumian, R. D. Optical vs. chemical driving for molecular machines. *Faraday Discuss.* **195**, 583–597 (2017).

20. Conyard, J. *et al.* Ultrafast dynamics in the power stroke of a molecular rotary motor. *Nat. Chem. 2012 47* **4**, 547–551 (2012).

21. Pooler, D. R. S. *et al.* Effect of charge-transfer enhancement on the efficiency and rotary mechanism of an oxindole-based molecular motor. *Chem. Sci.* **12**, 7486–7497 (2021).

22. Stranius, K. & Börjesson, K. Determining the Photoisomerization Quantum Yield of Photoswitchable Molecules in Solution and in the Solid State. *Sci. Rep.* **7**, 41145 (2017).

Spectra Appendix

^{1}H and ^{13}C NMR spectra for compound **S2**

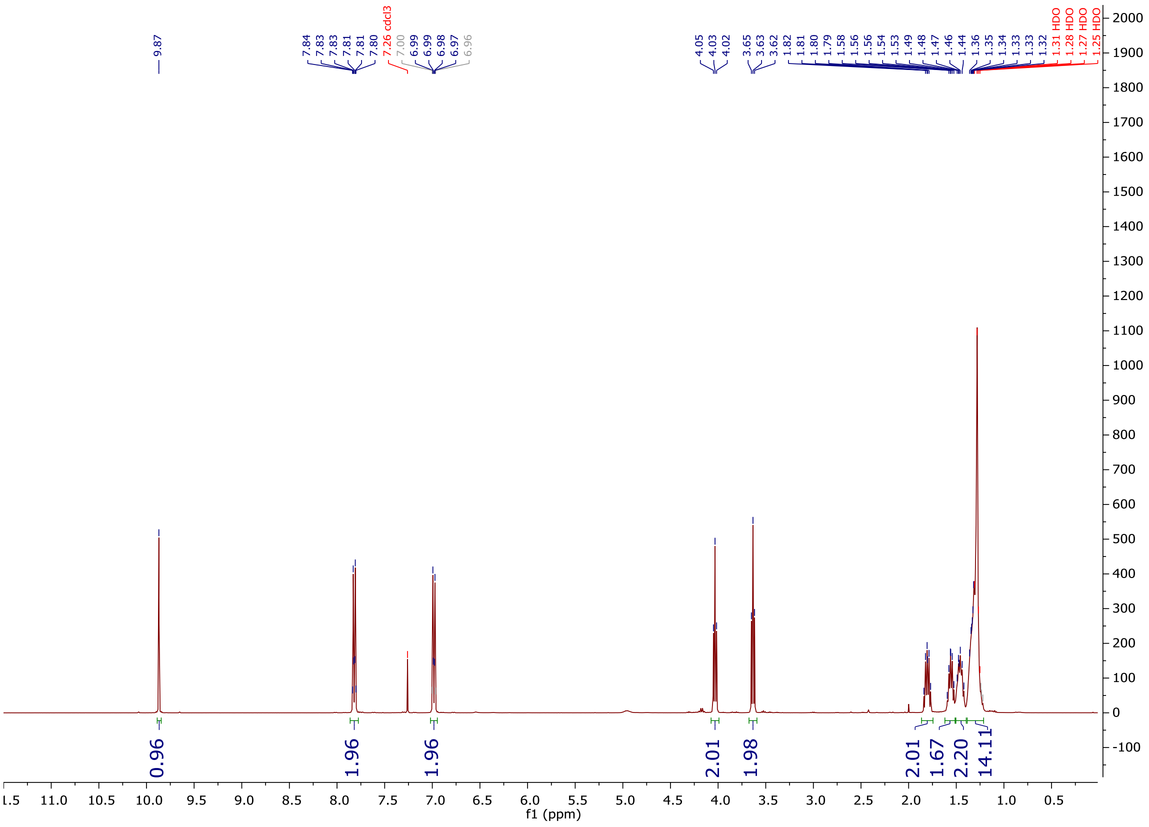

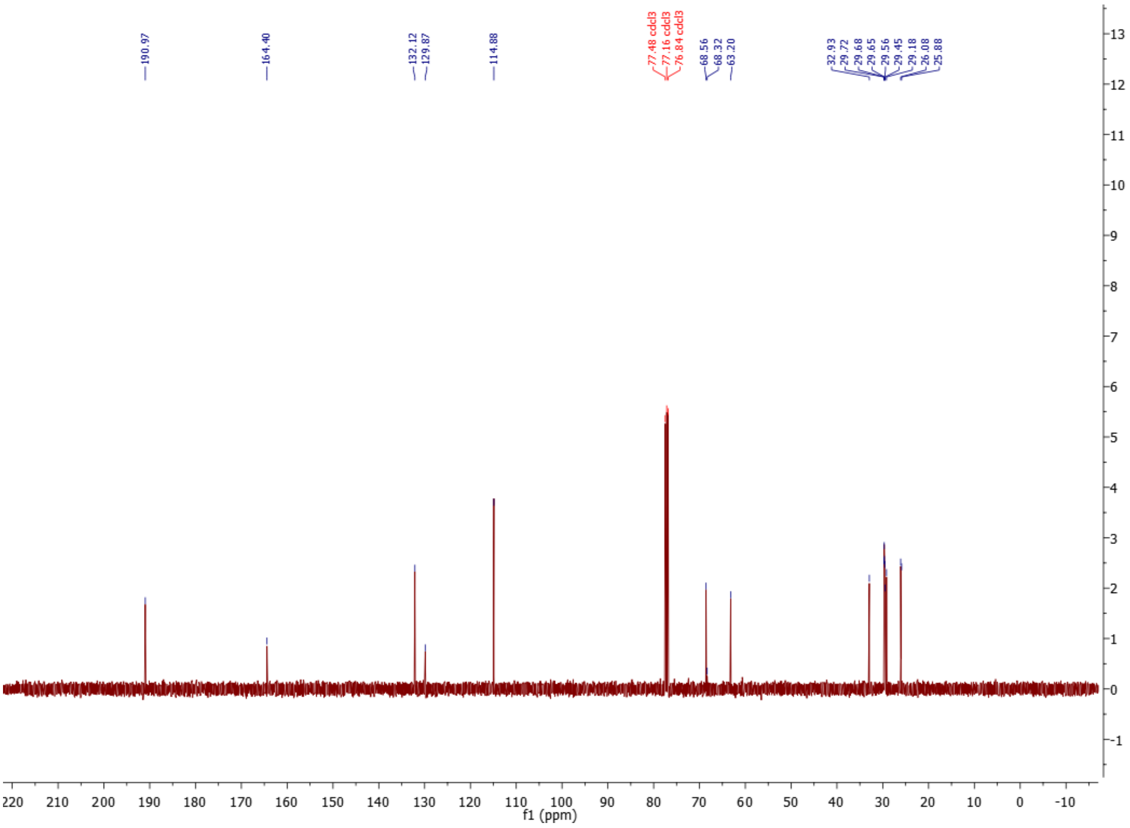

^{1}H and ^{13}C NMR spectra for compound **S3**

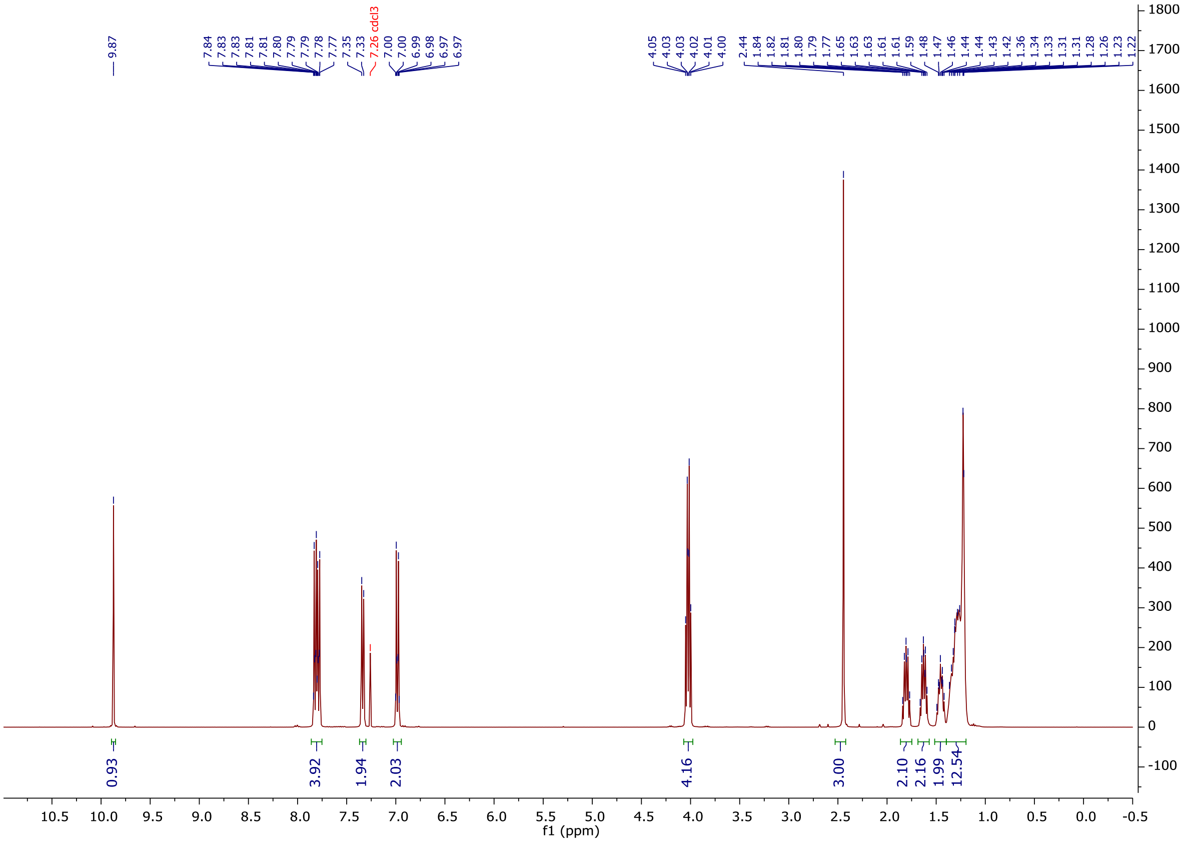

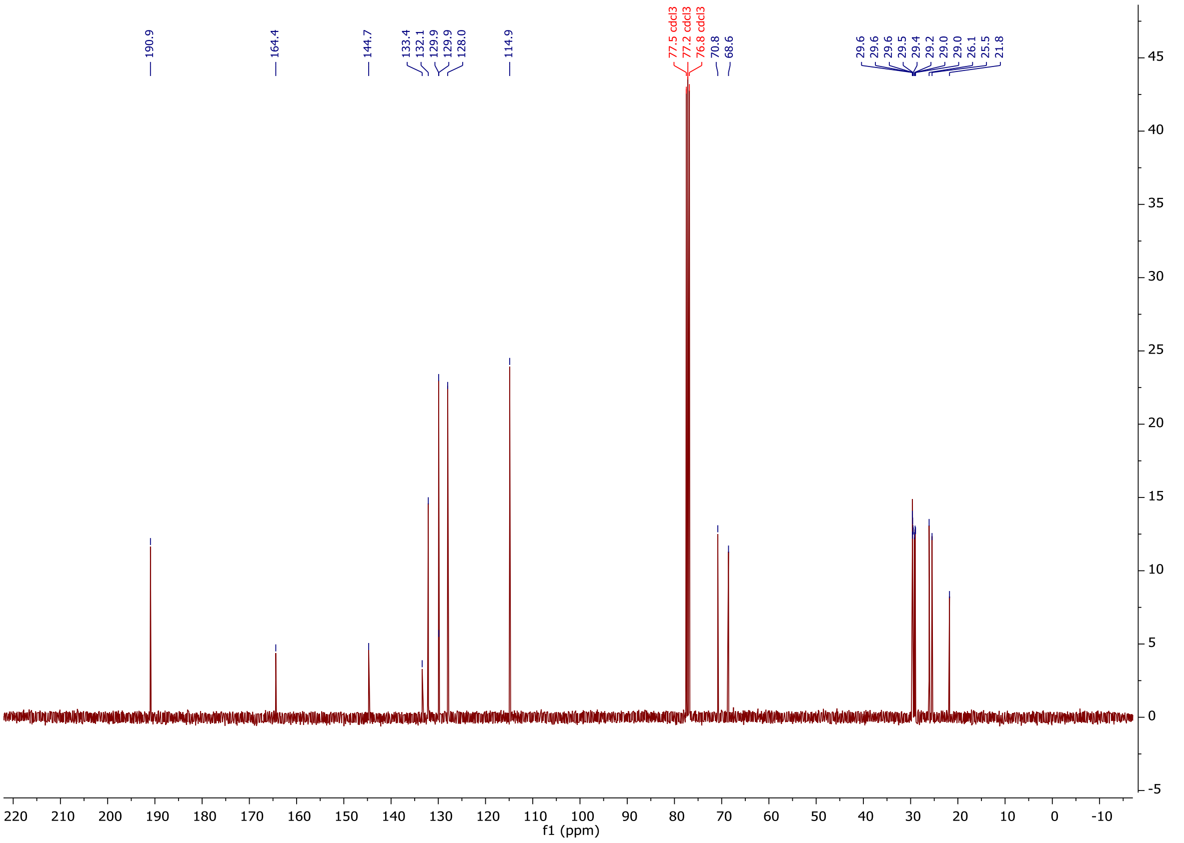

^{1}H and ^{13}C NMR spectra for compound **S5**

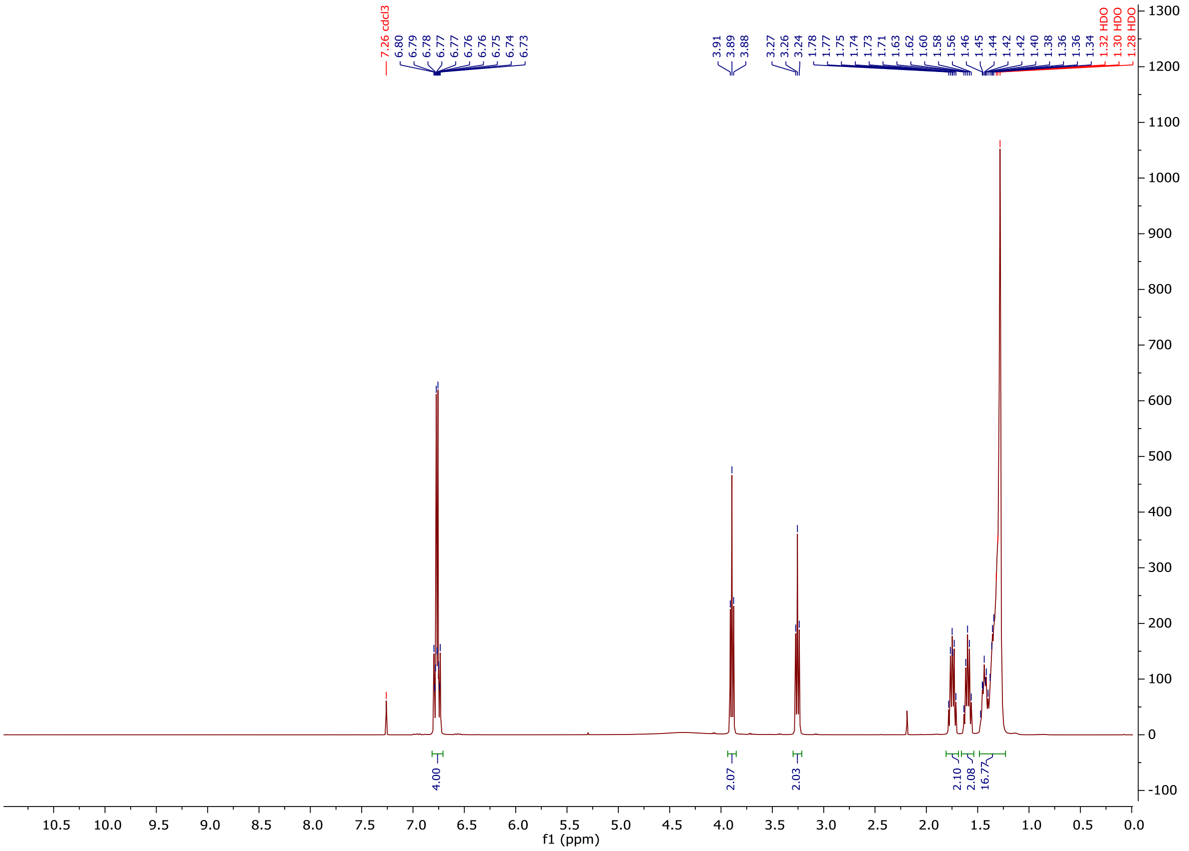

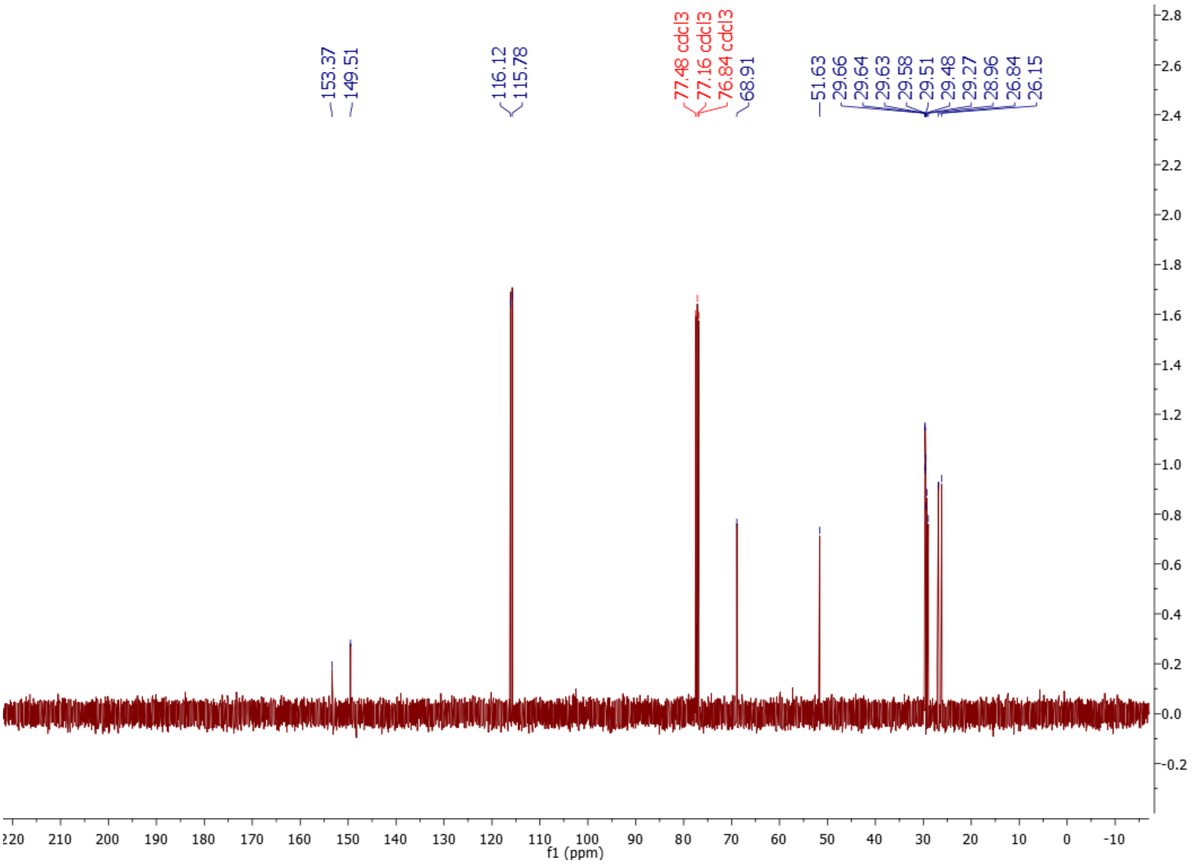

^{1}H and ^{13}C NMR spectra for compound **S10**

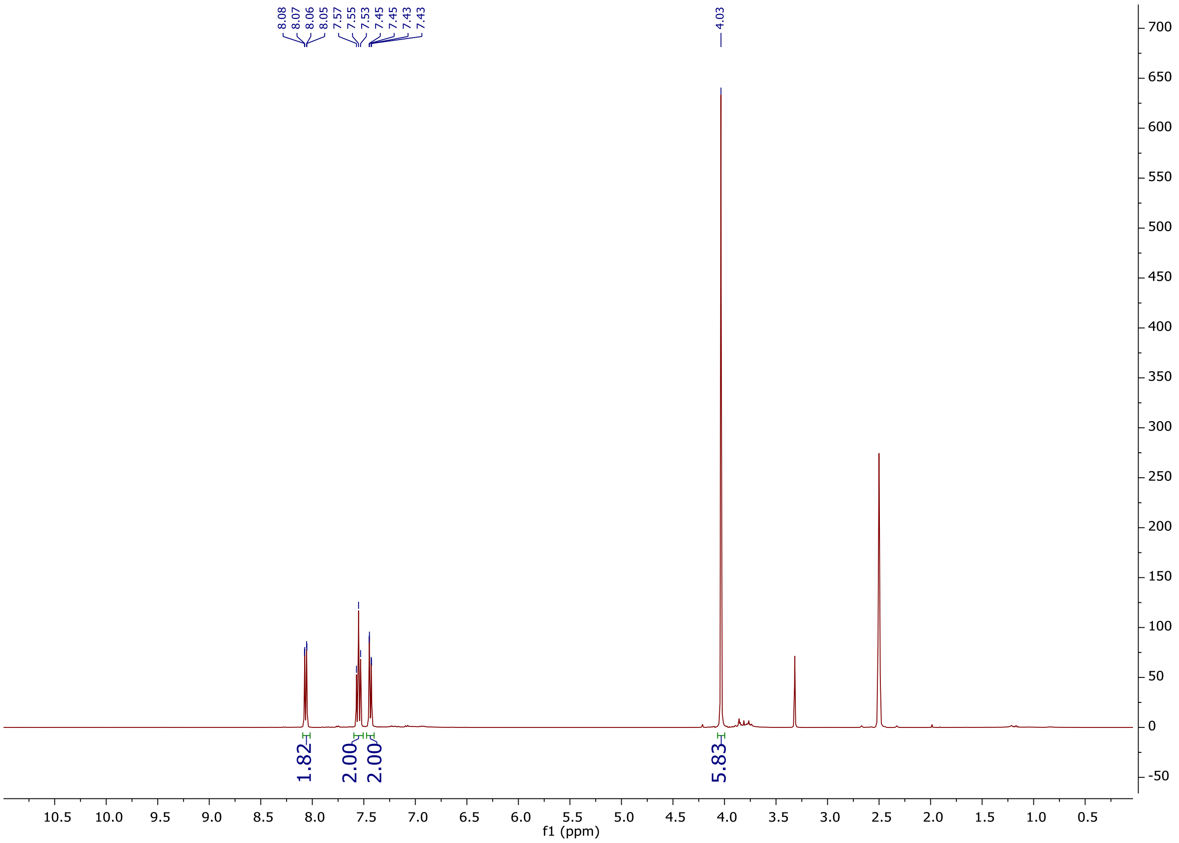

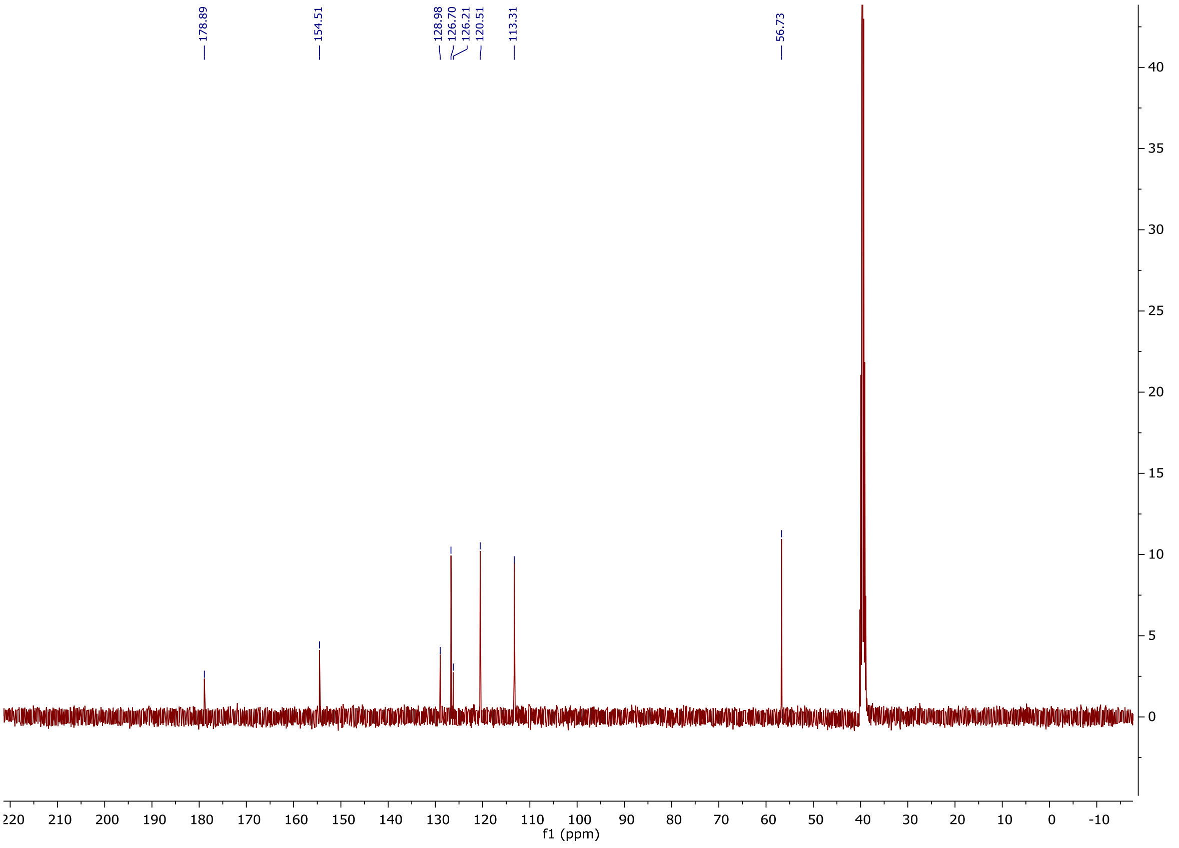

^{1}H NMR spectrum for compound **S12**

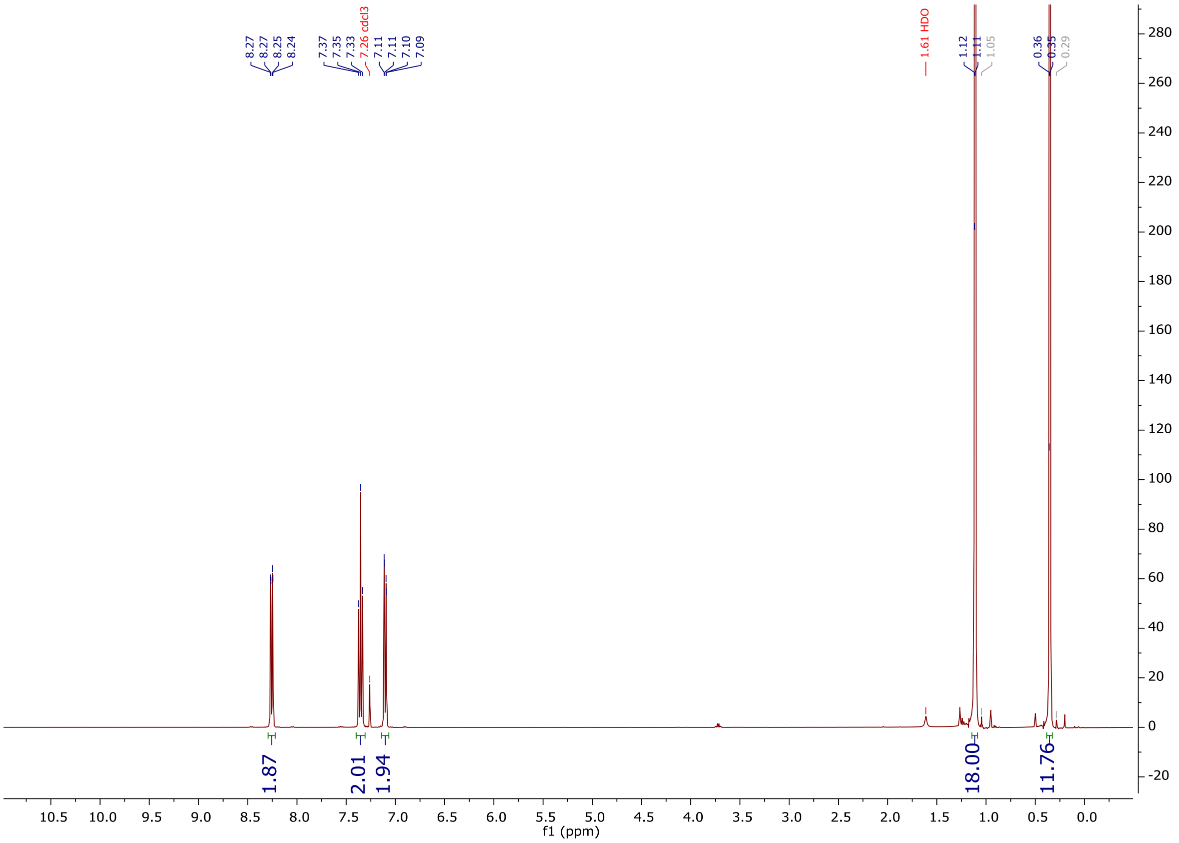

^1H NMR spectrum for compound **S13**

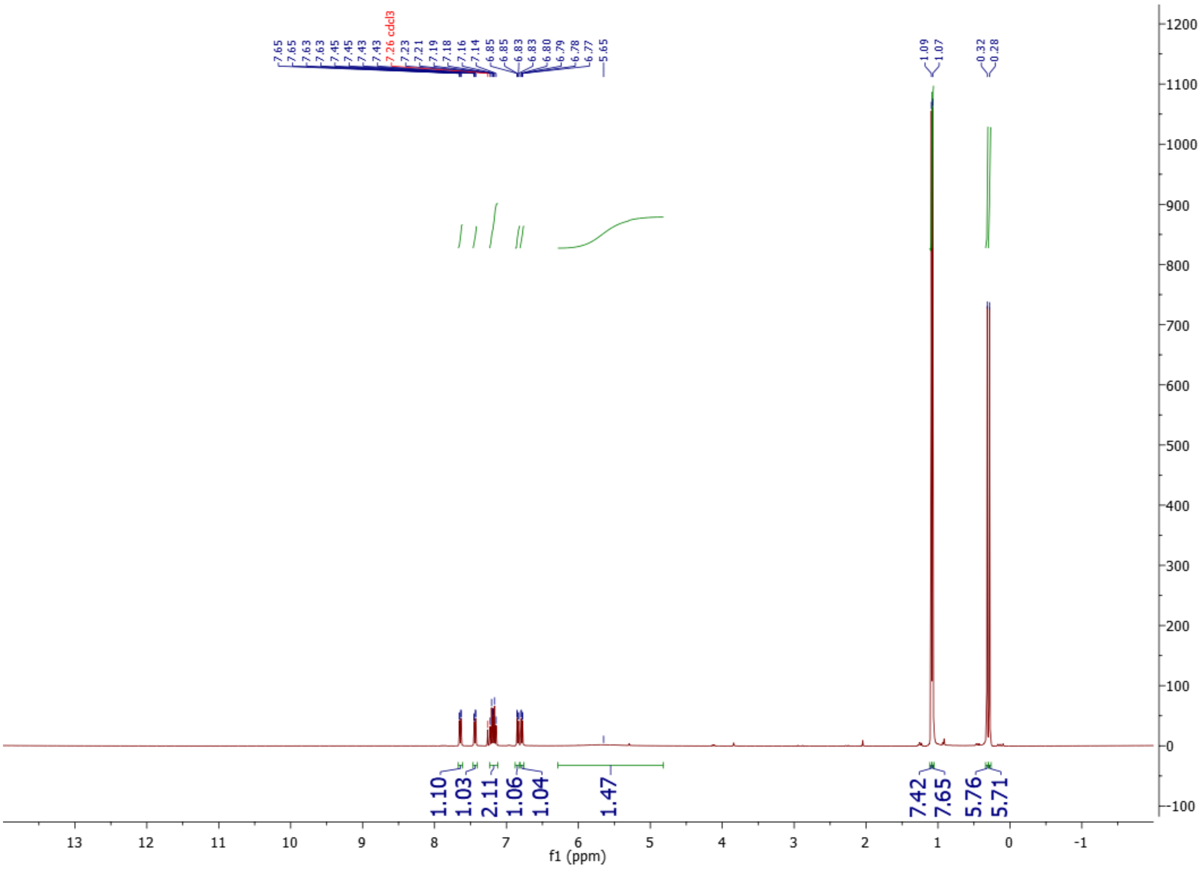

^{1}H NMR spectrum for compound (*R*,*R*,*R*)-**S15**

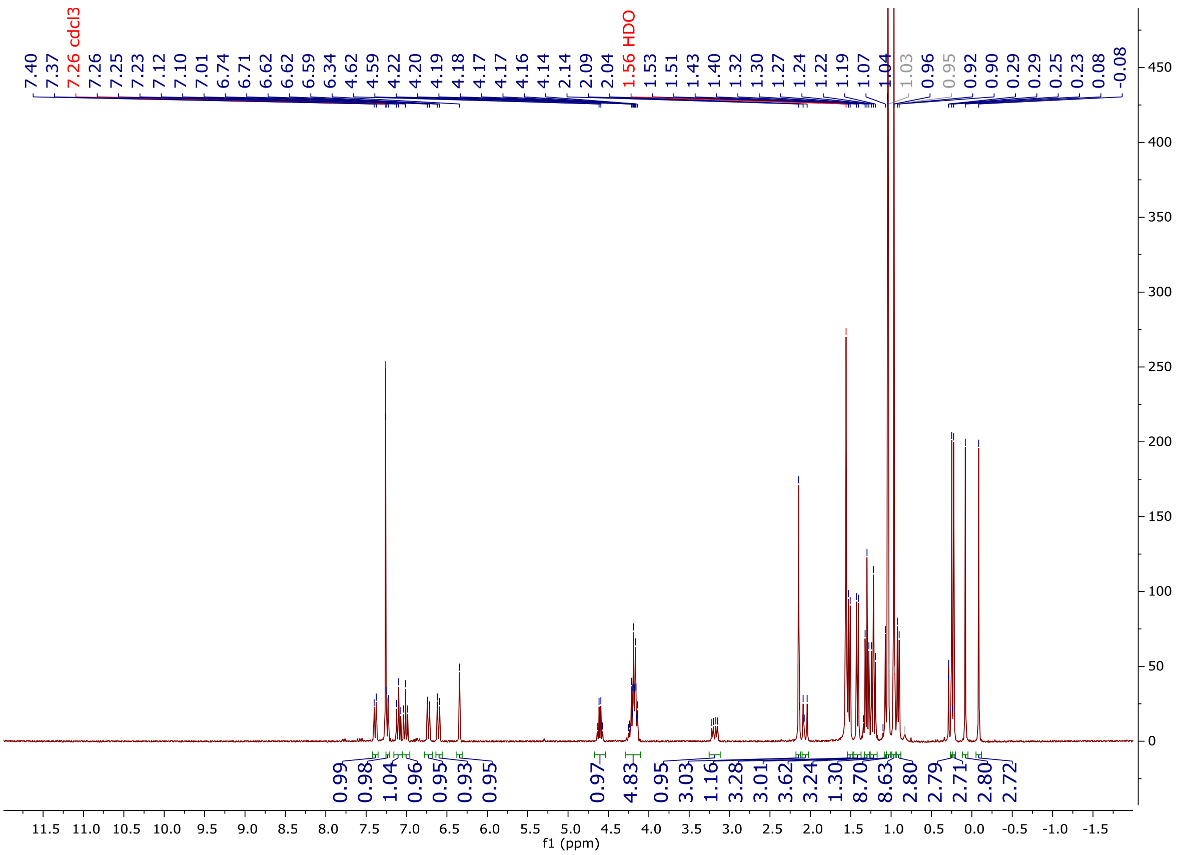

^{1}H NMR spectrum for compound (*S*,*R*,*R*)-**S15**

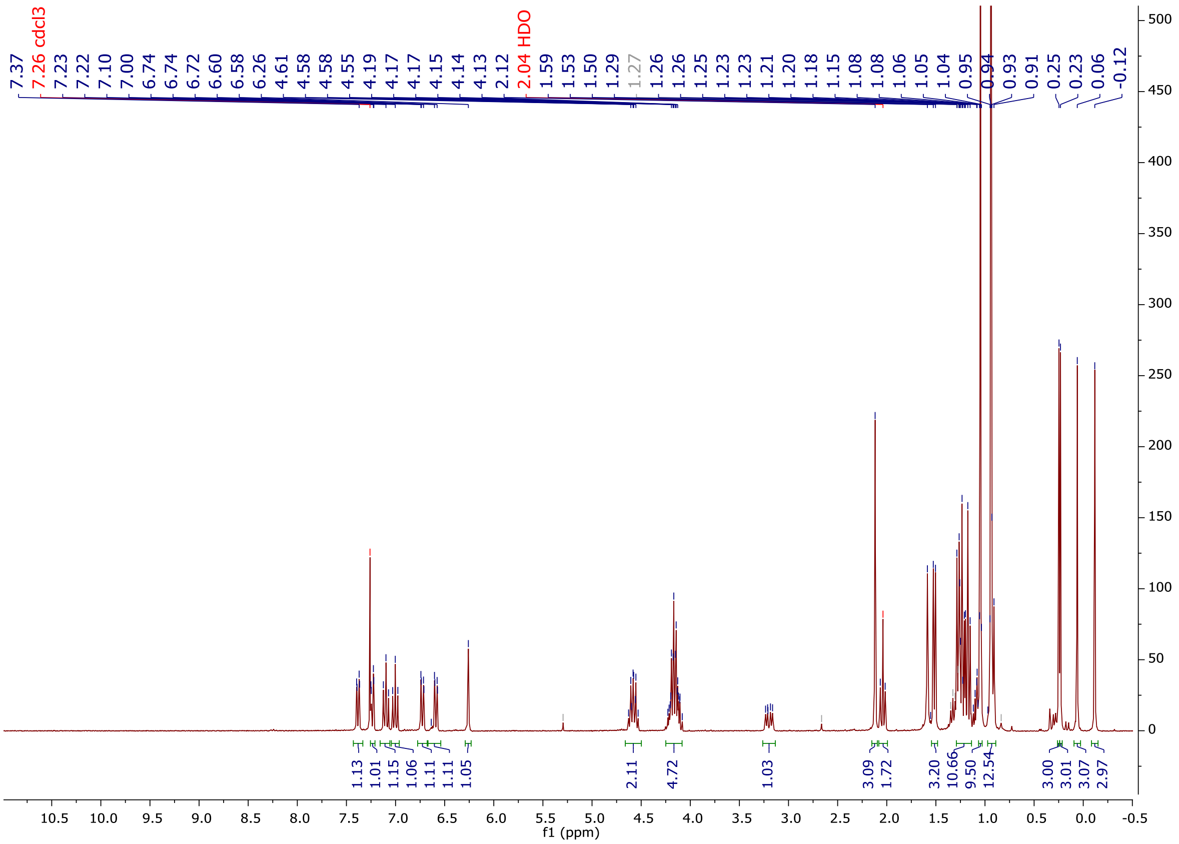

^{1}H and ^{13}C NMR spectra for compound (*R*,*R*,*R*)-**S16**

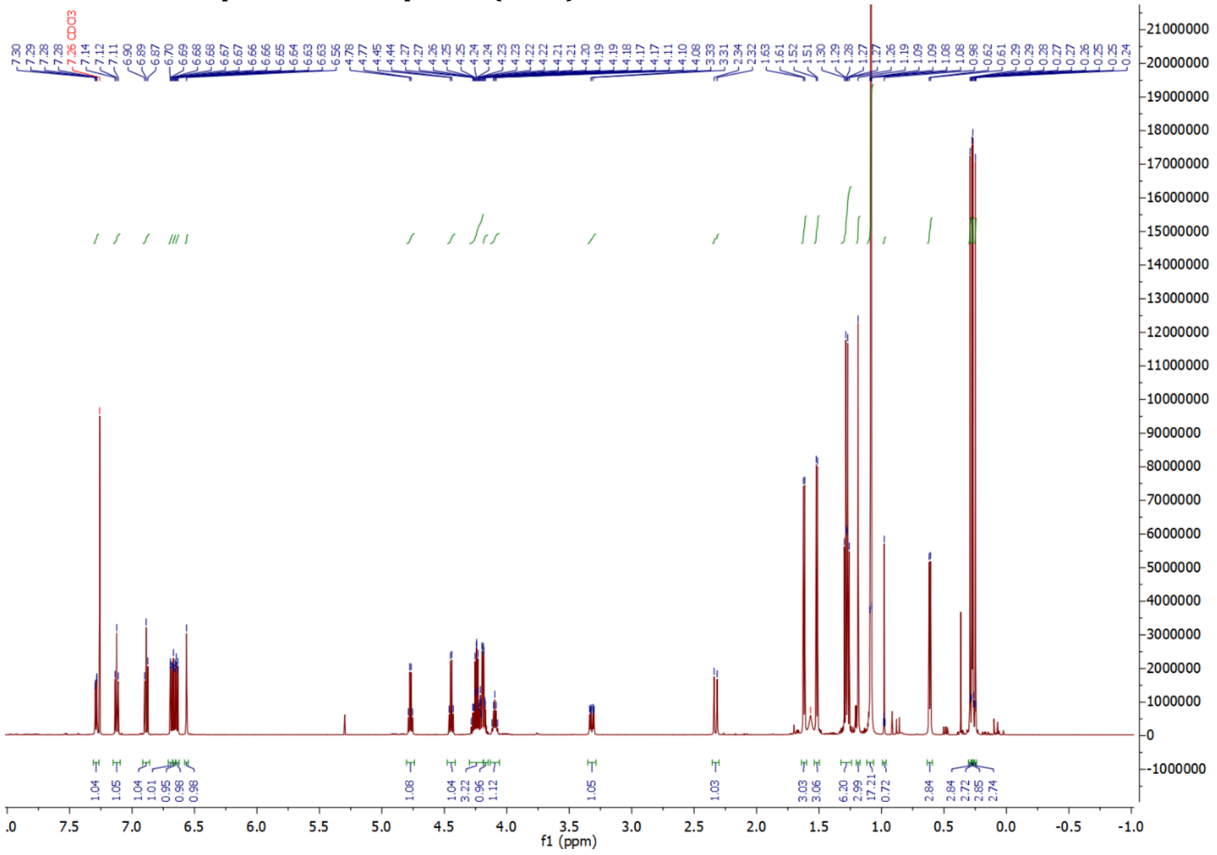

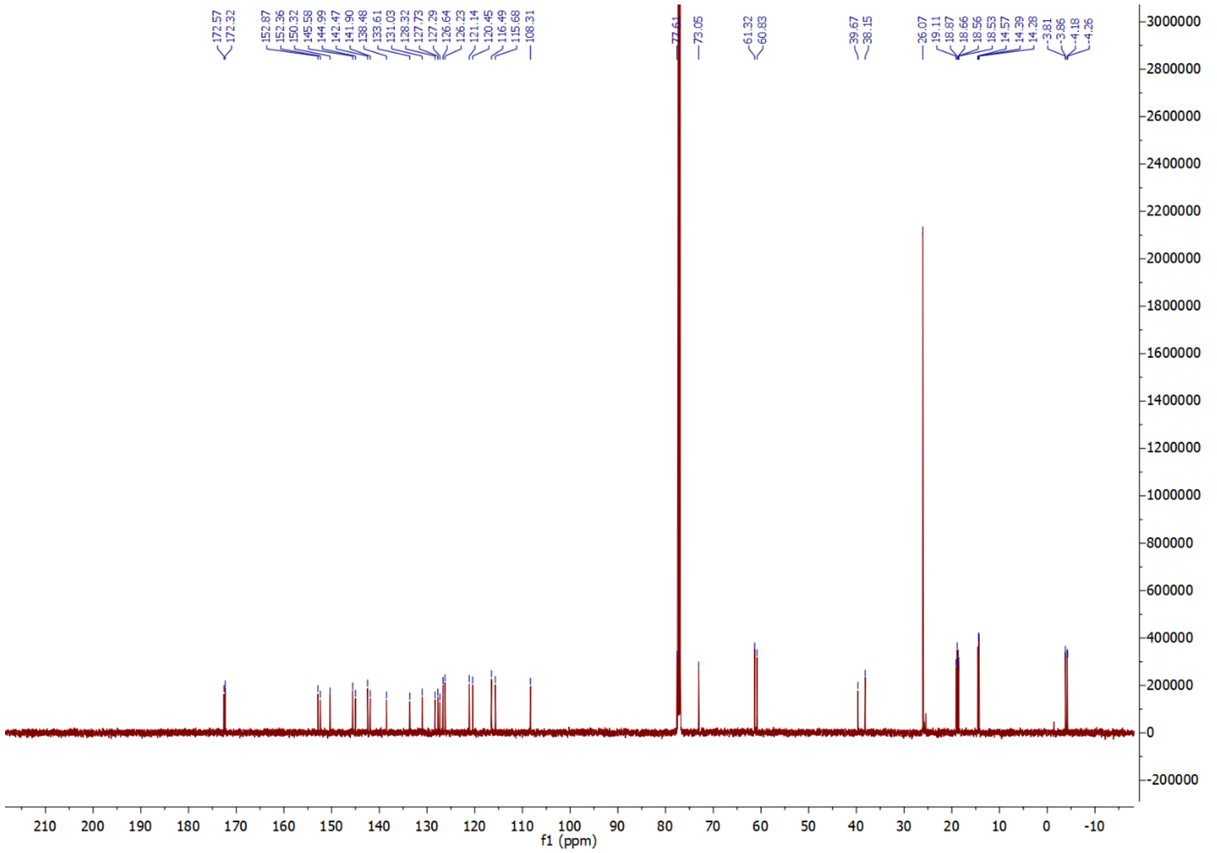

^{1}H,^{13}C HSQC NMR spectrum for compound (*R*,*R*,*R*)-**S16**

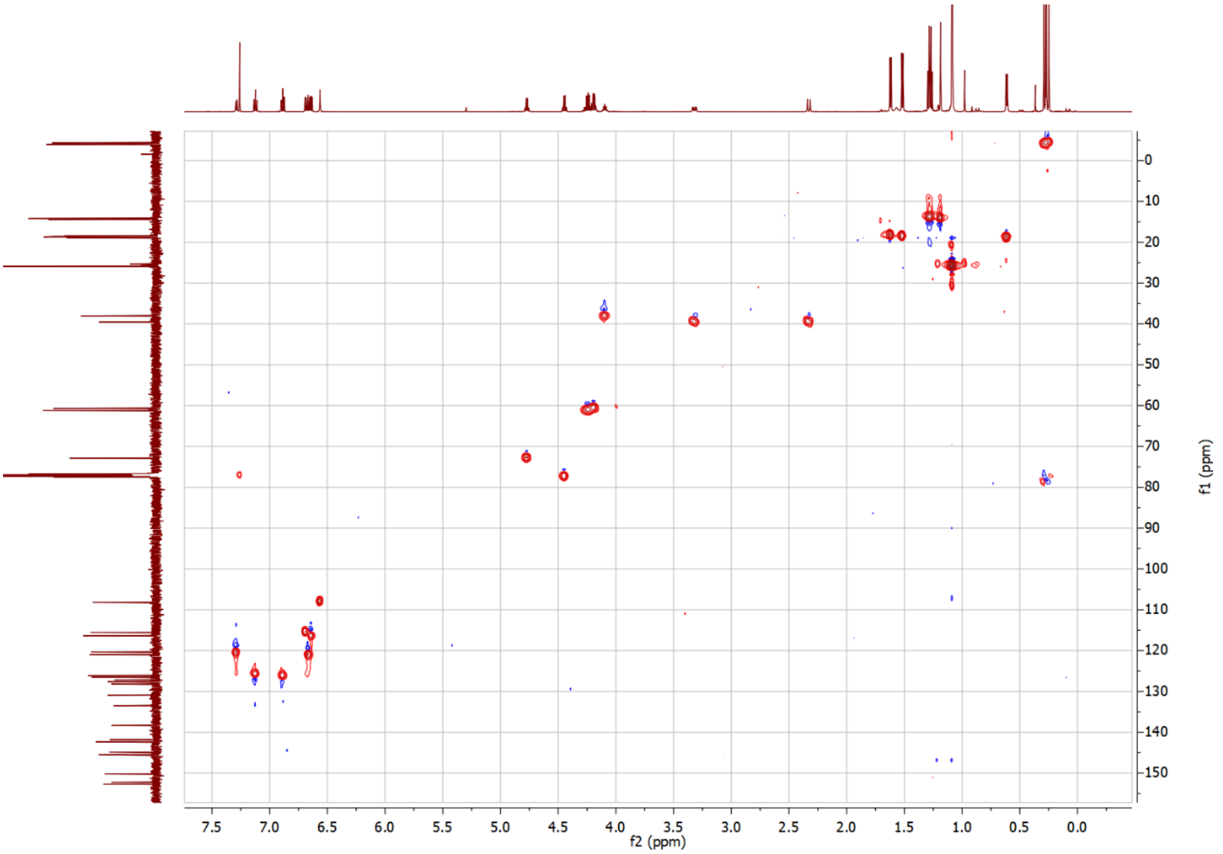

^{1}H,^{13}C HMBC NMR spectrum for compound (*R*,*R*,*R*)-**S16**

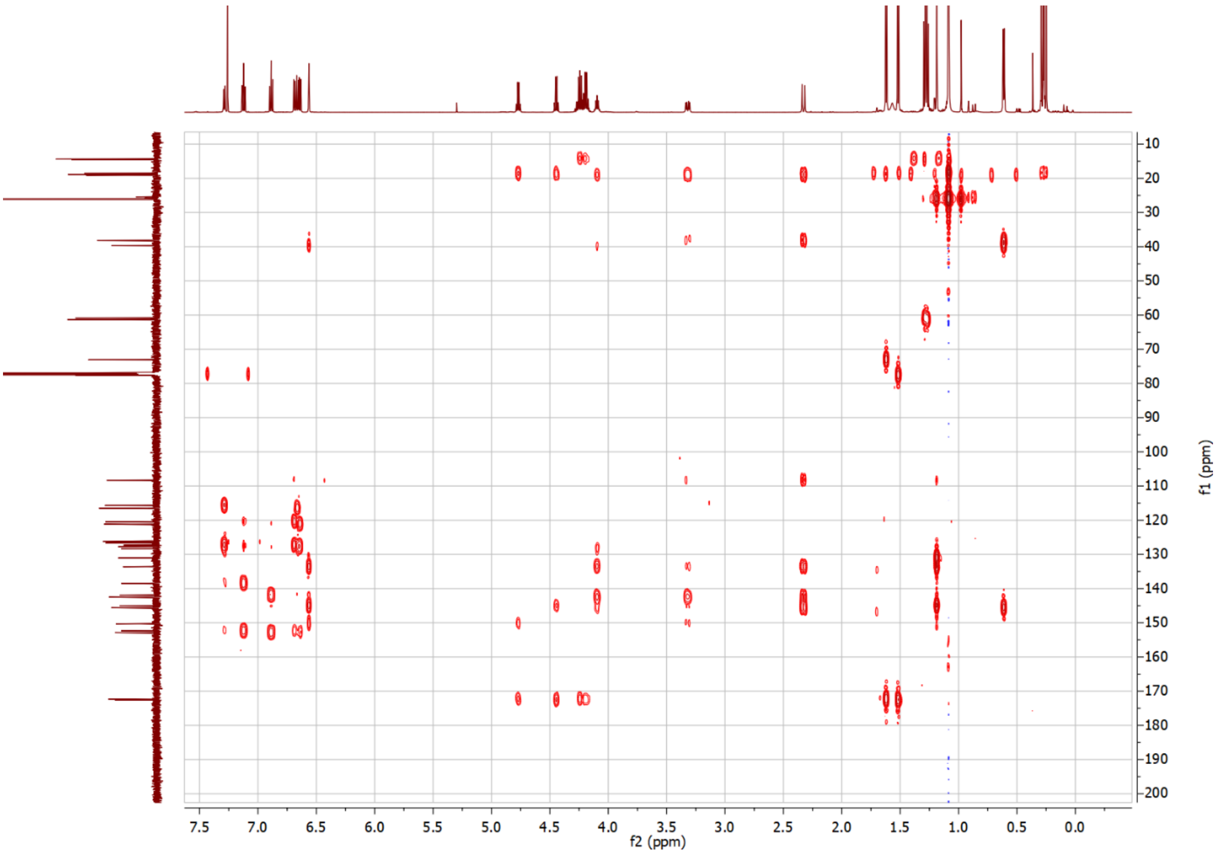

^{1}H and ^{13}C NMR spectra for compound (S,*R*,*R*)-**S16**

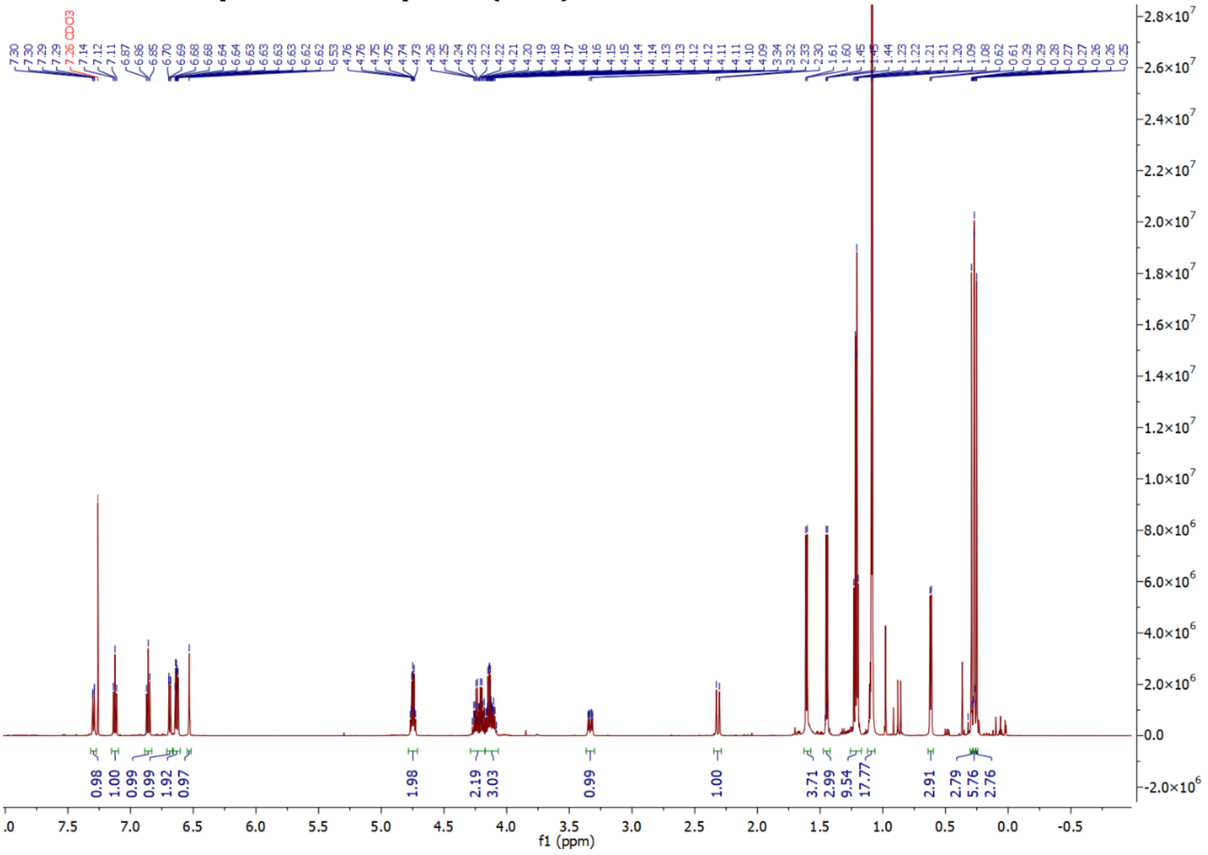

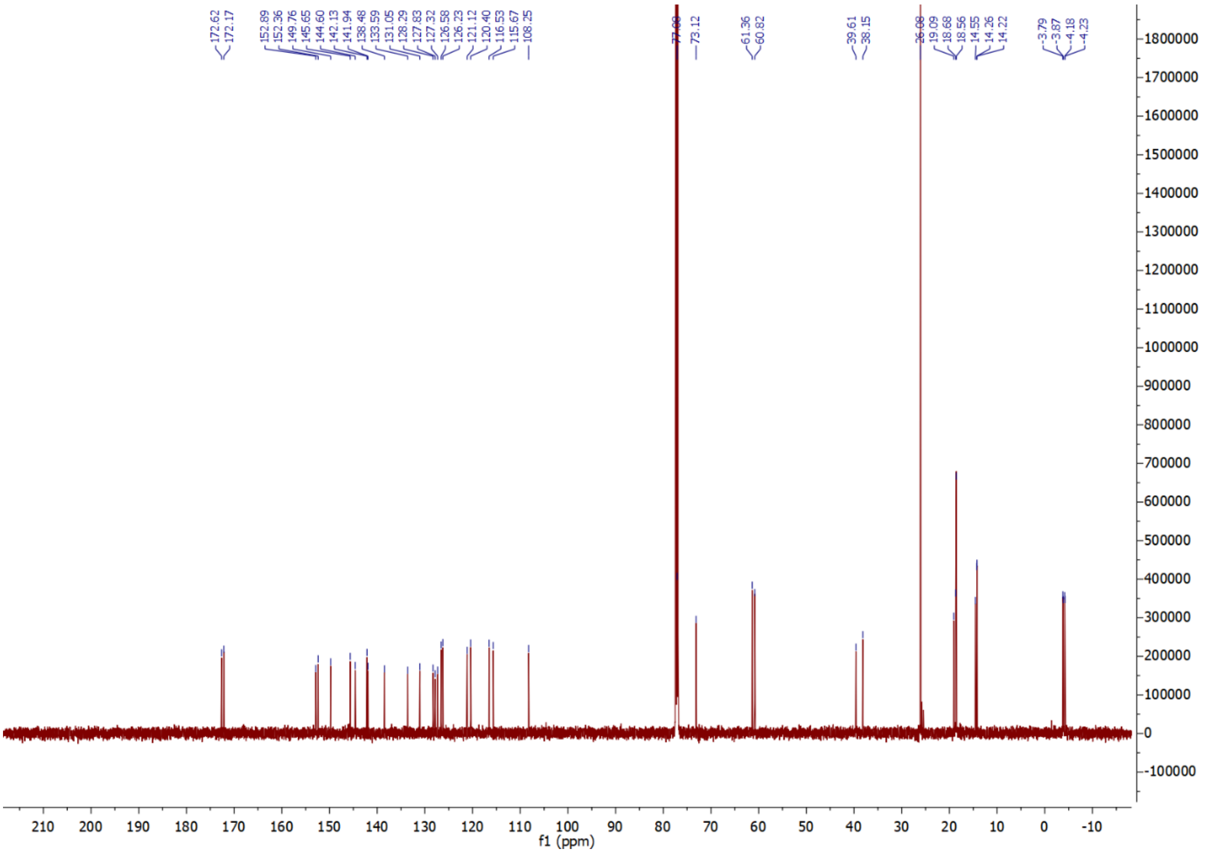

$^1H,^{13}C$ HSQC NMR spectrum for compound (S,*R*,*R*)-**S16**

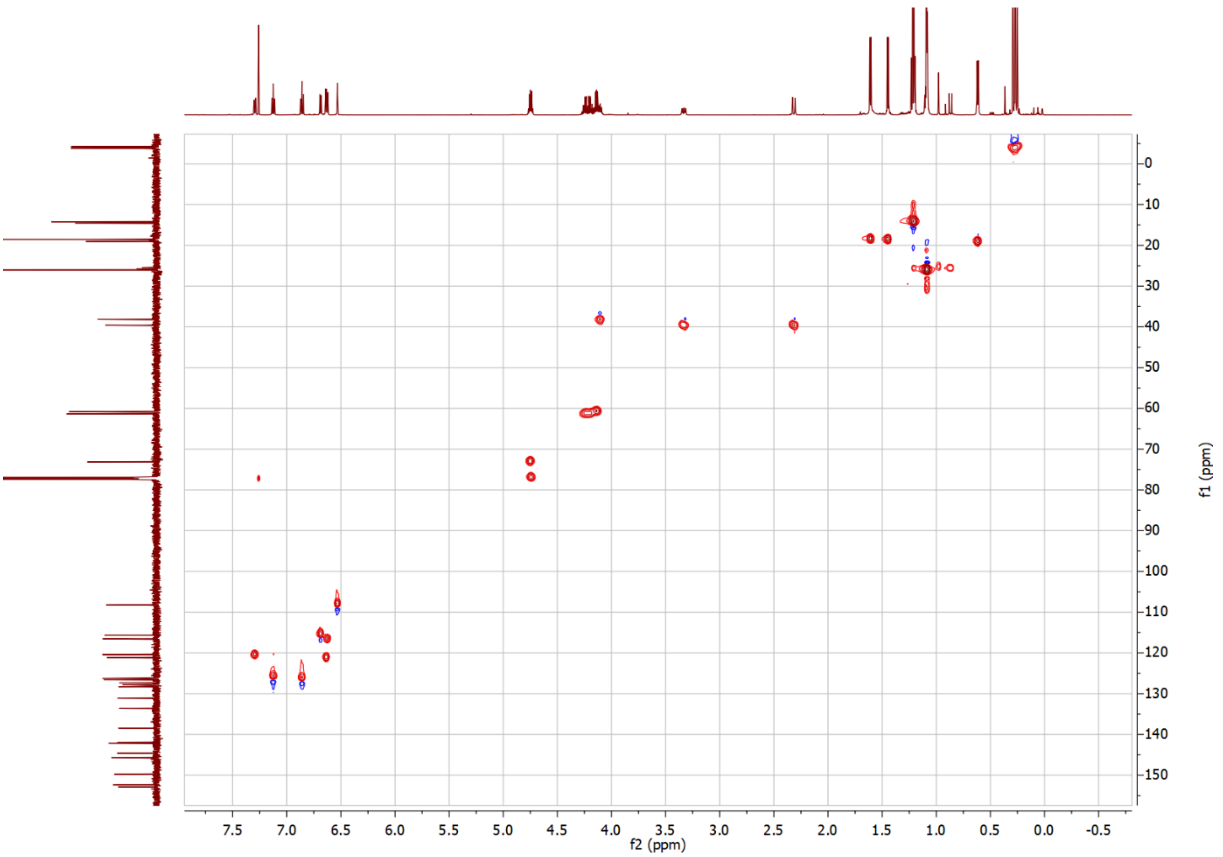

$^1H,^{13}C$ HMBC NMR spectrum for compound (*S*,*R*,*R*)-**S16**

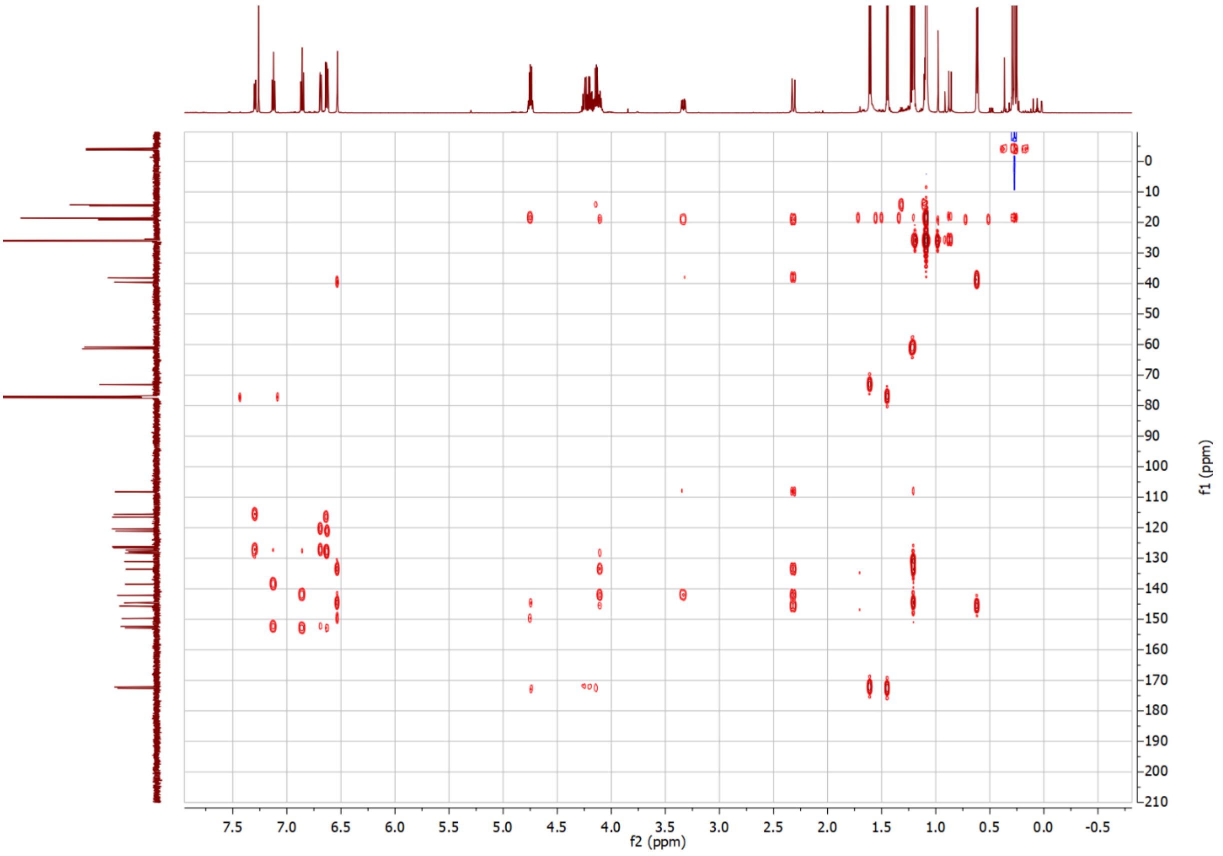

^1H and ^{13}C NMR spectra for compound (*R*,*R*,*R*)-**S17**

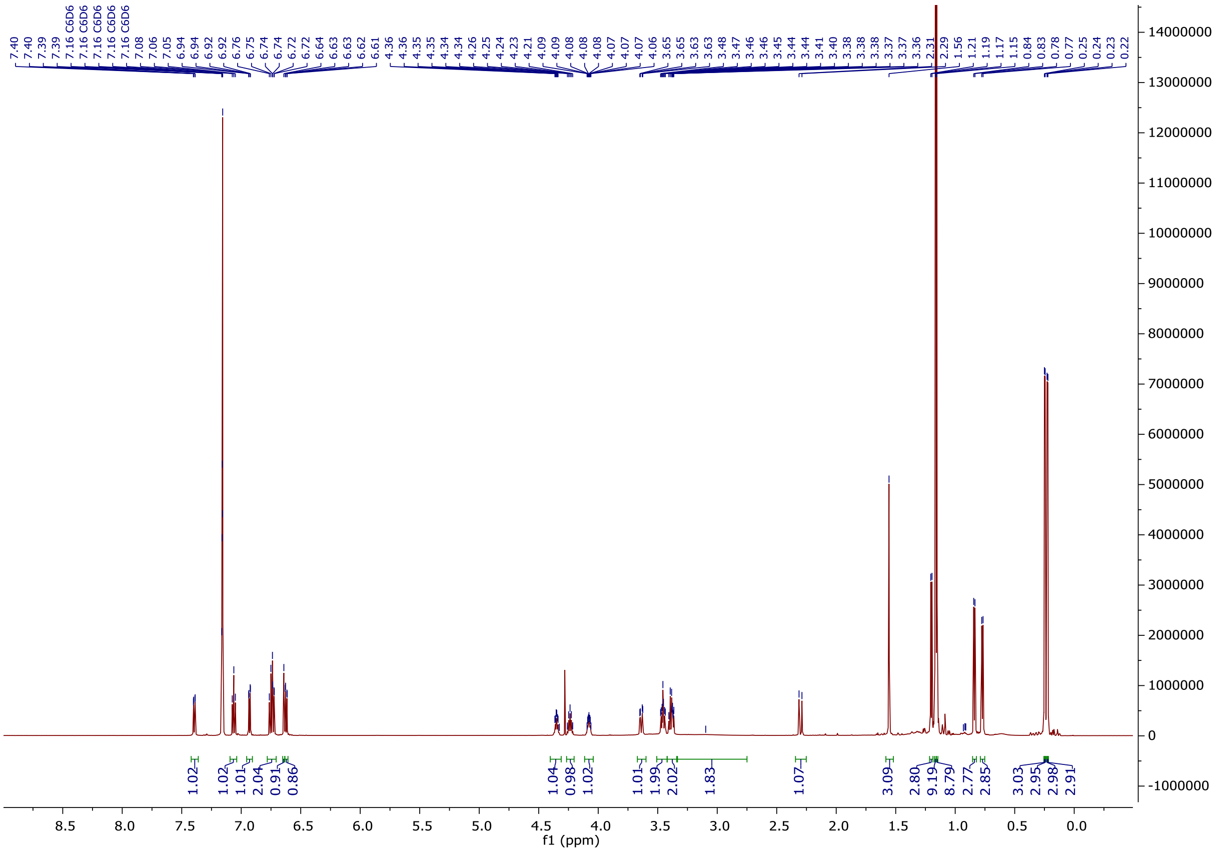

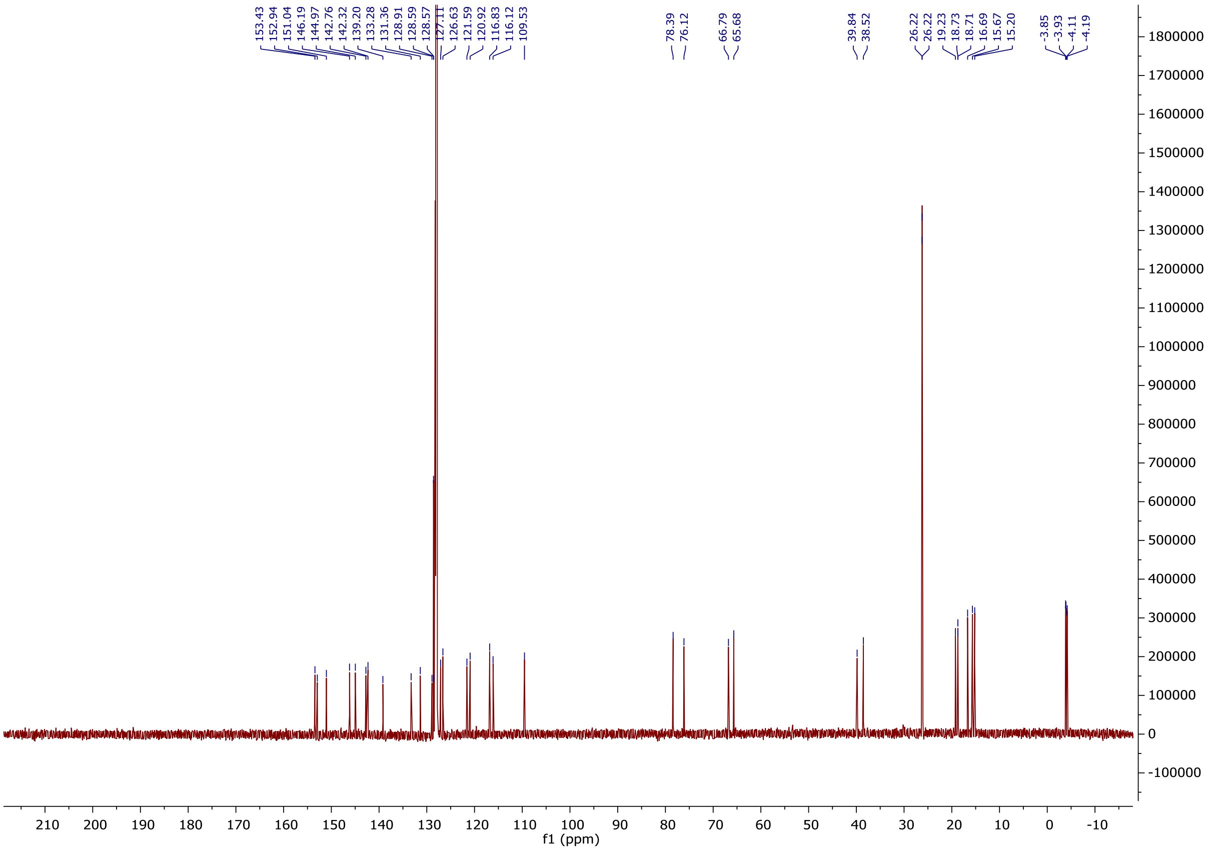

^{1}H and ^{13}C NMR spectrum for compound (*S*,*R*,*R*)-**S17**

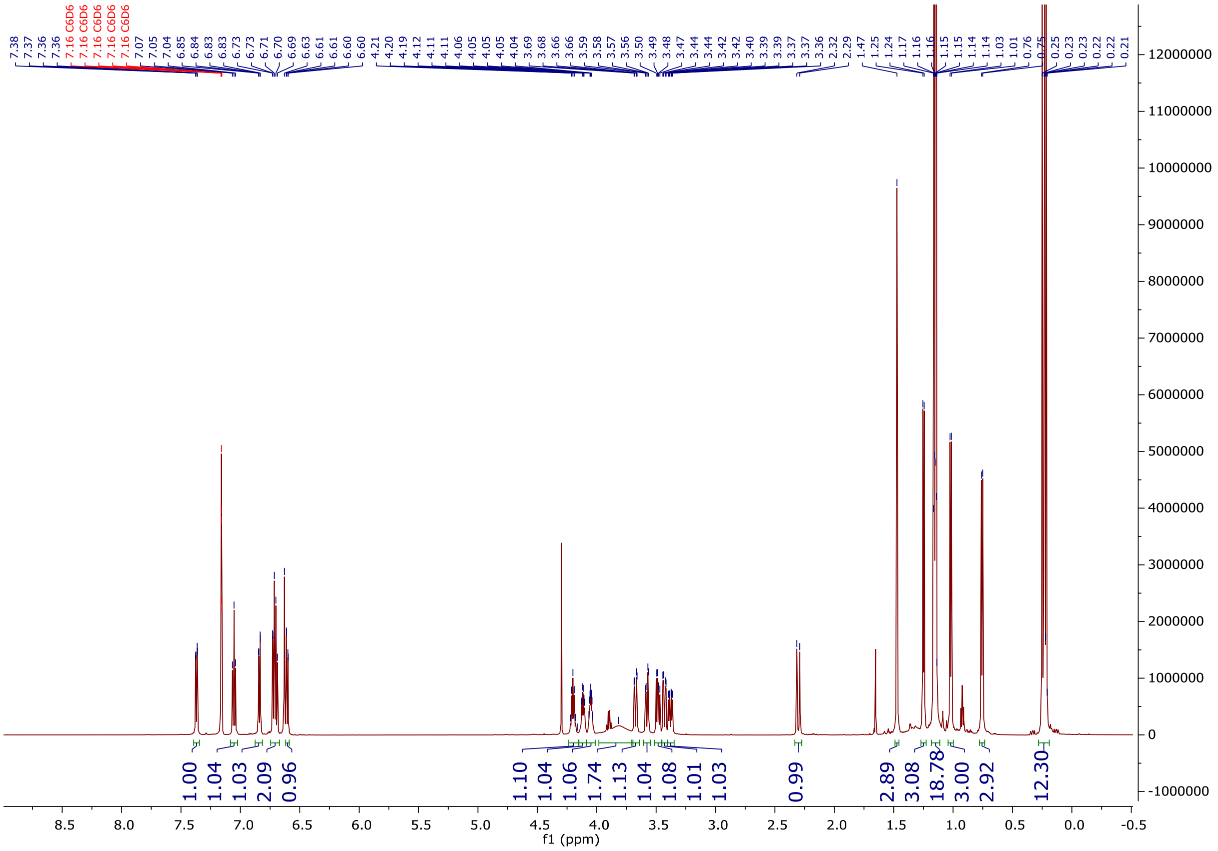

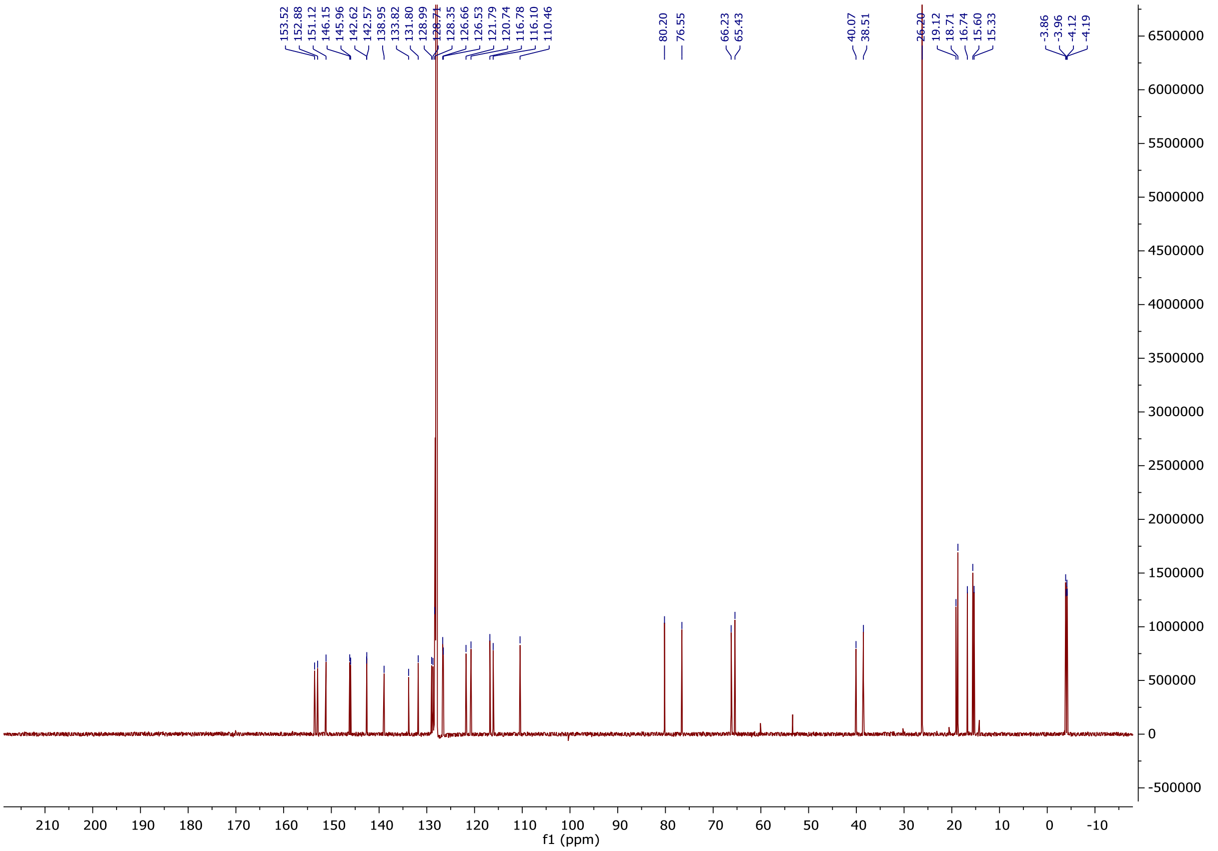

^{1}H and ^{13}C NMR spectra for compound (*R*,*R*,*R*)-**S19**

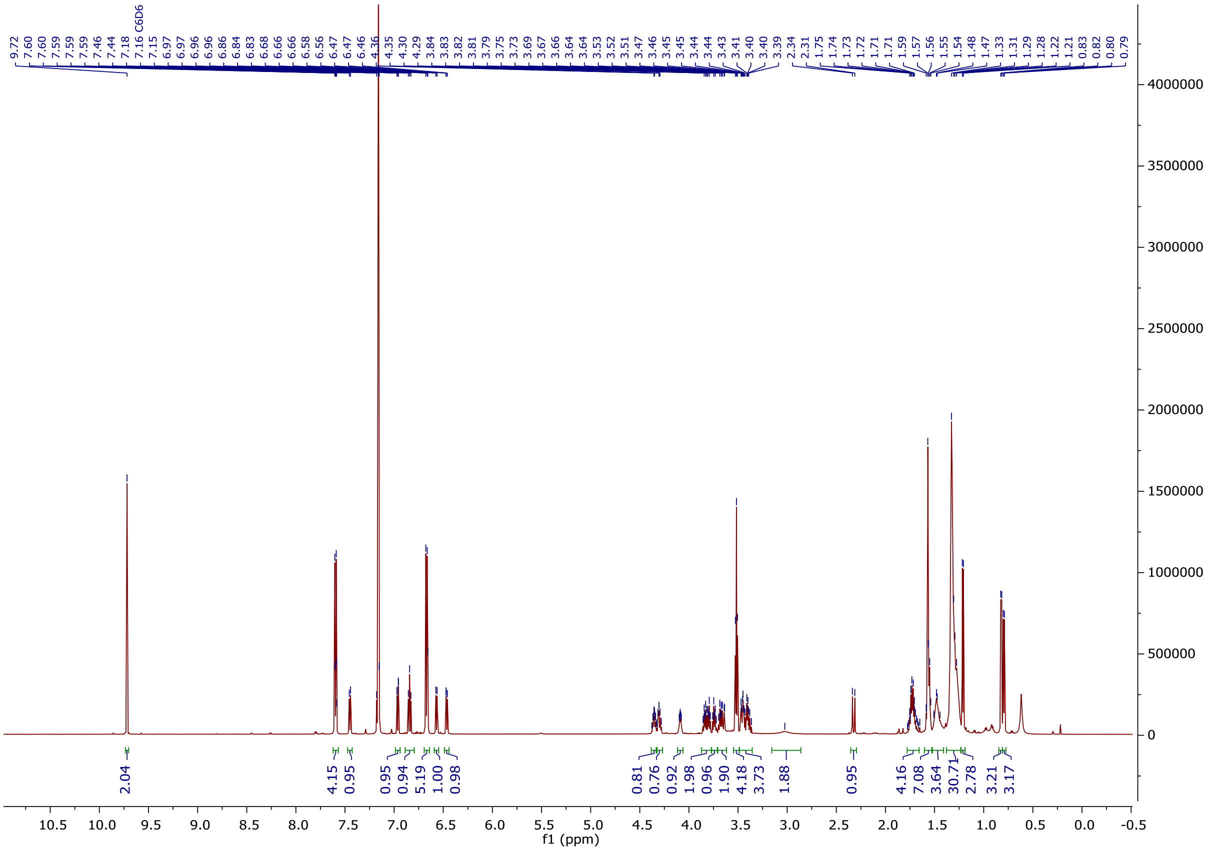

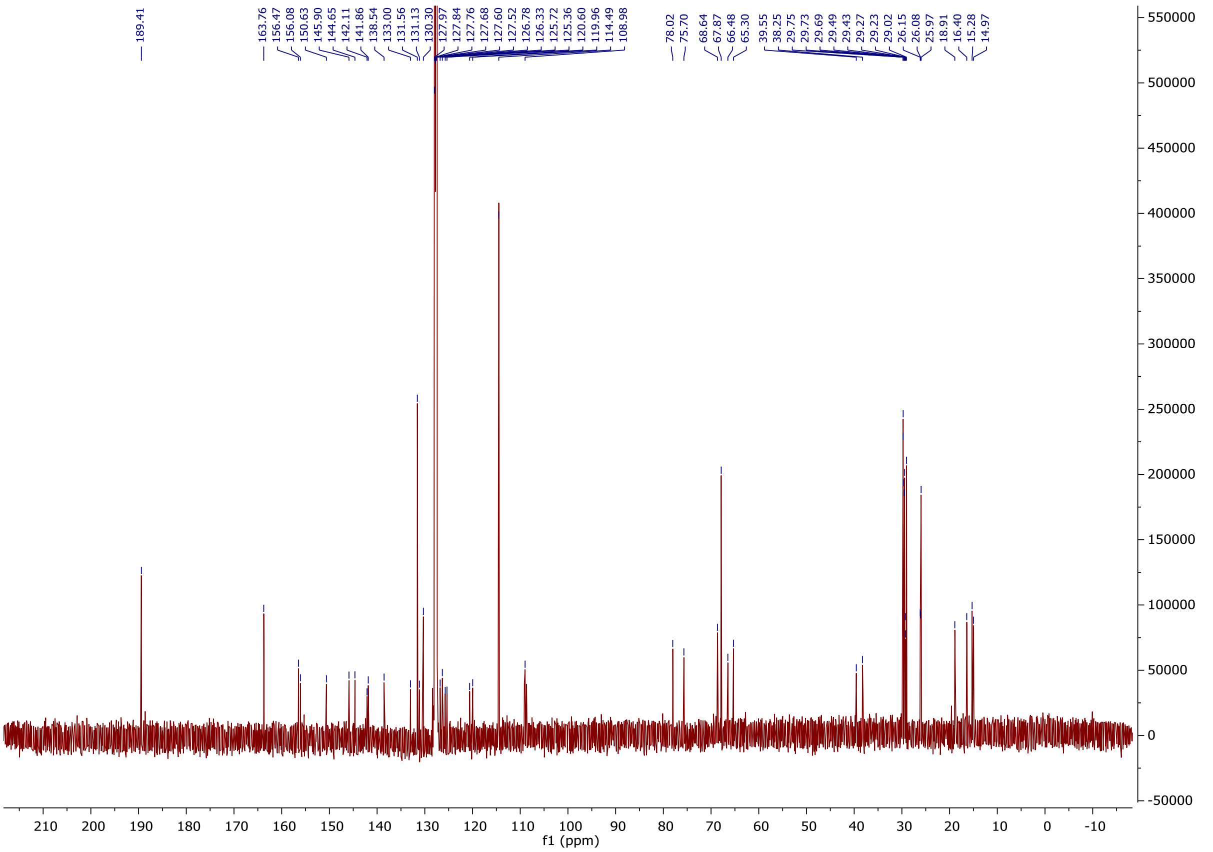

$^1H,^1H$ COSY NMR spectrum for compound (*R*,*R*,*R*)-**S19**

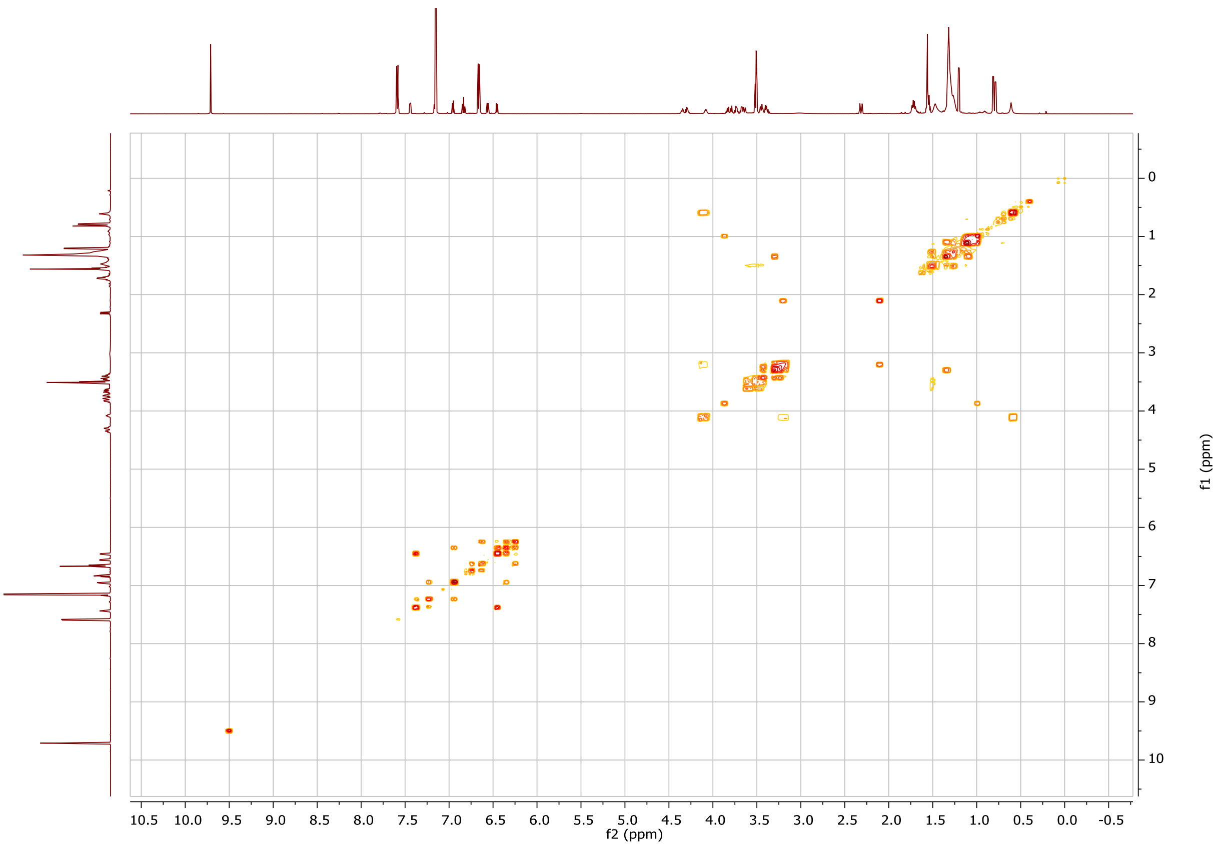

^{1}H and ^{13}C NMR spectrum for compound (*S*,*R*,*R*)-**S19**

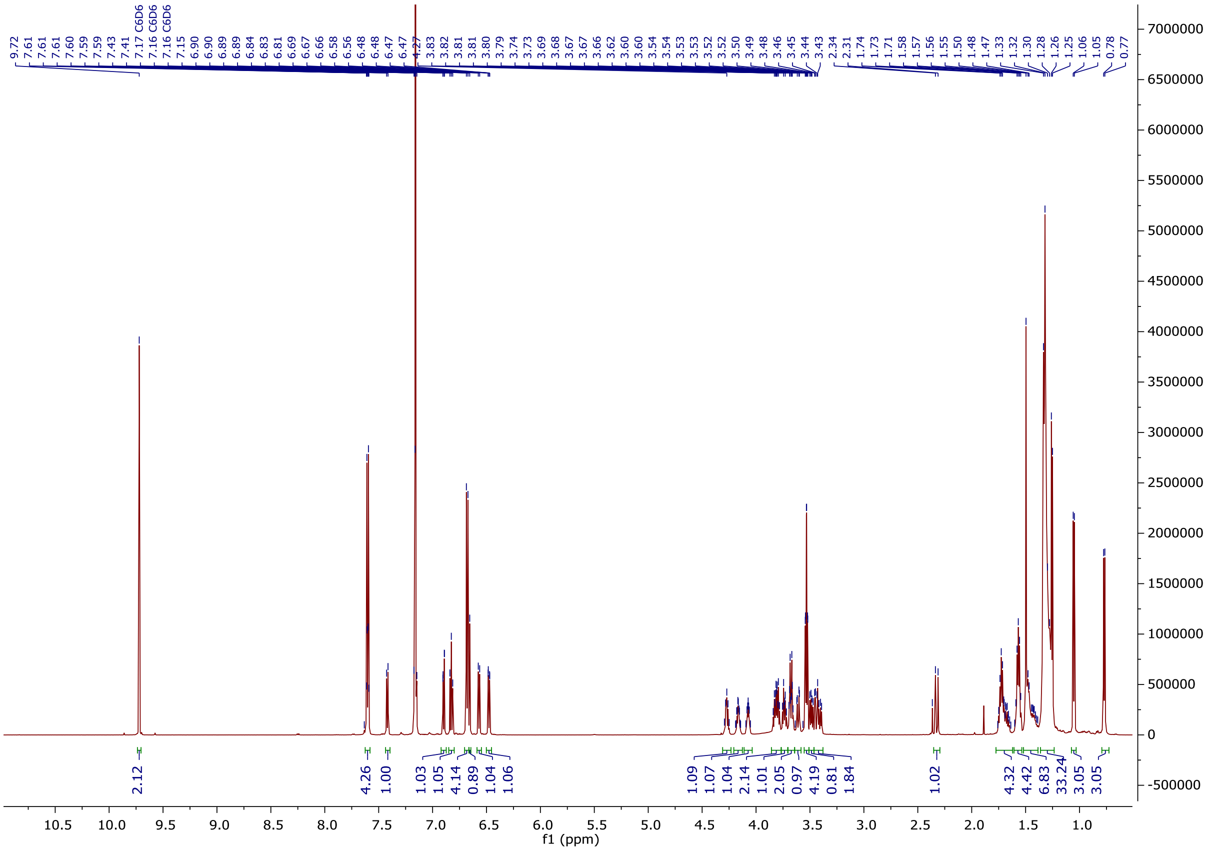

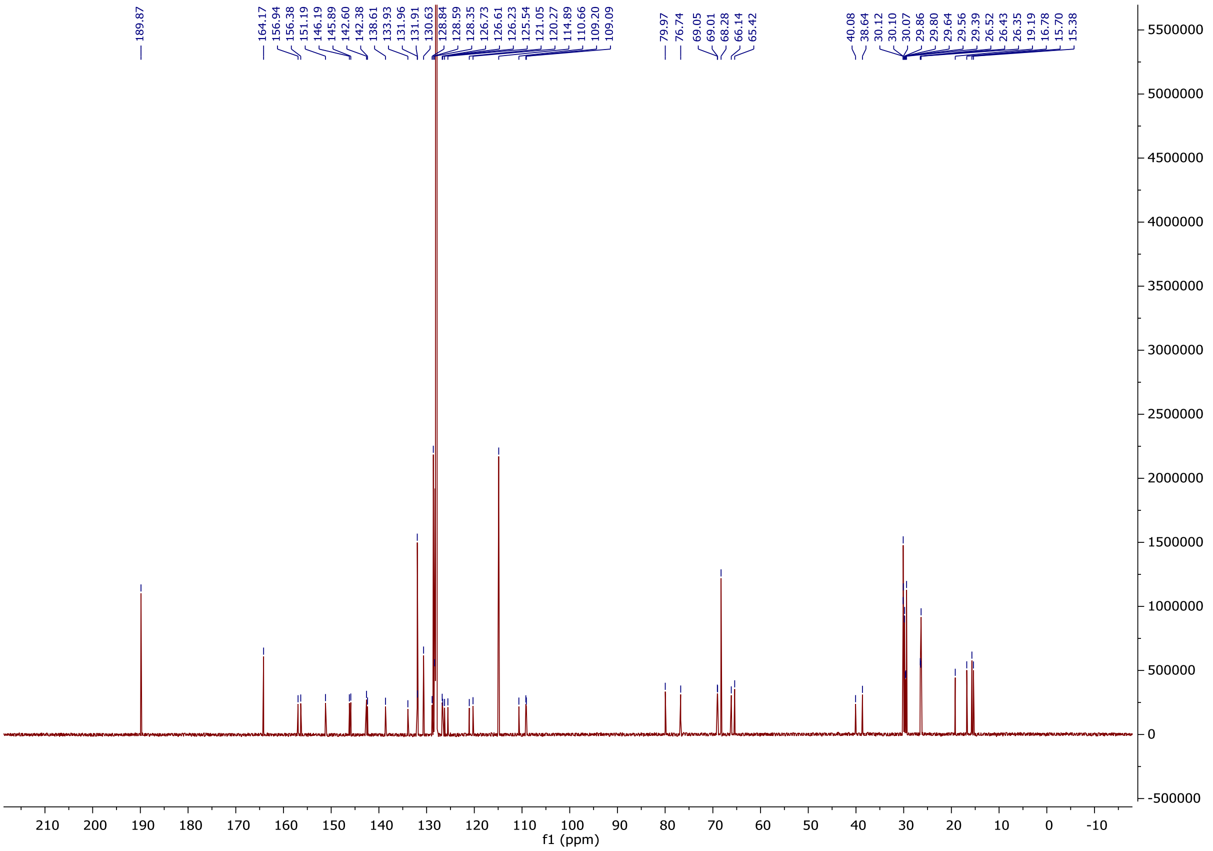

$^{1}H,^{1}H$ COSY NMR spectrum for compound (*S*,*R*,*R*)-**S19**

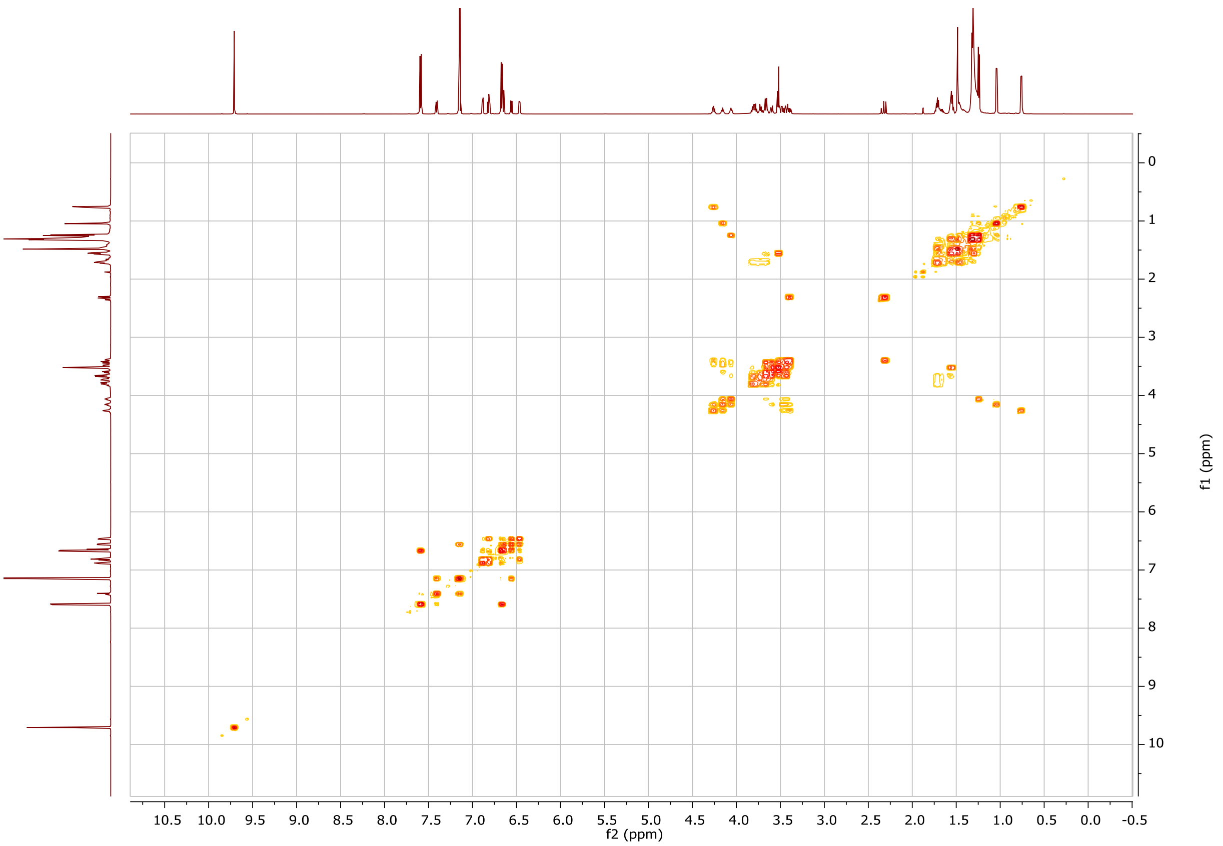

^{1}H and ^{13}C NMR spectra for compound (*R*,*R*,*R*)-**S20**

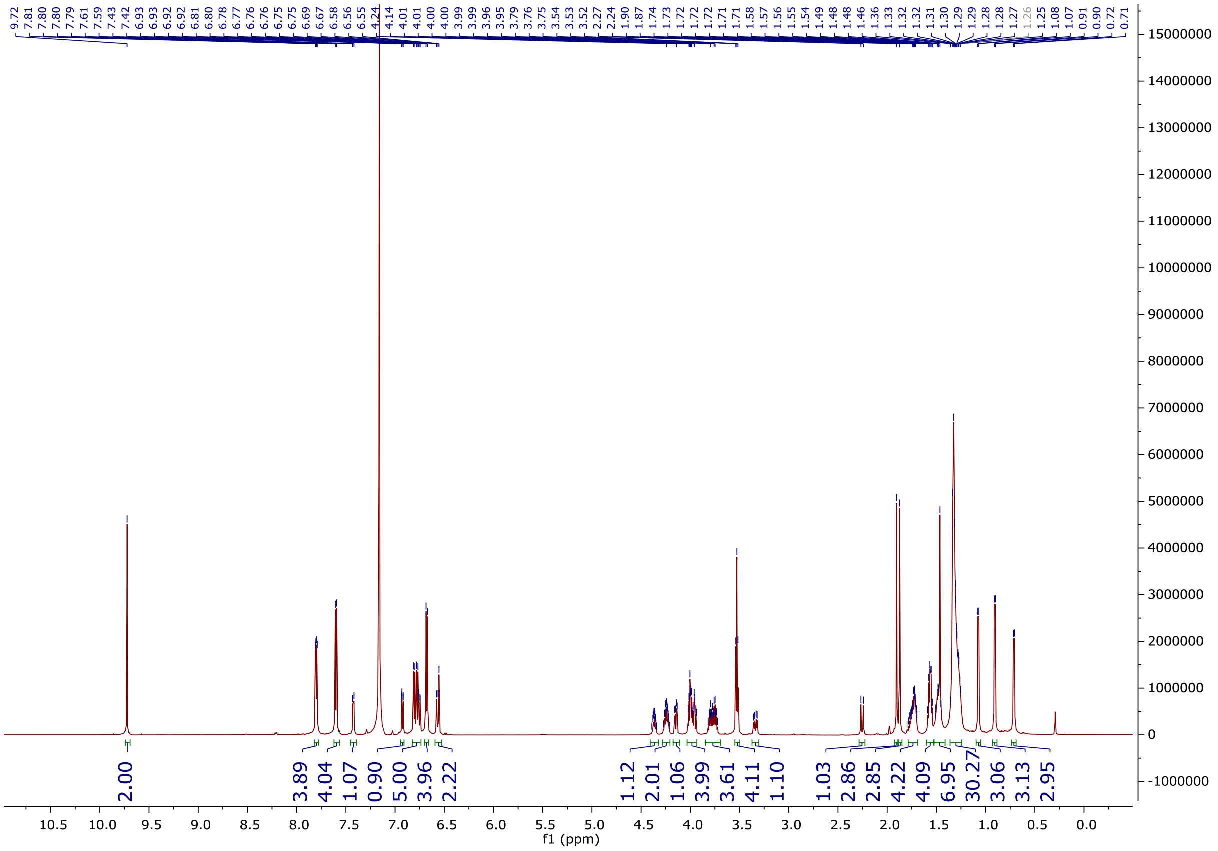

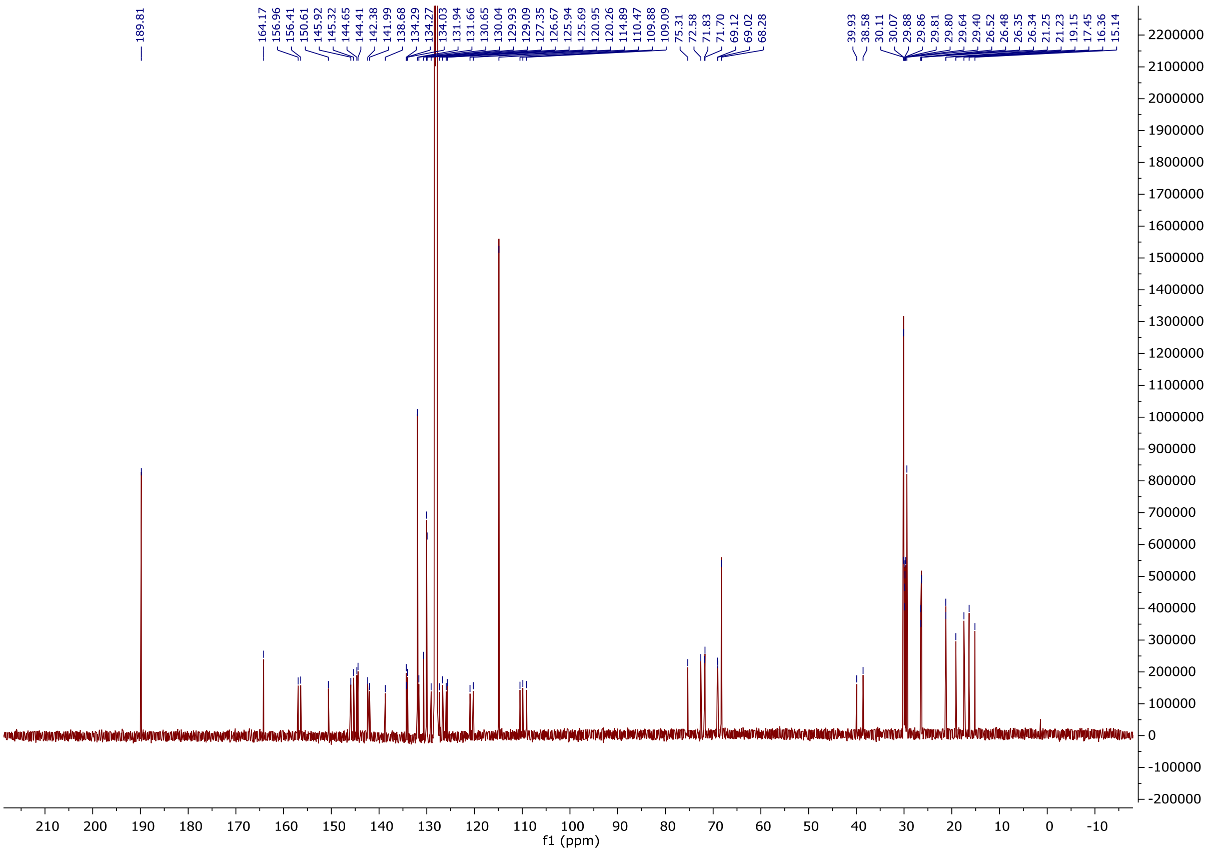

^{1}H,^{1}H COSY NMR spectrum for compound (*R*,*R*,*R*)-**S20**

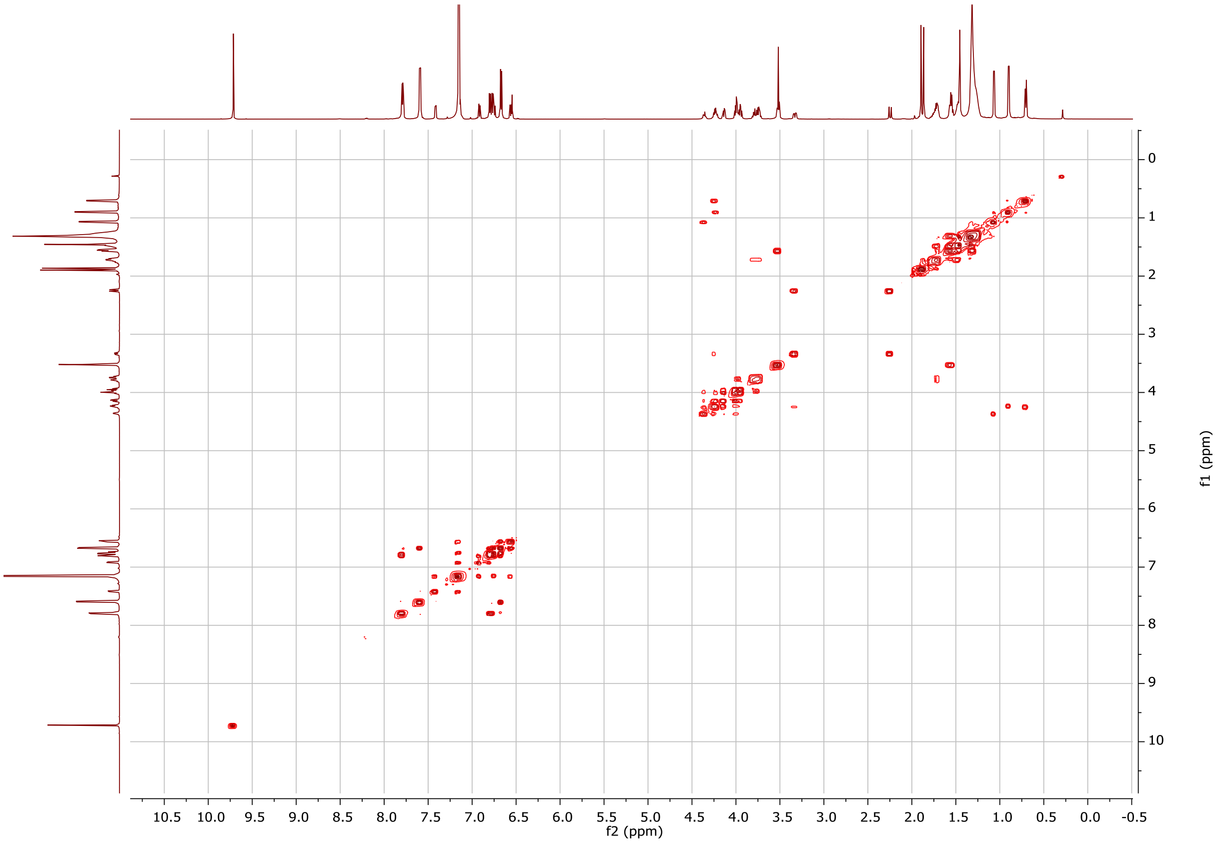

^{1}H and ^{13}C NMR spectra for compound (*S*,*R*,*R*)-**S20**

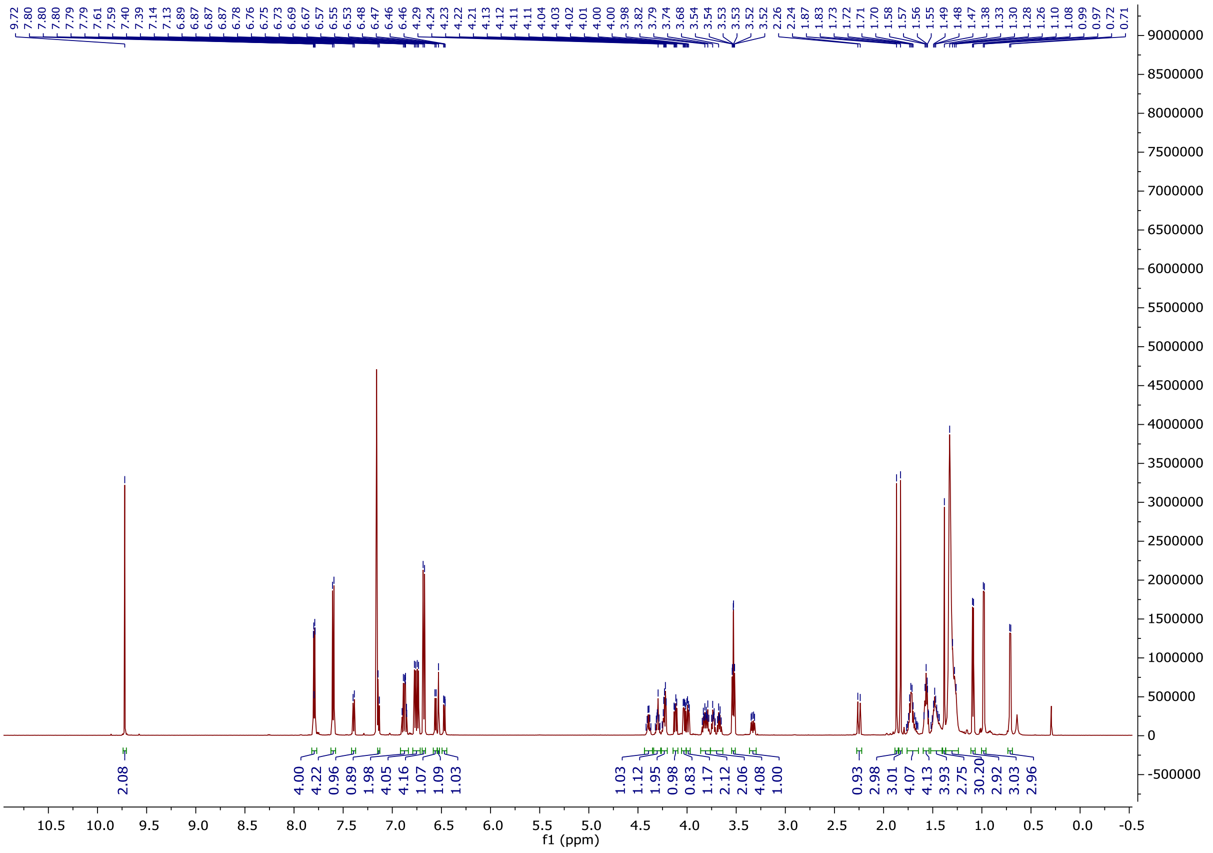

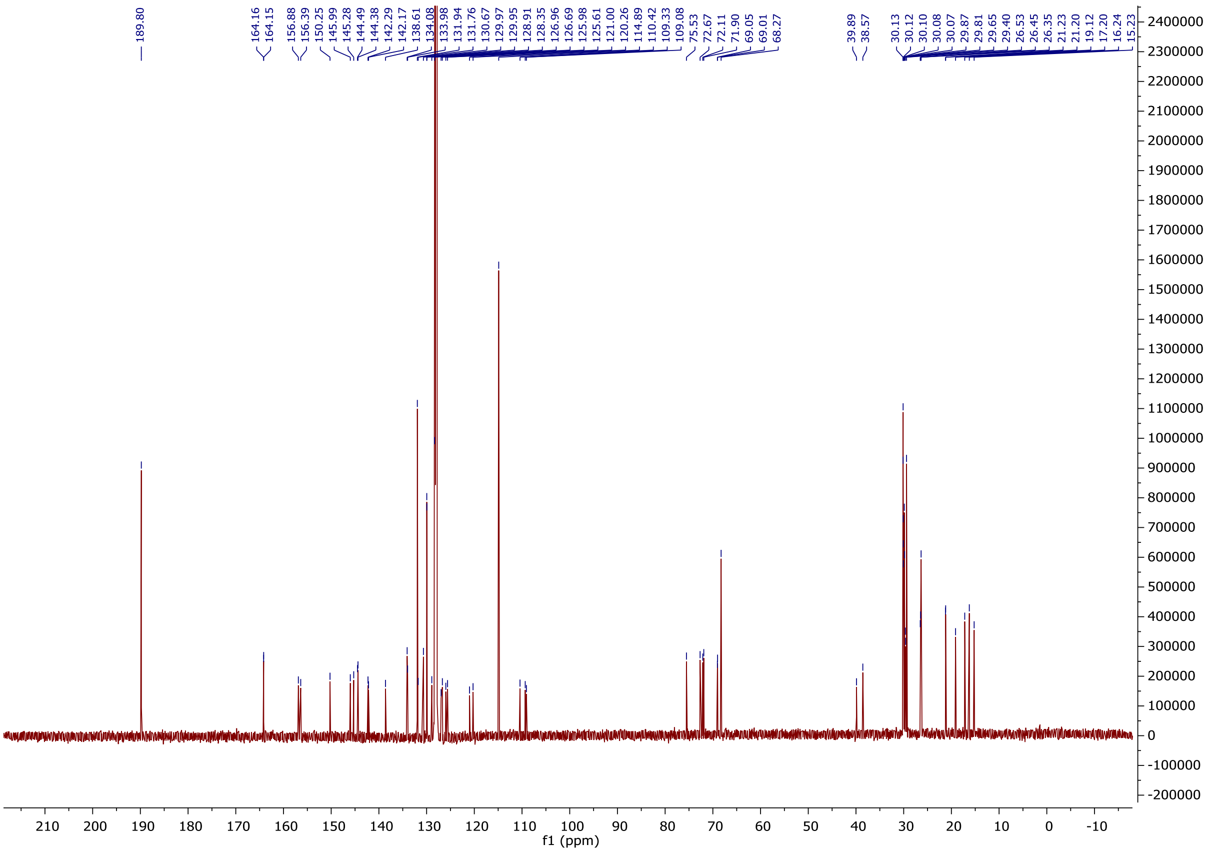

$^{1}H,^{1}H$ COSY NMR spectrum for compound (*S*,*R*,*R*)-**S20**

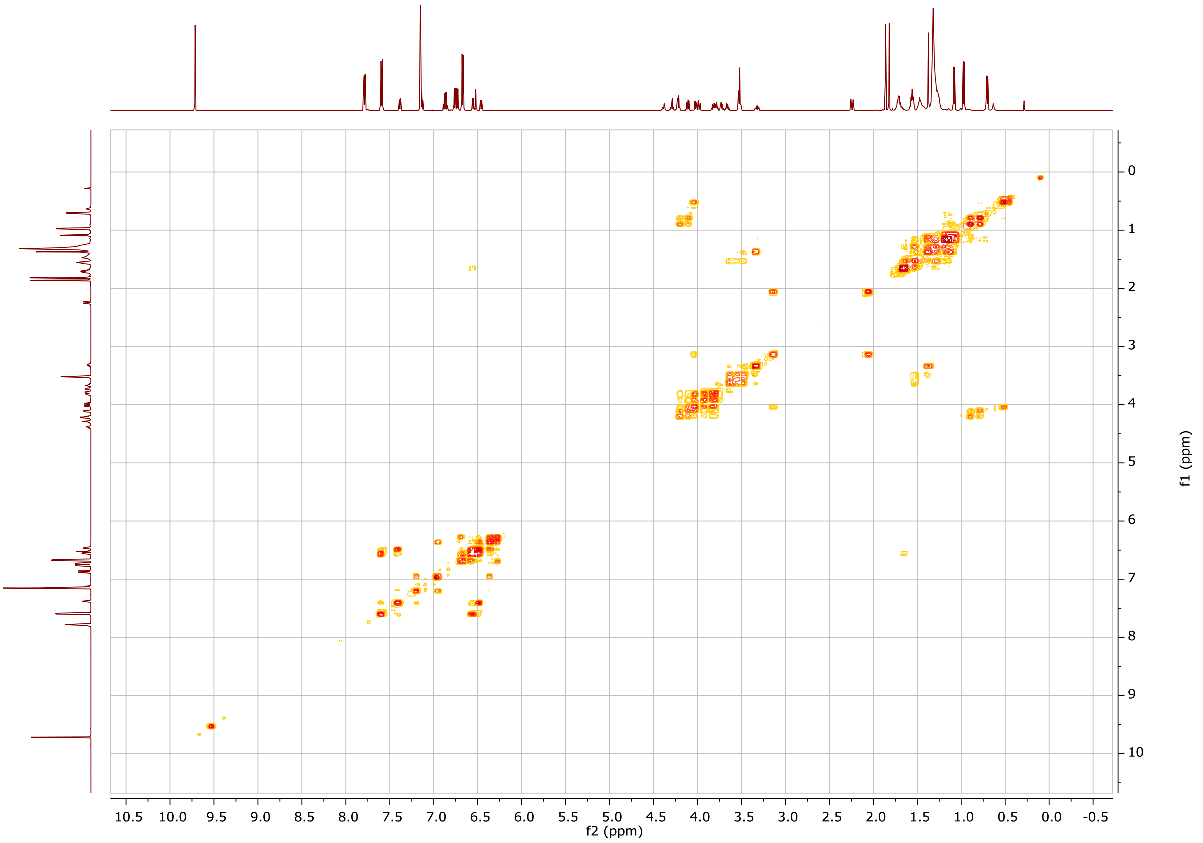

^{1}H and ^{13}C NMR spectra for compound (*R*,*R*,*R*)-**S21**

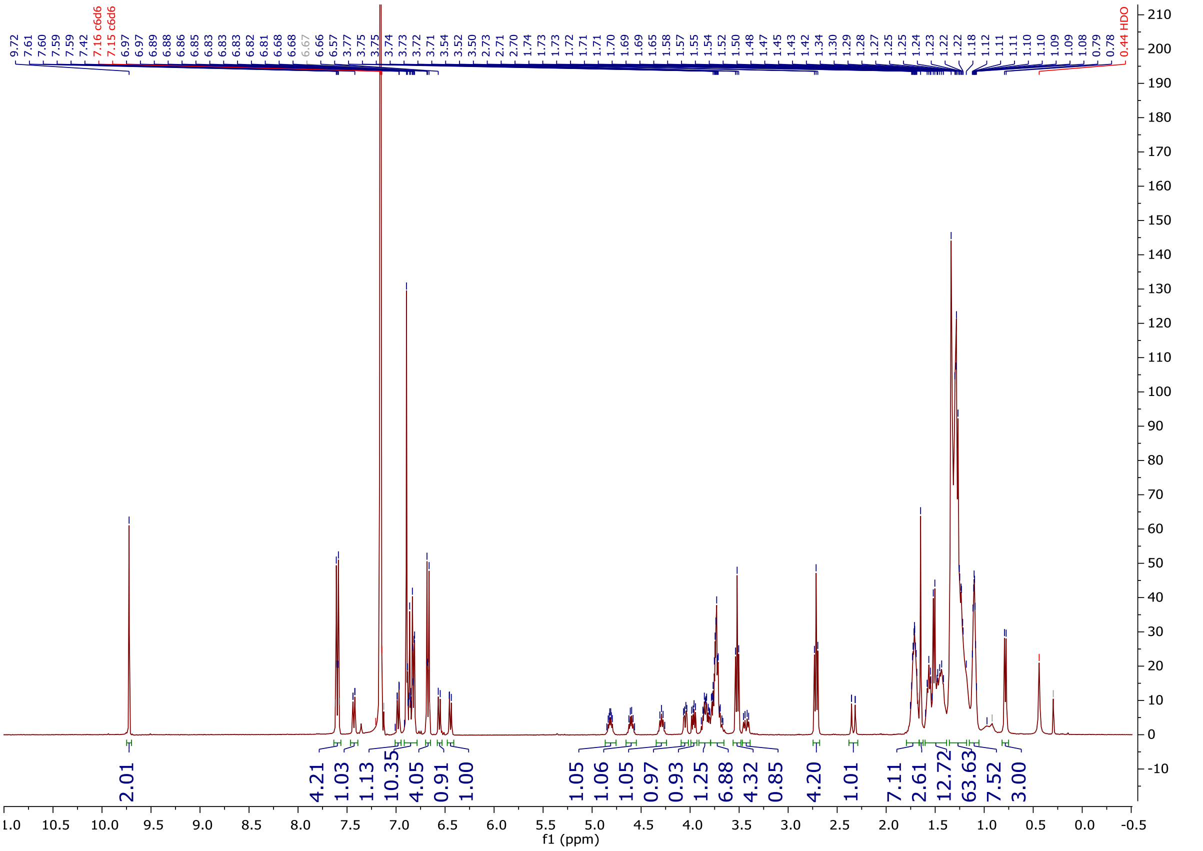

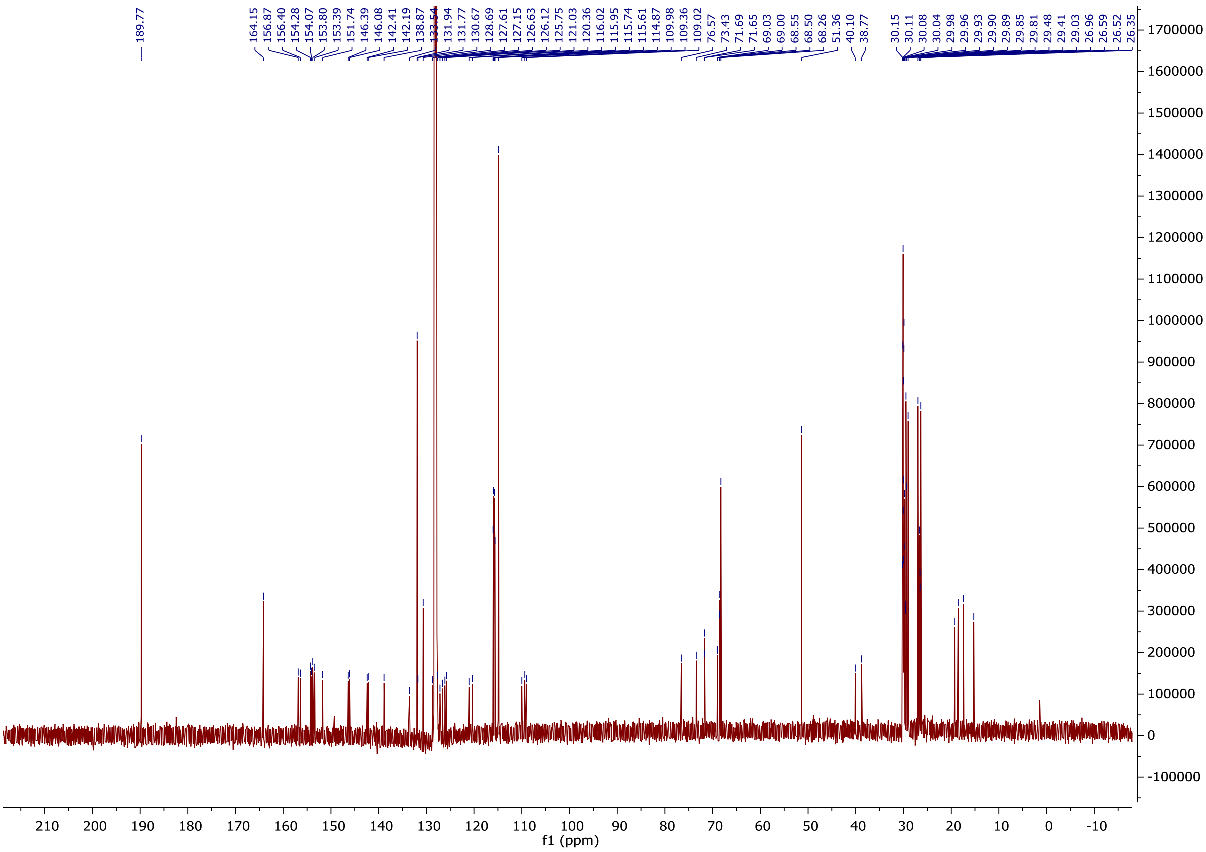

^{1}H,^{1}H COSY NMR spectrum for compound (*R*,*R*,*R*)-**S21**

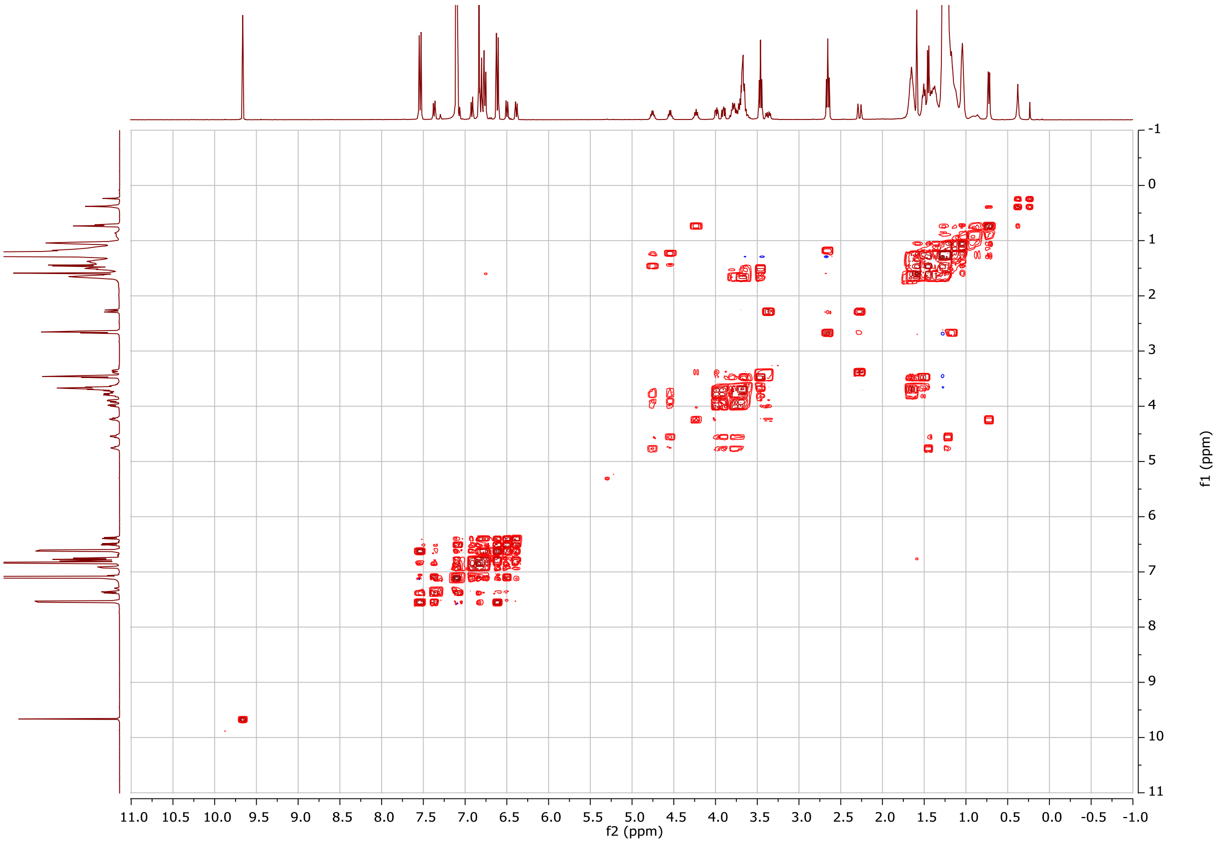

^{1}H and ^{13}C NMR spectra for compound (*S*,*R*,*R*)-**S21**

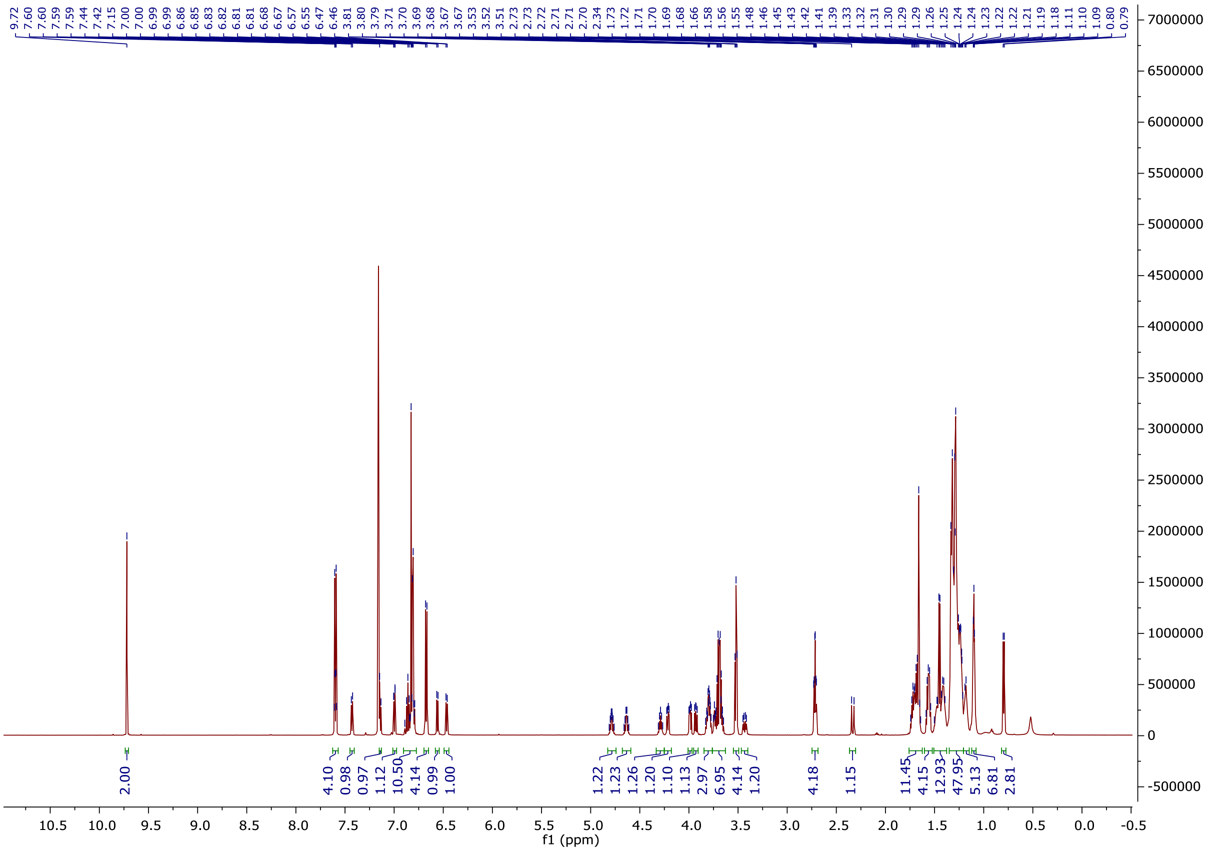

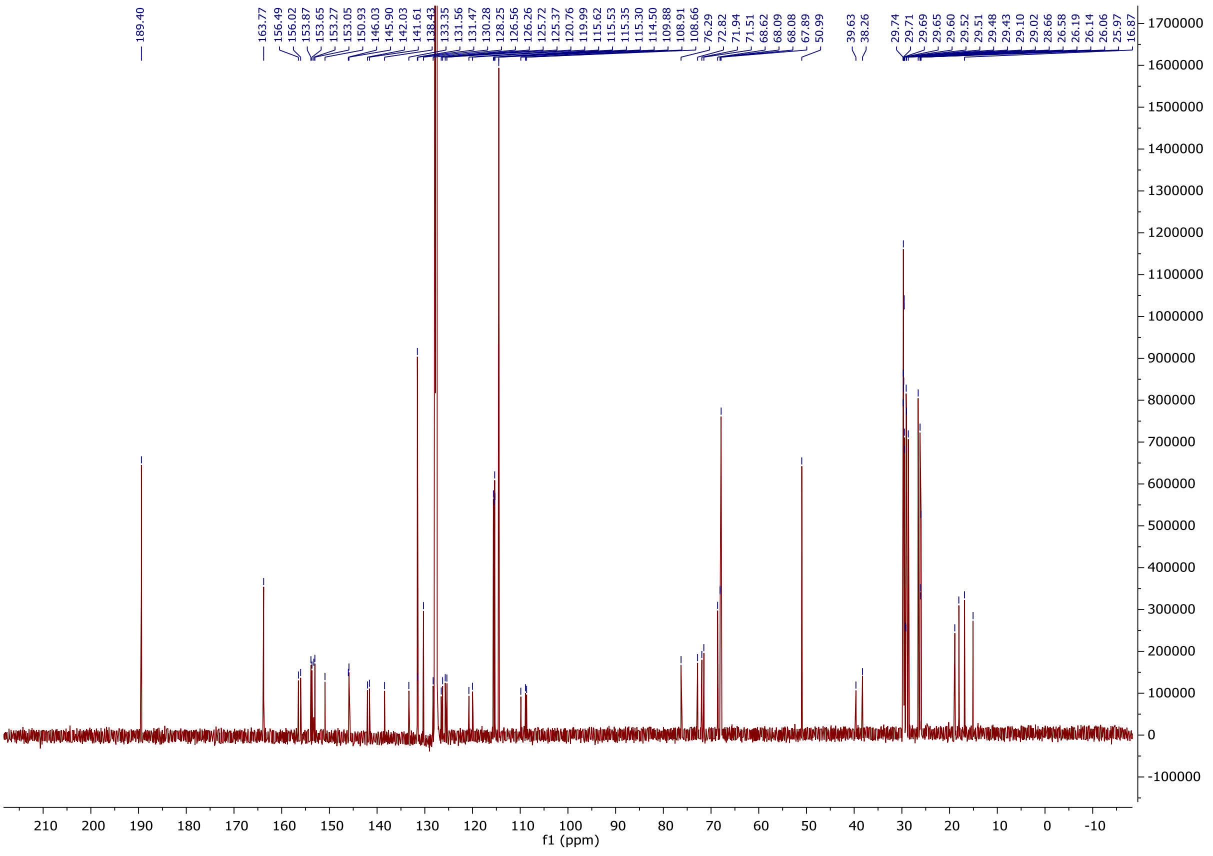

^{1}H,^{1}H COSY NMR spectrum for compound (*S*,*R*,*R*)-**S21**

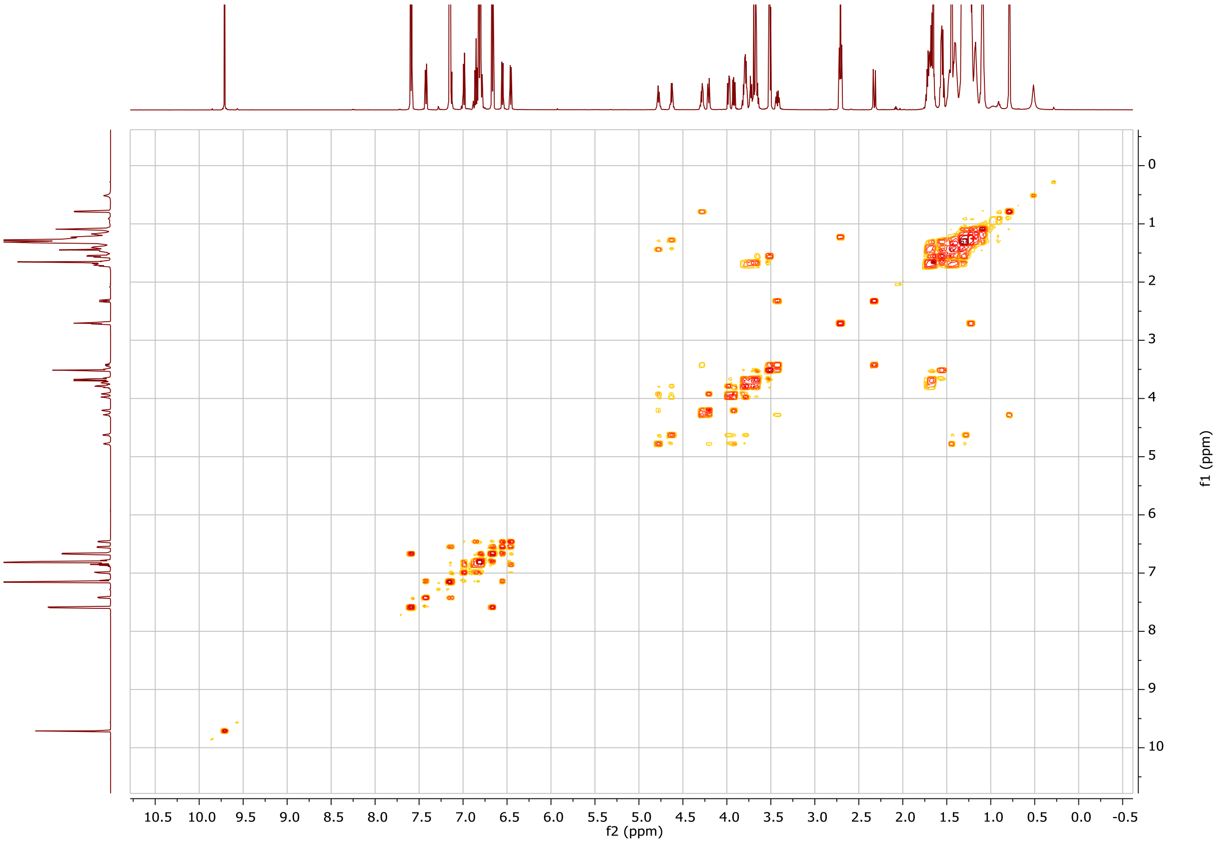

High-resolution ESI+ mass spectra of equilibrated (*S*,*R*,*R*)-**±n** sample

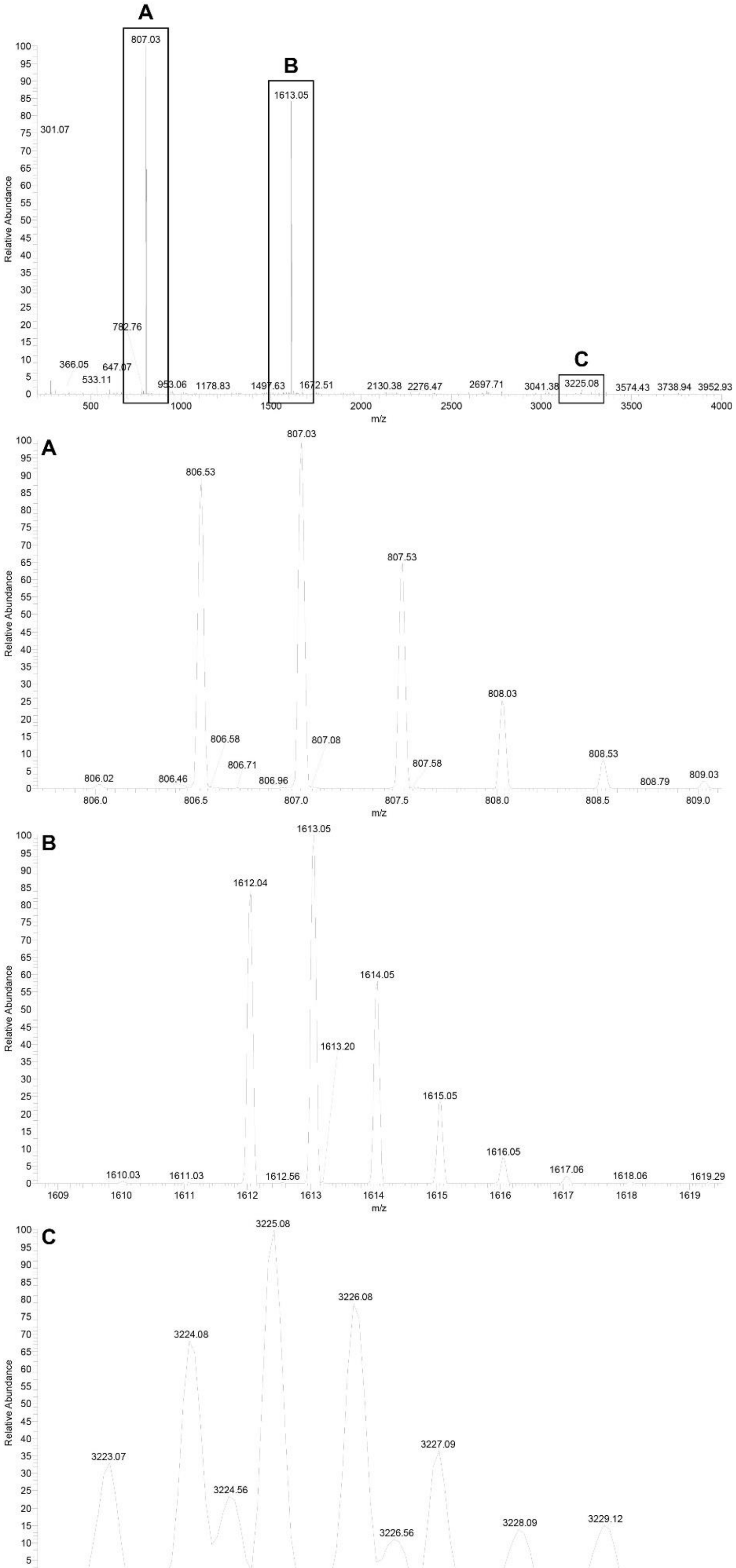

ESI+ mass spectra of equilibrated and illuminated (*S*,*R*,*R*)-**±n** sample

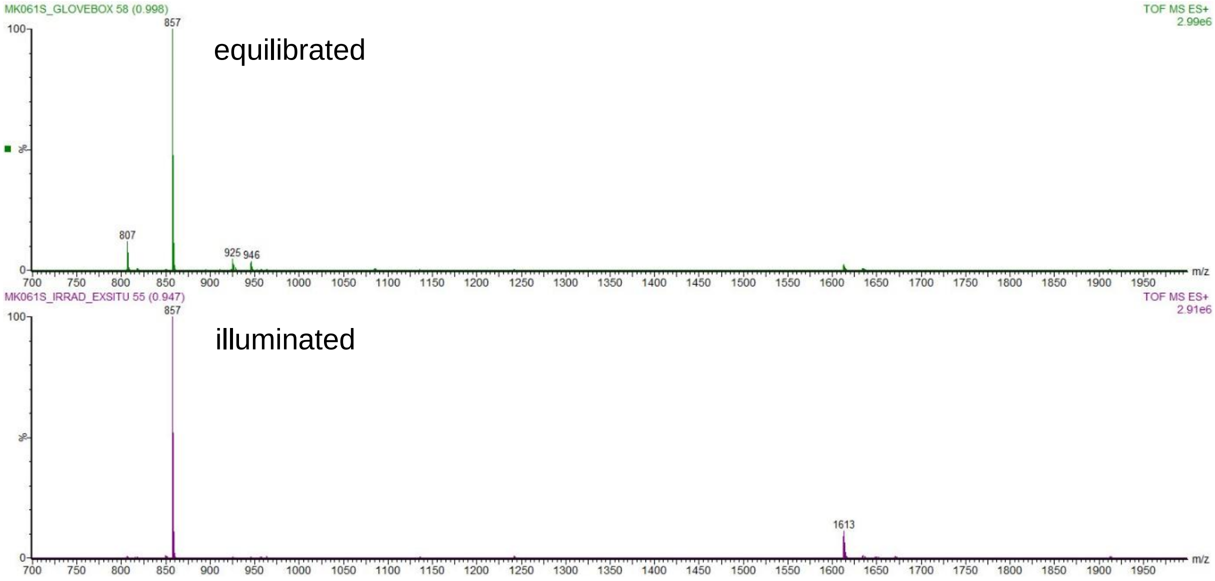

Supplement: Supplementary file 1 — Supplementary figures. [file 41565_2021_1021_MOESM1_ESM.pdf]
