# Supplementary material for: A light-fuelled nanoratchet shifts a coupled chemical equilibrium
Source: Nat Nanotechnol. 2021 Dec 16;17(2):159–65. doi: 10.1038/s41565-021-01021-z (PMC8956507; doi:10.1038/s41565-021-01021-z)
Supplement: Supplementary file 2 — Supplementary Data Set 1. Output files for kinetic analysis. [file 41565_2021_1021_MOESM2_ESM.zip › Supplementary_Data_Set_1_COPASI_Output/README.pdf]

For parameter estimation, COPASI minimizes an objective function that measures the distance between the model and the experimental data, i.e. a residual sum of squares (RSS).
For a set of parameters P, the following weighted objective function is minimized:

$$RSS(P) = \sum_{i=1}^{n} w_i(x_i - y_i(P))^2$$

where $y_i(P)$ are the simulated values for the experimental ones (x_i) and w_i the weights calculated in our case as a mean square of each species fitted.

For additional information, refer to 10.1093/bioinformatics/btl485.

We report here the objective function for the overall fit (RSS(P)) and the value for each species (e.g. RSS(+1)) of every experiment evaluated with COPASI.

Experiment	RSS(P)	RSS(**-1**)	RSS(**0**)	RSS(**+1**)	RSS(**+2**)	RSS(**+3**)	RSS(**I**)
40deg-0	0.176237			0.026318	0.007779	0.142140	
40deg-1	0.006334			0.003140	0.000180	0.003015	
60deg-0	0.173520	0.053131	0.008680	0.009218	0.102491		
60deg-1	0.055086	0.009965	0.007688	0.006802	0.030631		
cat-0	0.018373	0.002595	0.000873	0.001819	0.002860	0.002517	0.007710
cat-1	0.065148	0.016192	0.013267	0.008601	0.002124	0.002127	0.022837
cat-2	0.033005	0.010531	0.004070	0.003237	0.002102	0.010710	0.002354
QY-0	0.001045	0.000362	0.000219	0.000095	0.000269	0.000099	
QY-1	0.000901	0.000192	0.000295	0.000071	0.000256	0.000086	
