# Supplementary figures and images for: A light-fuelled nanoratchet shifts a coupled chemical equilibrium
Source: Nat Nanotechnol. 2021 Dec 16;17(2):159–65. doi: 10.1038/s41565-021-01021-z (PMC8956507; doi:10.1038/s41565-021-01021-z)

## Parameter Estimation Result

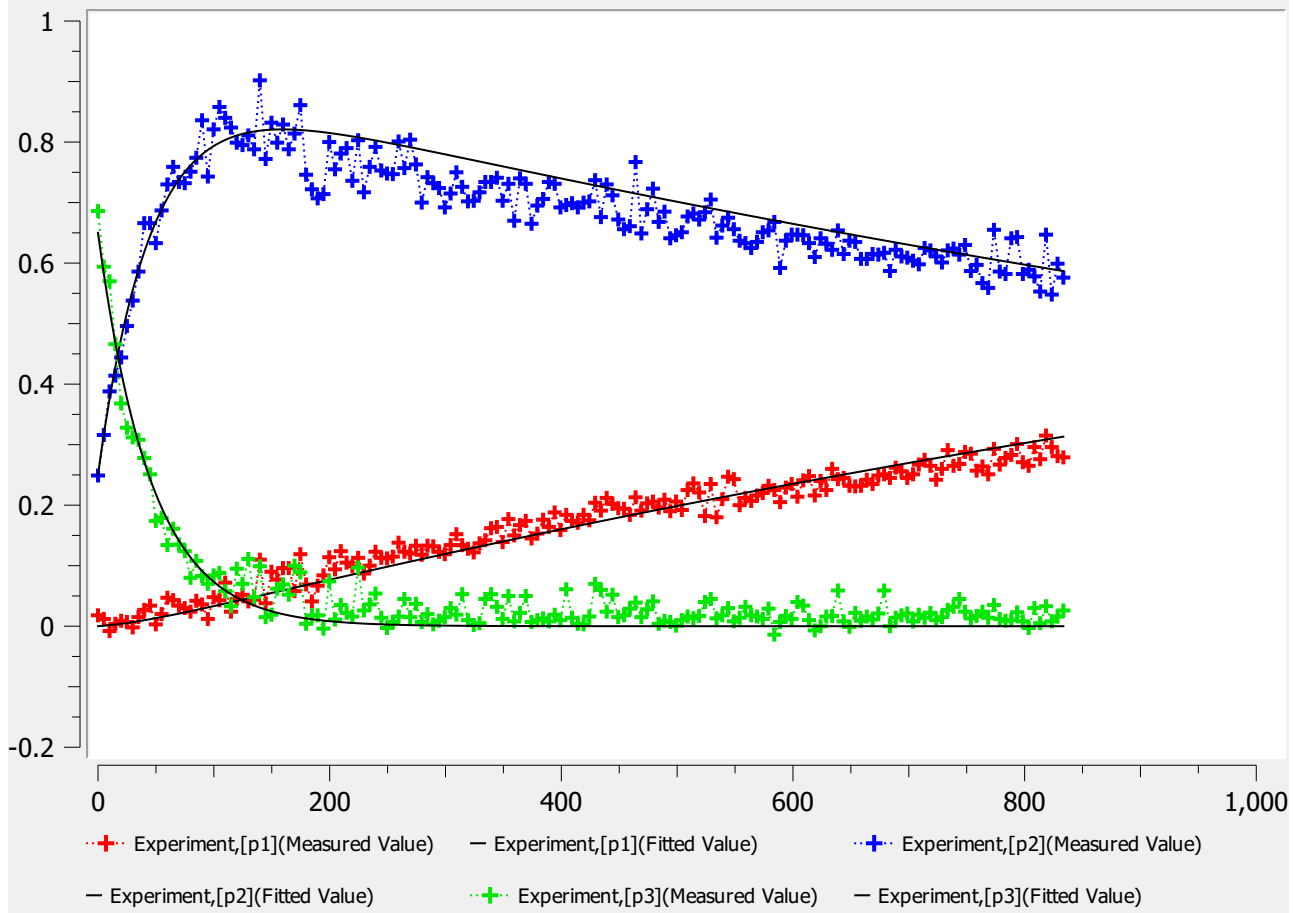

Supplement: Supplementary file 2 — Supplementary Data Set 1. Output files for kinetic analysis. [file 41565_2021_1021_MOESM2_ESM.zip › Supplementary_Data_Set_1_COPASI_Output/40deg-0-REPORT.pdf]

Parameter Estimation Result 1

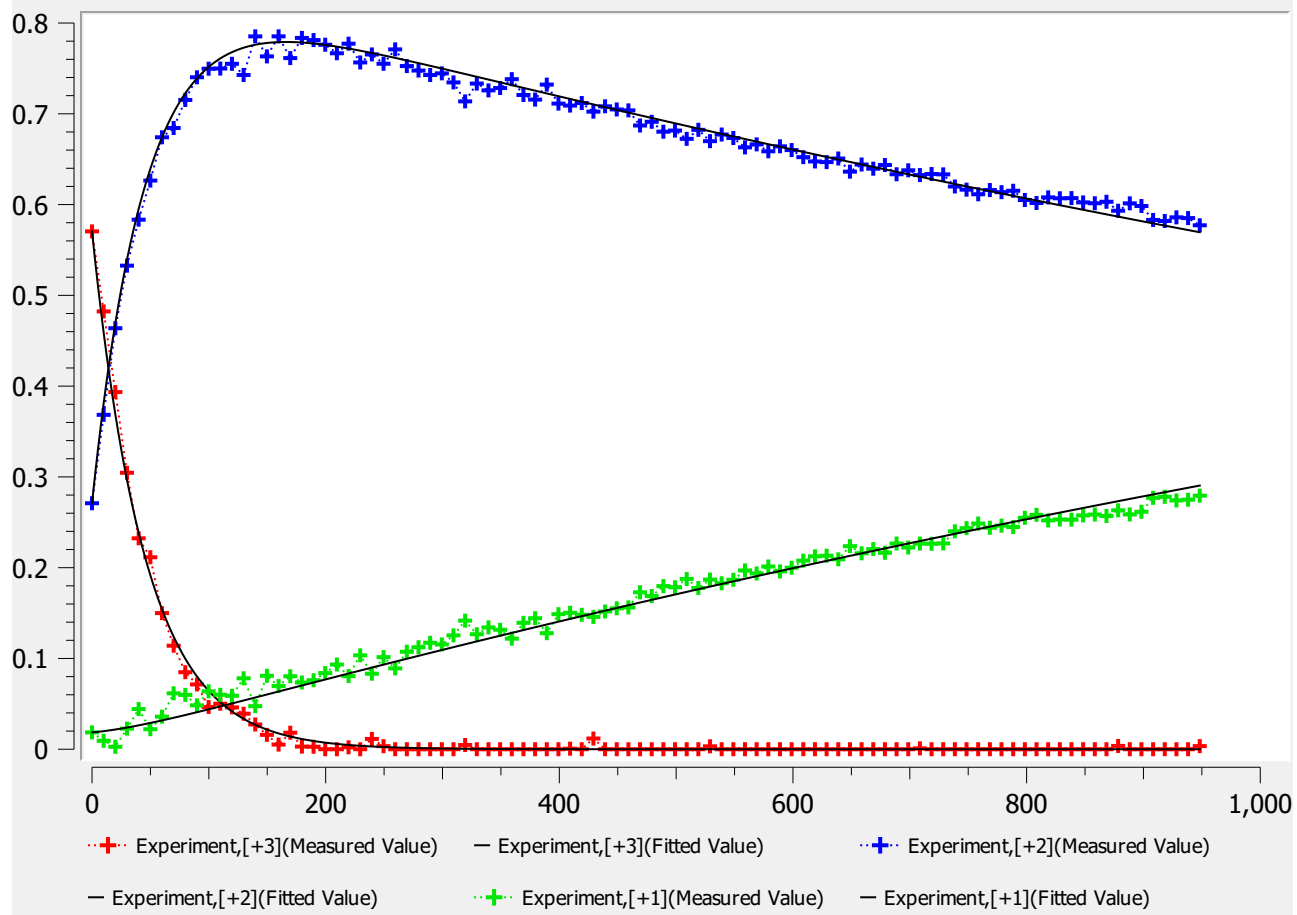

Supplement: Supplementary file 2 — Supplementary Data Set 1. Output files for kinetic analysis. [file 41565_2021_1021_MOESM2_ESM.zip › Supplementary_Data_Set_1_COPASI_Output/40deg-1-REPORT.pdf]

## Parameter Estimation Result

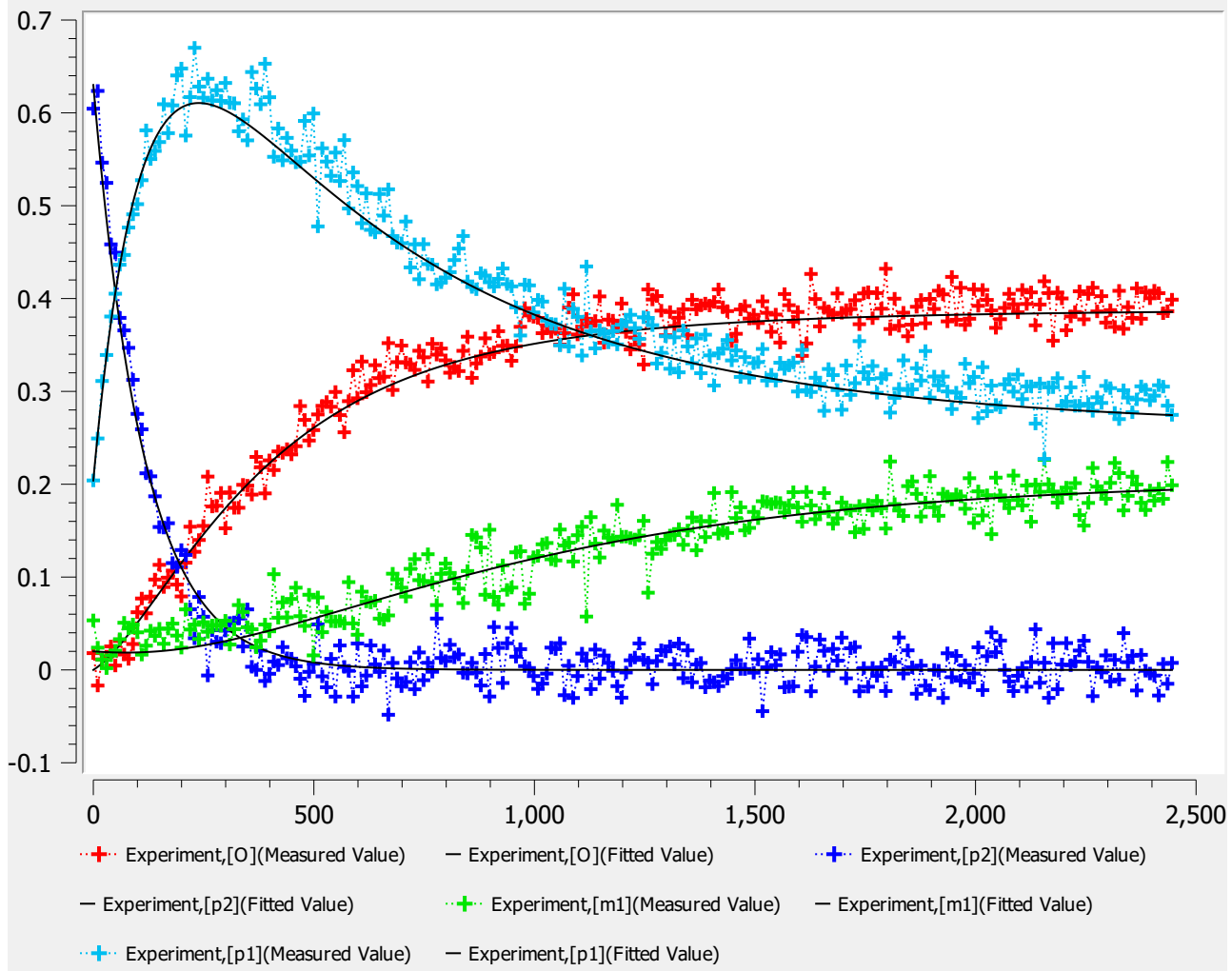

Supplement: Supplementary file 2 — Supplementary Data Set 1. Output files for kinetic analysis. [file 41565_2021_1021_MOESM2_ESM.zip › Supplementary_Data_Set_1_COPASI_Output/60deg-0-REPORT.pdf]

## Parameter Estimation Result

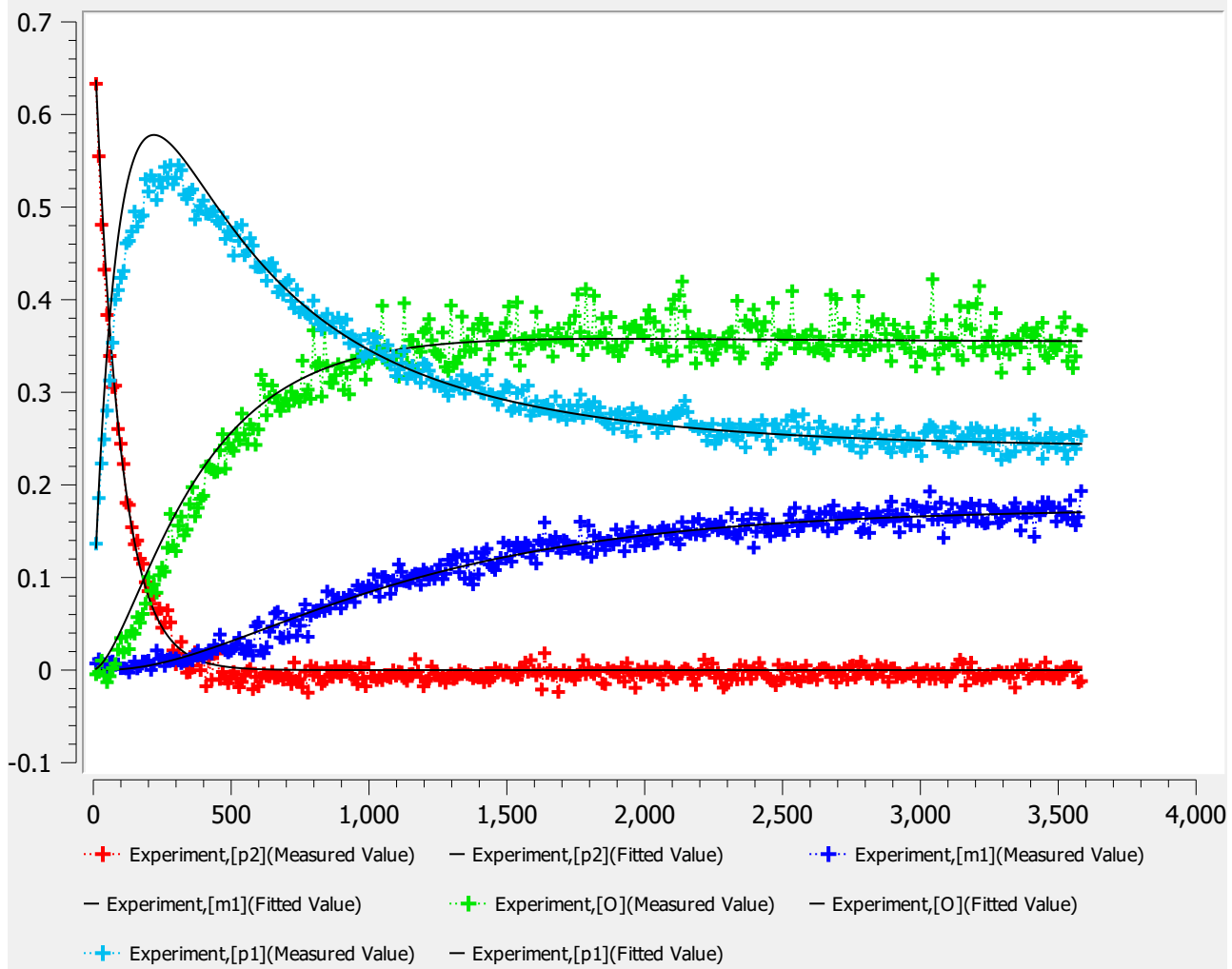

Supplement: Supplementary file 2 — Supplementary Data Set 1. Output files for kinetic analysis. [file 41565_2021_1021_MOESM2_ESM.zip › Supplementary_Data_Set_1_COPASI_Output/60deg-1-REPORT.pdf]

## Parameter Estimation Result

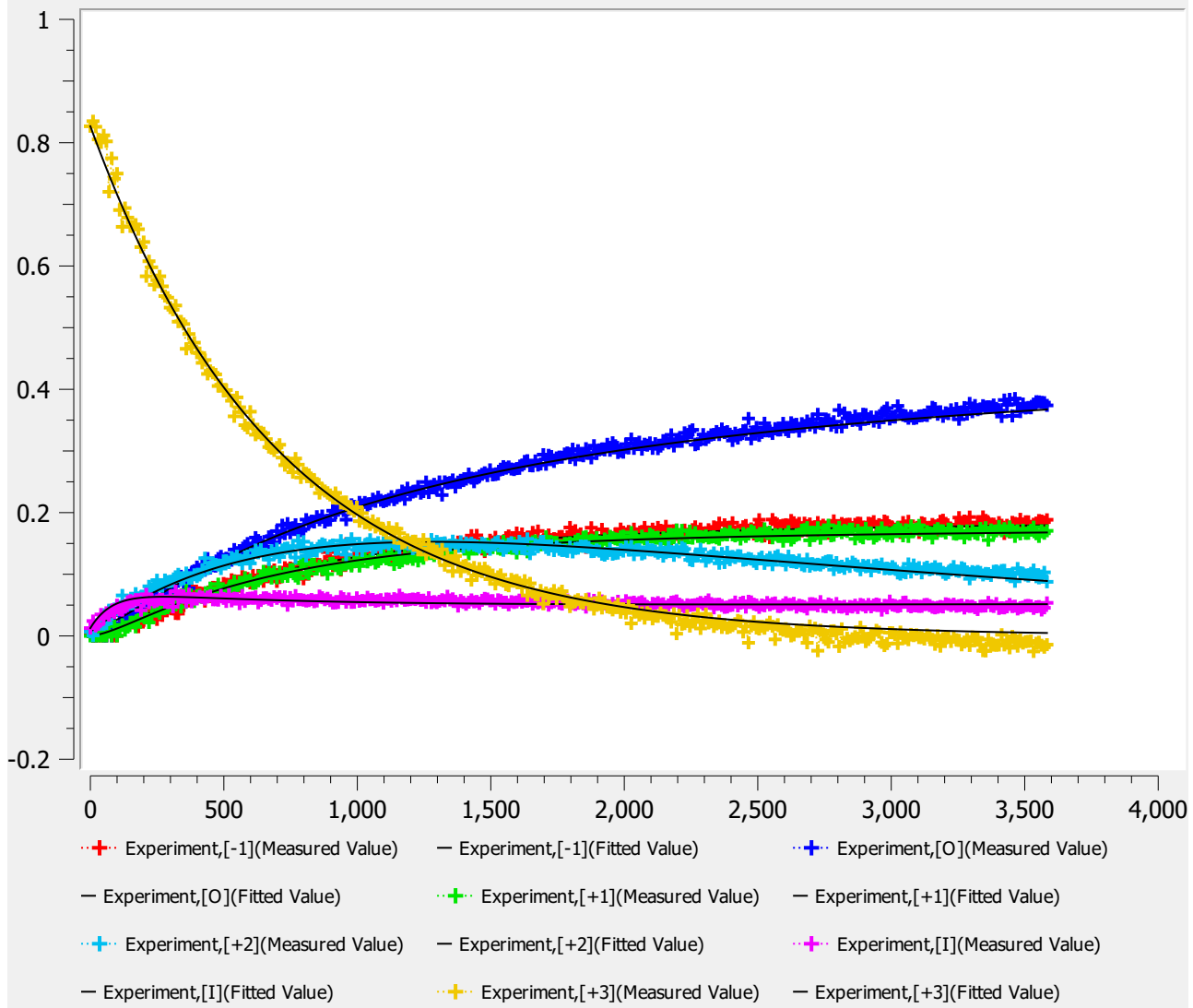

Supplement: Supplementary file 2 — Supplementary Data Set 1. Output files for kinetic analysis. [file 41565_2021_1021_MOESM2_ESM.zip › Supplementary_Data_Set_1_COPASI_Output/cat-0-REPORT.pdf]

## Parameter Estimation Result

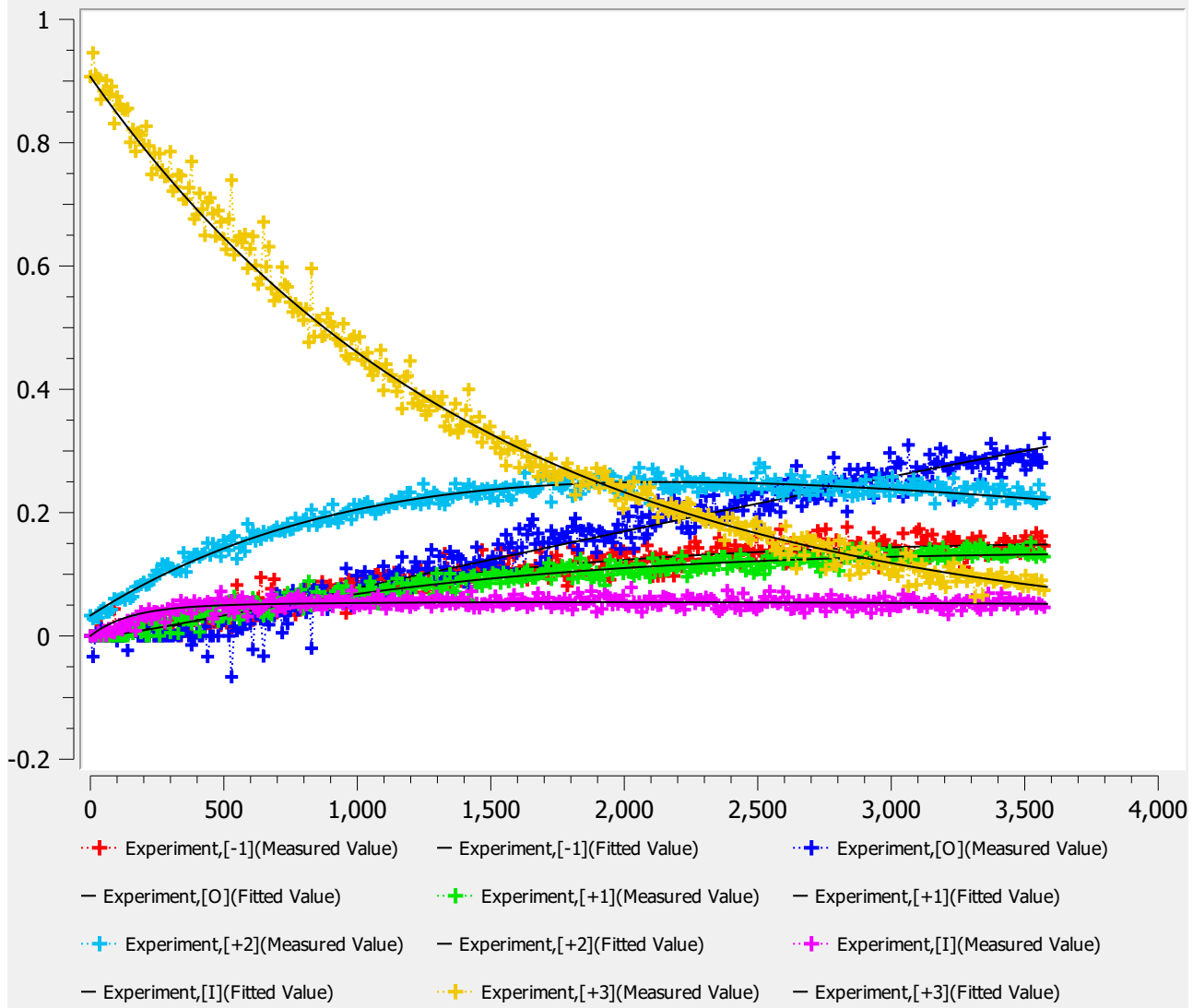

Supplement: Supplementary file 2 — Supplementary Data Set 1. Output files for kinetic analysis. [file 41565_2021_1021_MOESM2_ESM.zip › Supplementary_Data_Set_1_COPASI_Output/cat-1-REPORT.pdf]

## Parameter Estimation Result

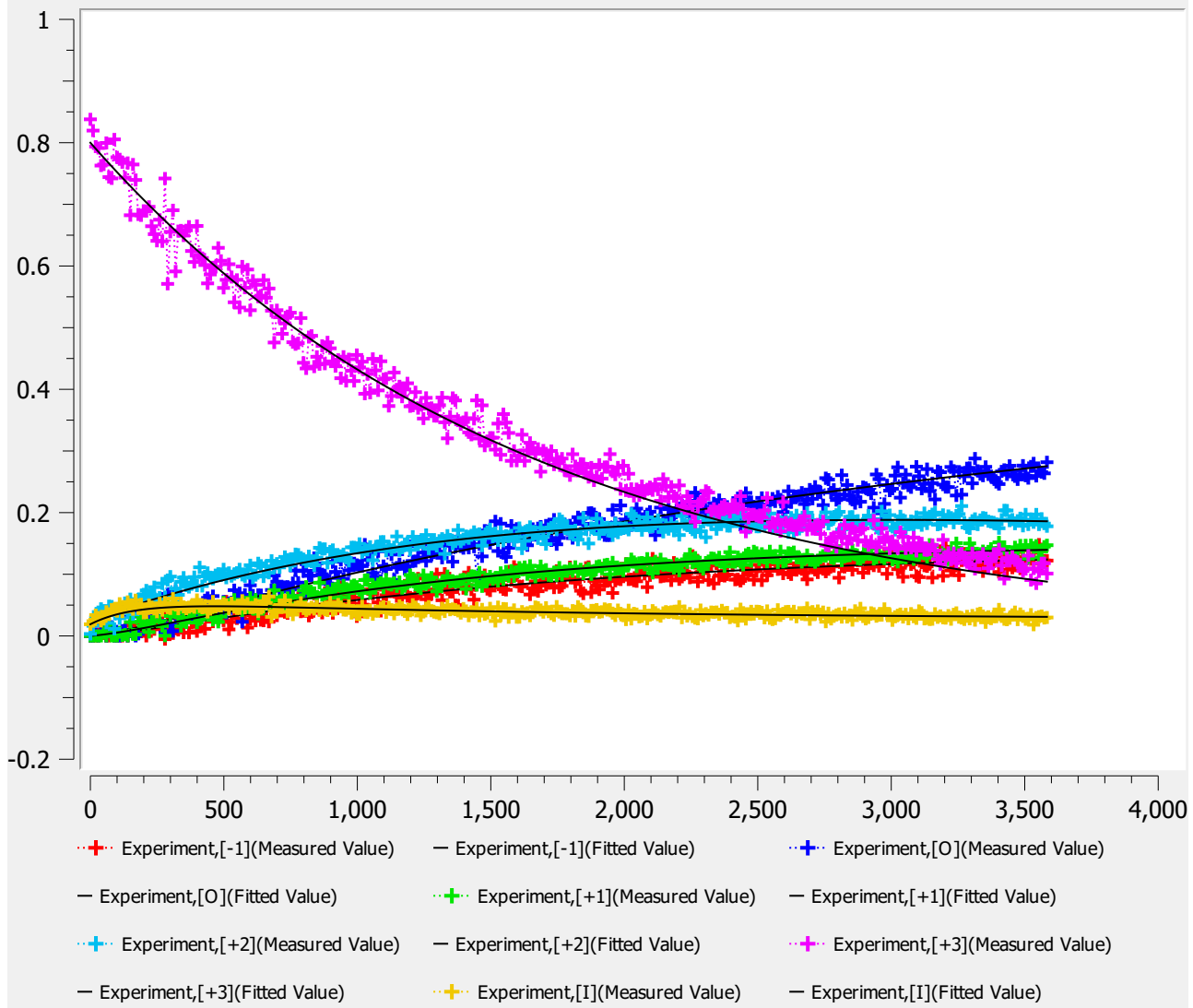

Supplement: Supplementary file 2 — Supplementary Data Set 1. Output files for kinetic analysis. [file 41565_2021_1021_MOESM2_ESM.zip › Supplementary_Data_Set_1_COPASI_Output/cat-2-REPORT.pdf]

# Parameter Estimation Result 1

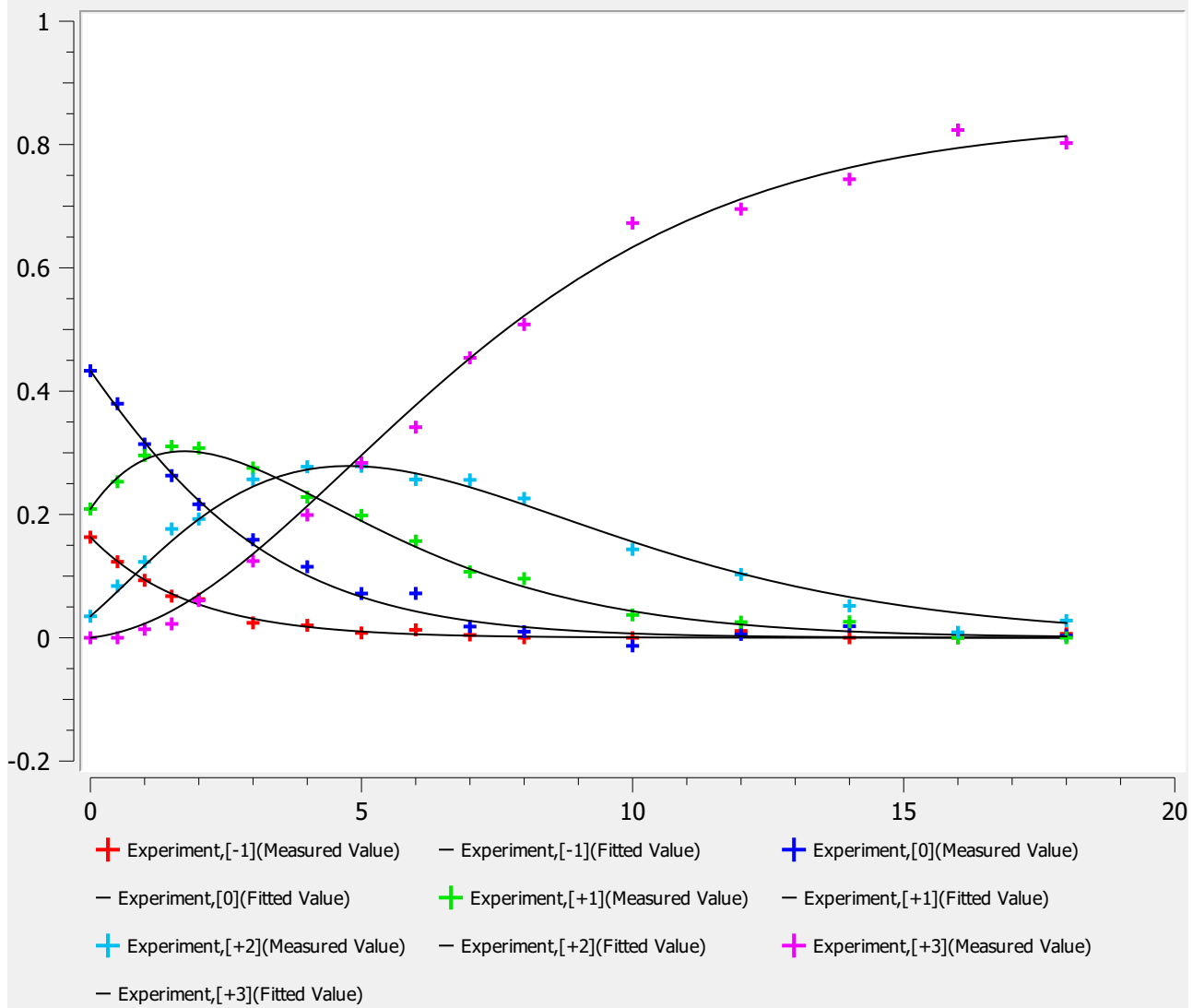

Supplement: Supplementary file 2 — Supplementary Data Set 1. Output files for kinetic analysis. [file 41565_2021_1021_MOESM2_ESM.zip › Supplementary_Data_Set_1_COPASI_Output/QY-0-REPORT.pdf]

# Parameter Estimation Result 1

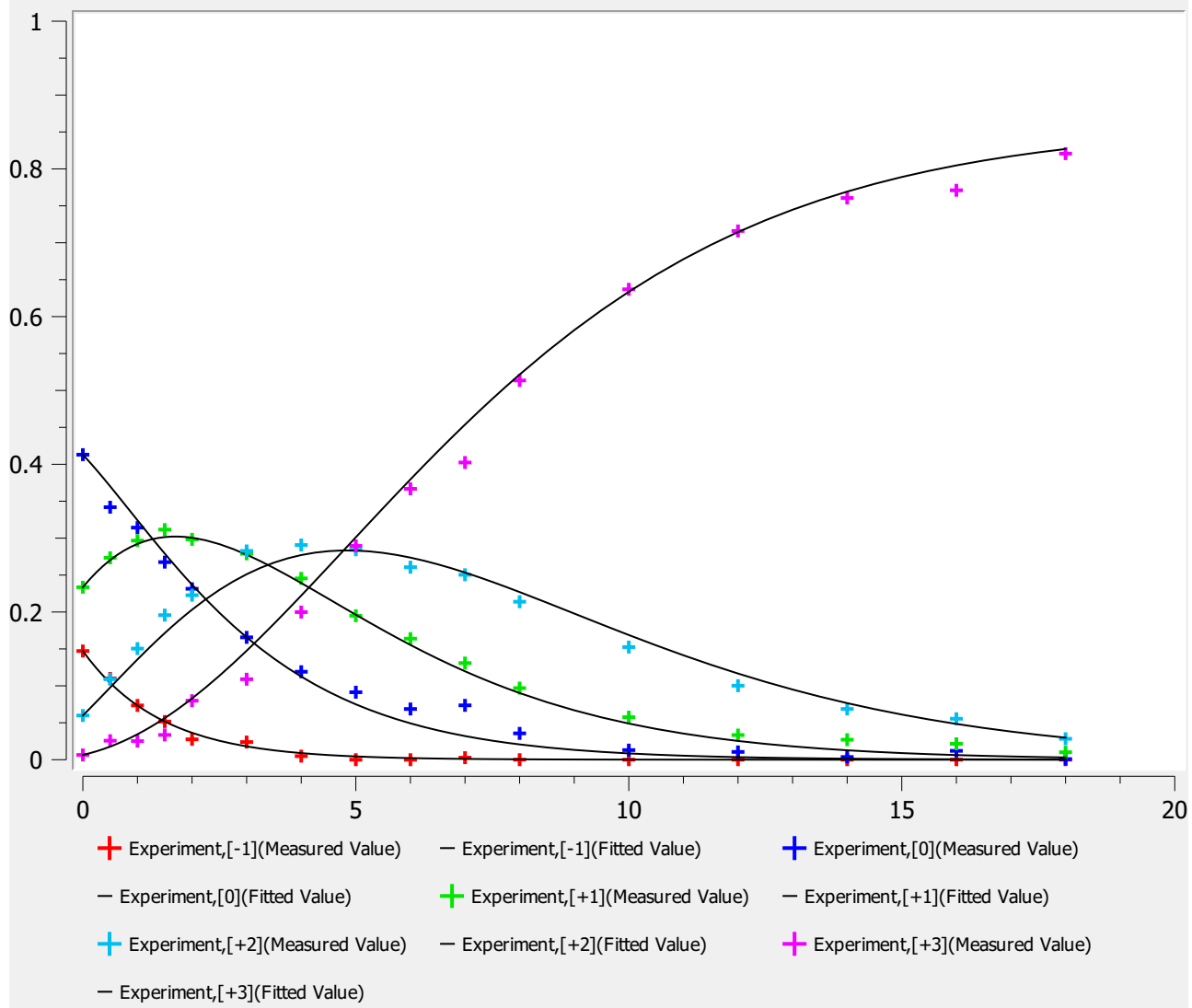

Supplement: Supplementary file 2 — Supplementary Data Set 1. Output files for kinetic analysis. [file 41565_2021_1021_MOESM2_ESM.zip › Supplementary_Data_Set_1_COPASI_Output/QY-1-REPORT.pdf]
